# Electrophilic Trapping of Semibenzenes

**DOI:** 10.1021/acs.joc.2c01331

**Published:** 2022-09-12

**Authors:** Cosimo Boldrini, Marta Castiñeira Reis, Syuzanna R. Harutyunyan

**Affiliations:** Stratingh Institute for Chemistry, University of Groningen, Nijenborgh 4, 9747 AG Groningen, The Netherlands

## Abstract

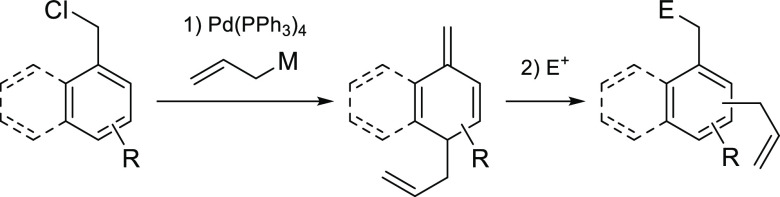

In this work, we demonstrate how allylative dearomatization of benzyl chlorides can provide direct access to a variety of semibenzenes. These scaffolds behave as highly reactive nucleophiles in the presence of carbocations. In addition, semibenzenes are susceptible to intramolecular rearrangements rendering a broad scope of functionalized arenes. An analysis of this new reactivity is reported, as well as the rationale behind the observed intramolecular reorganizations.

## Introduction

**Scheme 1:**
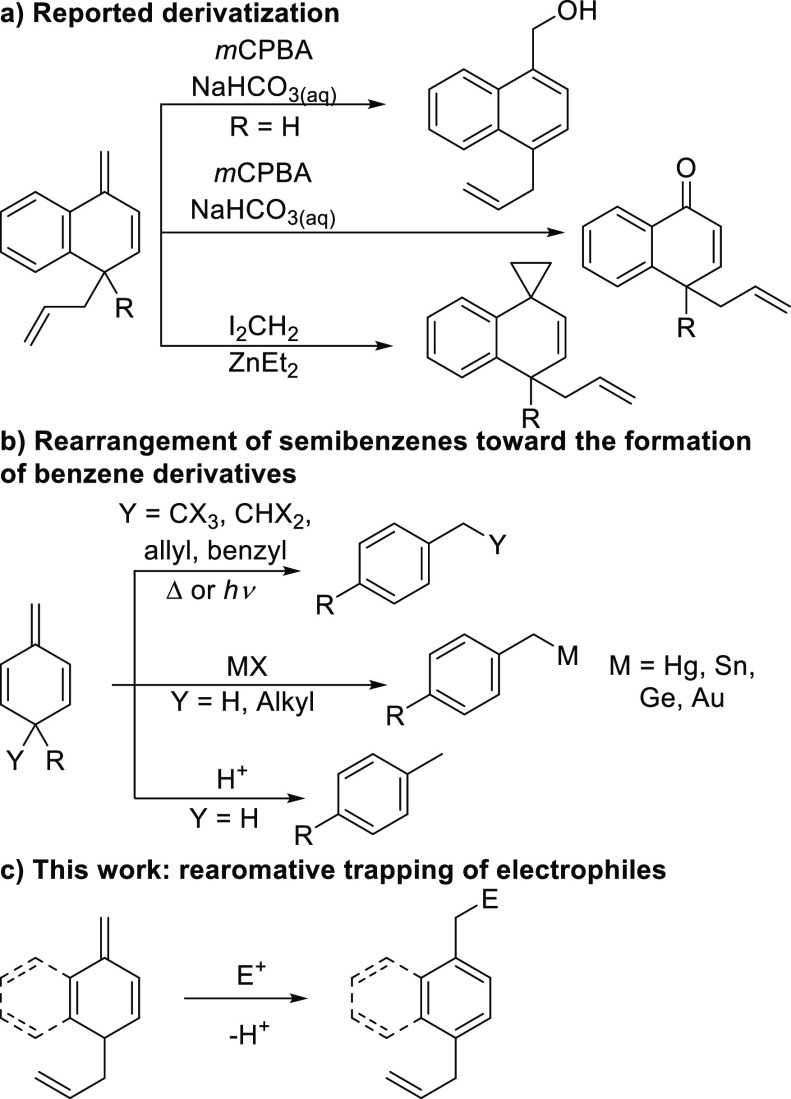
Transformations Involving Semibenzenes

Semibenzenes (3-methylenecyclohexa-1,4-dienes) are a class of unstable compounds often overlooked by chemists since they are challenging to synthesize and work with.^1−4^ However, in 2001, seminal work by Yamamoto et al. provided a remarkable turnaround in this regard, showing that the synthesis of mono- and di-substituted allylated semibenzenes is rather straightforward via Pd-catalyzed dearomative allylation of benzylic halides.^5^ Various modified and improved procedures have been reported since,^6^ but synthetic applications of semibenzenes remain scarce to this day. The only examples were reported by the group of Yamaguchi (Scheme 1a), namely, a cyclopropanation and an oxidation of the external double bond of semibenzenes, leading to the corresponding alcohols or α,β-unsaturated carbonyls.^6c,6d^

The intrinsic instability of semibenzenes is caused by their avidity to reorganize in order to recover aromaticity. The most common reorganization is the 1,5-shift of the substituents at the sp^3^ carbon to the benzylic position—namely, allyl, benzyl, or −CX_3_ (X = Cl, Br) substituents—with concomitant rearomatization of the molecule (Scheme 1b).^1,7^ The mechanism of this structural reorganization has been extensively investigated in the past, and most studies support a radical pathway.^8^ The only reported non-radical reaction is a sigmatropic rearrangement of propargyl substituted semibenzene to allenyl benzenes.^9^ Alternatively, it has also been shown that non-fully substituted semibenzenes quickly react in the presence of acids to yield the rearomatized compounds (by elimination of H^+^ from the sp^3^ carbon).^5,6,10^ In the 1980s, an analogous rearomatization was reported by the group of Reutov (Scheme 1b),^11^ who found that semibenzenes would form the corresponding benzylic organometallic reagent upon reaction with transition-metal salts (mainly Hg, but also Sn, Ge, and Au), in a so-called aromative metalation.^11,12^

Interestingly, it has been shown that upon reaction with a mixture of HgCl_2_ and HgO semibenzenes with a quaternary sp^3^ carbon undergo aromative metalation, yielding the rearomatized benzyl organometallic compounds, with a concomitant shift of one of the sp^3^ carbon substituents (no shift selectivity was observed in the reported compounds).^13^

Recently, we reported an alternative route to access semibenzenes, namely, a Pd-catalyzed dearomative allylation using Grignard.^6e^ This led us to commence detailed investigations into the largely unexplored reactivity of semibenzenes and their potential applications (Scheme 1c).

## Results and Discussion

During our initial studies, we discovered that it is possible to trigger rearomatization of semibenzenes with a quaternary sp^3^ carbon upon treatment with Brønsted or Lewis acids (e.g., TsOH, AcOH, FeCl_3_, MgCl_2_) (Table 1). This new protocol involves an initial palladium-catalyzed dearomatization of a phenyl or naphtyl core and a subsequent rearomatization/migration promoted by the acid. We found that the selectivity of the second step, that is, the shift of one of the groups at the sp^3^ center, is dependent on the nature of the substituents. For example, for R = alkyl, only the allyl group migrates, forming **3** (Table 1, entries 1 and 2). Interestingly, for **3b** (R = Et), a shift of the alkyl group to either the ortho or meta position was observed, with moderate selectivity toward the former. In the presence of a phenyl substituent (entry 4), the reaction gives a mixture of **3** and **4**, whereas high selectivity toward **4** is observed with benzyl and *p*-OMePh as substituents (entries 3 and 5). These results point to a strong sensitivity of the regioselectivity of the migration to the nature of the substituents at the sp^3^ center.

**Table 1:**
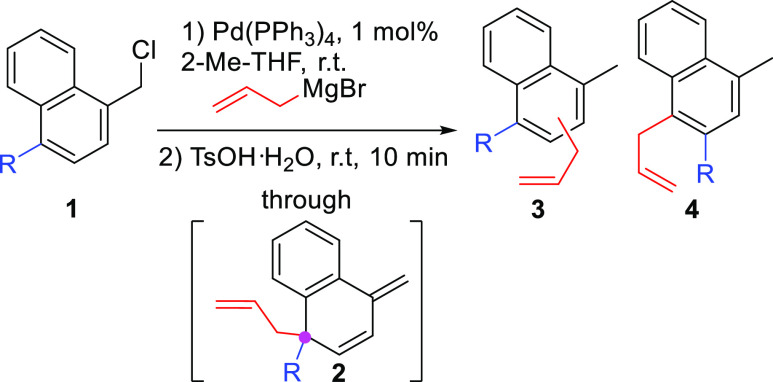
Acid-Promoted Rearomative Shift of Semibenzenes^a^

Entry	R	yield (%)^b^	3:4
1	Me—**1a**	64	100:0
2	Et—**1b**	84	100^c^:0
3	Bn—**1c**	72	<5:>95
4	Ph—**1d**	68^d^	67:33
5	*p*-OMe-Ph—**1e**	70	<5:>95

a General reaction conditions, **1** (0.30 mmol, 1.0 equiv), allylMgBr (1.0 M in Et_2_O, 0.36 mmol, 1.2 equiv), Pd(PPh_3_)_4_ (1 mol %), 2-Me-THF (1 mL), 15 min at r.t., then TsOH·H_2_O (0.60 mmol, 2.0 equiv), 10 min at r.t.

b Isolated yield.

c 85:15 ratio of *o*/*m* allyl (referring to the original position of R).

d Overall NMR yield.

**Scheme 2:**
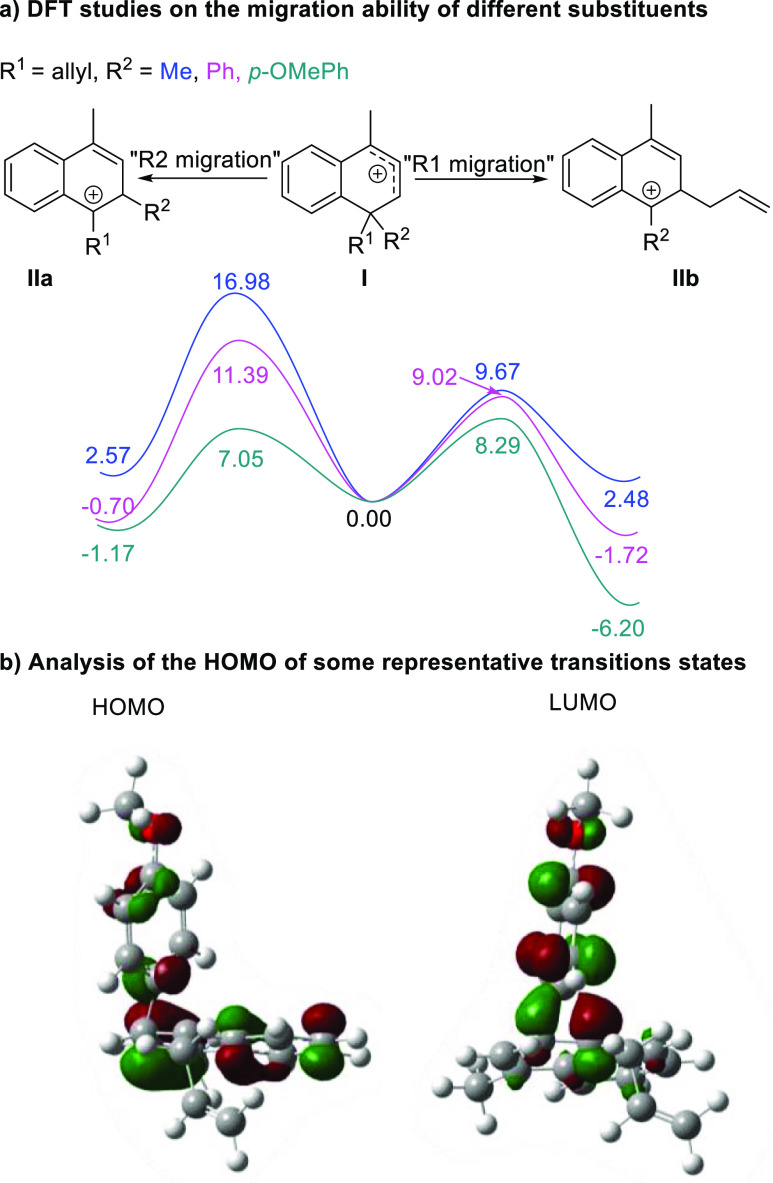
DFT Analysis of the Migration Step (a) Computed energy profiles for the migration of the different substituents at the tertiary center of semibenzene. (b) Cartoon of the HOMO and LUMO orbitals obtained for the transition state structure corresponding to the migration of the *p*-OMe-Ph substituent.

DFT studies indicate that the regioselectivity is determined by the ability of the migrating group to stabilize a charge deficit at the transition state structure (Scheme 2a). Note that in the transition state structure, the LUMO is mainly localized on the migrating group (Scheme 2b). Hence, the better the ability of the substituent to stabilize a positive charge in the migrating carbon (alkyl < phenyl < allyl < benzyl < *p*-OMe-Ph), the greater their predisposition to migrate.

The reaction we focused on in these initial studies (Table 1) is promoted by a proton, which acts as an electrophile and engages in an acid–base reaction with semibenzene. We envisioned that other electrophiles could also interact with the semibenzene core, potentially uncovering new roles and uses of these compounds.

First, we explored a series of soft electrophiles (e.g., aldehydes, acyl halides, anhydrides, etc.), but no conversion to the trapping product was observed. Then, we moved to exploring harder electrophiles such as carbocations.

The first carbocations we explored were tritylium cations (Ph_3_CX), which have found applications in electrophilic aromatic substitutions but are mostly used as hydride abstraction reagents, especially in rearomatization reactions.^14−17^ To our delight, this did result in the formation of the desired product (Table 2). Since the presence of protons in the reaction mixture cannot be avoided, the reaction of the nucleophile with the tritylium cations (to form **5f**) has to compete with protons (to form **6f**).^18^ During optimization of the reaction conditions, we noted that the nature of the solvent plays a crucial role in the reaction outcome (Table 2, entries 1–5), ranging from really poor selectivity with solvents such as THF and toluene to good selectivity when employing MeCN. Moreover, the use of an aprotic polar solvent is of utmost importance since protic solvents (e.g., EtOH) react with the electrophile. On the contrary, apolar solvents are incapable of solubilizing the substrate. We also explored the effect of counterions on the reaction outcome (entries 6 and 7), confirming that tetrafluoroborate provides the best selectivity. Reverse addition of the semibenzenes to a solution of the electrophile (entry 8) led to a further increase in selectivity (up to 95:5). Unfortunately, increasing the equivalents of the electrophile from 1.1 to 1.5 (entry 9) and lowering the temperature to 0 °C (entry 10) were not beneficial.

**Table 2:**
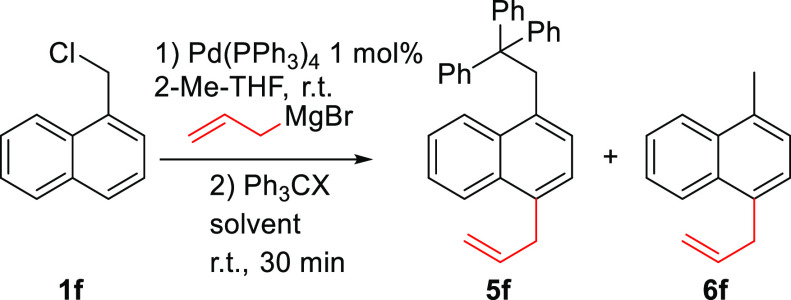
Screening of Conditions for the Trapping of Semibenzenes with Tritylium Cations^a^

entry	X	solvent	5:6 ratio
1	BF_4_^–^	CH_2_Cl_2_	15:85
2	BF_4_^–^	THF	5:95
3	BF_4_^–^	toluene	>1:<99
4	BF_4_^–^	acetone	10:90
5	BF_4_^–^	MeCN	80:20
6	PF_6_^–^	MeCN	55:45
7	SnCl_5_^–^	MeCN	33:66
8^b^	BF_4_^–^	MeCN	95:5
9^c^	BF_4_^–^	MeCN	95:5
10^d^	BF_4_^–^	MeCN	50:50

a General reaction conditions, **1f** (0.30 mmol, 1.0 equiv), allylMgBr (1.0 M in Et_2_O, 0.36 mmol, 1.2 equiv), Pd(PPh_3_)_4_ (1 mol %), 2-Me-THF (1 mL), 15 min at r.t., then Ph_3_CX (0.33 mmol, 1.1 equiv), in the specified solvent, 30 min at r.t.

b Addition of the dearomatized compound to a solution of the electrophile.

c 1.5 equiv of the electrophile.

d At 0 °C.

Following these encouraging results, we evaluated two more electrophiles, namely, tropylium tetrafluoroborate^19^ and 1,3-benzodithiolylium tetraborate.^20^ For tropylium tetrafluoroborate, we found that once again, the solvent is critical for the reaction outcome, with DMF yielding full conversion of the semibenzene and full selectivity toward the trapped product in less than 30 min, whereas MeCN, acetone, and CH_2_Cl_2_ gave very poor selectivity (Table S1). We chose 1,3-benzo dithiolylium tetrafluoroborate as the next electrophile since a similar reactivity to tropylium tetrafluoroborate has been reported for this carbocation,^19d^ making it a promising candidate to further increase the scope of this newly discovered transformation. For this electrophile, we found that acetone as a solvent provides the best results, allowing for full selectivity toward the trapping product (Table S2).

Having optimized the conditions for the trapping of each of these electrophiles, we moved to evaluate the effect of the nature of the substituents in the aromatic ring (Table 3). Trapping of a naphthalene core (entry 1) proceeds in good yield with all three electrophiles. Taking 1,3-benzo dithiolylium tetrafluoroborate as a benchmark, we looked at the effect of increased bulkiness near the reacting center (entry 2, substitution at the 2-position is usually detrimental for the dearomatization step and could also play a role in the trapping) and the presence of an electron-rich ring (entry 3, as these electrophiles could perform electrophilic aromatic substitution on such activated rings). In both cases, the desired trapping product was obtained in good overall yield. Substitution at the benzylic position, however, proved to have a bigger impact on the reaction outcome. For instance, in the presence of a phenyl group (entry 4), the yield is significantly reduced, likely due to sterics. On the other hand, the presence of linear substituents (entries 5 and 6) has smaller to no impact on the overall yields. The reaction of these compounds with tritylium tetrafluoroborate renders a mixture of atropoisomers.

**Table 3:**
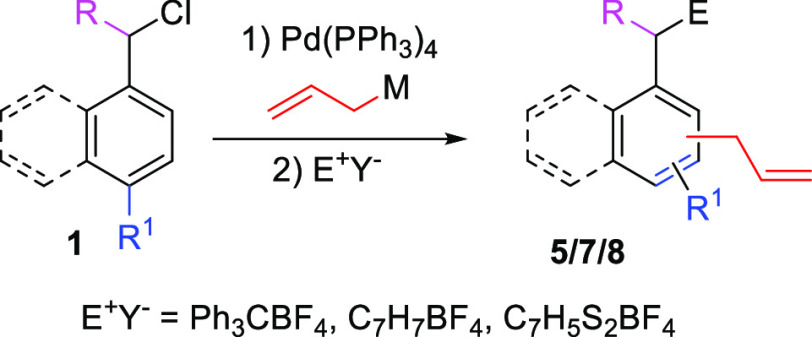

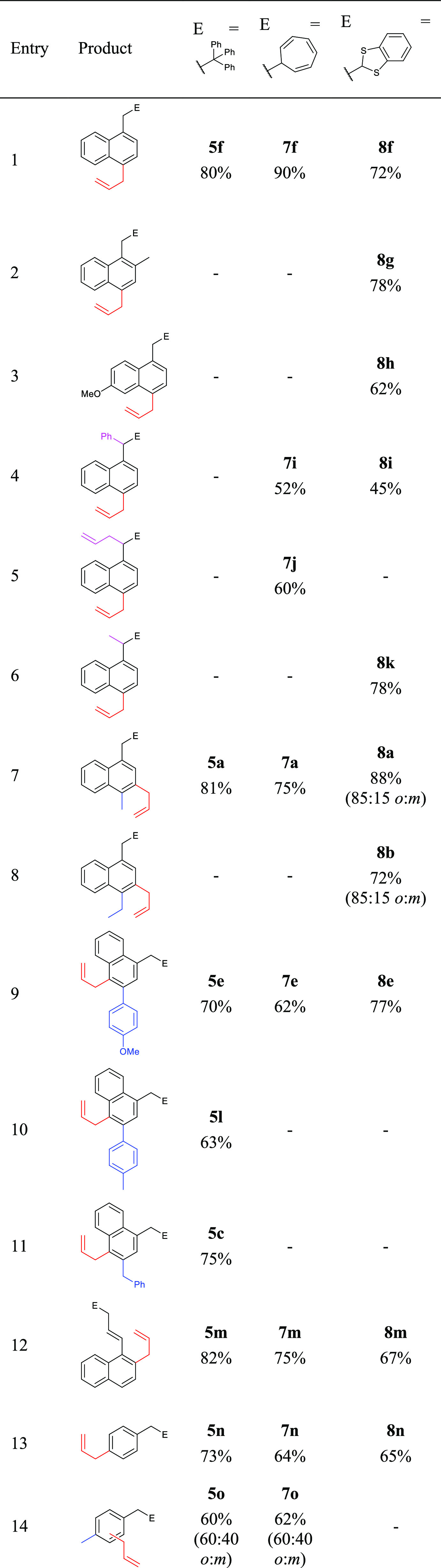
Tapping Results with Various Electrophiles^1^

1 Detailed experimental conditions are given in the Supporting Information. Reported yields are isolated yields over two steps. **1n** and **1o** were dearomatized employing allylSnBu_3_, and all other substrates were dearomatized with allylMgBr.

To our delight, semibenzenes derived from substituted (R^1^ ≠ H) benzylic substrates also yielded the trapping product. For these substrates, the concomitant shift of one of the substituents is observed in the trapping process. Specifically, for R^1^ = *p*-Me (entry 7), the preferential shift of the allyl group over the alkyl was observed. Interestingly, full regioselectivity toward the migration to the ortho position was observed on **5a** and **7a**, while a mixture of regioisomers was obtained in the reaction with 1,3-benzo dithiolylium tetrafluoroborate (**8a**), revealing that the nature of the electrophile also influences the regioselectivity of the process. The effect of the electrophile is more pronounced than that of the length of the alkyl chain. We observed that the presence of a longer and more electron-donating chain (entry 8, R^1^ = Et) had no beneficial effect on the shift selectivity. As expected, when aryl (Ar) *p*-substituted compounds are explored (entries 9 and 10), the migration of the Ar ring is observed preferentially. Likewise, for **5c**, we observed the shift of the benzyl group.

At this point, we wondered whether the newly discovered reactivity was also compatible with other aromatic cores. Both a conjugated system (entry 12) and a benzene ring (entry 13) were explored to this end. We obtained the expected trapping products in good, albeit slightly lower, yields. Moreover, we observed that the shift of the allyl occurs also on benzene cores (entry 14), but with almost no *o*/*m* selectivity. These results show that the reactivity presented here is not limited to dearomatized naphthalene structures but is characteristic of semibenzenes in general.

**Scheme 3:**
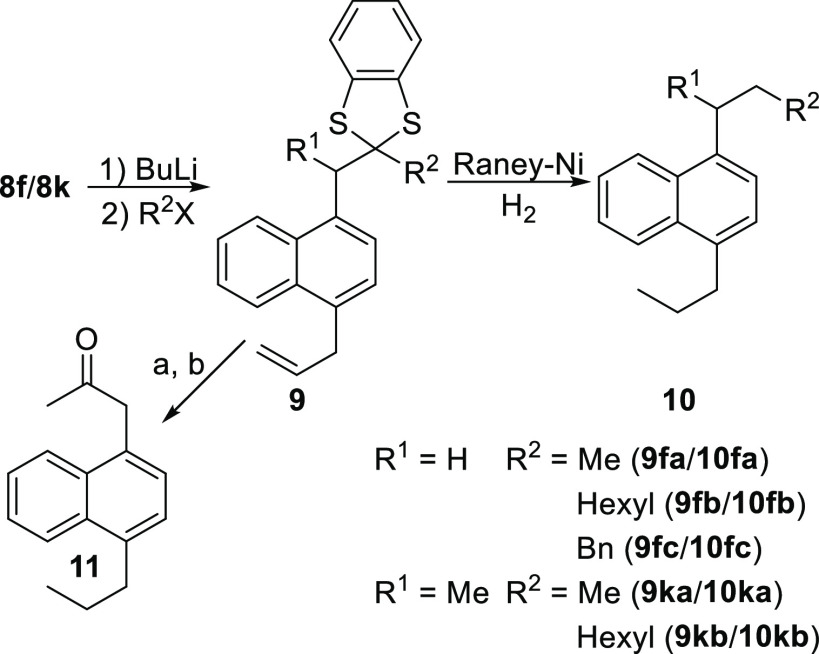
Alkylation and Removal of the Thioacetal Group (a) Pd/C, H_2_, r.t, overnight. (b) HgO, HBF_4_ (48% in H_2_O), r.t., 30 min.

Finally, the reaction with 1,3-benzo dithiolylium tetrafluoroborate allows for the introduction of a versatile functional group, which can act as an intermediate toward more complex scaffolds.^21,22^ An example of this is shown in Scheme 3, where we demonstrate the versatility of a dithiane-naphthalene core. Specifically, **8f** and **8k** were subjected to lithiation with *n*BuLi, followed by the addition of alkyl and benzyl electrophiles, yielding the alkylated (**9**) products in good yields. The dithiane can then be easily removed by Raney-Ni/H_2_, yielding alkanes **10**. Alternatively, **9fa** can evolve via oxidative deprotection toward a ketone. Unfortunately, this proved to be more challenging. Reaction of **9fa** with HgO yielded a complex product mixture, GC–MS analysis of which revealed the formation of only traces of the desired product. Under the assumption that the problem could arise from the presence of a terminal double bond, **9fa** was reduced by Pd/C–H_2_, prior to the deprotection with HgO, rendering **11** in 83% yield, allowing for a modular synthesis of homo-naphthyl carbonyls.

## Conclusions

In summary, we have reported a new protocol toward functionalized aromatic cores that exploits the nucleophilicity of in situ generated semibenzenes. We also show how the accessed naphthalenes can easily be derivatized into other scaffolds of interest for the synthetic community, such as naphthyl carbonyls.^22^

## Experimental Section

### General Information

All reactions using oxygen- and/or moisture-sensitive materials were carried out with anhydrous solvents under a nitrogen atmosphere using standard Schlenk techniques. Flash column chromatography was performed using Merck 60 Å 230–400 mesh silica gel. Thin-layer chromatography was performed using 0.25 mm E. Merck silica plates (60F-254). Components were visualized by UV light and permanganate staining. Reactions were monitored by TLC. NMR data (^1^H at 400 MHz; ^13^C at 101 MHz) was collected on a Varian VXR400 machine equipped with a 5 mm z-gradient broadband probe. Chemical shifts are reported in parts per million (ppm) relative to the residual solvent peak (CDCl_3_, ^1^H: 7.26 ppm; ^13^C: 77.2 ppm). Coupling constants are reported in Hertz. Multiplicity is reported with the usual abbreviations (s: singlet, d: doublet, dd: doublet of doublets, t: triplet, q: quadruplet, m: multiplet). Exact mass spectra were recorded on an LTQ Orbitrap XL apparatus with ESI or a 4800 MALDI TOF/TOF analyzer; exact masses are given for previously unreported compounds. The compounds here reported are known to fragment upon ionization to form rather stable ions.^23^ For this reason, molecular ions are not always detectable. For these molecules, however, the formed fragments give clear and intense signals in mass analysis. Hence, the detection of these fragments together with NMR analysis allows for an unequivocal product characterization. Unless otherwise indicated, reagents and substrates were purchased from commercial sources and used as received. Solvents not required to be dry were purchased as technical grade and used as received. Dry solvents were freshly collected from a dry solvent purification system prior to use. Inert atmosphere experiments were performed with standard Schlenk techniques with dried (P_2_O_5_) nitrogen gas. Grignard reagents and allylSnBu_3_ were purchased from Sigma-Aldrich. 1-(Chloromethyl)-naphthalene (**1f**), 4-methyl-1-(chloromethyl)-naphthalene (**1a**), 1-(chloromethyl)-2-methyl-naphthalene (**1g**), benzyl chloride (**1n**), and *p*-methyl-benzyl chloride (**1o**) were purchased from Sigma-Aldrich; other benzylic substrates were prepared following literature methods.^6e^

### General Procedure for the Trapping with TsOH **3a–e**

To an oven-dried Schlenk were added the substrate (0.30 mmol, 1.0 equiv), Pd(PPh_3_)_4_ (1 mol %), and dry 2-Me-THF (1 mL), and the mixture was stirred for 5 min under a nitrogen atmosphere. Allyl magnesium bromide (375 μL, 1.0 M in Et_2_O, 1.25 equiv) was added at once, and the mixture was stirred at r.t. until completion (TLC check, finished in 15 min). After complete consumption of the substrate, *p*-TsOH·H_2_O (0.60 mmol, 2.0 equiv) was added and the mixture was stirred for an additional 10 min. Then, a saturated solution of NaHCO_3_ (10 mL) was added, and the aqueous layer was extracted with Et_2_O (10 mL × 3). The combined organic layers were dried over Na_2_SO_4_, filtered, and concentrated under reduced pressure to give the crude of the rearomatized compound, which was then purified by column chromatography (silica gel) using pentane or a mixture of pentane and CH_2_Cl_2_ as an eluent.

#### 2-Allyl-1,4-dimethylnaphthalene (**3a**)^24^

The crude compound was purified by column chromatography (SiO_2_, pentane), giving **3a** as a colorless oil (37.7 mg, 64% yield). ^1^H NMR (400 MHz, CDCl_3_): δ 2.60 (s, 3H), 2.67 (s, 3H), 3.58 (d, *J* = 6.2 Hz, 2H), 4.96–5.10 (m, 2H), 5.97–6.08 (m, 1H), 7.16 (s, 1H), 7.47–7.57 (m, 2H), 7.98–8.01 (m, 1H), 8.07–8.10 (m, 1H) ppm. ^13^C{^1^H} NMR (101 MHz, CDCl_3_): δ 14.2, 19.3, 38.6, 115.3, 124.5 (2×C), 124.6, 125.4, 129.3, 129.4, 131.6, 132.0, 133.2, 134.3, 137.0 ppm.

#### 2-Allyl-1-ethyl-4-methylnaphthalene (**3ba**) + 2-Allyl-4-ethyl-1-methylnaphthalene (**3bb**)

The crude compounds were purified by column chromatography (SiO_2_, pentane), giving a non-isolable mixture of **3ba** and **3bb** (8:2 regioisomer ratio) as a colorless oil (53.0 mg, 84% yield). ^1^H NMR (400 MHz, CDCl_3_): δ 1.32 (t, *J* = 7.5 Hz, 3H, major), 1.40 (t, *J* = 7.5 Hz, 3H, minor), 2.62 (s, 3H, minor), 2.68 (s, 3H, major), 3.07–3.16 (m, 2H minor + 2H major), 3.56–3.63 (m, 2H minor + 2H major), 4.99–5.13 (m, 2H minor + 2H major), 6.00–6.14 (m, 1H minor + 1H major), 7.17–7.21 (m, 1H minor + 1H major), 7.48–7.59 (m, 2H minor + 2H major), 7.99–8.14 (m, 2H minor + 2H major) ppm. ^13^C{^1^H} NMR (101 MHz, CDCl_3_): δ 14.3 (minor), 15.3 (minor), 15.4 (major), 19.4 (major), 21.2 (major), 25.9 (minor), 37.8 (major), 38.7 (minor), 115.4 (minor), 115.5 (major), 124.1 (minor), 124.4 (major), 124.6 (major), 124.7 (minor × 2 + 1 major), 125.4 (minor), 125.5 (major), 127.6 (minor), 129.3 (major), 129.4 (minor), 130.8 (minor), 132.0 (major), 132.1 (major), 132.2 (major), 133.4 (minor), 133.7 (major), 134.4 (minor), 135.6 (major), 137.0 (minor), 137.6 (major), 138.1 (minor) ppm. HRMS (ESI^+^, *m*/*z*): calcd for C_16_H_17_ [M – H]^+^ 209.1323; found, 209.1325.

#### 1-Allyl-2-benzyl-4-methyl-naphthalene (**4c**)

The crude compound was purified by column chromatography (SiO_2_, pentane), giving **4c** as a colorless oil (58.8 mg, 72% yield). ^1^H NMR (400 MHz, CDCl_3_): δ 2.67 (s, 3H), 3.85 (d, *J* = 5.6 Hz, 2H), 4.19 (s, 2H), 4.90–4.98 (m, 1H), 5.01–5.06 (m, 1H), 5.96–6.07 (m, 1H), 7.15–7.23 (m, 4H), 7.26–7.32 (m, 2H), 7.49–7.57 (m, 2H), 8.00–8.10 (m, 2H) ppm. ^13^C{^1^H} NMR (101 MHz, CDCl_3_): δ 19.4, 32.5, 39.2, 115.6, 124.6, 124.8, 124.9, 125.7, 126.0, 128.4 (2×C), 128.7 (2×C), 130.0, 131.2, 132.0, 132.8, 132.9, 135.7, 136.6, 141.0 ppm. HRMS (ESI^+^, *m*/*z*): calcd for C_21_H_21_ [M + H]^+^ 273.1643; found, 273.1644.

#### 1-Allyl-2-(4-methoxyphenyl)-4-methylnaphthalene (**4d**)

The crude compound was purified by column chromatography (SiO_2_, pentane/CH_2_Cl_2_ 95:5), giving **4d** as a colorless oil (60.6 mg, 70% yield). ^1^H NMR (400 MHz, CDCl_3_): δ 2.71 (s, 3H), 3.74–3.79 (m, 2H), 3.88 (s, 3H), 4.84–4.91 (m, 1H), 5.06–5.11 (m, 1H), 6.07–6.18 (m, 1H), 6.98 (d, *J* = 8.8 Hz, 2H), 7.28 (s, 1H), 7.35 (d, *J* = 8.8 Hz, 2H), 7.51–7.58 (m, 2H), 8.03–8.12 (m, 2H) ppm. ^13^C{^1^H} NMR (101 MHz, CDCl_3_): δ 19.4, 33.7, 55.3, 113.4 (2×C), 115.9, 124.6, 125.1, 125.8 (2xC), 129.5, 130.3 (2×C), 130.4, 132.2, 132.5, 132.6, 134.9, 138.1, 139.0, 158.6 ppm. HRMS (ESI^+^, *m*/*z*): calcd for C_21_H_21_O [M + H]^+^ 289.1586; found, 289.1583.

### General Procedure for the Trapping with Ph_3_CBF_4_

#### Method A **5a–m**

To an oven-dried Schlenk were added the substrate (0.30 mmol, 1.0 equiv) and Pd(PPh_3_)_4_ (1 mol %); then dry 2-Me-THF (1 mL) was added, and the mixture was stirred for 5 min under a nitrogen atmosphere. Allyl magnesium bromide (375 μL, 1.0 M in Et_2_O, 1.25 equiv) was added at once, and the mixture was stirred at r.t. until completion (TLC check, finished in 15 min). After complete consumption of the substrate, pentane (20 mL) was added to precipitate out the salts and the suspension was filtered through a plug of Celite. Evaporation of the solvent yielded the crude dearomatized product, which was dissolved in CH_2_Cl_2_ (0.5 mL) and added dropwise to a stirred solution of Ph_3_CBF_4_ (0.33 mmol, 1.1 equiv) in MeCN (6 mL) at r.t. The mixture was stirred for 30 min at r.t. Then, the solvent was evaporated under reduced pressure, and the crude product was purified by column chromatography (silica gel) using a mixture of pentane and CH_2_Cl_2_ as an eluent.

#### 2-Allyl-1-methyl-4-(2,2,2-triphenylethyl)naphthalene (**5a**)

The crude compound was purified by column chromatography (SiO_2_, pentane/CH_2_Cl_2_ 90:10), giving **5a** as a white solid (106.6 mg, 81% yield). ^1^H NMR (400 MHz, CDCl_3_): δ 2.50 (s, 3H), 3.22 (d, *J* = 6.3 Hz, 2H), 4.41 (s, 2H), 4.60–4.77 (m, 1H), 4.80–4.90 (m, 1H), 5.58–5.69 (m, 1H), 6.87 (s, 1H), 7.13–7.27 (m, 16H), 7.37 (t, *J* = 8.5 Hz, 1H), 7.52 (d, *J* = 8.5 Hz, 1H), 7.96 (d, *J* = 8.5 Hz, 1H) ppm. ^13^C{^1^H} NMR (101 MHz, CDCl_3_): δ 14.2, 38.4, 41.6, 57.9, 115.2, 123.7, 124.2, 124.7, 125.9 (3×C), 127.6 (6xC), 129.3, 129.9 (6×C), 130.6, 131.4, 132.0, 132.4, 132.8, 133.6, 136.6, 146.8 (3×C) ppm. HRMS (ESI^+^, *m*/*z*): fragmentation observed. Calcd for C_19_H_15_ [M]^+^ 243.1174; found, 243.1174. Calcd for C_15_H_15_ [M]^+^ 195.1174; found, 195.1173.

#### 1-Allyl-2-(benzyl)-4-(2,2,2-triphenylethyl)naphthalene (**5c**)

The crude compound was purified by column chromatography (SiO_2_, pentane/CH_2_Cl_2_ 90:10), giving **5c** as a white solid (115.8 mg, 75% yield). ^1^H NMR (400 MHz, CDCl_3_): δ 3.71 (d, *J* = 5.4 Hz, 2H), 3.80 (s, 2H), 4.44 (s, 2H), 4.83 (dd, *J*_1_ = 1.7 Hz, *J*_2_ = 17.2 Hz, 1H), 4.96 (dd, *J*_1_ = 1.7 Hz, *J*_2_ = 10.2 Hz, 1H), 5.85–5.97 (m, 1H), 6.72–6.79 (m, 2H), 7.02 (s, 1H), 7.08–7.24 (m, 19H), 7.32 (t, *J* = 7.3 Hz, 1H), 7.51 (d, *J* = 8.5 Hz, 1H), 7.90 (d, *J* = 8.5 Hz, 1H) ppm. ^13^C{^1^H} NMR (101 MHz, CDCl_3_): δ 32.4, 39.5, 41.4, 57.9, 115.4, 123.7, 124.3, 124.5, 124.8, 125.6, 125.9 (3×C), 127.6 (6×C), 128.2 (2×C), 128.6 (2×C), 129.8 (6×C), 131.1, 132.2, 132.4, 132.8 (2×C), 135.1, 136.4, 140.7, 146.6 (3×C) ppm. HRMS (ESI^+^, *m*/*z*): fragmentation observed. Calcd for C_19_H_15_ [M]^+^ 243.1174; found, 243.1175. Calcd for C_21_H_19_ [M]^+^ 271.1487; found, 271.1494.

#### 1-Allyl-2-(4-methoxyphenyl)-4-(2,2,2-triphenylethyl)naphthalene (**5e**)

The crude compound was purified by column chromatography (SiO_2_, pentane/CH_2_Cl_2_ 80:20), giving **5e** as a white solid (113.0 mg, 71% yield). ^1^H NMR (400 MHz, CDCl_3_): δ 3.65–3.70 (m, 2H), 3.82 (s, 3H), 4.43 (s, 2H), 4.80 (dd, *J*_1_ = 1.7 Hz, *J*_2_ = 17.3 Hz, 1H), 5.06 (dd, *J*_1_ = 1.7 Hz, *J*_2_ = 10.4 Hz, 1H), 6.04–6.15 (m, 1H), 6.7–6.84 (m, 4H), 7.02 (s, 1H), 7.08–7.20 (m, 16H), 7.34 (t, *J* = 8.8 Hz, 1H), 7.45 (d, *J* = 8.8 Hz, 1H), 7.94 (d, *J* = 8.8 Hz, 1H) ppm. ^13^C{^1^H} NMR (101 MHz, CDCl_3_): δ 36.3, 44.2, 57.9, 60.8, 115.7 (2×C), 118.6, 126.4, 127.2, 127.6, 128.1, 128.5 (3×C), 130.3 (6×C), 132.6 (6×C), 132.8, 133.1 (2×C), 134.3, 134.9, 135.3, 135.7, 137.3, 140.5, 140.8, 149.3 (3×C), 161.1 ppm. HRMS (ESI^+^, *m*/*z*): calcd for C_40_H_35_O [M + H]^+^ 531.2682; found, 531.2718.

#### 1-Allyl-4-(2,2,2-triphenylethyl)naphthalene (**5f**)

The crude compound was purified by column chromatography (SiO_2_, pentane/CH_2_Cl_2_ 95:5), giving **5f** as a white solid (101.9 mg, 80% yield). ^1^H NMR (400 MHz, CDCl_3_): δ 3.73 (d, *J* = 6.2 Hz, 2H), 4.45 (s, 2H), 4.97–5.08 (m, 2H), 6.00–6.11 (m, 1H), 6.95 (d, *J* = 7.4 Hz, 1H), 7.01 (d, *J* = 7.4 Hz, 1H), 7.10–7.18 (m, 10H), 7.20–7.25 (m, 6H), 7.30–7.36 (m, 1H), 7.49 (d, *J* = 8.7 Hz, 1H), 7.92 (d, *J* = 8.5 Hz, 1H) ppm. ^13^C{^1^H} NMR (101 MHz, CDCl_3_): δ 39.9, 44.0, 60.7, 118.6, 126.6, 126.9, 127.2, 127.5, 127.9, 128.6 (3×C), 130.2 (6×C), 130.8, 132.5 (6×C), 134.3, 135.9, 136.5, 136.8, 139.8, 149.3 (3×C) ppm. HRMS (ESI^+^, *m*/*z*): fragmentation observed. Calcd for C_19_H_15_ [M]^+^ 243.1174; found, 243.1176 calcd for C_14_H_13_ [M]^+^ 181.1017; found, 181.1019.

#### 1-Allyl-2-(*p*-tolyl)-4-(2,2,2-triphenylethyl)naphthalene (**5l**)

The crude compound was purified by column chromatography (SiO_2_, pentane/CH_2_Cl_2_ 90:10), giving **5l** as a white solid (97.3 mg, 63% yield). ^1^H NMR (400 MHz, CDCl_3_): δ 2.37 (s, 3H), 3.66–3.72 (m, 2H), 4.45 (s, 2H), 4.79–4.86 (m, 1H), 5.05–5.10 (m, 1H), 6.05–6.16 (m, 1H), 6.80 (d, *J* = 8.0 Hz, 2H), 7.05 (s, 1H), 7.08 (d, *J* = 8.0 Hz, 2H), 7.11–7.24 (m, 16H), 7.33–7.38 (m, 1H), 7.48 (d, *J* = 8.6, 1H), 7.97 (d, *J* = 8.6, 1H) ppm. ^13^C{^1^H} NMR (101 MHz, CDCl_3_): δ 21.1, 33.7, 41.6, 58.1, 115.9, 123.8, 124.6, 124.9, 125.5, 125.9 (3×C), 127.6 (6×C), 128.3 (2×C), 129.3 (2×C), 130.0 (× 6C), 130.1, 131.6, 132.2, 132.7, 133.1, 136.2, 138.2 (2×C), 139.2, 146.6 (3×C) ppm. HRMS (ESI^+^, *m*/*z*): calcd for C_40_H_35_ [M + H]^+^ 515.2694; found, 515.2733.

#### (*E*)-2-Allyl-1-(4,4,4-triphenylbut-1-en-1-yl)naphthalene (**5m**)

The crude compound was purified by column chromatography (SiO_2_, pentane/CH_2_Cl_2_ 95:5), giving **5m** as a white solid (110.8 mg, 82% yield). ^1^H NMR (400 MHz, CDCl_3_): δ 3.36 (d, *J* = 6.0 Hz, 2H), 3.78 (d, *J* = 6.7 Hz, 2H), 4.89 (d, *J* = 17.1 Hz, 1H), 4.99 (d, *J* = 10.1 Hz, 1H), 5.71–5.90 (m, 2H), 6.70 (d, *J* = 16.3 Hz, 1H), 7.19–7.34 (m, 17H), 7.34–7.40 (m, 1H), 7.54 (d, *J* = 8.5 Hz, 1H), 7.64 (d, *J* = 8.5 Hz, 1H), 7.73 (d, *J* = 8.1 Hz, 1H) ppm. ^13^C{^1^H} NMR (101 MHz, CDCl_3_): δ 40.7, 48.0, 59.2, 118.1, 127.6, 128.2, 128.5, 128.7 (3×C), 129.5, 130.5 (2×C), 130.6 (6×C), 131.5, 132.1 (6×C), 134.9 (2×C), 137.0 (2×C), 140.1, 149.9 (3×C) ppm. (Missing peak carbon due to overlapping signals). HRMS (ESI^+^, *m*/*z*): calcd for C_35_H_31_ [M + H]^+^ 451.2423; found, 451.2420.

### General Procedure for the Trapping with Ph_3_CBF_4_

#### Method B **5n-oa/ob**

To an oven-dried Schlenk, Pd(PPh_3_)_4_ (10 mol %) was dissolved in CH_2_Cl_2_ (3 mL); then the substrate was added (0.30 mmol, 1.0 equiv), and the mixture was stirred for 5 min under a nitrogen atmosphere. AllylSnBu_3_ (0.30 mmol, 1.0 equiv) was added at once, and the mixture stirred at r.t. until completion (TLC checks). After complete consumption of the substrate, the solvent was evaporated and the crude was filtered through a plug of basic alumina (Ø = 1 cm, *h* ∼ 8–10 cm) using pentane as an eluent (∼100 mL). After evaporation of the solvent, the crude was dissolved in CH_2_Cl_2_ (0.5 mL) and added dropwise to a stirred solution of Ph_3_CBF_4_ (0.33 mmol, 1.1 equiv) in MeCN (6 mL) at r.t. The mixture was stirred for 30 min at r.t.; then the solvent was evaporated under reduced pressure, and the crude product was purified by column chromatography (silica gel) using a mixture of pentane and CH_2_Cl_2_ as an eluent.

#### (2-(4-Allylphenyl)ethane-1,1,1-triyl)tribenzene (**5n**)

The crude compound was purified by column chromatography (SiO_2_, pentane/CH_2_Cl_2_ 95:5), giving **5n** as a white solid (78.6 mg, 73% yield). ^1^H NMR (400 MHz, CDCl_3_): δ 3.25 (d, *J* = 6.5 Hz, 2H), 3.91 (s, 2H), 4.95–5.03 (m, 2H), 5.84–5.95 (m, 1H), 6.55 (d, *J* = 8.1 Hz, 2H), 6.80 (d, *J* = 8.1 Hz, 2H), 7.14–7.23 (m, 15H) ppm. ^13^C{^1^H} NMR (101 MHz, CDCl_3_): δ 42.3, 48.5, 61.1, 118.1, 128.5 (3×C), 130.1 (× 6C), 130.2 (2×C) 132.4 (6×C), 133.8 (2×C), 138.8, 140.2, 140.3, 149.3 (3×C) ppm. HRMS (ESI^+^, *m*/*z*): fragmentation observed. Calcd for C_19_H_15_ [M]^+^ 243.1174; found, 243.1175 calcd for C_10_H_11_ [M]^+^ 131.0861; found, 131.0857.

#### (2-(3-Allyl-4-methylphenyl)ethane-1,1,1-triyl)tribenzene (**5oa**)

The crude compound was purified by column chromatography (SiO_2_, pentane), giving **5oa** as a white solid (47.8 mg, 41% yield). ^1^H NMR (400 MHz, CDCl_3_): δ 2.17 (s, 3H), 3.10 (d, *J* = 6.3 Hz, 2H), 3.90 (s, 2H), 4.81–4.87 (m, 1H), 4.93–4.98 (m, 1H), 5.64–5.75 (m, 1H), 6.33 (s, 1H), 6.50 (d, *J* = 7.6 Hz, 1H), 6.80 (d, *J* = 7.6 Hz, 1H), 7.15–7.24 (m, 15H) ppm. ^13^C{^1^H} NMR (101 MHz, CDCl_3_): δ 18.8, 37.5, 46.0, 58.4, 115.3, 125.8 (3×C), 127.5 (6×C), 128.9, 129.1, 129.9 (6×C), 132.4, 133.8, 135.9, 136.5, 136.8, 146.7 (3×C) ppm. HRMS (ESI^+^, *m*/*z*): fragmentation observed. Calcd for C_19_H_15_ [M]^+^ 243.1174; found, 243.1170 calcd for C_11_H_13_ [M]^+^ 145.1174; found, 145.1012.

#### (2-(2-Allyl-4-methylphenyl)ethane-1,1,1-triyl)tribenzene (**5ob**)

The crude compound was purified by column chromatography (SiO_2_, pentane), giving **5ob** as a white solid (32.6 mg, 28% yield). ^1^H NMR (400 MHz, CDCl_3_): δ 2.22 (s, 3H), 2.46 (d, *J* = 6.3 Hz, 2H), 3.92 (s, 2H), 4.75–4.82 (m, 1H), 4.94–4.99 (m, 1H), 5.84–5.95 (m, 1H), 6.65–6.69 (m, 1H), 6.74–6.79 (m, 2H), 7.13–7.23 (m, 15H) ppm. ^13^C{^1^H} NMR (101 MHz, CDCl_3_): δ 20.9, 36.6, 40.8, 58.2, 115.3, 125.9 (3×C), 126.2, 127.5 (6×C), 129.7, 129.9 (6×C), 130.4, 133.8, 135.6, 137.4, 140.0, 146.6 (3×C) ppm. HRMS (ESI^+^, *m*/*z*): fragmentation observed. Calcd for C_19_H_15_ [M]^+^ 243.1174; found, 243.1169 calcd for C_11_H_13_ [M]^+^ 145.1174; found, 145.1010.

### General Procedure for the Trapping with Tropylium Tetrafluoroborate

#### Method C **7a–m**

To an oven-dried Schlenk were added the substrate (0.30 mmol, 1.0 equiv) and Pd(PPh_3_)_4_ (1 mol %); then dry 2-Me-THF (1 mL) was added, and the mixture was stirred for 5 min under a nitrogen atmosphere. AllylMgBr (375 μL, 1.0 M in Et_2_O, 1.25 equiv) was added at once, and the mixture was stirred at r.t. until the substrate was consumed completely (TLC check, finished in 15 min). Then, pentane (20 mL) was added to precipitate out the salts and the suspension was filtered through a plug of Celite. Evaporation of the solvent yielded the crude dearomatized product, which was dissolved in CH_2_Cl_2_ (0.5 mL) and added dropwise to a stirred solution of tropylium tetrafluoroborate (0.45 mmol, 1.5 equiv) in DMF (3 mL) at r.t. The mixture was stirred for 30 min at r.t.; then water (10 mL) and Et_2_O were added (10 mL), the mixture was stirred for 5 min, and the layers were separated. The aqueous layer was extracted with Et_2_O (10 mL × 2), and the combined organic layers were dried over Na_2_SO_4_, filtered, and concentrated under reduced pressure. The crude product was purified by column chromatography (SiO_2_) using a mixture of pentane and CH_2_Cl_2_ as an eluent.

#### 1-Allyl-4-(1-(cyclohepta-2,4,6-trien-1-yl)but-3-en-1-yl)naphthalene (**7a**)

The crude compound was purified by column chromatography (SiO_2_, pentane), giving **7a** as a white solid (64.4 mg, 75% yield). ^1^H NMR (400 MHz, CDCl_3_): δ 2.16–2.26 (m, 1H), 2.61 (s, 3H), 3.45 (d, *J* = 8.5 Hz, 2H), 3.60 (dt, *J*_1_ = 1.7 Hz, *J*_2_ = 6.2 Hz, 2H), 4.96–5.03 (m, 1H), 5.06–5.11 (m, 1H), 5.36 (dd, *J*_1_ = 4.5 Hz, *J*_2_ = 9.3 Hz, 2H), 5.98–6.10 (m, 1H), 6.15–6.22 (m, 2H), 6.60–6.68 (m, 2H), 7.21 (s, 1H), 7.43–7.54 (m, 2H), 7.98–8.02 (m, 1H), 8.07–8.11 (m, 1H) ppm. ^13^C{^1^H} NMR (101 MHz, CDCl_3_): δ 14.3, 36.2, 38.6, 39.2, 115.4, 124.0, 124.7, 124.8 (3×C), 125.4, 126.4 (2×C), 129.6, 130.1, 130.9 (2×C), 131.1, 133.5, 133.6, 134.1, 136.9 ppm. HRMS (ESI^+^, *m*/*z*): fragmentation observed. Calcd for C_7_H_7_ [M]^+^ 91.0548; found, 91.0540. Calcd for C_15_H_15_ [M]^+^ 195.1174; found, 195.1167.

#### 1-Allyl-4-(1′-(cyclohepta-2,4,6-trien-1-yl)ethyl)-2-(4′-methoxyphenyl)-naphthalene (**7e**)

The crude compound was purified by column chromatography (SiO_2_, pentane/CH_2_Cl_2_ 95:5), giving **7e** as a white solid (70.4 mg, 62% yield). ^1^H NMR (400 MHz, CDCl_3_): δ 2.21–2.30 (m, 1H), 3.48 (d, *J* = 7.8 Hz, 2H), 3.74–3.80 (m, 2H), 3.89 (s, 3H), 4.85–4.92 (m, 1H), 5.06–5.12 (m, 1H), 5.39 (dd, *J*_1_ = 5.5 Hz, *J*_2_ = 9.2 Hz, 2H), 6.07–6.24 (m, 3H), 6.59–6.67 (m, 2H), 7.00 (d, *J* = 8.5 Hz, 2H), 7.34 (s, 1H), 7.38 (d, *J* = 8.5 Hz, 2H), 7.46–7.55 (m, 2H), 8.02–8.12 (m, 2H) ppm. ^13^C{^1^H} NMR (101 MHz, CDCl_3_): δ 33.9, 36.3, 39.2, 55.3, 113.4 (2×C), 116.0, 124.1, 124.9 (2×C), 125.3, 125.8, 126.0, 126.3 (2×C), 129.7, 130.4 (2×C), 130.9 (2×C), 131.0, 131.7, 132.9, 134.2, 134.9, 138.0, 138.8, 158.7 ppm. HRMS (ESI^+^, *m*/*z*): calcd for C_28_H_27_O [M + H]^+^ 379.2017; found, 379.2056.

#### 1-Allyl-4-(cyclohepta-2,4,6-trien-1-ylmethyl)naphthalene (**7f**)

The crude compound was purified by column chromatography (SiO_2_, pentane) using pentane as an eluent, giving **7f** as a white solid (73.5 mg, 90% yield). ^1^H NMR (400 MHz, CDCl_3_): δ 2.17–2.25 (m, 1H), 3.46 (d, *J* = 7.8 Hz, 2H), 3.83 (d, *J* = 6.3 Hz, 2H), 5.07–5.14 (m, 2H), 5.36 (dd, *J*_1_ = 5.4 Hz, *J*_2_ = 9.0 Hz, 2H), 6.06–6.23 (m, 3H), 6.60–6.67 (m, 2H), 7.27–7.37 (m, 2H), 7.46–7.54 (m, 2H), 8.01–8.08 (m, 2H) ppm. ^13^C{^1^H} NMR (101 MHz, CDCl_3_): δ 36.2, 37.3, 39.1, 116.1, 124.2, 124.8 (2×C), 124.9, 125.4, 125.5, 125.8, 126.3, 126.5 (2×C), 131.0 (2×C), 132.3, 132.4, 134.6, 134.8, 137.1 ppm. HRMS (ESI^+^, *m*/*z*): fragmentation observed. Calcd for C_14_H_13_ [M]^+^ 181.1017; found, 181.1016 calcd for C_7_H_7_ [M]^+^ 91.0548; found, 91.0541.

#### 1-Allyl-4-(cyclohepta-2,4,6-trien-1-yl(phenyl)methyl)naphthalene (**7i**)

The crude compound was purified by column chromatography (SiO_2_, pentane), giving **7i** as a white solid (54.4, 52% yield). ^1^H NMR (400 MHz, CDCl_3_): δ 2.50–2.58 (m, 1H), 3.82 (d, *J* = 6.4 Hz, 2H), 5.03–5.17 (m, 4H), 5.39–5.46 (m, 1H), 6.06–6.21 (m, 3H), 6.69–6.77 (m, 2H), 7.14 (t, *J* = 7.4 Hz, 1H), 7.21–7.27 (m, 2H), 7.32–7.36 (m, 4H), 7.45–7.54 (m, 2H), 8.03–8.08 (m, 1H), 8.31–8.36 (m, 1H) ppm. ^13^C{^1^H} NMR (101 MHz, CDCl_3_): δ 37.4, 44.0, 48.4, 116.3, 124.2, 124.3, 124.7, 124.9, 125.2, 125.3, 125.7, 125.8, 126.0, 126.3, 128.4 (2×C), 128.6 (2×C), 130.9, 131.0, 132.6, 132.7, 134.9, 137.0, 137.8, 143.6 ppm. HRMS (ESI^+^, *m*/*z*): fragmentation observed. Calcd for C_7_H_7_ [M]^+^ 91.0548; found, 91.0542. Calcd for C_20_H_17_ [M]^+^ 257.1330; found, 257.1327.

#### 1-Allyl-4-(1-(cyclohepta-2,4,6-trien-1-yl)but-3-en-1-yl)naphthalene (**7j**)

The crude compound was purified by column chromatography (SiO_2_, pentane), giving **7j** as a white solid (56.2 mg, 60% yield). ^1^H NMR (400 MHz, CDCl_3_): δ 2.03–2.13 (m, 1H), 2.57–2.68 (m, 1H), 2.78–2.87 (m, 1H), 3.85 (d, *J* = 6.6 Hz, 2H), 3.94–4.05 (m, 1H), 4.78–4.84 (m, 1H), 4.89–4.98 (m, 2H), 5.08–5.16 (m, 2H), 5.47–5.58 (m, 2H), 5.98 (dd, *J*_1_ = 5.6 Hz, *J*_2_ = 9.4 Hz, 1H), 6.09–6.20 (m, 1H), 6.32 (dd, *J*_1_ = 5.3 Hz, *J*_2_ = 9.4 Hz, 1H), 6.64–6.76 (m, 2H), 7.19 (d, *J* = 7.5 Hz, 1H), 7.33 (d, *J* = 7.5 Hz, 1H), 7.50–7.57 (m, 2H), 8.07–8.12 (m, 1H), 8.18–8.25 (m, 1H) ppm. ^13^C{^1^H} NMR (101 MHz, CDCl_3_): δ 37.4, 39.0, 40.6 (broad), 43.9, 116.2, 116.3, 123.9, 124.2, 124.9, 125.0 (2×C), 125.2, 125.4, 125.5, 126.0, 130.6, 130.9, 132.3, 133.2, 134.3, 135.8, 137.1, 138.2 ppm. (Missing peak carbon due to overlapping signals). HRMS (ESI^+^, *m*/*z*): calcd for C_24_H_25_ [M + H]^+^ 313.1950; found, 313.1938.

#### (*E*)-2-Allyl-1-(3-(cyclohepta-2,4,6-trien-1-yl)prop-1-en-1-yl)naphthalene (**7m**)

The crude compound was purified by column chromatography (SiO_2_, pentane), giving **7m** as a white solid (67.1 mg, 75% yield). ^1^H NMR (400 MHz, CDCl_3_): δ 1.88–1.96 (m, 1H), 2.76–2.82 (m, 2H), 3.61 (d, *J* = 6.2 Hz, 2H), 4.96–5.03 (m, 1H), 5.04–5.09 (m, 1H), 5.37 (dd, *J*_1_ = 5.5 Hz, *J*_2_ = 9.0 Hz, 2H), 5.87–6.07 (m, 2H), 6.23–6.30 (m, 2H), 6.67–6.74 (m, 2H), 6.82 (d, *J* = 16.1 Hz, 1H), 7.35 (d, *J* = 8.5 Hz, 1H), 7.41–7.50 (m, 2H), 7.72 (d, *J* = 8.5 Hz, 1H), 7.78–7.84 (m, 1H), 8.10–8.15 (m, 1H) ppm. ^13^C{^1^H} NMR (101 MHz, CDCl_3_): δ 36.9, 38.3, 38.6, 115.6, 124.9 (2×C), 125.0, 125.7 (2×C), 126.1 (2×C), 126.9, 127.8, 128.0 (2×C), 131.0 (2×C), 132.4, 134.5 (2×C), 135.0 (2×C), 137.5 ppm. HRMS (ESI^+^, *m*/*z*): calcd for C_23_H_23_ [M + H]^+^ 299.1755; found, 299.1793.

### General Procedure for the Trapping with Tropylium Tetrafluoroborate

#### Method D **7n**–**7oa/ob**

To an oven-dried Schlenk, Pd(PPh_3_)_4_ (10 mol %) was dissolved in CH_2_Cl_2_ (3 mL); then the substrate was added (0.30 mmol, 1.0 equiv), and the mixture was stirred for 5 min under a nitrogen atmosphere. AllylSnBu_3_ (0.30 mmol, 1.0 equiv) was added at once, and the mixture was stirred at r.t. until the substrate was fully consumed. Then, the solvent was evaporated under reduced pressure in a rotatory evaporator, and the crude was passed through a small amount of basic alumina (Ø = 1 cm, h∼8–10 cm) using pentane as an eluent (∼100 mL). After evaporation of the solvent, the crude was dissolved in CH_2_Cl_2_ (0.5 mL) and added dropwise to a stirred solution of tropylium tetrafluoroborate (0.45 mmol, 1.5 equiv) in DMF (3 mL) at r.t. The mixture was stirred for 30 min at r.t.; then water (10 mL) and Et_2_O (10 mL) were added. Then, the resulting mixture was stirred for 5 min, and the layers were separated. The aqueous layer was extracted with Et_2_O (10 mL × 2), and the combined organic layers were dried over Na_2_SO_4_, filtered, and concentrated under reduced pressure. The crude product was purified by column chromatography (SiO_2_) using a mixture of pentane and CH_2_Cl_2_ as an eluent.

#### 7-(4-Allylbenzyl)cyclohepta-1,3,5-triene (**7n**)

The crude compound was purified by column chromatography (SiO_2_, pentane), giving **7n** as a white solid (42.7 mg, 64% yield). ^1^H NMR (400 MHz, CDCl_3_): δ 1.95–2.04 (m, 1H), 3.01 (d, *J* = 8.0 Hz, 2H), 3.37 (d, *J* = 6.8 Hz, 2H), 5.04–5.12 (m, 2H), 5.27 (dd, *J*_1_ = 5.5 Hz, *J*_2_ = 9.2 Hz, 2H), 5.92–6.04 (m, 1H), 6.13–6.22 (m, 2H) 6.62–6.69 (m, 2H), 7.10–7.17 (m, 4H) ppm. ^13^C{^1^H} NMR (101 MHz, CDCl_3_): δ 38.6, 39.9, 40.1, 115.7, 124.9 (2×C), 126.2 (2×C), 128.5 (2×C), 129.0 (2×C), 130.9 (2×C), 137.6, 137.8 (2×C) ppm. HRMS (ESI^+^, *m*/*z*): fragmentation observed. Calcd for C_7_H_7_ [M]^+^ 91.0548; found, 91.0541.

#### 7-(3-Allyl-4-Methyl-benzyl)cyclohepta-1,3,5-triene (**7oa**) + 7-(2-Allyl-4-methyl-benzyl)cyclohepta-1,3,5-triene (**7ob**)

The crude compound was purified by column chromatography (SiO_2_, pentane), giving a mixture of **7oa** and **7ob** as a white solid (43.9 mg, 62% yield—6:4 regioisomer ratio). ^1^H NMR (400 MHz, CDCl_3_): δ 1.95–2.08 (m, 1H_major_ + 1H_minor_), 2.27 (s, 3H_major_), 2.32(s, 3H_minor_), 2.97–3.04 (m, 2H_major_ + 2H_minor_), 3.34–3.40 (m, 2H_major_ + 2H_minor_), 4.97–5.10 (m, 2H_major_ + 2H_minor_), 5.24–5.31 (m, 2H_major_ + 2H_minor_), 5.90–6.02 (m, 1H_major_ + 1H_minor_), 6.15–6.22 (m, 2H_major_ + 2H_minor_), 6.63–6.70 (m, 2H_major_ + 2H_minor_), 9.97–7.12 (m, 3H_major_ + 3H_minor_) ppm. ^13^C{^1^H} NMR (101 MHz, CDCl_3_): δ 18.9, 21.0, 35.3, 37.1, 37.7, 38.7, 39.1, 40.1, 115.6, 115.7, 124.8, 124.9, 126.3 (2×C), 126.7, 127.0, 128.3, 128.8, 129.0, 129.1, 129.4, 129.9 (2×C), 130.1 (2×C), 130.4, 130.9 (2×C), 134.0, 134.9, 135.8, 136.7, 137.2, 137.7, 137.9, 138.0 ppm. HRMS (ESI^+^, *m*/*z*): calcd for C_18_H_21_ [M + H]^+^ 237.1638; found, 237.1646.

### General Procedure for the Trapping with 1,3-Benzo Dithiolylium Tetrafluoroborate

#### Method E **8a–m**

To an oven-dried Schlenk, under a nitrogen atmosphere, were added the substrate (0.30 mmol, 1.0 equiv) and Pd(PPh_3_)_4_ (1 mol %); then dry 2-Me-THF (1 mL) was added, and the mixture was stirred for 5 min. Allyl magnesium bromide (375 μL, 1.0 M in Et_2_O, 1.25 equiv) was added at once, and the mixture was stirred at r.t. until completion (TLC check, finished in 15 min). After complete consumption of the substrate, pentane (20 mL) was added to precipitate out the salts and the suspension was filtered through a plug of Celite. Evaporation of the solvent yielded the crude dearomatized product, which was dissolved in 0.5 mL of CH_2_Cl_2_ and added dropwise to a stirred solution of 1,3-benzo dithiolylium tetrafluoroborate (0.39 mmol, 1.3 equiv) in acetone (6 mL) at r.t. The mixture was stirred for 30 min at r.t.; then the solvent was evaporated under reduced pressure, and the crude product was purified by column chromatography (SiO_2_) using a mixture of pentane and CH_2_Cl_2_ as an eluent.

#### 2-((3-Allyl-4-methylnaphthalen-1-yl)methyl)-benzo[*d*][1,3]dithiole (**8aa**) + 2-((2-Allyl-4-methylnaphthalen-1-yl)methyl)benzo[*d*][1,3]dithiole (**8ab**)

The crude compound was purified by column chromatography (SiO_2_, pentane/CH_2_Cl_2_ from 90:10 to 85:15), giving a mixture of **8aa** and **8ab** as a colorless sticky oil (92.0 mg, 88% yield—85:15 regioisomer ratio). ^1^H NMR (400 MHz, CDCl_3_): δ 2.63 (s, 3H major), 2.69 (s, 3H minor), 3.60–3.69 (m, 4H major + 2H minor), 3.75 (d, *J* = 7.6 Hz, 2H minor), 4.87–4.98 (m, 1H minor), 4.99–5.14 (m, 2H major + 1H minor), 5.21–5.30 (m, 1H major + 1H minor), 5.92–6.11 (m, 1H major + 1H minor), 7.04–7.13 (m, 2H major + 2H minor), 7.21–7.33 (m, 3H major + 3H minor), 7.48–7.58 (m, 2H major + 2H minor), 7.93–8.01 (m, 1H major + 1H minor), 8.02–8.05 (m, 1H minor), 8.09–8.15 (m, 1H major) ppm. ^13^C{^1^H} NMR (101 MHz, CDCl_3_): δ 17.1 (major), 22.2 (minor), 39.3 (minor), 40.9 (minor), 41.2 (major), 44.6 (major), 57.7 (major), 58.1 (minor), 118.3 (major), 118.6 (minor), 125.3 (major), 125.5 (minor), 126.3 (major), 126.9 (minor), 127.6 (minor), 127.7 (major + minor), 127.8 (major), 128.2 (major), 128.3 (major), 128.4 (minor), 128.6 (minor), 131.3 (minor), 132.0 (minor), 133.3 (major), 133.5 (major), 133.7 (major), 134.0 (major), 134.7 (minor), 135.0 (minor), 136.2 (major), 136.6 (minor), 136.9 (major), 139.2 (minor), 139.4 (major), 139.8 (minor), 140.0 (major), 140.2 (minor) ppm. HRMS (ESI^+^, *m*/*z*): calcd for C_22_H_20_S_2_ [M + H]^+^ 349.1079; found, 349.1070.

#### 2-((3-Allyl-4-ethylnaphthalen-1-yl)methyl)benzo[*d*][1,3]dithiole (**8ba**) + 2-((2-Allyl-4-ethylnaphthalen-1-yl)methyl)-benzo[*d*][1,3]dithiole (**8bb**)

The crude compound was purified by column chromatography (SiO_2_, pentane/CH_2_Cl_2_ 90:10), giving a mixture of **8ba** and **8bb** as a colorless sticky oil (83.7 mg, 72% yield—82:18 regioisomer ratio). ^1^H NMR (400 MHz, CDCl_3_): δ 1.32 (t, *J* = 7.5 Hz, 2H major), 1.14 (t, *J* = 7.5 Hz, 2H minor), 3.08–3.17 (m, 2H major + 2H minor), 3.60–3.70 (m, 4H major + 2H minor), 3.75 (d, *J* = 7.6 Hz, 2H minor), 4.87–4.94 (m, 1H minor), 5.03–5.15 (m, 2H major + 1H minor), 5.21–5.30 (m, 1H major + 1H minor), 5.93–6.03 (m, 1H minor), 6.04–6.14 (m, 1H major), 7.05–7.14 (m, 2H major + 2H minor), 7.23–7.33 (m, 3H major + 3H minor),7.49–7.58 (m, 2H major + 2H minor), 7.94–8.02 (m, 1H major + 1H minor), 8.08–8.12 (m, 1H minor), 8.13–8.17 (m, 1H major) ppm. ^13^C{^1^H} NMR (101 MHz, CDCl_3_): δ 17.7 (minor), 17.9 (major), 24.1 (major), 28.6 (minor), 39.4 (minor), 40.4 (major), 41.1 (minor), 44.7 (major), 57.6 (major), 58.1 (minor), 118.4 (major), 118.6 (minor), 125.3 (major), 125.5, 126.5 (major), 127.0 (minor), 127.3 (minor), 127.5 (minor), 127.6 (major), 127.8 (major), 128.2 (major), 128.3 (major), 128.4, 128.5 (minor), 130.3 (minor), 131.3 (minor), 133.7 (major + minor), 133.8 (major + minor), 135.2 (major), 135.3 (minor), 136.3 (major + minor), 139.8 (minor), 140.0 (major), 140.1 (major), 140.2 (minor), 142.5 (minor) ppm. HRMS (ESI^+^, *m*/*z*): calcd for C_23_H_23_S_2_ [M + H]^+^ 363.1235; found, 363.1237.

#### 2-((4-Allyl-3-(4-methoxyphenyl)naphthalen-1-yl)methyl)benzo[*d*][1,3]dithiole (**8e**)

The crude compound was purified by column chromatography (SiO_2_, pentane/CH_2_Cl_2_ from 90:10 to 85:15), giving **8e** as a white solid (101.8 mg, 77% yield). ^1^H NMR (400 MHz, CDCl_3_): δ 3.68 (d, *J* = 7.4 Hz, 2H), 3.78–3.82 (m, 2H), 3.90 (s, 3H), 4.86–4.94 (m, 1H), 5.09–5.15 (m, 1H), 5.26 (t, *J* = 7.4 Hz, 1H), 6.09–6.20 (m, 1H), 6.99–7.10 (m, 4H), 7.24–7.30 (m, 2H), 7.36–7.43 (m, 3H), 7.52–7.60 (m, 2H), 7.99–8.04 (m, 1H), 8.11–8.17 (m, 1H) ppm. ^13^C{^1^H} NMR (101 MHz, CDCl_3_): δ 33.9, 42.1, 54.9, 55.3, 113.5 (2×C), 116.2, 122.7 (2×C), 123.7, 125.6 (2×C), 125.7, 126.0, 126.2, 130.5 (2×C), 131.0, 131.2, 131.5, 132.1, 133.0, 134.6, 137.2 (2×C), 137.8, 138.8, 158.8. ppm. HRMS (ESI^+^, *m*/*z*): calcd for C_28_H_23_OS_2_ [M – H]^+^ 439.1190; found, 439.1192.

#### 2-((4-Allylnaphthalen-1-yl)methyl)benzo[*d*][1,3]dithiole (**8f**)

The crude compound was purified by column chromatography (SiO_2_, pentane/CH_2_Cl_2_ 90:10), giving **8f** as a colorless sticky oil (72.2 mg, 72% yield). ^1^H NMR (400 MHz, CDCl_3_): δ 3.68 (d, *J* = 7.4 Hz, 2H), 3.86 (d, *J* = 6.3 Hz, 2H), 5.09–5.18 (m, 2H), 5.23 (t, *J* = 7.4 Hz, 1H), 6.08–6.20 (m, 1H), 7.05–7.11 (m, 2H), 7.25–7.31 (m, 2H), 7.32–7.38 (m, 2H), 7.52–7.59 (m, 2H), 7.96–8.03 (m, 1H), 8.08–8.15 (m, 1H) ppm. ^13^C{^1^H} NMR (101 MHz, CDCl_3_): δ 37.4, 42.2, 54.9, 116.4, 122.7 (2×C), 123.9, 125.1, 125.6 (3×C), 125.8, 125.9, 128.1, 131.9, 132.0, 132.4, 136.0, 136.9, 137.2 (2×C) ppm. HRMS (ESI^+^, *m*/*z*): calcd for C_21_H_17_S_2_ [M – H]^+^ 333.0850; found, 333.0771.

#### 2-((4-Allyl-2-methylnaphthalen-1-yl)methyl)-benzo[d][1,3]dithiole (**8g**)

The crude compound was purified by column chromatography (SiO_2_, pentane/CH_2_Cl_2_ 90:10), giving **8g** as a white solid (81.5 mg, 78% yield). ^1^H NMR (400 MHz, CDCl_3_): δ 3.50 (s, 3H), 3.74 (d, *J* = 7.7 Hz, 2H), 3.82 (d, *J* = 6.3 Hz, 2H), 5.10–5.17 (m, 2H), 5.36 (t, *J* = 7.7 Hz, 1H), 6.07–6.19 (m, 1H), 7.06–7.12 (m, 2H), 7.23 (s, 1H) 7.27–7.33 (m, 2H), 7.44–7.55 (m, 2H), 7.96 (d, *J* = 8.4 Hz, 1H), 8.05 (d, *J* = 8.4 Hz, 1H) ppm. ^13^C{^1^H} NMR (101 MHz, CDCl_3_): δ 23.7, 39.9, 40.0, 57.9, 118.9, 125.4 (2×C), 126.7, 127.3, 127.5, 128.4 (2×C), 128.6, 131.8, 132.5, 133.7, 135.2, 137.8, 138.0, 139.6, 140.2 ppm. (Missing peak carbon due to overlapping signals). HRMS (ESI^+^, *m*/*z*): calcd for C_22_H_19_S_2_ [M – H]^+^ 347.0928; found, 347.0926.

#### 2-((4-Allyl-6-methoxynaphthalen-1-yl)methyl)-benzo[*d*][1,3]dithiole (**8h**)

The crude compound was purified by column chromatography (SiO_2_, pentane/CH_2_Cl_2_ from 90:10 to 85:15), giving **8h** as a white solid (67.8 mg, 62% yield). ^1^H NMR (400 MHz, CDCl_3_): δ 3.63 (d, *J* = 7.4 Hz, 2H), 3.80 (d, *J* = 6.3 Hz, 2H), 3.94 (s, 3H), 5.12–5.22 (m, 3H), 6.06–6.18 (m, 1H), 7.04–7.11 (m, 2H), 7.19–7.33 (m, 5H), 7.36 (d, *J* = 2.5 Hz, 1H), 7.90 (d, *J* = 9.3 Hz, 1H) ppm. ^13^C{^1^H} NMR (101 MHz, CDCl_3_): δ 37.8, 42.1, 55.1, 55.3, 103.9, 116.3, 118.0, 122.7 (2×C), 125.5, 125.6 (2×C), 125.7, 126.4, 127.3, 132.0, 133.7, 134.6, 136.7, 137.2 (2×C), 157.3 ppm. HRMS (ESI^+^, *m*/*z*): calcd for C_22_H_19_OS_2_ [M – H]^+^ 363.0877; found, 363.0878.

#### 2-((4-Allylnaphthalen-1-yl) (phenyl)methyl)benzo[*d*][1,3]dithiole (**8i**)

The crude compound was purified by column chromatography (SiO_2_, pentane/CH_2_Cl_2_ from 90:10 to 85:15), giving **8i** as a white solid (55.4 mg, 45% yield). ^1^H NMR (400 MHz, CDCl_3_): δ 3.85 (d, *J* = 6.3 Hz, 2H), 5.11–5.18 (m, 2H), 5.38 (d, *J* = 11.1 Hz, 1H), 6.05 (d, *J* = 11.1 Hz, 1H), 6.08–6.19 (m, 1H), 6.95–7.08 (m, 3H), 7.15–7.24 (m, 2H), 7.27–7.32 (m, 2H), 7.40–7.55 (m, 6H) 8.03–8.09 (m, 1H), 8.14–8.19 (m, 1H) ppm. ^13^C{^1^H} NMR (101 MHz, CDCl_3_): δ 37.4, 53.2, 59.7, 116.5, 122.2 (2×C), 123.6, 124.2, 124.8, 125.4, 125.5, 125.6, 125.7, 126.0, 127.3, 128.4 (2×C), 128.6 (2×C), 132.2, 132.6, 135.9, 136.4, 136.7, 137.4, 137.9, 141.3 ppm. HRMS (ESI^+^, *m*/*z*): fragmentation observed. Calcd for C_7_H_5_S_2_ [M]^+^ 152.9833; found, 152.9827. Calcd for C_20_H_17_ [M]^+^ 257.1330; found, 257.1324.

#### 2-(1-(4-Allylnaphthalen-1-yl)ethyl)benzo[*d*][1,3]dithiole (**8k**)

The crude compound was purified by column chromatography (SiO_2_, pentane/CH_2_Cl_2_ 90:10), giving **8k** as a white solid (81.5 mg, 78% yield). ^1^H NMR (400 MHz, CDCl_3_): δ 1.56 (d, *J* = 7.0 Hz, 3H), 3.80–3.93 (m, 2H), 4.17 (m, 1H), 5.11–5.20 (m, 2H), 5.41 (d, *J* = 7.0 Hz, 1H), 6.10–6.22 (m, 1H), 6.99–7.06 (m, 2H), 7.10–7.15 (m, 1H), 7.22–7.27 (m, 1H), 7.37–7.44 (m, 2H), 7.51–7.58 (m, 2H), 8.05–5.16 (m, 2H) ppm. ^13^C{^1^H} NMR (101 MHz, CDCl_3_): δ 17.7, 37.4, 41.6, 60.4, 116.4, 121.9, 122.0, 123.5, 123.6, 125.1, 125.3, 125.4, 125.6, 125.9 (2×C), 131.8, 132.4, 135.5, 136.9, 137.8 (2×C), 138.1 ppm. HRMS (ESI^+^, *m*/*z*): calcd for C_22_H_19_S_2_ [M – H]^+^ 347.0928; found, 347.0919. Fragmentation observed (HRMS – ESI): calcd for C_7_H_5_S_2_ [M]^+^ 152.9833; found, 152.9827. Calcd for C_15_H_15_ [M]^+^ 195.1174; found, 195.1166.

#### (*E*)-2-(3-(2-Allylnaphthalen-1-yl)allyl)benzo[d][1,3]dithiole (**8m**)

The crude compound was purified by column chromatography (SiO_2_, pentane/CH_2_Cl_2_ 95:5), giving **8m** as a colorless sticky oil (72.5 mg, 67% yield). ^1^H NMR (400 MHz, CDCl_3_): δ 2.99 (d, *J*_1_ = 1.4 Hz *J*_2_ = 7.0 Hz, 2H), 3.61 (d, *J* = 6.2 Hz, 2H), 4.99–5.13 (m, 3H), 5.80–5.90 (m, 1H), 5.98–6.09 (m, 1H), 6.86 (d, *J* = 16.0 Hz, 1H), 7.04–7.09 (m, 2H), 7.26–7.31 (m, 2H), 7.37 (d, *J* = 8.4 Hz, 1H), 7.44–7.52 (m, 2H), 7.74 (d, *J* = 8.4 Hz, 1H), 7.81–7.85 (m, 1H), 8.08–8.14 (m, 1H) ppm. ^13^C{^1^H} NMR (101 MHz, CDCl_3_): δ 38.3, 43.0, 53.9, 115.8, 122.6 (2×C), 125.1, 125.6 (2×C), 125.7, 126.0, 127.3, 128.0, 128.1, 130.5, 131.9, 132.2, 132.4, 133.9, 134.6, 137.2 (2×C), 137.4 ppm. HRMS (ESI^+^, *m*/*z*): calcd for C_23_H_21_S_2_ [M + H]^+^ 361.1075; found, 361.1089.

### General Procedure for the Trapping with 1,3-Benzo Dithiolylium Tetrafluoroborate

#### Method F **8n**

To an oven-dried Schlenk, under a nitrogen atmosphere, Pd(PPh_3_)_4_ (10 mol %) was dissolved in CH_2_Cl_2_ (3 mL); then the substrate was added (0.30 mmol, 1.0 equiv), and the mixture was stirred for 5 min. AllylSnBu_3_ (0.30 mmol, 1.0 equiv) was added at once, and the mixture stirred at r.t. until the substrate was fully consumed. Then, the solvent was evaporated, and the crude was passed through a small amount of basic alumina (Ø = 1 cm, *h* ∼ 8–10 cm) using pentane as an eluent (∼100 mL). After evaporation of the solvent, the crude was dissolved in 0.5 mL of CH_2_Cl_2_ and added dropwise to a stirred solution of 1,3-benzo dithiolylium tetrafluoroborate (0.39 mmol, 1.3 equiv) in acetone (6 mL) at r.t. The mixture was stirred for 30 min at r.t.; then the solvent was evaporated under reduced pressure, and the crude product was purified by column chromatography (SiO_2_) using a mixture of pentane and CH_2_Cl_2_ as an eluent.

#### 2-(4-Allylbenzyl)benzo[*d*][1,3]dithiole (**8n**)

The crude compound was purified by column chromatography (SiO_2_, pentane/CH_2_Cl_2_ 90:10), giving **8n** as a colorless sticky oil (55.5 mg, 65% yield). ^1^H NMR (400 MHz, CDCl_3_): δ 3.18 (d, *J* = 7.5 Hz, 2H), 3.38 (d, *J* = 6.7 Hz, 2H), 4.99 (t, *J* = 7.5 Hz, 1H), 5.05–5.11 (m, 2H), 5.91–6.06 (m, 1H), 7.01–7.06 (m, 2H), 7.12–7.18 (m, 4H), 7.20–7.25 (m, 2H) ppm. ^13^C{^1^H} NMR (101 MHz, CDCl_3_): δ 39.9, 44.6, 55.7, 115.9, 122.6 (2×C), 125.5 (2×C), 128.6 (2×C), 129.5 (2×C), 135.2, 137.0, 137.3, 138.9 ppm. (Missing peak due to overlapping signals; it is not possible to distinguish which signal belongs to the two symmetric quaternary aromatic carbons bonded to sulfur). HRMS (ESI^+^, *m*/*z*): calcd for C_17_H_15_S_2_ [M – H]^+^ 283.0615; found, 283.0605. Fragmentation observed. HRMS (ESI^+^, *m*/*z*): calcd for C_7_H_5_S_2_ [M]^+^ 152.9833; found, 152.9825. Calcd for C_10_H_11_ [M]^+^ 131.0861; found, 131.0852.

### General Procedure for the Alkylation of benzo[*d*][1,3]dithiole Derivatives **9fa–kb**

Following a literature procedure,^25^ a solution of *n*BuLi (2.5 M in hexanes, 0.50 mmol, 1.05 equiv) was added dropwise to a solution of 2-((4-allylnaphthalen-1-yl)methyl)benzo[d] [1,3]dithiole (**8f**) (0.50 mmol, 1.0 equiv) in anhydrous in THF (5 mL) at 0 °C. The mixture turns to a deep-blue color. After 5 min, MeI (1.00 mmol, 2.0 equiv) was added and the solution slowly turned to pale yellow. The solution was stirred for 5 min, and then water (5 mL) was added. The organic layer was separated, and the aqueous layer was extracted with Et_2_O (10 mL × 2). The collected organic layers were washed with brine (10 mL), dried over Na_2_SO_4_, and concentrated under reduced pressure. The crude product was purified by column chromatography (SiO_2_) using a mixture of pentane and CH_2_Cl_2_ as an eluent.

#### 2-((4-Allylnaphthalen-1-yl)methyl)-2-methylbenzo[*d*][1,3]dithiole (**9fa**)

The compound was synthesized using the general procedure for alkylation (using MeI). The crude compound was purified by column chromatography (SiO_2_, pentane/CH_2_Cl_2_ 95:5), giving **9fa** as a colorless sticky oil (127.2 mg, 73% yield). ^1^H NMR (400 MHz, CDCl_3_): δ 1.87 (s, 3H), 3.86 (d, *J* = 6.3 Hz, 2H), 3.92 (s, 2H), 5.08–5.19 (m, 2H), 6.05–6.25 (m, 1H), 7.02–7.10 (m, 2H), 7.19–7.25 (m, 2H), 7.31–7.41 (m, 1H), 7.47–7.56 (m, 3H), 8.05–8.23 (m, 2H) ppm. ^13^C{^1^H} NMR (101 MHz, CDCl_3_): δ 29.0, 37.4, 43.8, 70.6, 116.3, 122.8 (2×C), 124.7, 125.3, 125.4 (3×C), 125.5, 125.5, 129.3, 131.7, 132.3, 133.1, 136.0, 136.9, 138.4 (2×C) ppm. HRMS (ESI^+^, *m*/*z*): fragmentation observed. Calcd for C_8_H_7_S_2_ [M]^+^ 166.9989; found, 166.9984. Calcd for C_14_H_13_ [M]^+^ 181.1017; found, 181.1011.

#### 2-((4-Allylnaphthalen-1-yl)methyl)-2-hexylbenzo[*d*][1,3]dithiole (**9fb**)

The compound was synthesized using the general procedure for alkylation (using HexylI). The crude compound was purified by column chromatography (SiO_2_, pentane/CH_2_Cl_2_ 95:5), giving **9fb** as a colorless sticky oil (175.8 mg, 84% yield). ^1^H NMR (400 MHz, CDCl_3_): δ 0.87 (d, *J* = 6.8 Hz, 3H), 1.23–1.35 (m, 6H), 1.63–1.73 (m, 2H), 2.07–2.14 (m, 2H), 3.81–3.86 (m, 4H), 5.07–5.16 (m, 2H), 6.06–6.18 (m, 1H), 6.91–7.05 (m, 2H), 7.10–7.15 (m, 2H), 7.30 (d, *J* = 7.3 Hz, 1H), 7.43 (d, *J* = 7.3 Hz, 1H), 7.45–7.53 (m, 2H), 8.02–8.07 (m, 1H), 8.09–8.14 (m, 1H) ppm. ^13^C{^1^H} NMR (101 MHz, CDCl_3_): δ 14.1, 22.6, 26.8, 29.3, 31.7, 37.4, 40.3, 42.6, 75.4, 116.3, 122.5 (2×C), 124.5, 125.0, 125.3 (3×C), 125.4, 125.5, 129.3, 131.5, 132.2, 133.5, 135.8, 136.9, 138.4 (2×C) ppm. HRMS (ESI^+^, *m*/*z*): fragmentation observed. Calcd for C_13_H_17_S_2_ [M]^+^ 237.0772; found, 237.0776. Calcd for C_14_H_13_ [M]^+^ 181.1017; found, 181.1013.

#### 2-((4-Allylnaphthalen-1-yl)methyl)-2-benzylbenzo[*d*][1,3]dithiole (**9fc**)

The compound was synthesized using the general procedure for alkylation (using BnBr). The crude compound was purified by column chromatography (SiO_2_, pentane/CH_2_Cl_2_ 95:5), giving **9fc** as a white solid (154.9 mg, 73% yield). ^1^H NMR (400 MHz, CDCl_3_): δ 3.51 (s, 2H), 3.80–3.89 (m, 4H), 5.07–5.18 (m, 2H), 6.07–6.19 (m, 1H), 6.83–6.89 (m, 2H), 6.96–7.01 (m, 2H), 7.26–7.35 (m, 4H), 7.37–7.42 (m, 2H), 7.44–7.53 (m, 3H), 7.96 (d, *J* = 8.1 Hz, 1H), 8.05 (d, *J* = 8.1 Hz, 1H) ppm. ^13^C{^1^H} NMR (101 MHz, CDCl_3_): δ 37.4, 41.5, 48.3, 75.4, 116.3, 122.3 (2×C), 124.6, 125.1 (2×C), 125.2 (2×C), 125.3, 125.5, 127.1, 127.7 (2×C), 129.7, 131.4 (2×C), 131.5, 132.2, 133.2, 135.8, 136.2, 136.9, 138.2 (2×C) ppm. HRMS (ESI^+^, *m*/*z*): fragmentation observed. Calcd for C_14_H_11_S_2_ [M]^+^ 243.0302; found, 243.0308. Calcd for C_14_H_13_ [M]^+^ 181.1017; found, 181.1011.

#### 2-(1-(4-Allylnaphthalen-1-yl)ethyl)-2-methylbenzo[*d*][1,3]dithiole (**9ka**)

The compound was synthesized using the general procedure for alkylation (using MeI). The crude compound was purified by column chromatography (SiO_2_, pentane/CH_2_Cl_2_ 95:5), giving **9ka** as a colorless sticky oil (134.1 mg, 74% yield). ^1^H NMR (400 MHz, CDCl_3_): δ 1.70 (d, *J* = 6.9 Hz, 3H), 1.84 (s, 3H), 3.86 (d, *J* = 6.4 Hz, 2H), 4.53 (q, *J* = 6.9 Hz, 1H), 5.09–5.17 (m, 2H), 6.07–6.20 (m, 1H), 6.97–7.04 (m, 2H), 7.08–7.13 (m, 1H), 7.17–7.21 (m, 1H), 7.37 (d, *J* = 7.4 Hz, 1H), 7.48–7.55 (m, 2H), 7.62 (d, *J* = 7.4 Hz, 1H), 8.05–8.12 (m, 1H), 8.22–8.28 (m, 1H) ppm. ^13^C{^1^H} NMR (101 MHz, CDCl_3_): δ 19.6, 28.6, 37.5, 42.2, 74.9, 116.3, 122.3, 122.4, 124.5, 124.8, 124.9, 125.2, 125.3 (2×C), 125.4, 125.6, 132.2, 132.6, 135.5, 136.9, 137.2, 138.3, 138.4 ppm. HRMS (ESI^+^, *m*/*z*): fragmentation observed. Calcd for C_8_H_7_S_2_ [M]^+^ 166.9989; found, 166.9984. Calcd for C_15_H_15_ [M]^+^ 195.1174; found, 195.1171.

#### 2-(1-(4-Allylnaphthalen-1-yl)ethyl)-2-hexylbenzo[*d*][1,3]dithiole (**9kb**)

The compound was synthesized using the general procedure for alkylation (using HexyI). The crude compound was purified by column chromatography (SiO_2_, pentane/CH_2_Cl_2_ 95:5), giving **9kb** as a colorless sticky oil (168.7 mg, 78% yield). ^1^H NMR (400 MHz, CDCl_3_): δ 0.80 (d, *J* = 6.9 Hz, 3H), 1.08–1.22 (m, 6H), 1.54–1.65 (m, 2H), 1.71 (d, *J* = 6.8 Hz, 3H), 1.93–2.10 (m, 2H), 3.84 (d, *J* = 6.3 Hz, 2H), 4.37 (q, *J* = 6.8 Hz, 1H), 5.08–5.18 (m, 2H), 6.08–6.20 (m, 1H), 6.91–7.01 (m, 2H), 7.04–7.08 (m, 1H), 7.10–7.14 (m, 1H), 7.34 (d, *J* = 7.5 Hz, 1H), 7.50–7.56 (m, 2H), 7.74 (d, *J* = 7.5 Hz, 1H), 8.05–8.12 (m, 1H), 8.17–8.24 (m, 1H) ppm. ^13^C{^1^H} NMR (101 MHz, CDCl_3_): δ 14.0, 19.7, 22.5, 26.2, 29.2, 31.5, 37.5, 41.1, 42.6, 79.6, 116.3, 121.4, 121.6, 124.2, 124.8, 124.9, 125.0, 125.2, 125.3 (2×C), 125.7, 132.2, 132.7, 135.1, 136.9, 137.6, 138.5, 138.9 ppm. HRMS (ESI^+^, *m*/*z*): fragmentation observed. Calcd for C_13_H_17_S_2_ [M]^+^ 237.0772; found, 237.0769. Calcd for C_15_H_15_ [M]^+^ 195.1174; found, 195.1170.

### General Procedure for the Reductive Removal of Benzothiol Group **10fa**–**kb**

Following a literature procedure,^25^ to a solution of **9fa** (0.10 mmol, 1.0 equiv) in ethanol (2 mL), Ni-Raney (0.50 g, slurry in water) was added, and the reaction was maintained under a H_2_ atmosphere (1.0 atm). After 3 h, the reaction mixture was filtered through a plug of Celite and the organic solvent was removed under reduced pressure. The residue was diluted with AcOEt (10 mL), the organic layer was separated, and the aqueous layer was extracted with AcOEt (10 mL × 2). The collected organic layers were washed with brine (10 mL), dried over Na_2_SO_4_, and filtered, and the solvent was removed under reduced pressure in a rotatory evaporator. The crude product was purified by column chromatography (SiO_2_) using pentane as an eluent.

#### 1,4-Dipropylnaphthalene (**10fa**)^26^

The crude compound was purified by column chromatography (SiO_2_, pentane), giving **10fa** as a colorless oil (16.2 mg, 76% yield). ^1^H NMR (400 MHz, CDCl_3_): δ 1.03 (t, *J* = 7.3 Hz, 6H), 1.78 (m, 4H), 3.02 (t, *J* = 7.3 Hz, 4H), 7.24 (s, 2H), 7.46–7.53 (m, 2H), 8.04–8.10 (m, 2H) ppm. ^13^C{^1^H} NMR (101 MHz, CDCl_3_): δ 14.3 (2×C), 23.9 (2×C), 35.2 (2×C), 124.6 (2×C), 125.0 (2×C), 125.6 (2×C), 132.2 (2×C), 136.8 (2×C) ppm.

#### 1-Propyl-4-octyl-naphthalene (**10fb**)

The crude compound was purified by column chromatography (SiO_2_, pentane), giving **10fb** as a colorless oil (24.8 mg, 88% yield). ^1^H NMR (400 MHz, CDCl_3_): δ 0.88 (t, *J* = 6.7 Hz, 3H), 1.03 (t, *J* = 7.3 Hz, 3H), 1.24–1.38 (m, 8H), 1.39–1.49 (m, 2H), 1.39–1.83 (m, 4H), 2.99–3.06 (m, 4H), 7.24 (s, 2H), 7.47–7.53 (m, 2H), 8.04–8.10 (m, 2H) ppm. ^13^C{^1^H} NMR (101 MHz, CDCl_3_): δ 14.1, 14.3, 22.7, 23.9, 29.3, 29.5, 29.9, 30.9, 31.9, 33.2, 35.2, 124.6 (2×C), 125.0 (2×C), 125.5, 125.6, 132.2, 132.2, 136.7, 137.1 ppm. HRMS (MALDI–TOF): calcd for C_21_H_31_ [M]^+^ 283.2426; found, 283.2428.

#### 1-(3-Phenylpropyl)-4-propyl-naphthalene (**10fc**)

The crude compound was purified by column chromatography (SiO_2_, pentane/CH_2_Cl_2_ 99:1), giving **10fc** as a colorless oil (24.2 mg, 84% yield). ^1^H NMR (400 MHz, CDCl_3_): δ 1.05 (t, *J* = 7.3 Hz, 3H), 1.79 (m, 2H), 2.11 (m, 2H), 2.78 (t, *J* = 7.3 Hz, 2H), 3.04 (t, *J* = 7.8 Hz, 2H), 3.10 (t, *J* = 7.8 Hz, 2H), 7.18–7.28 (m, 5H), 7.29–7.34 (m, 2H), 7.49–7.54 (m, 2H), 7.98–8.04 (m, 1H), 5.05–8.12 (m, 1H) ppm. ^13^C{^1^H} NMR (101 MHz, CDCl_3_): δ 14.3, 23.9, 32.3, 32.6, 35.2, 35.9, 124.5, 124.6, 125.1, 125.2, 125.6, 125.6, 125.8, 128.3 (2×C), 128.5 (2×C), 132.2, 132.3, 136.5, 137.0, 142.3 ppm. HRMS (MALDI–TOF): calcd for C_22_H_25_ [M]^+^ 289.1956; found, 289.1950.

#### 1-(1-Methyl-propyl)-4-propylnaphthalene (**10ka**)

The crude compound was purified by column chromatography (SiO_2_, pentane), giving **10ka** as a colorless oil (19.2 mg, 85% yield). ^1^H NMR (400 MHz, CDCl_3_): δ 0.94 (t, *J* = 7.4 Hz, 3H), 1.04 (t, *J* = 7.4 Hz, 3H), 1.37 (d, *J* = 6.9 Hz, 3H), 1.64–1.92 (m, 4H), 3.03 (t, *J* = 7.4 Hz, 2H), 3.50 (m, 1H), 7.30 (s, 2H), 7.47–7.53 (m, 2H), 8.06–8.11 (m, 1H), 8.13–8.19 (m, 1H) ppm. ^13^C{^1^H} NMR (101 MHz, CDCl_3_): δ 12.3, 14.4, 21.2, 23.9, 30.5, 35.1, 35.3, 122.1, 123.9, 124.7, 124.9, 125.0, 125.7, 132.1, 132.3, 136.3, 141.7 ppm. HRMS (MALDI–TOF): calcd for C_17_H_23_ [M]^+^ 227.1800; found, 227.1799.

#### 1-(1-Methyl-octyl)-4-propylnaphthalene (**10kb**)

The crude compound was purified by column chromatography (SiO_2_, pentane), giving **10kb** as a colorless oil (24.0 mg, 89% yield). ^1^H NMR (400 MHz, CDCl_3_): δ 0.88 (t, *J* = 6.7 Hz, 3H), 1.05 (t, *J* = 7.4 Hz, 3H), 1.19–1.42 (m, 13H), 1.59–1.71 (m, 1H), 1.74–1.87 (m, 3H), 3.04 (t, *J* = 7.7 Hz, 3H), 3.57 (m, 1H), 7.32 (s, 2H), 7.48–7.54 (m, 2H), 8.07–8.12 (m, 1H), 8.15–8.20 (m, 1H) ppm. ^13^C{^1^H} NMR (101 MHz, CDCl_3_): δ 14.1, 14.4, 21.7, 22.7, 23.9, 27.9, 29.3, 29.9, 31.9, 33.5, 35.3, 37.9, 122.1, 123.8, 124.7, 124.9, 125.0, 125.8, 132.0, 132.3, 136.3, 142.1 ppm. HRMS (MALDI–TOF): calcd for C_22_H_33_ [M]^+^ 297.2582; found, 297.2580.

#### Synthesis of 1-(4-Propylnaphthalen-1-yl)propan-2-one **(11)**

After two vacuum/H_2_ cycles, to replace air inside the reaction tube with hydrogen, the mixture of substrate **9fa** (0.20 mmol, 1.0 equiv) and 10% Pd/C (10 wt % of the substrate) in MeOH (2 mL) was vigorously stirred at room temperature under a H_2_ atmosphere for 24 h. The reaction mixture was filtered through a plug of Celite, and the filtrate was concentrated to provide the product, which was used in the next step without further purification. Following a literature procedure,^25^ to a suspension of HgO (0.40 mmol, 2.0 equiv) in THF, 48% solution of HBF_4_ in water was added (200 μL). After 5 min, a solution of dithiane (in 1 mL THF) was slowly added and the precipitated was dissolved. After 30 min, a saturated solution of NaHCO_3_ was slowly added at 0 °C until basic pH. The solid was filtered through a plug of Celite, the organic solvent was evaporated, and the residue was diluted with AcOEt (10 mL). The organic layer was separated, and the aqueous layer was extracted with AcOEt (10 mL × 2). The collected organic layers were washed with brine (10 mL), dried over Na_2_SO_4_, and concentrated under reduced pressure. The crude compound was purified by column chromatography (SiO_2_, pentane/CH_2_Cl_2_ 50:50), giving **11** as a colorless oil (37.6 mg, 83% yield). ^1^H NMR (400 MHz, CDCl_3_): δ 1.04 (t, *J* = 7.7 Hz, 3H), (m, 2H), 2.11 (s, 3H), 3.05 (t, *J* = 7.7 Hz, 2H), 4.09 (s, 2H), 7.28–7.34 (m, 2H), 7.49–7.56 (m, 2H), 7.87–7.92 (m, 1H), 8.07–8.12 (m, 1H) ppm. ^13^C{^1^H} NMR (101 MHz, CDCl_3_): δ 14.3, 23.9, 28.9, 35.2, 49.4, 124.5, 124.7, 125.6, 125.7, 126.0, 128.0, 129.2, 132.4, 132.5, 138.8, 207.4 ppm. HRMS (MALDI–TOF): calcd for C_16_H_19_O [M + H]^+^ 227.1413; found, 227.1415.

## Supporting Information Available

Experimental procedures, general computational information, characterization data, NMR spectra of products, and Cartesians (PDF)
