# Supplementary material for: Electrophilic Trapping of Semibenzenes
Source: J Org Chem. 2022 Sep 12;87(19):12772–82. doi: 10.1021/acs.joc.2c01331 (PMC9552181; doi:10.1021/acs.joc.2c01331)
Supplement: Supplementary file 1 — jo2c01331_si_001.pdf [file jo2c01331_si_001.pdf]

Supplementary Materials for

Electrophilic trapping of semibenzenes

Cosimo Boldrini†, Marta Castiñeira Reis† and Syuzanna R. Harutyunyan†*

†Stratingh Institute for Chemistry, Nijenborgh 4, 9747 AG, Groningen (The Netherlands)

*Corresponding author. E-mail: s.harutyunyan@rug.nl

This pdf includes

Materials and Methods

Table S1 to S4

Figure S1 to S2

Scheme S1

Cartesians

Table of Contents

1. General experimental information 4

2. Chemicals 4

3. Computational details 5

4. Synthesis of starting materials 5

4.1 General procedure 1: synthesis of 1-chloromethyl-4-ethylnaphthalene derivatives 5

4.2 General procedure 2: Synthesis of 1-chloromethyl-4-Benzylnaphthalene derivatives 6

4.3. General procedure 3: synthesis of *p*-aryl compounds 8

4.4 General procedure 4: synthesis (6-methoxynaphthalene-1-yl)chloromethane 9

4.5. General procedure 5: synthesis of benzyl substituted compounds 10

5. Regioselectivity of the protonation and electrophilic activation of semibenzenes 16

6. Optimization of the trapping protocols with the different electrophiles....17

6.1. Procedures for the optimization of the solvent in the trapping protocols 19

7. Computational studies on the reorganization of semibenzene 19

8. General protocols for the trapping with electrophiles: 24

8.1 Trapping with TsOH 24

8.2 Trapping with Ph_3CBF_4 24

8.3 Trapping with tropylium tetrafluoroborate 25

8.4 Trapping with 1,3-Benzo dithiolylium tetrafluoroborate 26

8.5 Alkylation of benzo[d][1,3]dithiole derivatives 27

8.6 Reductive removal of benzothiol group 27

8.7 Oxidative removal of benzothiol group 27

9. Characterization of products 29

10. NMR spectra 57

11. Cartesian coordinates 160

12. Reference 219

1. General experimental information

All reactions using oxygen- and/or moisture-sensitive materials were carried out with anhydrous solvents under a nitrogen atmosphere using standard Schlenk techniques. Flash column chromatography was performed using Merck 60 Å 230–400 mesh silica gel. Thin layer chromatography was performed using 0.25 mm E. Merck silica plates (60F-254). Components were visualized by UV light and permanganate staining. Reactions were monitored by TLC. NMR data (^{1}H at 400 MHz; ^{13}C at 101 MHz) was collected on a Varian VXR400 machine equipped with a 5 mm z-gradient broadband probe. Chemical shifts are reported in parts per million (ppm) relative to residual solvent peak ($CDCl_3$, ^{1}H: 7.26 ppm; ^{13}C: 77.2 ppm). Coupling constants are reported in Hertz. Multiplicity is reported with the usual abbreviations (s: singlet, d: doublet, dd: doublet of doublets, t: triplet, q: quadruplet, m: multiplet). Exact mass spectra were recorded on a LTQ Orbitrap XL apparatus with ESI ionization or a 4800 MALDI TOF/TOF™ analyzer, exact masses are given for previously unreported compounds. The compounds here reported are known to fragment upon ionization to form rather stable ions.[1] For this reason, molecular ions are not always detectable. For these molecules, however, the formed fragments give clear and intense signals in mass analysis. Hence, the detection of these fragments together with NMR analysis allows for an unequivocal product characterization.

2. Chemicals

Unless otherwise indicated, reagents and substrates were purchased from commercial sources and used as received. Solvents not required to be dry were purchased as technical grade and used as received. Dry solvents were freshly collected from a dry solvent purification system prior to use. Inert atmosphere experiments were performed with standard Schlenk techniques with dried (P_2O_5) nitrogen gas. Grignard reagents and allyl$SnBu_3$ were purchased from Sigma-Aldrich. 1-(Chloromethyl)-naphthalene (**1f**), 4-methyl-1-(chloromethyl)-naphthalene (**1a**), 1-(Chloromethyl)-2-Methyl-naphthalene (**1g**), benzyl chloride (**1n**) and *p*-methyl-benzyl chloride (**1o**) were purchased from Sigma-Aldrich, other benzylic substrates were prepared following literature methods (references given below). Please note that characterization is only reported for the target halide. All reported compounds were characterized by ^{1}H and ^{13}C NMR and compared with literature data. All new compounds were

fully characterized by ^{1}H, ^{13}C NMR and HRMS techniques. Please note that trapping reactions were performed without an inert atmosphere and with wet solvents.

3. Computational details

We have used the Density Functional Theory (DFT) in the Kohn-Sham formulation[2] to optimize the stationary points presented and discussed along this manuscript. Specifically, the geometries of all the stationary points were fully optimized at the B3LYP[3]-GD3/def2svpp[4] computational level. The effect of the solvent was modeled using the polarizable continuum model (PCM)[5] with the default parameters implemented in the Gaussian09 package.[6] All geometry optimizations have been performed using tight convergence criteria in the SCF and requesting a pruned (99.590) grid to guarantee the accuracy of the reported results. Moreover, calculations were performed considering 1.0 atm and 298.25 K in accordance to the reaction conditions.

Harmonic analysis was used to establish the nature of all optimized structures as either minima or transition structures. For all stationary points, the stability of the wave function was also confirmed.[7]

IRC[8] calculations were conducted for transition states to ensure their connectivity with the expected reactants and products. When the substrates showed conformational freedom, conformational analysis was performed manually, it must be indicated that only the most stable conformer of each stationary point was considered and reported. The visualization of the reported structures was performed using MOLDEN.[9] The representation of the structures here presented were generated using CYLView.[10]

4. Synthesis of starting materials

4.1 General procedure 1: synthesis of 1-chloromethyl-4-ethylnaphthalene derivatives

Following a modified literature procedure.[11] In a dried two-necked round bottom flask, a solution of 4-ethyl-1-naphthoic acid (10.00 mmol, 1.0 equiv.) in dry THF (20 ml) was prepared, under nitrogen atmosphere. Then, it was added dropwise to a solution of $LiAlH_4$ (16.50 mmol, 1.6 equiv.) in dry THF (16 mL) at 0 °C. The resulting mixture was allowed to warm to r.t. and stirred for 16 h. The reaction was cooled to 0 °C and quenched by adding an aqueous solution

of KOH (1.0 M, 40 mL) dropwise. Then, Et_2O (60 mL) was added and the mixture stirred for 30 min at r.t. The aqueous and organic layers were separated and the combined aqueous layers extracted with Et_2O (50 mL x 2). The combined organic layers were washed with aqueous HCl (1.0 M, 50 mL) and brine (50 mL). Then, they were dried over $MgSO_4$, filtered and concentrated under reduced pressure in a rotatory evaporator to give the product, which was used in the next step without further purification.

Following a modified literature procedure.[12] In a dried two-necked round bottom flask, under nitrogen atmosphere, a solution of 4-ethyl-1-hydroxymethyl-naphthalene (9.50 mmol, 1.0 equiv.) in dry CH_2Cl_2 (50 mL) was prepared. It was cooled at 0 ℃ and $SOCl_2$ (19.00 mmol, 2.0 equiv.) was slowly added, the mixture was stirred for 5 h at 0 ℃. After the reaction was completed, an aqueous solution of $NaHCO_3$ was slowly added to quench the reaction (0.5 M, 50 mL). Then, Et_2O was added (100 mL), the mixture was washed with an aqueous solution of $NaHCO_3$ (0.5 M) until the pH of the aqueous layer was greater than 7. Then, the organic layer was washed with brine and dried over Na_2SO_4, filtered and concentrated under reduced pressure in a rotatory evaporator. The crude was subjected to column chromatography (silica gel, pentane).

4.2 General procedure 2: Synthesis of 1-chloromethyl-4-Benzylnaphthalene derivatives

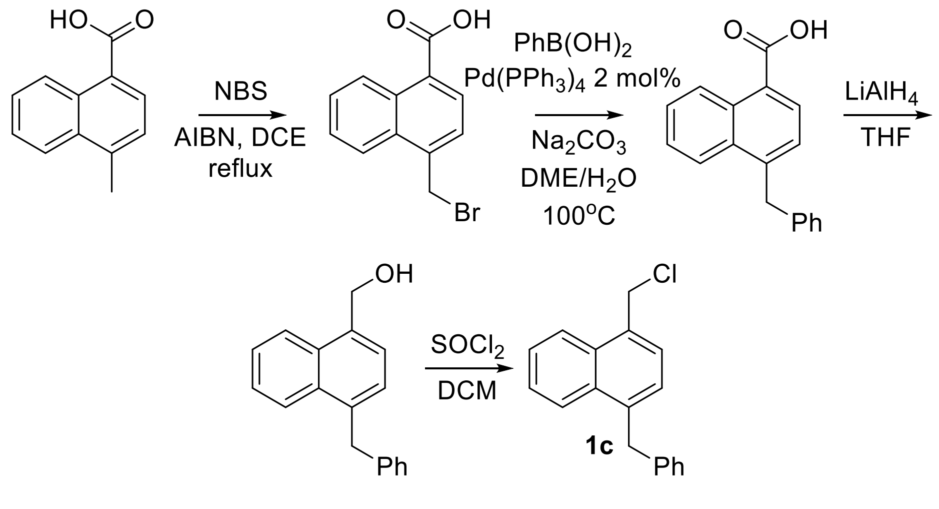

Following a modified literature procedure.[13] A mixture of 4-methyl-1-naphthoic acid (26.80 mmol, 1.0 equiv.), NBS (26.80 mmol, 1.0 equiv.), and AIBN (2 mol%) in DCE (100 mL) was refluxed for 3 h with oil bath heating. The reaction mixture was concentrated under reduced pressure in a rotatory evaporator and redissolved in EtOAc (50 mL). The organic solution was

washed with water (20 mL) and brine (20 mL), dried with $MgSO_4$, filtered and the solvent was removed under reduced pressure in a rotatory evaporator. The crude was used in the next step without further purification.

Following a modified literature procedure.[14] To a solution of benzyl bromide (4.00 mmol, 1.0 equiv.) in 1,2-dimethoxyethane (8 mL) and water (4 mL) was added boronic acid (4.80 mmol, 1.2 equiv.), Na_2CO_3 solution (8.40 mmol, 2.1 equiv.) and $Pd(PPh_3)_4$ (2 mol%). The mixture was deoxygenated under reduced pressure and flushed with nitrogen five times. Then heated to 100° C with an oil bath while stirring overnight. The solvent was removed under reduced pressure in a rotatory evaporator and the residue used in the next step without further purification.

Following a modified literature procedure.[11] In a dried round bottom flask, 4-benzyl-1-naphthoic acid (4.00 mmol, 1.0 equiv.) was dissolved in dry THF (16 mL), under nitrogen atmosphere. The solution was cooled to 0 °C and a solution of $LiAlH_4$ (6.60 mmol, 1.6 equiv.) in THF (6 mL) was added slowly. The reaction was then allowed to reach r.t. and stirred overnight. Water (10 mL) was initially added and then HCl (6.0 M) until acid pH. The organic layer was separated, dried over $MgSO_4$, filtered and the solvent was removed under reduced pressure in a rotatory evaporator. The crude was then purified by column chromatography (SiO_2, pentane:EtOAc 70:30) to obtain the desired product.

Following a modified literature procedure.[12] In a dried two-necked round bottom flask, a solution of the alcohol (1.70 mmol, 1.0 equiv.) in dry CH_2Cl_2 (10 mL) was prepared, under nitrogen atmosphere. Then, it was cooled at 0 °C and $SOCl_2$ (3.40 mmol, 2.0 equiv.) was slowly added, the mixture was stirred for 2 h at 0 °C. After the reaction was completed, aqueous $NaHCO_3$ solution (0.5 M, 20 mL) was slowly added to quench the reaction. Then, Et_2O was added (20 mL), the mixture was washed with an aqueous solution of $NaHCO_3$ (0.5 M) until the pH of the aqueous layer was greater than 7. Then the organic layer was washed with brine and dried over Na_2SO_4, filtered and concentrated under reduced pressure in a rotatory evaporator. The resulting crude was purified by column chromatography (SiO_2, pentane:EtOAc 90:10) to obtain the desired product.

4.3. General procedure 3: synthesis of *p*-aryl compounds

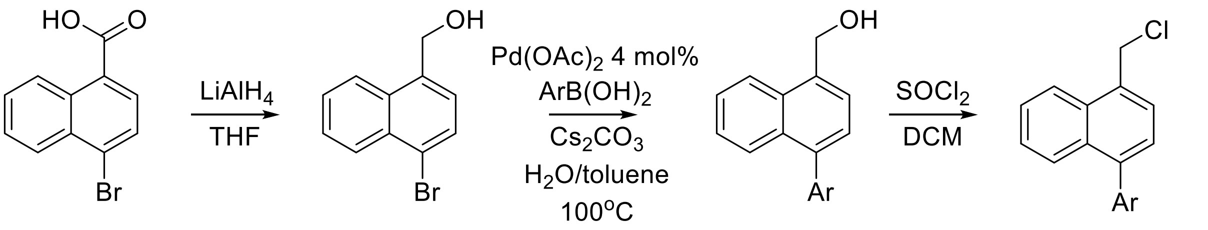

Following a modified literature procedure.[11] In a dried round bottom flask, the corresponding acid (60.00 mmol, 1.0 equiv.) was dissolved in dry THF (250 mL), under nitrogen atmosphere. To the mixture, a solution of $LiAlH_4$ (96.00 mmol, 1.6 equiv.) in THF (100 mL) was added slowly and the reaction was then heated to reflux overnight with oil bath heating. The mixture was allowed to cool to r.t. and quenched by slow addition of ice cold water (200 mL). Then the aqueous layer was acidified by addition of an aqueous HCl solution (6.0 M) and extracted with Et_2O (100 mL x 3). Then the organic layer was washed with brine and dried over Na_2SO_4, filtered and concentrated under reduced pressure. The crude was purified by column chromatography (SiO_2, pentane:EtOAc from 90:10 to 70:30) to give the corresponding alcohol as a white solid.

Following a modified literature procedure.[14] The corresponding alcohol (4.20 mmol, 1.0 equiv.) was dissolved in toluene (29 mL) and an aqueous Cs_2CO_3 solution was added (2.0 M, 27.00 mmol, 6.4 equiv.), under nitrogen atmosphere. Phenyl boronic acid (8.40 mmol, 2.0 equiv.) was dissolved in EtOH (13 mL) and added to the mixture. Then TBAB (Tetrabutylammonium bromide) (4.20 mmol, 1.0 equiv.) was added to the reaction mixture. The mixture was deoxygenated under reduced pressure and flushed with nitrogen for five times. $Pd(OAc)_2$ (4 mol%) was added and the resulting suspension was heated under reflux with oil bath heating for 8 h. After cooling, EtOAc (10 mL) and water (10 mL) were added and the organic layer separated. The aqueous layer was extracted with EtOAc (10 mL x 2). The combined organic layers were washed with brine, dried over Na_2SO_4, filtered over a short plug of Celite®, and the solvent was evaporated under reduced pressure. The crude was subjected to column chromatography (SiO_2, pentane:EtOAc 90:10) to give the corresponding arylated alcohol.

Following a modified literature procedure.[12] In a dried two-necked round bottom flask, a solution of the corresponding arylated alcohol (3.80 mmol, 1.0 equiv.) in dry CH_2Cl_2 (19 mL) was prepared, under nitrogen atmosphere. It was cooled to 0 °C and $SOCl_2$ (7.60 mmol, 2.0 equiv.) was slowly added, the mixture was stirred for 2 h at 0 °C. After the reaction was completed, an aqueous solution of $NaHCO_3$ was slowly added to quench the reaction (0.5 M, 20 mL). Then, Et_2O was added (20 mL), the mixture was washed with an aqueous solution of $NaHCO_3$ solution (0.5 M) until the pH of the aqueous layer was greater than 7. Then, the combined organic layers were washed with brine and dried over Na_2SO_4, filtered and the solvent evaporated under reduced pressure. The crude was subjected to column chromatography (SiO_2, pentane).

4.4 General procedure 4: synthesis (6-methoxynaphthalene-1-yl)chloromethane[15]

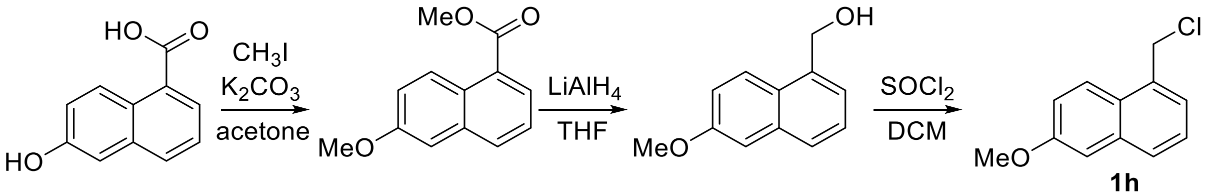

1h

The corresponding acid (13.30 mmol, 1.0 equiv.), K_2CO_3 (39.90 mmol, 3.0 equiv.) and MeI (39.90 mmol, 3.0 equiv.) were dissolved in acetone (30 mL) and the mixture was heated with an oil bath to 50 °C overnight, resulting in a heterogeneous mixture. This mixture was filtered (to remove the formed salts) and the solids were washed with acetone (20 mL). The solvent of the filtrate was evaporated under reduced pressure in a rotatory evaporator. Then, the residue partitioned in H_2O (20 mL) and CH_2Cl_2 (20 mL), the layers were separated and the aqueous one extracted once again with CH_2Cl_2 (20 mL). Then, the combined organic layers were dried over Na_2SO_4, filtered and the solvent was removed under reduced pressure in a rotatory evaporator. The product was used in the next step without further purification.

A solution of methyl 6-methoxy-1-naphthoate (13.00 mmol, 1.0 equiv.) in THF (20 mL) was prepared, under a nitrogen atmosphere. Then, it was added dropwise to a solution of $LiAlH_4$ (30.00 mmol, 2.3 equiv.) in dry THF (30 mL), previously cooled at 0°C. After complete addition, the mixture was allowed to reach r.t. and it was stirred for 2 h. Then, the mixture was cooled to 0 °C and poured into aqueous HCl (2.0 M, 40 mL). The mixture was evaporated to remove the THF and the residue was mixed with H_2O (30 mL). The resulting mixture was extracted

with CH_2Cl_2 (30 mL × 3), the combined extracts were washed with saturated brine (15 mL × 3), dried over $MgSO_4$, filtered and the solvent was removed under reduced pressure in a rotatory evaporator. After the solvent was evaporated, the crude product was used in the next reaction without purification.

In a dried two-necked round bottom flask, a solution of the corresponding alcohol (10.00 mmol, 1.0 equiv.) in dry CH_2Cl_2 (50 mL) was prepared, under nitrogen atmosphere. Then, it was cooled at 0 °C and $SOCl_2$ (20.00 mmol, 2.0 equiv.) was slowly added. The mixture was stirred for 2 h at 0 °C. After the reaction was completed, aqueous $NaHCO_3$ (0.5 M) solution was slowly added to quench the reaction (50 mL). Then, Et_2O was added (50 mL), the mixture was washed with an aqueous $NaHCO_3$ solution (0.5 M) until the pH of the aqueous layer was higher than 7. Then, the combined organic layers were washed with brine and dried over Na_2SO_4, filtered and evaporated under reduced pressure in a rotatory evaporator. Purification on column chromatography (SiO_2, pentane:EtOAc from 99:1 to 95:5) yielded the corresponding chloride.

4.5. General procedure 5: synthesis of benzyl substituted compounds[16]

In a dried two-necked round bottom flask, under nitrogen atmosphere, a solution of the corresponding aldehyde (11.00 mmol, 1.0 equiv.) in dry THF (15 mL) was prepared. Then, to this solution PhMgBr (16.50 mmol, 1.5 equiv.) was added dropwise. The mixture was stirred until reaction completion. Then, the reaction was quenched by adding a saturated solution of NH_4Cl (10 mL). The aqueous layer was extracted with EtOAc (20 mL x 2), the combined organic layers were washed with water (20 mL), and twice with brine (10 mL). Then, the combined organic layers were dried over Na_2SO_4, filtered and the solvent evaporated by using reduced pressure in a rotatory evaporator. The crude was used without further purification in the next step.

In a dried two-necked round bottom flask, a solution of the corresponding naphtalene (10.00 mmol, 1.0 equiv.) in dry CH_2Cl_2 (50 mL) was prepared, under nitrogen atmosphere. Then, it was cooled at 0 °C and $SOCl_2$ (20.00 mmol, 2.0 equiv.) was slowly added. The mixture was stirred for 2 h at 0 °C. After the reaction was completed, an aqueous $NaHCO_3$ solution was slowly added to quench the reaction (0.5 M, 50 mL). Then, Et_2O was added (50 mL). The mixture was washed with an aqueous $NaHCO_3$ solution (0.5 M) until the pH of the aqueous layer was higher than 7. Then, the combined organic layers were washed with brine and dried over Na_2SO_4,

filtered and evaporated under reduced pressure in a rotatory evaporator. Then, hexane (10 ml) was added to the crude, forming a hetereogeneous mixture. The insoluble residue in this mixture was filtered and the solvent of the mother liquour evaporated under reduced pressure in a rotatory evaporator to obtain the product.

4.6. General procedure 6: synthesis of (3-chloroprop-1-en-1-yl)naphthalenes[17]

In a dried two-necked round bottom flask, under nitrogen atmosphere, a solution of the corresponding aldehyde (15.00 mmol, 1.0 equiv.) in dry THF (15 mL) was prepared. This solution was cooled to 0°C and to it was added the Grignard (22.50 mmol, 1.5 equiv.) dropwise. The mixture was stirred until reaction completion, then quenched by adding a saturated solution of NH_4Cl (10 mL). The resulting aqueous layer was extracted with ethyl acetate. The combined organic layers were washed with water and twice with brine, then dried over Na_2SO_4. Purification on column chromatography (SiO_2, pentane:EtOAc from 90:10 to 80:20) yielded the corresponding alcohol.

In a dried two-necked round bottom flask, under nitrogen atmosphere, a solution of the corresponding hydroxy-naphtalene (11.60 mmol, 1.0 equiv.) in dry CH_2Cl_2 (58 mL) was prepared. Then, it was cooled at 0 ℃ and $SOCl_2$ (23.20 mmol, 2.0 equiv.) was slowly added. The mixture was stirred for 2 h at 0 ℃. After the reaction was completed, an aqueous $NaHCO_3$ solution (0.5 M, 50 mL) was slowly added to quench it. Then, Et_2O was added (100 mL), the mixture was washed with an aqueous $NaHCO_3$ solution (0.5 M) until the pH of the aqueous layer was greater than 7. Then, the organic layer was washed with brine and dried over Na_2SO_4, filtered and the solvent removed under reduced pressure in a rotatory evaporator. Pentane (5 mL) was added to the crude forming a heterogeneous mixture, this mixture was filtered in order to remove the solids. The mother liquor was then concentrated by using reduced pressure in a rotatory evaporator.

1-(Chloromethyl)-4-ethylnaphthalene (1b)[18]

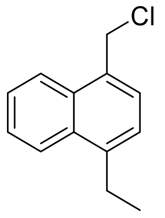

Following **general procedure 1**, compound **1b** was obtained as a white solid (1.47 g, 73% overall yield). The NMR data are in agreement with literature precedents.

^{1}H NMR (400 MHz, $CDCl_3$): δ 1.38 (t, *J*=7.5 Hz, 3H), 3.11 (q, *J*=7.5 Hz, 2H), 5.04 (s, 2H), 7.29 (d, *J*=7.3 Hz, 1H), 7.45 (d, *J*=7.3 Hz, 1H), 7.53-7.62 (m, 2H), 8.11 (d, *J*=8.0 Hz, 1H), 8.18 (d, *J*=8.0 Hz, 1H) ppm.

^{13}C{^{1}H} NMR (101 MHz, $CDCl_3$): δ 15.0, 26.1, 45.0, 124.3, 124.4, 124.6, 126.0, 126.2, 127.7, 131.1, 131.4, 132.3, 142.3 ppm.

1-Benzyl-4-(chloromethyl)naphthalene (1c)[18]

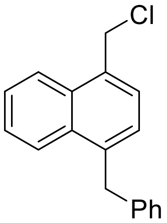

Following **general procedure 2**, compound **1c** was obtained as a white solid (437.3 mg, 41% yield from benzyl bromide intermediate). The NMR data are in agreement with literature precedents.

^{1}H NMR (400 MHz, $CDCl_3$): δ 4.46 (s, 2H), 5.06 (s, 2H), 7.18-7.32 (m, 6H), 7.45-7.64 (m, 3H), 8.07 (d, *J*=8.4 Hz, 1H), 8.19 (d, *J*=8.4 Hz, 1H) ppm.

^{13}C{^{1}H} NMR (101 MHz, $CDCl_3$): δ 39.2, 44.8, 124.3, 125.1, 126.2, 126.2, 126.4, 126.7, 127.5, 128.5 (2XC), 128.8 (2XC), 131.5, 131.9, 132.6, 138.6, 140.2 ppm.

1-(Chloromethyl)-4-phenylnaphthalene (1d)[18]

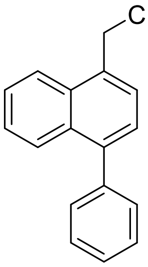

Following **general procedure 3** compound **1d** was obtained as a sticky oil (955.3 mg, 90% yield, starting from 4-bromo-1-hydroxymethyl-naphthalene). The NMR data are in agreement with literature precedents.

^{1}H NMR (400 MHz, $CDCl_3$): δ 5.10 (s, 2H), 7.37 (d, *J*=7.1 Hz, 1H), 7.41-7.52 (m, 6H), 7.57 (d, *J*=7.1 Hz, 1H), 7.61 (t, *J*=8.5 Hz, 1H), 7.93 (d, *J*=8.5 Hz, 1H), 8.21 (d, *J*=8.5 Hz, 1H) ppm.

^{13}C{^{1}H} NMR (101 MHz, $CDCl_3$): δ 47.4, 126.5, 128.8, 129.0, 129.2, 129.7, 129.9, 130.1, 131.0 (2XC), 132.7 (2XC), 134.0, 134.9, 135.0, 143.0, 144.7 ppm.

1-(Chloromethyl)-4-(4-methoxyphenyl)naphthalene (**1e**)[18]

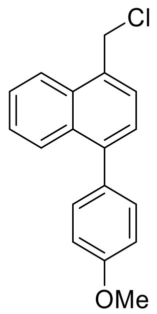

Following **general procedure 3**, compound **1e** was obtained as a white solid (950.1 mg, 80% yield from 4-bromo-1-hydroxymethyl-naphthalene). The NMR data are in agreement with literature precedents.

^{1}H NMR (400 MHz, $CDCl_3$): δ 3.90 (s, 3H), 5.11 (s, 2H), 7.02-7.07 (m, 2H), 7.37 (d, *J*=7.3 Hz, 1H), 7.39-7.43 (m, 2H), 7.46-7.52 (m, 1H), 7.57 (d, *J*=7.3 Hz, 1H), 7.60-7.65 (m, 1H), 7.98 (d, *J*=8.4 Hz, 1H), 8.22 (d, *J*=8.4 Hz, 1H) ppm.

^{13}C{^{1}H} NMR (101 MHz, $CDCl_3$): δ 44.8, 55.4, 113.8 (2XC), 123.9, 126.1, 126.3, 126.5, 127.1, 127.3, 131.1 (2XC), 131.4, 132.1, 132.4, 132.7, 141.8, 159.1 ppm.

1-(Chloromethyl)-6-methoxynaphthalene (1h)[18]

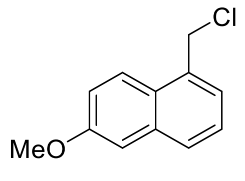

Following **general procedure 4** compound **1h** was obtained as a white solid (2.02 g, 74% overall yield). The NMR data are in agreement with literature precedents.

^{1}H NMR (400 MHz, $CDCl_3$): δ 3.93 (s, 3H), 5.02 (s, 2H), 7.19 (d, *J*=2.7 Hz, 1H), 7.24-7.29 (m, 1H), 7.36-7.42 (m, 2H), 7.72-7.77 (m, 1H), 8.06 (d, *J*=9.6 Hz, 1H) ppm.

^{13}C{^{1}H} NMR (101 MHz, $CDCl_3$): δ 44.6, 55.3, 106.8, 119.3, 125.2, 125.4, 125.9, 126.5, 128.7, 133.0, 135.3, 157.7 ppm.

1-(Chloro(phenyl)methyl)naphthalene (**1i**)[18]

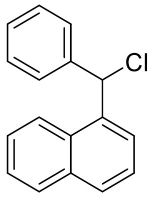

Following **general procedure 5** compound **1i** was obtained as a slightly yellow solid (2.39 g, 86% yield). The NMR data are in agreement with literature precedents.

^{1}H NMR (400 MHz, $CDCl_3$): δ 6.90 (s, 1H), 7.40-7.29 (m, 3H), 7.55-7.42 (m, 5H), 7.61 (d, *J*=6.90 Hz, 1H), 7.94-7.81 (m, 2H), 8.12-8.03 (m, 1 H) ppm.

^{13}C{^{1}H} NMR (101 MHz, $CDCl_3$): δ 61.7, 123.7, 125.2, 125.8, 126.5, 126.8, 128.0 (2XC), 128.1, 128.6 (2XC), 128.9, 129.3, 130.4, 133.9, 136.0, 140.4 ppm.

1-(1-Chlorobut-3-en-1-yl)naphthalene (**1j**)[18]

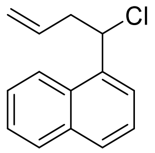

Following **general procedure 5** (employing allylMgBr), compound **1j** was obtained as a slightly yellow sticky oil (1.88 g, 79% yield). The NMR data are in agreement with literature precedents.

^{1}H NMR (400 MHz, $CDCl_3$): δ 3.03–3.07 (m, 2H), 5.08–5.26 (m, 2H), 5.64–5.76 (m, 1H), 5.90 (m, 1H), 7.45–7.62 (m, 3H), 7.70 (d, *J*=7.4 Hz, 1H), 7.79–7.92 (m, 2H), 8.14 (d, *J*=8.1 Hz, 1H) ppm.

$^{13}C\{^1H\}$ NMR (101 MHz, $CDCl_3$): δ 42.8, 58.6, 118.9, 122.8, 124.6, 125.2, 125.8, 126.5, 129.0, 129.1, 130.4, 133.8, 134.2, 136.3 ppm.

1-(1-Chloroethyl)naphthalene (**1k**)[18]

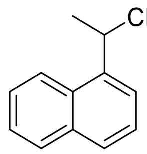

Following **general procedure 5** (but employing MeMgBr), compound **1k** was obtained as a slightly yellow oil (1.82 g, 87% yield). The NMR data are in agreement with literature precedents.

^1H NMR (400 MHz, $CDCl_3$): δ 2.06 (d, *J*=6.7 Hz, 3H), 5.91 (q, *J*=6.7 Hz, 1H), 7.46–7.54 (m, 2H), 7.56–7.62 (m, 1H), 7.72 (d, *J*=6.7 Hz, 1H), 7.83 (d, *J*=7.9 Hz, 1H), 7.89 (d, *J*=7.9 Hz, 1H), 8.19 (d, *J*=8.7 Hz, 1H) ppm.

$^{13}C\{^1H\}$ NMR (101 MHz, $CDCl_3$): δ 24.9, 54.4, 122.9, 123.4, 125.1, 125.7, 126.3, 128.8, 129.0, 130.3, 133.7, 137.4 ppm.

1-(Chloromethyl)-4-(*p*-tolyl)naphthalene (**1l**)[18]

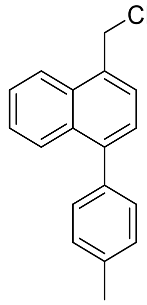

Following **general procedure 3**, compound **1l** was obtained as a white solid (750.1 mg, 67% yield, from 4-bromo-1-hydroxymethyl-naphthalene). The NMR data are in agreement with literature precedents.

^1H NMR (400 MHz, $CDCl_3$): δ 2.46 (s, 3H), 5.11 (s, 2H), 7.31 (d, *J*=7.3 Hz, 2H), 7.34-7.39 (m, 3H), 7.44-7.50 (m, 1H), 7.55-7.64 (m, 2H), 7.96 (d, *J*=8.4 Hz, 1H), 8.21 (d, *J*=8.4 Hz, 1H) ppm.

$^{13}C\{^1H\}$ NMR (101 MHz, $CDCl_3$): δ 21.2, 44.8, 123.8, 126.1, 126.3, 126.5, 127.2, 127.3, 129.0 (2XC), 129.9 (2XC), 131.4, 132.2, 132.3, 137.2, 137.4, 142.1 ppm.

(*E*)-1-(3-chloroprop-1-en-1-yl)naphthalene (1m)[17]

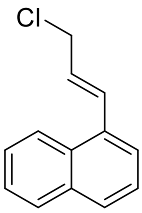

Following **general procedure 6** compound **1m** was obtained as a white off solid (2.10 g, 69% yield). The NMR data are in agreement with literature precedents.

^1H NMR (400 MHz, $CDCl_3$): δ 4.36 (dd, J_1 = 1.2 Hz, J_2 = 7.4 Hz, 2H), 6.32-6.41 (m, 1H), 7.40-7.64 (m, 5H), 7.79-7.89 (m, 2H), 8.07-8.13 (m, 1H) ppm.

$^{13}C\{^1H\}$ NMR (101 MHz, $CDCl_3$): δ 45.5, 123.6, 124.3, 125.6, 125.9, 126.3, 128.0, 128.6, 128.6, 131.1, 131.3, 133. 5, 133.6 ppm.

5. Regioselectivity of the protonation and electrophilic activation of semibenzenes

Initially, we wondered why the activation of the semibenzene core would take place in the external double bond and not in the internal one. To understand this aspect, we analyzed the APT charges of **III** and its bond distances. We observe that semibenzene has predominantly one contributing resonance form in which the terminal double bond is polarized (charges are 0.25 and -0.21 a.u.).

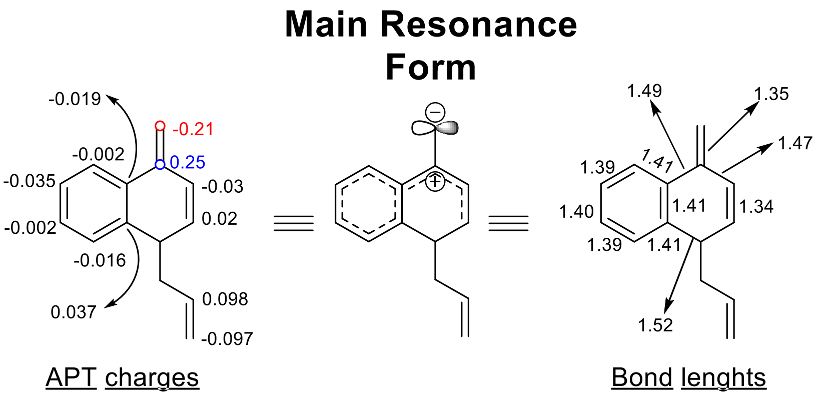

III

Figure S1. Analysis of the APT charges with hydrogens summed into heavy atoms and bond distances on semibenzene **III**.

Having identified why the terminal double bond is more reactive than the internal one, we moved to optimize the reaction conditions for its trapping with different electrophiles.

6. Optimization of the trapping protocols with the different electrophiles

The protocols for trapping the semibenzene derivatives formed in the reaction protocol, proved to be sensitive to the solvent used. Hence, they were subjected to solvent optimization (see below). We have obtained that in the case of using tropylium tetrafluoroborate, DMF provides the highest conversion to **7a**, independently of the dilution degree (**Table S1**). In the case of using 1,3-Benzo-dithiolylium tetrafluoroborate as electrophile, the solvent providing the highest conversion to **8a** is acetone (**Table S2**).

Table S1. Trapping with tropylium tetrafluoroborate

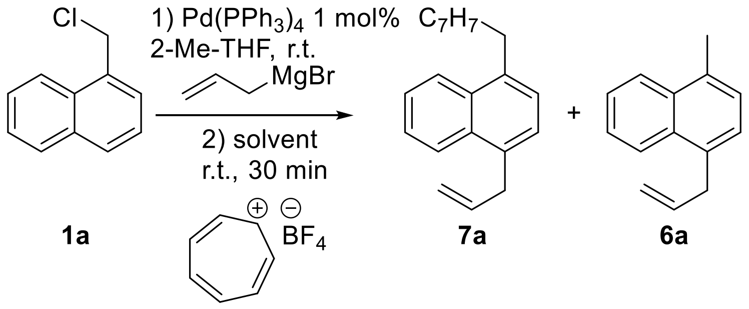

Entry	Solvent (mL)	Conversion (%)	Product ratio (7a:6a)
1	CH_2Cl_2 (9 mL)	Full	20:80
2	THF (9 mL)	Full	30:70

3	Acetone (9 mL)	Full	70:30
4	MeCN (9 mL)	Full	40:60
**5**	**DMF (9 mL)**	**Full**	**>99:trace**
**6**	**DMF (6 mL)**	**Full**	**>99:trace**
**7**	**DMF (3 mL)**	**Full**	**>99:trace**
**8**	**DMF (1.5 mL)**	**Full**	**>99:trace**

Table S2. Trapping with 1,3-Benzo dithiolylium tetrafluoroborate

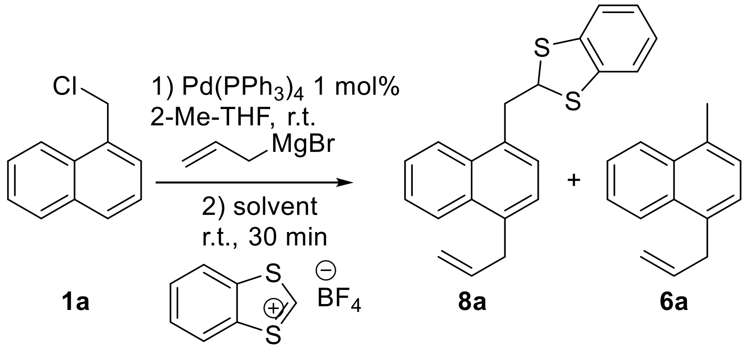

Entry	Solvent (mL)	Conversion (%)	Product ratio (7a:6a)
1	CH_2Cl_2 (9 mL)	full	30:30
2	MeCN (9 mL)	full	93:7
3	DMF (9 mL)	full	88:12
**4**	**Acetone (9 mL)**	**full**	**>99:trace**
**5**	**Acetone (6 mL)**	**full**	**>99:trace**
6	Acetone (3 mL)	full	95:5

6.1. Procedures for the optimization of the solvent in the trapping protocols

6.1.1. Trapping with tropylium tetrafluoroborate

To an oven dried Schlenk, under nitrogen atmosphere, were added the substrate **1a** (0.30 mmol, 1.0 equiv.), $Pd(PPh_3)_4$ (1 mol%) and dry 2-Me-THF (1 mL) and the mixture was stirred for 5 minutes. Allyl magnesium bromide (1.0 M in Et_2O, 0.36 mmol, 1.2 equiv.) was added at once and the mixture stirred at r.t. until the substrate was fully consumed (TLC check, finished in 15 minutes). After complete consumption of the substrate, pentane (20 mL) was added to precipitate out the salts and the suspension was filtered through a plug of Celite®. The mother liquour was evaporated under reduced pressure in a rotatory evaporator. This crude was redissolved in CH_2Cl_2 (0.5 mL) and added dropwise to a stirred solution of tropylium tetrafluoroborate (0.45 mmol, 1.5 equiv.) in the specified solvent at r.t. The mixture was stirred for 30 minutes. Then the solvent was evaporated under reduced pressure. The product ratio was determined by ^{1}H NMR of the crude mixture. Results are shown in **Table S1.**

6.1.2. Trapping with 1,3-benzodithiolylium tetrafluoroborate

To an oven dried Schlenk, under nitrogen atmosphere, were added the substrate **1a** (0.30 mmol, 1.0 equiv.), $Pd(PPh_3)_4$ (1 mol%) and dry 2-Me-THF (1 mL). The mixture was stirred for 5 minutes. Allyl magnesium bromide (1.0 M in Et_2O, 0.36 mmol, 1.2 equiv.) was added at once and the mixture stirred at r.t. until the substrate was fully consumed. After complete consumption of the substrate, pentane (20 mL) was added to precipitate out the salts and the suspension was filtered through a plug of Celite®. Evaporation of the solvent yielded the crude of the dearomatized product, which was dissolved in CH_2Cl_2 (0.5 mL) and added dropwise to a stirred solution of 1,3-Benzo dithiolylium tetrafluoroborate (0.39 mmol, 1.3 equiv.) in the specified solvent at r.t. The mixture was stirred for 30 minutes at r.t., then the solvent was evaporated under reduced pressure. The product ratio was determined by ^{1}H NMR of the crude mixture. Results are shown in **Table S2**.

7. Computational studies on the reorganization of semibenzene

To shed some light in the reorganization of the semibenzenes described in **Table 1** and rationalize the regioselectivity observed in their reorganization, we resorted to DFT. We have found that the selectivity of the 1,2-migrations of the different substituents at the tertiary carbon depends on the

ability of the group to stabilize a charge deficiency at the transition state structure. The better its ability to stabilize the charge, the greater its migration ability and viceversa. Specifically, DFT results show that allyl group migrates preferentially than Me and Ph, however *p*-OMePh outperforms them as migrating group (**Figure S2a**). The analysis of the LUMOS of the associated transition state structures sheds light on this aspect. We can see in **Figure S2b** that the LUMOS are centered on the migrating group, hence the more the ability of the group to stabilize a deficit of the electron density, the lower the energy barrier.

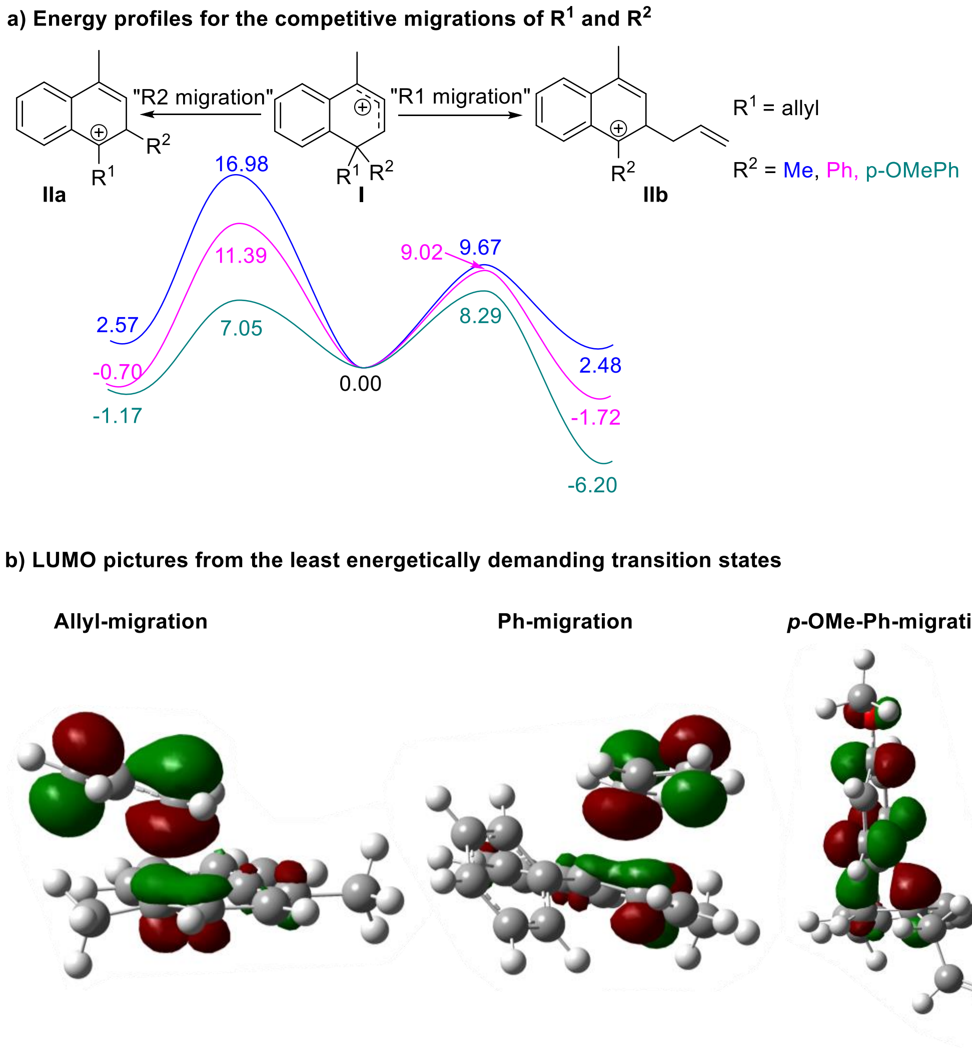

Figure S2. a) Analysis of the competitive energy paths for the migration for R^1 and R^2 and b) cartoon of the LUMO orbitals obtained for the isosurface 0.05 with a density of 0.0004 a.u.

Table S3. Summary of the energies for the competitive migrations depicted in **Figure S2**.[a]

ID	ImFreqs	Stable	SCF_LL (B3LYP-GD3/def2svpp)	SCF_HL (B3LYP-GD3/def2tzvpp)	Correction = SCF_HL-SCF_LL	SCF+ZPVE	H[b]	G[c]	Gcorr (G+correction)

**I-Me**	-	Yes	-581.1921703	-581.8641823	-0.672011965	-580.916	-580.901	-580.957	-581.628955
**TS-(I-IIa-Me)**	-88.37	Yes	-581.1748408	-581.8486043	-0.673763499	-580.9	-580.886	-580.94	-581.6135425
**IIa-Me**	-	Yes	-581.1891032	-581.8608685	-0.671765252	-580.913	-580.898	-580.953	-581.6250063
**TS-(I-IIb-Me)**	-422.73	Yes	-581.163667	-581.8372357	-0.673568728	-580.889	-580.874	-580.928	-581.6019017
**IIb-Me**	-	Yes	-581.1883049	-581.8605007	-0.67219579	-580.912	-580.897	-580.953	-581.6248608
**I-Ph**	-	Yes	-772.7926217	-773.6717056	-0.879083865	-772.464	-772.445	-772.509	-773.3876969
**TS-(I-IIa-Ph)**	-102.08	Yes	-772.775592	-773.6567121	-0.881120143	-772.448	-772.43	-772.492	-773.3733261
**IIa-Ph**	-	Yes	-772.7967025	-773.6752944	-0.878591924	-772.467	-772.449	-772.512	-773.3904369
**TS-(I-IIb-Ph)**	-267.45	Yes	-772.7756366	-773.6534032	-0.877766602	-772.448	-772.43	-772.492	-773.3695526
**IIb-Ph**	-	Yes	-772.7922708	-773.67181	-0.87953923	-772.463	-772.445	-772.509	-773.3888132
**I-*p*OMePh**	-	Yes	-887.2325837	-888.2488374	-1.016253771	-886.871	-886.85	-886.919	-887.9354748
**TS-(I-IIa-*p*OMePh)**	-114.75	Yes	-887.2163524	-888.2345529	-1.018200487	-886.856	-886.836	-886.904	-887.9222685
**IIa-*p*OMePh**	-	Yes	-887.2433527	-888.2596511	-1.016298451	-886.881	-886.86	-886.929	-887.9453495
**TS-(I-IIb-*p*OMePh)**	-96.78	Yes	-887.2247813	-888.238841	-1.014059651	-886.863	-886.844	-886.91	-887.9242327
**IIb-*p*OMePh**	-	Yes	-887.2324071	-888.249145	-1.016737924	-886.871	-886.85	-886.921	-887.9373389

[a]Energies are expressed in a.u. and the imaginary frequencies in cm^{-1}. [b]H represents enthalpies. [c]G Represents Gibbs free Energies

Additionally, the different migration patterns obtained for **5a** and **5d** puzzled us, since in principle, they should behave analogously, *i.e.* expectedly they should all show a single 1,2 migration of the allyl group. However, **5a** rearomatizes before the migration occurs. To get some insight into this, we explore the reactivity of the substrates after protonation.

In the case of **5a**, DFT reveals that despite the migration of the allyl group being feasible, the rearomatization of the scaffold is faster and renders a thermodynamically preferred product. Hence, the formation of **5a** is the only reorganization observed experimentally.

In the case of **5d**, DFT results indicate that the migration of the allyl moiety is extremely fast (6.15 kcal/mol), and once it happens the product is quickly quenched via deprotonation by water. A second migration of the allyl group could be operative, however it is overruled by the rearomatization similarly to the reactivity found in **5a**.

Thus, changing the tertiary sp^3 center for a quaternary one favors the migration of the allyl group over the rearomatization of the structure.

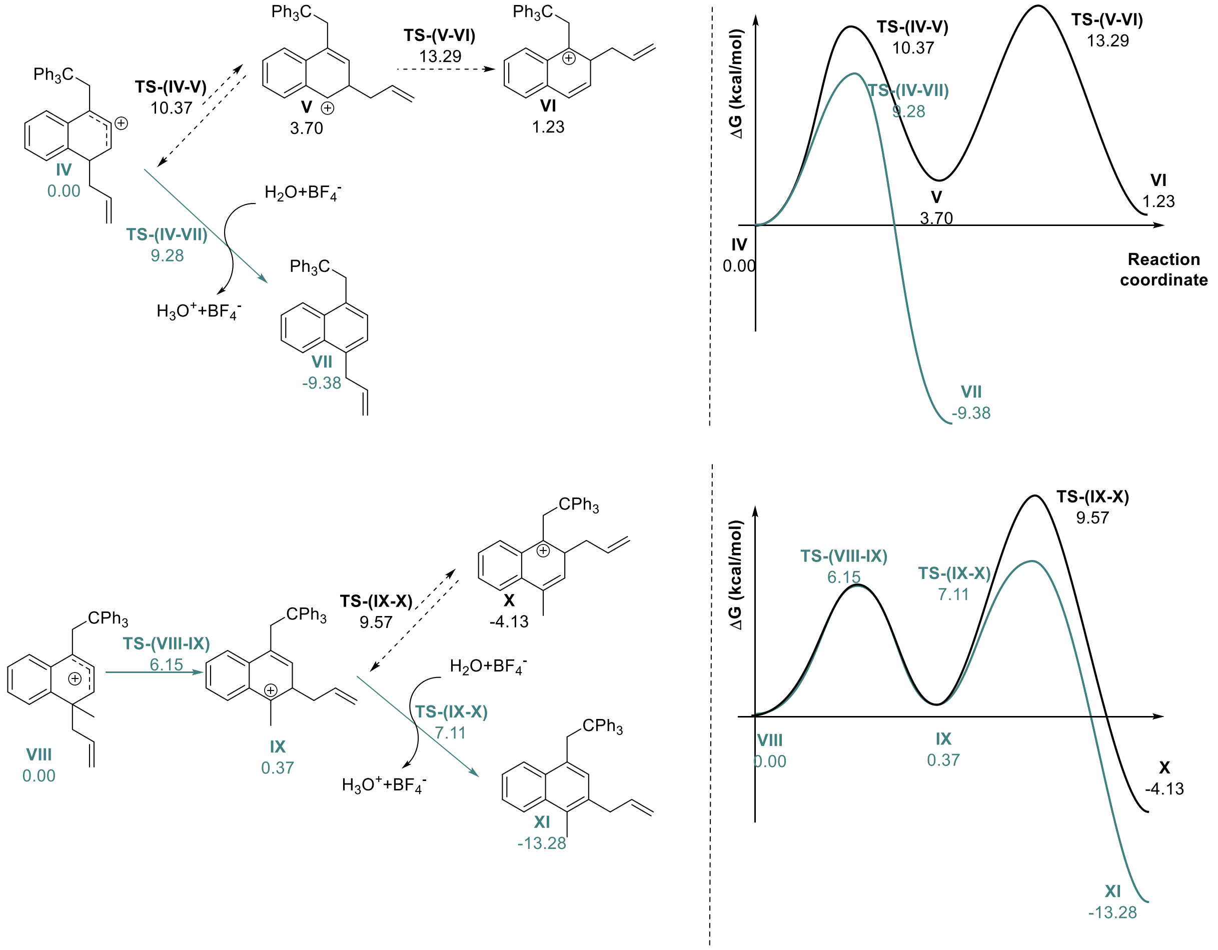

Scheme S1. DFT results on the exploration of the reactivity of **5a** and **5d**. Right: Reaction schemes, left: energy profiles.

Table S4. Summary of the energies for the competitive migrations depicted in **Scheme S1.**

ID	ImFreqs	Stable	SCF_LL (B3LYP-GD3/def2svpp)	SCF_HL (B3LYP-GD3/def2tzvpp)	Correction = SCF_HL-SCF_LL	SCF+ZPVE	H[b]	G[c]	Gcorr (G+correction)
**BF_4**	-	Yes	-424.2825302	-424.8196976	-0.53716741	-424.268	-424.263	-424.293	-424.8305014
**H_2O**	-	Yes	-76.34557301	-76.47224041	-0.126667398	-76.325	-76.3213	-76.3427	-76.4693834
**IV**	-	Yes	-1273.845933	-1275.281833	-1.4359003	-1273.32	-1273.3	-1273.38	-1274.819489

**TS-(IV-V)**	-148.44	Yes	-1273.827305	-1275.264567	-1.43726167	-1273.31	-1273.28	-1273.37	-1274.802958
**V**	-	Yes	-1273.838102	-1275.273776	-1.43567427	-1273.32	-1273.29	-1273.38	-1274.813598
**TS-(IV-VII)**	-377.52	Yes	-1350.205829	-1351.755514	-1.54968518	-1349.66	-1349.63	-1349.72	-1351.274083
**VII**	-	Yes	-1774.557234	-1776.627174	-2.06994009	-1774	-1773.96	-1774.06	-1776.134319
**VIII**	-	Yes	-1313.130458	-1314.614055	-1.48359681	-1312.58	-1312.55	-1312.64	-1314.123868
**TS-(VIII-IX)**	-77.75	Yes	-1313.116437	-1314.601537	-1.48509991	-1312.57	-1312.54	-1312.63	-1314.114069
**IX**	-	Yes	-1313.128546	-1314.612016	-1.48347022	-1312.58	-1312.55	-1312.64	-1314.123276
**TS-(IX-X)**	-91.61	Yes	-1313.110898	-1314.595867	-1.48496926	-1312.56	-1312.53	-1312.62	-1314.108619
**X**	-	Yes	-1313.135444	-1314.618888	-1.48344375	-1312.59	-1312.56	-1312.65	-1314.130457
**TS-(IX-XI)**	-125.74	Yes	-1389.492437	-1391.089896	-1.59745891	-1388.92	-1388.89	-1388.98	-1390.581923
**XI**	-	Yes	-1813.843182	-1815.962204	-2.11902171	-1813.25	-1813.22	-1813.33	-1815.444918

[a]Energies are expressed in a.u. and the imaginary frequencies in cm^{-1}. [b]H represents enthalpies. [c]G Represents Gibbs free Energies

8. General protocols for the trapping with electrophiles:

8.1 Trapping with TsOH

To an oven dried Schlenk, were added the substrate (0.30 mmol, 1.0 equiv.), $Pd(PPh_3)_4$ (1 mol%) and dry 2-Me-THF (1 mL) and the mixture was stirred for 5 minutes, under nitrogen atmosphere. Allyl magnesium bromide (375 µL, 1.0 M in Et_2O, 1.25 equiv.) was added at once and the mixture stirred at r.t. until completion (TLC check, finished in 15 minutes). After complete consumption of the substrate, *p*-TsOH·H_2O (0.60 mmol, 2.0 equiv.) was added and the mixture was stirred for an additional 10 minutes. Then, a saturated solution of $NaHCO_3$ (10 mL) was added, and the aqueous layer was extracted with Et_2O (10 mL x 3). The combined organic layers were dried over Na_2SO_4, filtered and concentrated under reduced pressure to give the crude rearomatized compound, which was then purified by column chromatography (silica gel) using pentane or a mixture of pentane and EtOAc as an eluent.

8.2 Trapping with Ph_3CBF_4

Method A. Generation of semibenzenes with allyl Grignard:

To an oven dried Schlenk, were added the substrate (0.30 mmol, 1.0 equiv.) and $Pd(PPh_3)_4$ (1 mol%), then dry 2-Me-THF (1 mL) was added and the mixture was stirred for 5 minutes, under nitrogen atmosphere. Allyl magnesium bromide (375 µL, 1.0 M in Et_2O, 1.25 equiv.) was added at once and the mixture stirred at r.t. until completion (TLC check, finished in 15 minutes). After complete consumption of the substrate, pentane (20 mL) was added to precipitate out the salts and the suspension was filtered through a plug of Celite®. Evaporation of the solvent yielded the crude dearomatized product, which was dissolved in CH_2Cl_2 (0.5 mL) and added dropwise to a stirred solution of Ph_3CBF_4 (0.33 mmol, 1.1 equiv.) in MeCN (6 mL) at r.t. The mixture was stirred for 30 minutes at r.t. Then, the solvent was evaporated under reduced pressure and the crude product was purified by column chromatography (silica gel) using a mixture of pentane and CH_2Cl_2 as an eluent.

Method B. Generation of semibenzenes with allyl stannane:

To an oven dried Schlenk, $Pd(PPh_3)_4$ (10 mol%) was dissolved in CH_2Cl_2 (3 mL), then the substrate was added (0.30 mmol, 1.0 equiv.) and the mixture was stirred for 5 minutes, under

nitrogen atmosphere. $AllylSnBu_3$ (0.30 mmol, 1.0 equiv.) was added at once and the mixture stirred at r.t. until completion (TLC checks). After complete consumption of the substrate, the solvent was evaporated and the crude was filtered through a plug of basic alumina (Ø = 1 cm, h~8-10 cm) using pentane as eluent (~100 mL). After evaporation of the solvent, the crude was dissolved in CH_2Cl_2 (0.5 mL) and added dropwise to a stirred solution of Ph_3CBF_4 (0.33 mmol, 1.1 equiv.) in MeCN (6 mL) at r.t. The mixture was stirred for 30 minutes at r.t., then the solvent was evaporated under reduced pressure and the crude product was purified by column chromatography (silica gel) using a mixture of pentane and CH_2Cl_2 as an eluent.

8.3 Trapping with tropylium tetrafluoroborate

Method C. Generation of semibenzenes with allyl Grignard:

To an oven dried Schlenk, were added the substrate (0.30 mmol, 1.0 equiv.) and $Pd(PPh_3)_4$ (1 mol%), then dry 2-Me-THF (1 mL) was added and the mixture was stirred for 5 minutes, under nitrogen atmosphere. AllylMgBr (375 µL, 1.0 M in Et_2O, 1.25 equiv.) was added at once and the mixture stirred at r.t. until the substrate is consumed completely (TLC check, finished in 15 minutes). After, pentane (20 mL) was added to precipitate out the salts and the suspension was filtered through a plug of Celite®. Evaporation of the solvent yielded the crude dearomatized product, which was dissolved in CH_2Cl_2 (0.5 mL) and added dropwise to a stirred solution of tropylium tetrafluoroborate (0.45 mmol, 1.5 equiv.) in DMF (3 mL) at r.t. The mixture was stirred for 30 minutes at r.t., then water (10 mL) and Et_2O were added (10 mL), the mixture stirred for 5 minutes and the layers were separated. The aqueous layer was extracted with Et_2O (10 mL x 2) and the combined organic layers were dried over Na_2SO_4, filtered and concentrated under reduced pressure. The crude product was purified by column chromatography (SiO_2) using a mixture of pentane and CH_2Cl_2 as an eluent.

Method D. Generation of semibenzenes with allyl stannane:

To an oven dried Schlenk, $Pd(PPh_3)_4$ (10 mol%) was dissolved in CH_2Cl_2 (3 mL), then the substrate was added (0.30 mmol, 1.0 equiv.) and the mixture was stirred for 5 minutes, under nitrogen atmosphere. $AllylSnBu_3$ (0.30 mmol, 1.0 equiv.) was added at once and the mixture stirred at r.t. until the substrate is fully consumed. After, the solvent was evaporated under reduced pressure in a rotatory evaporator and the crude was passed through a small amount

of basic alumina (Ø = 1 cm, h~8-10 cm) using pentane as eluent (~100 mL). After evaporation of the solvent, the crude was dissolved in CH_2Cl_2 (0.5 mL) and added dropwise to a stirred solution of tropylium tetrafluoroborate (0.45 mmol, 1.5 equiv.) in DMF (3 mL) at r.t. The mixture was stirred for 30 minutes at r.t., then water (10 mL) and Et_2O (10 mL) were added. Then, the resulting mixture was stirred for 5 minutes and the layers were separated. The aqueous layer was extracted with Et_2O (10 mL x 2) and the combined organic layers were dried over Na_2SO_4, filtered and concentrated under reduced pressure. The crude product was purified by column chromatography (SiO_2) using a mixture of pentane and CH_2Cl_2 as an eluent.

8.4 Trapping with 1,3-Benzo dithiolylium tetrafluoroborate

Method E. Generation of semibenzenes with allyl Grignard:

To an oven dried Schlenk, under nitrogen atmosphere, were added the substrate (0.30 mmol, 1.0 equiv.) and $Pd(PPh_3)_4$ (1 mol%), then dry 2-Me-THF (1 mL) was added and the mixture was stirred for 5 minutes. Allyl magnesium bromide (375 µL, 1.0 M in Et_2O, 1.25 equiv.) was added at once and the mixture stirred at r.t. until completion (TLC check, finished in 15 minutes). After complete consumption of the substrate, pentane (20 mL) was added to precipitate out the salts and the suspension was filtered through a plug of Celite®. Evaporation of the solvent yielded the crude dearomatized product, which was dissolved in 0.5 mL of CH_2Cl_2 and added dropwise to a stirred solution of 1,3-Benzo dithiolylium tetrafluoroborate (0.39 mmol, 1.3 equiv.) in acetone (6 mL) at r.t. The mixture was stirred for 30 minutes at r.t., then the solvent was evaporated under reduced pressure and the crude product was purified by column chromatography (SiO_2) using a mixture of pentane and CH_2Cl_2 as an eluent.

Method F. Generation of semibenzenes with allyl stannane:

To an oven dried Schlenk, under nitrogen atmosphere, $Pd(PPh_3)_4$ (10 mol%) was dissolved in CH_2Cl_2 (3 mL), then the substrate was added (0.30 mmol, 1.0 equiv.) and the mixture was stirred for 5 minutes. $AllylSnBu_3$(0.30 mmol, 1.0 equiv.) was added at once and the mixture stirred at r.t. until the substrate is fully consumed. After, the solvent was evaporated and the crude was passed through a small amount of basic alumina (Ø = 1 cm, h~8-10 cm) using pentane as eluent (~100 mL). After evaporation of the solvent, the crude was dissolved in 0.5 mL of CH_2Cl_2 and added dropwise to a stirred solution of 1,3-Benzo dithiolylium

tetrafluoroborate (0.39 mmol, 1.3 equiv.) in acetone (6 mL) at r.t. The mixture was stirred for 30 minutes at r.t., then the solvent was evaporated under reduced pressure and the crude product was purified by column chromatography (SiO_2) using a mixture of pentane and CH_2Cl_2 as an eluent.

8.5 Alkylation of benzo[d][1,3]dithiole derivatives

Following a literature procedure,[19] a solution of *n*BuLi (2.5 M in hexanes, 0.50 mmol, 1.05 equiv.) was added dropwise to a solution of 2-((4-allylnaphthalen-1-yl)methyl)benzo[d][1,3]dithiole (**8f**) (0.50 mmol, 1.0 equiv.) in anhydrous in THF (5 mL) at 0°C. The mixture turns to a deep blue color. After 5 minutes MeI (1.00 mmol, 2.0 equiv.) was added and the solution slowly turns to pale yellow. The solution was stirred for 5 minutes and then water (5 mL) was added. The organic layer was separated, and the aqueous layer was extracted with Et_2O (10 mL x 2). The collected organic layers were washed with brine (10 mL), dried over Na_2SO_4 and concentrated under reduce pressure. The crude product was purified by column chromatography (SiO_2) using a mixture of pentane and CH_2Cl_2 as an eluent.

8.6 Reductive removal of benzothiol group

Following a literature procedure,[19] to a solution of **9fa** (0.10 mmol, 1.0 equiv.) in ethanol (2 mL), Ni-Raney (0.50 g, slurry in water) was added and the reaction was kept under H_2 atmosphere (1.0 atm). After 3 h the reaction mixture was filtered through a plug of Celite® and the organic solvent was removed under reduced pressure. The residue was diluted with AcOEt (10 mL), the organic layer was separated, and the aqueous layer was extracted with AcOEt (10 mL x 2). The collected organic layers were washed with brine (10 mL), dried over Na_2SO_4, filtered and the solvent was removed under reduce pressure in a rotatory evaporator. The crude product was purified by column chromatography (SiO_2) using pentane as an eluent.

8.7 Oxidative removal of benzothiol group

After two vacuum/H_2 cycles, to replace air inside reaction tube with hydrogen, the mixture of the substrate **9a** (0.20 mmol, 1.0 equiv.), 10% Pd/C (10 wt % of the substrate) in MeOH (2 mL) was vigorously stirred at room temperature under H_2 atmosphere for 24 h. The reaction

mixture was filtered through a plug of Celite® and the filtrate was concentrated to provide the product, which was used in the next step without further purification.

Following a literature procedure,[19] to a suspension of HgO (0.40 mmol, 2.0 equiv.) in THF 48% solution of HBF_4 in water was added (200 µL). After 5 minutes a solution of dithiane (in 1 mL THF) was slowly added and the precipitated dissolved. After 30 minutes a saturated solution of $NaHCO_3$ was slowly added at 0°C until basic pH. The solid was filtered through a plug of Celite®, the organic solvent was evaporated and the residue was diluted with AcOEt (10 mL). The organic layer was separated, and the aqueous layer was extracted with AcOEt (10 mL x 2). The collected organic layers were washed with brine (10 mL), dried over Na_2SO_4 and concentrated under reduce pressure. The crude product was purified by column chromatography (SiO_2) using a mixture of pentane and CH_2Cl_2 as an eluent.

9. Characterization of products

2-Allyl-1,4-dimethylnaphthalene (**3a**)[20]

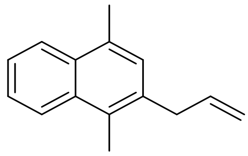

The compound was synthesized using the general procedure for the trapping with TsOH, the crude compound was purified by column chromatography (SiO_2, pentane) giving **3a** as a colorless oil (37.7 mg, 64% yield)

^{1}H NMR (400 MHz, $CDCl_3$): δ 2.60 (s, 3H), 2.67 (s, 3H), 3.58 (d, *J*= 6.2 Hz, 2H), 4.96-5.10 (m, 2H), 5.97-6.08 (m, 1H), 7.16 (s, 1H), 7.47-7.57 (m, 2H), 7.98-8.01 (m, 1H), 8.07-8.10 (m, 1H) ppm.

^{13}C{^{1}H} NMR (101 MHz, $CDCl_3$): δ 14.2, 19.3, 38.6, 115.3, 124.5 (2xC), 124.6, 125.4, 129.3, 129.4, 131.6, 132.0, 133.2, 134.3, 137.0 ppm.

2-Allyl-1-ethyl-4-methylnaphthalene (**3ba**) + **2-allyl-4-ethyl-1-methylnaphthalene** (**3bb**)

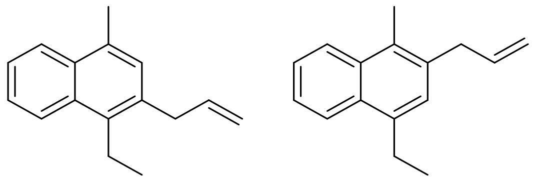

The compound was synthesized using the general procedure for the trapping with TsOH, the crude compounds was purified by column chromatography (SiO_2, pentane), giving an non isolable mixture of **3ba** and **3bb** (8:2 regioisomer ratio) as a colorless oil (53.0 mg, 84% yield).

^{1}H NMR (400 MHz, $CDCl_3$): δ 1.32 (t, *J*= 7.5 Hz, 3H, major), 1.40 (t, *J*= 7.5 Hz, 3H, minor), 2.62 (s, 3H, minor), 2.68 (s, 3H, major), 3.07-3.16 (m, 2H minor + 2H major), 3.56-3.63 (m, 2H minor + 2H major), 4.99-5.13 (m, 2H minor + 2H major), 6.00-6.14 (m, 1H minor + 1H major), 7.17-7.21 (m, 1H minor + 1H major), 7.48-7.59 (m, 2H minor + 2H major), 7.99-8.14 (m, 2H minor + 2H major) ppm.

^{13}C{^{1}H} NMR (101 MHz, $CDCl_3$): δ 14.3 (minor), 15.3 (minor), 15.4 (major), 19.4 (major), 21.2 (major), 25.9 (minor), 37.8 (major), 38.7 (minor), 115.4 (minor), 115.5 (major), 124.1 (minor),

124.4 (major), 124.6 (major), 124.7 (minor x 2 + 1 major), 125.4 (minor), 125.5 (major), 127.6 (minor), 129.3 (major), 129.4 (minor), 130.8 (minor), 132.0 (major), 132.1 (major), 132.2 (major), 133.4 (minor), 133.7 (major), 134.4 (minor), 135.6 (major), 137.0 (minor), 137.6 (major), 138.1 (minor) ppm.

HRMS(ESI+, M/Z): Calcd. for $C_{16}H_{17}$ $[M-H]^+$ 209.1323, found 209.1325

1-Allyl-2-benzyl-4-methyl-naphthalene (4c)

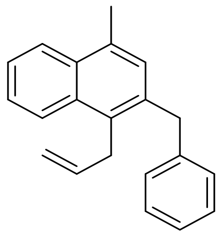

The compound was synthesized using the general procedure for the trapping with TsOH, the crude compound was purified by column chromatography (SiO_2, pentane), giving **4c** as a colorless oil (58.8 mg, 72% yield).

^{1}H NMR (400 MHz, $CDCl_3$): δ 2.67 (s, 3H), 3.85 (d, *J*= 5.6 Hz, 2H), 4.19 (s, 2H), 4.90-4.98 (m, 1H), 5.01-5.06 (m, 1H), 5.96-6.07 (m, 1H), 7.15-7.23 (m, 4H), 7.26-7.32 (m, 2H), 7.49-7.57 (m, 2H), 8.00-8.10 (m, 2H) ppm.

^{13}C{^{1}H} NMR (101 MHz, $CDCl_3$): δ 19.4, 32.5, 39.2, 115.6, 124.6, 124.8, 124.9, 125.7, 126.0, 128.4 (2xC), 128.7 (2xC), 130.0, 131.2, 132.0, 132.8, 132.9, 135.7, 136.6, 141.0 ppm.

HRMS(ESI+, M/Z): Calcd. for $C_{21}H_{21}$ $[M+H]^+$ 273.1643, found 273.1644.

1-Allyl-2-(4-methoxyphenyl)-4-methylnaphthalene (**4d**)

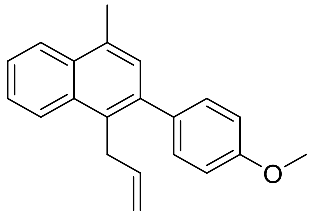

The compound was synthesized using the general procedure for the trapping with TsOH, the crude compound was purified by column chromatography (SiO_2, pentane:CH_2Cl_2 95:5) giving **4d** as a colorless oil (60.6 mg, 70% yield).

^{1}H NMR (400 MHz, $CDCl_3$): δ 2.71 (s, 3H), 3.74-3.79 (m, 2H), 3.88 (s, 3H), 4.84-4.91 (m, 1H), 5.06-5.11 (m, 1H), 6.07-6.18 (m, 1H), 6.98 (d, *J*= 8.8 Hz, 2H), 7.28 (s, 1H), 7.35 (d, *J*= 8.8 Hz, 2H), 7.51-7.58 (m, 2H), 8.03-8.12 (m, 2H) ppm.

^{13}C{^{1}H} NMR (101 MHz, $CDCl_3$): δ 19.4, 33.7, 55.3, 113.4 (2xC), 115.9, 124.6, 125.1, 125.8 (2xC), 129.5, 130.3 (2xC), 130.4, 132.2, 132.5, 132.6, 134.9, 138.1, 139.0, 158.6 ppm.

HRMS(ESI+, M/Z): Calcd. for $C_{21}H_{21}O$ $[M+H]^+$ 289.1586, found 289.1583.

2-Allyl-1-methyl-4-(2,2,2-triphenylethyl)naphthalene (**5a**)

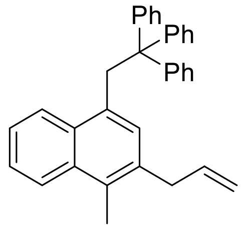

The compound was synthesized using the general procedure for the trapping with Ph_3CBF_4, Method A. The crude compound was purified by column chromatography (SiO_2, pentane:CH_2Cl_2 90:10), giving **5a** as a white solid (106.6 mg, 81% yield).

^{1}H NMR (400 MHz, $CDCl_3$): δ 2.50 (s, 3H), 3.22 (d, *J*=6.3 Hz, 2H), 4.41 (s, 2H), 4.60-4.77 (m, 1H), 4.80-4.90 (m, 1H), 5.58-5.69 (m, 1H), 6.87 (s, 1H), 7.13-7.27 (m, 16H), 7.37 (t, *J*=8.5 Hz, 1H), 7.52 (d, *J*=8.5 Hz, 1H), 7.96 (d, *J*=8.5 Hz, 1H) ppm.

^{13}C{^{1}H} NMR (101 MHz, $CDCl_3$): δ 14.2, 38.4, 41.6, 57.9, 115.2, 123.7, 124.2, 124.7, 125.9 (3xC), 127.6 (6xC), 129.3, 129.9 (6xC), 130.6, 131.4, 132.0, 132.4, 132.8, 133.6, 136.6, 146.8 (3xC) ppm.

HRMS(ESI+, M/Z): Fragmentation observed. Calcd. for $C_{19}H_{15}$ $[M]^+$ 243.1174, found 243.1174. Calcd. for $C_{15}H_{15}$ $[M]^+$ 195.1174, found 195.1173.

1-Allyl-2-(benzyl)-4-(2,2,2-triphenylethyl)naphthalene (**5c**)

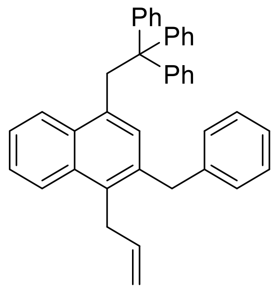

The compound was synthesized using the general procedure for the trapping with Ph_3CBF_4, Method A. The crude compound was purified by column chromatography (SiO_2, pentane:CH_2Cl_2 90:10), giving **5c** as a white solid (115.8 mg, 75% yield).

^{1}H NMR (400 MHz, $CDCl_3$): δ 3.71 (d, *J*=5.4 Hz, 2H), 3.80 (s, 2H), 4.44 (s, 2H), 4.83 (dd, J_1=1.7 Hz, J_2=17.2 Hz, 1H), 4.96 (dd, J_1=1.7 Hz, J_2=10.2 Hz, 1H), 5.85-5.97 (m, 1H), 6.72-6.79 (m, 2H), 7.02 (s, 1H), 7.08-7.24 (m, 19H), 7.32 (t, *J*=7.3 Hz, 1H), 7.51 (d, *J*=8.5 Hz, 1H), 7.90 (d, *J*=8.5 Hz, 1H) ppm.

^{13}C{^{1}H} NMR (101 MHz, $CDCl_3$): δ 32.4, 39.5, 41.4, 57.9, 115.4, 123.7, 124.3, 124.5, 124.8, 125.6, 125.9 (3xC), 127.6 (6xC), 128.2 (2xC), 128.6 (2xC), 129.8 (6xC), 131.1, 132.2, 132.4, 132.8 (2xC), 135.1, 136.4, 140.7, 146.6 (3xC) ppm.

HRMS(ESI+, M/Z): Fragmentation observed. Calcd. for $C_{19}H_{15}$ $[M]^+$ 243.1174, found 243.1175. Calcd. for $C_{21}H_{19}$ $[M]^+$ 271.1487, found 271.1494.

1-Allyl-2-(4-methoxyphenyl)-4-(2,2,2-triphenylethyl)naphthalene (**5e**)

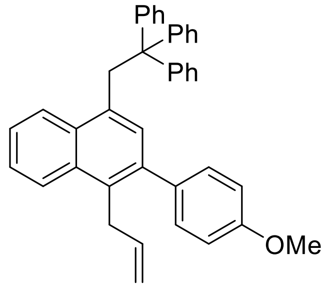

The compound was synthesized using the general procedure for the trapping with Ph_3CBF_4, Method A. The crude compound was purified by column chromatography (SiO_2, pentane:CH_2Cl_2 80:20), giving **5e** as a white solid (113.0 mg, 71% yield).

^{1}**H NMR** (400 MHz, $CDCl_3$): δ 3.65-3.70 (m, 2H), 3.82 (s, 3H), 4.43 (s, 2H), 4.80 (dd, J_1=1.7 Hz, J_2=17.3 Hz, 1H), 5.06 (dd, J_1=1.7 Hz, J_2=10.4 Hz, 1H), 6.04-6.15 (m, 1H), 6.7-6.84 (m, 4H), 7.02 (s, 1H), 7.08-7.20 (m, 16H), 7.34 (t, J=8.8 Hz, 1H), 7.45 (d, J=8.8 Hz, 1H), 7.94 (d, J=8.8 Hz, 1H) ppm.

^{13}**C{^{1}H} NMR** (101 MHz, $CDCl_3$): δ 36.3, 44.2, 57.9, 60.8, 115.7 (2xC), 118.6, 126.4, 127.2, 127.6, 128.1, 128.5 (3xC), 130.3 (6xC), 132.6 (6xC), 132.8, 133.1 (2xC), 134.3, 134.9, 135.3, 135.7, 137.3, 140.5, 140.8, 149.3 (3xC), 161.1 ppm.

HRMS(ESI+, M/Z): Calcd. for $C_{40}H_{35}O$ $[M+H]^+$ 531.2682, found 531.2718.

1-allyl-4-(2,2,2-triphenylethyl)naphthalene (**5f**)

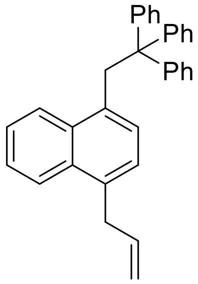

The compound was synthesized using the general procedure for the trapping with Ph_3CBF_4, Method A. The crude compound was purified by column chromatography (SiO_2, pentane:CH_2Cl_2 95:5) giving **5f** as a white solid (101.9 mg, 80% yield).

^{1}**H NMR** (400 MHz, $CDCl_3$): δ 3.73 (d, J=6.2 Hz, 2H), 4.45 (s, 2H), 4.97-5.08 (m, 2H), 6.00-6.11 (m, 1H), 6.95 (d, J=7.4 Hz, 1H), 7.01 (d, J=7.4 Hz, 1H), 7.10-7.18 (m, 10H), 7.20-7.25 (m, 6H), 7.30-7.36 (m, 1H), 7.49 (d, J=8.7 Hz, 1H), 7.92 (d, J=8.5 Hz, 1H) ppm.

^{13}**C{^{1}H} NMR** (101 MHz, $CDCl_3$): δ 39.9, 44.0, 60.7, 118.6, 126.6, 126.9, 127.2, 127.5, 127.9, 128.6 (3xC), 130.2 (6xC), 130.8, 132.5 (6xC), 134.3, 135.9, 136.5, 136.8, 139.8, 149.3 (3xC) ppm.

HRMS(ESI+, M/Z): Fragmentation observed. Calcd. for $C_{19}H_{15}$ $[M]^+$ 243.1174, found 243.1176 Calcd. for $C_{14}H_{13}$ $[M]^+$ 181.1017, found 181.1019.

1-Allyl-2-(*p*-tolyl)-4-(2,2,2-triphenylethyl)naphthalene (**5l**)

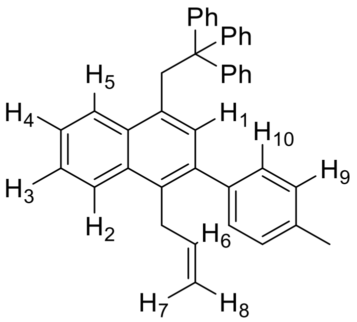

The compound was synthesized using the general procedure for the trapping with Ph_3CBF_4, Method A. The crude compound was purified by column chromatography (SiO_2, pentane:CH_2Cl_2 90:10), giving **5l** as a white solid (97.3 mg, 63% yield).

^{1}H NMR (400 MHz, $CDCl_3$): δ 2.37 (s, 3H, CH_3), 3.66-3.72 (m, 2H, CH_2 allylic), 4.45 (s, 2H, CH_2 benzylic), 4.79-4.86 (m, 1H, H_7), 5.05-5.10 (m, 1H, H_8), 6.05-6.16 (m, 1H, H_6), 6.80 (d, *J*= 8.0 Hz, 2H, H_{10}), 7.05 (s, 1H, H_1), 7.08 (d, *J*= 8.0 Hz, 2H, H_9), 7.11-7.24 (m, 16H), 7.33-7.38 (m, 1H, H_3), 7.48 (d, *J*= 8.6, 1H, H_5), 7.97 (d, *J*= 8.6, 1H, H_2) ppm.

^{13}C{^{1}H} NMR (101 MHz, $CDCl_3$): δ 21.1, 33.7, 41.6, 58.1, 115.9, 123.8, 124.6, 124.9, 125.5, 125.9 (3xC), 127.6 (6xC), 128.3 (2xC), 129.3 (2xC), 130.0 (x 6C), 130.1, 131.6, 132.2, 132.7, 133.1, 136.2, 138.2 (2xC), 139.2, 146.6 (3xC) ppm.

HRMS(ESI+, M/Z): Calcd. for $C_{40}H_{35}$ $[M+H]^+$ 515.2694, found 515.2733.

(*E*)-2-allyl-1-(4,4,4-triphenylbut-1-en-1-yl)naphthalene (**5m**)

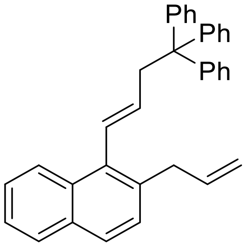

The compound was synthesized using the general procedure for the trapping with Ph_3CBF_4, Method A. The crude compound was purified by column chromatography (SiO_2, pentane:CH_2Cl_2 95:5), giving **5m** as a white solid (110.8 mg, 82% yield).

^{1}H NMR (400 MHz, $CDCl_3$): δ 3.36 (d, *J*=6.0 Hz, 2H), 3.78 (d, *J*=6.7 Hz, 2H), 4.89 (d, *J*=17.1 Hz, 1H), 4.99 (d, *J*=10.1 Hz, 1H), 5.71-5.90 (m, 2H), 6.70 (d, *J*=16.3 Hz, 1H), 7.19-7.34 (m, 17H), 7.34-7.40 (m, 1H), 7.54 (d, *J*=8.5 Hz, 1H), 7.64 (d, *J*=8.5 Hz, 1H), 7.73 (d, *J*=8.1 Hz, 1H) ppm.

^{13}C{^{1}H} NMR (101 MHz, $CDCl_3$): δ 40.7, 48.0, 59.2, 118.1, 127.6, 128.2, 128.5, 128.7 (3xC), 129.5, 130.5 (2xC), 130.6 (6xC), 131.5, 132.1 (6xC), 134.9 (2xC), 137.0 (2xC), 140.1, 149.9 (3xC) ppm.*

*Missing peak carbon due to overlapping signals.

HRMS(ESI+, M/Z): Calcd. for $C_{35}H_{31}$ $[M+H]^+$ 451.2423, found 451.2420.

(2-(4-Allylphenyl)ethane-1,1,1-triyl)tribenzene (**5n**)

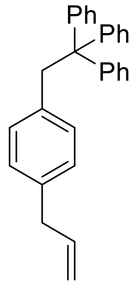

The compound was synthesized using the general procedure for the trapping with Ph_3CBF_4, Method B. The crude compound was purified by column chromatography (SiO_2, pentane:CH_2Cl_2 95:5), giving **5n** as a white solid (78.6 mg, 73% yield).

^{1}H NMR (400 MHz, $CDCl_3$): δ 3.25 (d, *J*=6.5 Hz, 2H), 3.91 (s, 2H), 4.95-5.03 (m, 2H), 5.84-5.95 (m, 1H), 6.55 (d, *J*=8.1 Hz, 2H), 6.80 (d, *J*=8.1 Hz, 2H), 7.14-7.23 (m, 15H) ppm.

^{13}C{^{1}H} NMR (101 MHz, $CDCl_3$): δ 42.3, 48.5, 61.1, 118.1, 128.5 (3xC), 130.1 (x 6C), 130.2 (2xC) 132.4 (6xC), 133.8 (2xC), 138.8, 140.2, 140.3, 149.3 (3xC) ppm.

HRMS(ESI+, M/Z): Fragmentation observed. Calcd. for $C_{19}H_{15}$ $[M]^+$ 243.1174, found 243.1175 Calcd. for $C_{10}H_{11}$ $[M]^+$ 131.0861, found 131.0857.

(2-(3-Allyl-4-methylphenyl)ethane-1,1,1-triyl)tribenzene (**5oa**)

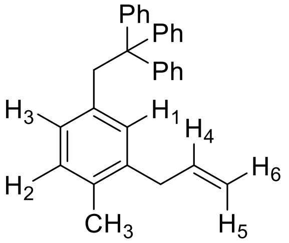

The compound was synthesized using the general procedure for the trapping with Ph_3CBF_4, Method B. The crude compound was purified by column chromatography (SiO_2, pentane), giving **5oa** as a white solid (47.8 mg, 41% yield).

^{1}H NMR (400 MHz, $CDCl_3$): δ 2.17 (s, 3H, CH_3), 3.10 (d, *J*=6.3 Hz, 2H, allylic), 3.90 (s, 2H, benzylic), 4.81- 4.87 (m, 1H, H_5), 4.93-4.98 (m, 1H, H_6), 5.64-5.75 (m, 1H, H_4), 6.33 (s, 1H, H_1), 6.50 (d, *J*= 7.6 Hz, 1H, H_3), 6.80 (d, *J*= 7.6 Hz, 1H, H_2), 7.15-7.24 (m, 15H) ppm.

^{13}C{^{1}H} NMR (101 MHz, $CDCl_3$): δ 18.8, 37.5, 46.0, 58.4, 115.3, 125.8 (3xC), 127.5 (6xC), 128.9, 129.1, 129.9 (6xC), 132.4, 133.8, 135.9, 136.5, 136.8, 146.7 (3xC) ppm.

HRMS(ESI+, M/Z): Fragmentation observed. Calcd. for $C_{19}H_{15}$ $[M]^+$ 243.1174, found 243.1170 Calcd. for $C_{11}H_{13}$ $[M]^+$ 145.1174, found 145.1012.

(2-(2-Allyl-4-methylphenyl)ethane-1,1,1-triyl)tribenzene (**5ob**)

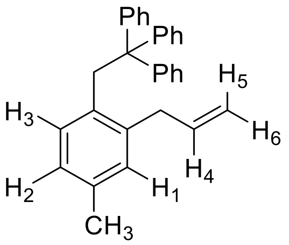

The compound was synthesized using the general procedure for the trapping with Ph_3CBF_4, Method B. The crude compound was purified by column chromatography (SiO_2, pentane), giving **5ob** as a white solid (32.6 mg, 28% yield).

^{1}H NMR (400 MHz, $CDCl_3$): δ 2.22 (s, 3H, CH_3), 2.46 (d, *J*=6.3 Hz, 2H, allylic), 3.92 (s, 2H, benzylic), 4.75- 4.82 (m, 1H, H_5), 4.94-4.99 (m, 1H, H_6), 5.84-5.95 (m, 1H, H_4), 6.65-6.69 (m, 1H, H_2), 6.74-6.79 (m, 2H, H_1+H_3), 7.13-7.23 (m, 15H) ppm.

^{13}C{^{1}H} NMR (101 MHz, $CDCl_3$): δ 20.9, 36.6, 40.8, 58.2, 115.3, 125.9 (3xC), 126.2, 127.5 (6xC), 129.7, 129.9 (6xC), 130.4, 133.8, 135.6, 137.4, 140.0, 146.6 (3xC) ppm.

HRMS(ESI+, M/Z): Fragmentation observed. Calcd. for $C_{19}H_{15}$ $[M]^+$ 243.1174, found 243.1169 Calcd. for $C_{11}H_{13}$ $[M]^+$ 145.1174, found 145.1010.

1-allyl-4-(1-(cyclohepta-2,4,6-trien-1-yl)but-3-en-1-yl)naphthalene (**7a**)

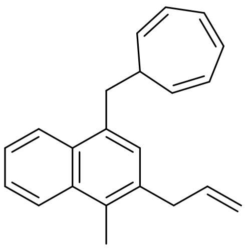

The compound was synthesized using the general procedure for the trapping with tropylium tetrafluoroborate, Method C. The crude compound was purified by column chromatography (SiO_2, pentane), giving **7a** as a white solid (64.4 mg, 75% yield).

^{1}H NMR (400 MHz, $CDCl_3$): δ 2.16-2.26 (m, 1H), 2.61 (s, 3H), 3.45 (d, J=8.5 Hz, 2H), 3.60 (dt, J_1=1.7 Hz, J_2=6.2 Hz, 2H), 4.96-5.03 (m, 1H), 5.06-5.11 (m, 1H), 5.36 (dd, J_1= 4.5 Hz, J_2= 9.3 Hz, 2H), 5.98-6.10 (m, 1H), 6.15-6.22 (m, 2H), 6.60-6.68 (m, 2H), 7.21 (s, 1H), 7.43-7.54 (m, 2H), 7.98-8.02 (m, 1H), 8.07-8.11 (m, 1H) ppm.

^{13}C{^{1}H} NMR (101 MHz, $CDCl_3$): δ 14.3, 36.2, 38.6, 39.2, 115.4, 124.0, 124.7, 124.8 (3xC), 125.4, 126.4 (2xC), 129.6, 130.1, 130.9 (2xC), 131.1, 133.5, 133.6, 134.1, 136.9 ppm.

HRMS(ESI+, M/Z): Fragmentation observed. Calcd. for C_7H_7 $[M]^+$ 91.0548, found 91.0540. Calcd. for $C_{15}H_{15}$ $[M]^+$ 195.1174, found 195.1167.

1-allyl-4-(1'-(cyclohepta-2,4,6-trien-1-yl)ethyl)-2-(4'-methoxyphenyl)-naphthalene (**7e**)

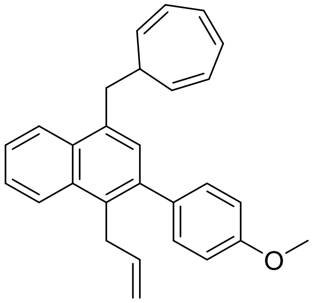

The compound was synthesized using the general procedure for the trapping with tropylium tetrafluoroborate, Method C. The crude compound was purified by column chromatography (SiO_2, pentane:CH_2Cl_2 95:5), giving **7e** as a white solid (70.4 mg, 62% yield).

^{1}H NMR (400 MHz, $CDCl_3$): δ 2.21-2.30 (m, 1H), 3.48 (d, *J*=7.8 Hz, 2H), 3.74-3.80 (m, 2H), 3.89 (s, 3H), 4.85-4.92 (m, 1H), 5.06-5.12 (m, 1H), 5.39 (dd, J_1= 5.5 Hz, J_2= 9.2 Hz, 2H), 6.07-6.24 (m, 3H), 6.59-6.67 (m, 2H), 7.00 (d, *J*= 8.5 Hz, 2H), 7.34 (s, 1H), 7.38 (d, *J*= 8.5 Hz, 2H), 7.46-7.55 (m, 2H), 8.02-8.12 (m, 2H) ppm.

^{13}C{^{1}H} NMR (101 MHz, $CDCl_3$): δ 33.9, 36.3, 39.2, 55.3, 113.4 (2xC), 116.0, 124.1, 124.9 (2xC), 125.3, 125.8, 126.0, 126.3 (2xC), 129.7, 130.4 (2xC), 130.9 (2xC), 131.0, 131.7, 132.9, 134.2, 134.9, 138.0, 138.8, 158.7 ppm.

HRMS(ESI+, M/Z): Calcd. for $C_{28}H_{27}O$ $[M+H]^+$ 379.2017, found 379.2056.

1-Allyl-4-(cyclohepta-2,4,6-trien-1-ylmethyl)naphthalene (**7f**)

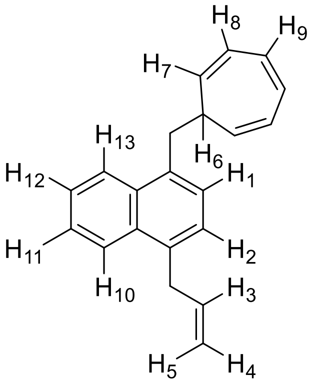

The compound was synthesized using the general procedure for the trapping with tropylium tetrafluoroborate, Method C. The crude compound was purified by column chromatography (SiO_2, pentane) using pentane as eluent, giving **7f** as a white solid (73.5 mg, 90% yield).

^{1}H NMR (400 MHz, $CDCl_3$): δ 2.17-2.25 (m, 1H, H_6), 3.46 (d, *J*= 7.8 Hz, 2H, CH_2 benzylic), 3.83 (d, *J*= 6.3 Hz, 2H, CH_2 allylic), 5.07-5.14 (m, 2H, H_4+H_5), 5.36 (dd, J_1= 5.4 Hz, J_2= 9.0 Hz, 2H, H_7), 6.06-6.23 (m, 3H, H_3+H_8), 6.60-6.67 (m, 2H, H_9), 7.27-7.37 (m, 2H, H_1+H_2), 7.46-7.54 (m, 2H, H_{11}+H_{12}), 8.01-8.08 (m, 2H, H_{10}+H_{13}) ppm.

$^{13}C\{^1H\}$ NMR (101 MHz, $CDCl_3$): δ 36.2, 37.3, 39.1, 116.1, 124.2, 124.8 (2xC), 124.9, 125.4, 125.5, 125.8, 126.3, 126.5 (2xC), 131.0 (2xC), 132.3, 132.4, 134.6, 134.8, 137.1 ppm.

HRMS(ESI+, M/Z): Fragmentation observed. Calcd. for $C_{14}H_{13}$ $[M]^+$ 181.1017, found 181.1016 Calcd. for C_7H_7 $[M]^+$ 91.0548, found 91.0541.

1-Allyl-4-(cyclohepta-2,4,6-trien-1-yl(phenyl)methyl)naphthalene (**7i**)

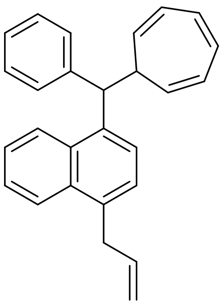

The compound was synthesized using the general procedure for the trapping with tropylium tetrafluoroborate, Method C. The crude compound was purified by column chromatography (SiO_2, pentane), giving **7i** as a white solid (54.4, 52% yield).

^1H NMR (400 MHz, $CDCl_3$): δ 2.50-2.58 (m, 1H), 3.82 (d, *J*= 6.4 Hz, 2H), 5.03-5.17 (m, 4H), 5.39-5.46 (m, 1H), 6.06-6.21 (m, 3H), 6.69-6.77 (m, 2H), 7.14 (t, *J*= 7.4 Hz, 1H), 7.21-7.27 (m, 2H), 7.32-7.36 (m, 4H), 7.45-7.54 (m, 2H), 8.03-8.08 (m, 1H), 8.31-8.36 (m, 1H) ppm.

$^{13}C\{^1H\}$ NMR (101 MHz, $CDCl_3$): δ 37.4, 44.0, 48.4, 116.3, 124.2, 124.3, 124.7, 124.9, 125.2, 125.3, 125.7, 125.8, 126.0, 126.3, 128.4 (2xC), 128.6 (2xC), 130.9, 131.0, 132.6, 132.7, 134.9, 137.0, 137.8, 143.6 ppm.

HRMS(ESI+, M/Z): Fragmentation observed. Calcd. for C_7H_7 $[M]^+$ 91.0548, found 91.0542. Calcd. for $C_{20}H_{17}$ $[M]^+$ 257.1330, found 257.1327.

1-Allyl-4-(1-(cyclohepta-2,4,6-trien-1-yl)but-3-en-1-yl)naphthalene (**7j**)

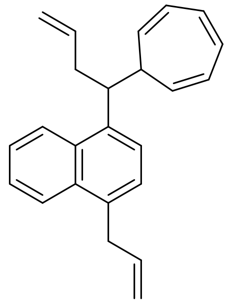

The compound was synthesized using the general procedure for the trapping with tropylium tetrafluoroborate, Method C. The crude compound was purified by column chromatography (SiO_2, pentane), giving **7j** as a white solid (56.2 mg, 60% yield).

^{1}H NMR (400 MHz, $CDCl_3$): δ 2.03-2.13 (m, 1H), 2.57-2.68 (m, 1H), 2.78-2.87 (m, 1H), 3.85 (d, *J*=6.6 Hz, 2H), 3.94-4.05 (m, 1H), 4.78-4.84 (m, 1H), 4.89-4.98 (m, 2H), 5.08-5.16 (m, 2H), 5.47-5.58 (m, 2H), 5.98 (dd, J_1= 5.6 Hz, J_2= 9.4 Hz, 1H), 6.09-6.20 (m, 1H), 6.32 (dd, J_1= 5.3 Hz, J_2= 9.4 Hz, 1H), 6.64-6.76 (m, 2H), 7.19 (d, *J*=7.5 Hz, 1H), 7.33 (d, *J*=7.5 Hz, 1H), 7.50-7.57 (m, 2H), 8.07-8.12 (m, 1H), 8.18-8.25 (m, 1H) ppm.

^{13}C{^{1}H} NMR (101 MHz, $CDCl_3$): δ 37.4, 39.0, 40.6 (broad), 43.9, 116.2, 116.3, 123.9, 124.2, 124.9, 125.0 (2xC), 125.2, 125.4, 125.5, 126.0, 130.6, 130.9, 132.3, 133.2, 134.3, 135.8, 137.1, 138.2 ppm.*

*Missing peak carbon due to overlapping signals.

HRMS(ESI+, M/Z): Calcd. for $C_{24}H_{25}$ $[M+H]^+$ 313.1950, found 313.1938.

(*E*)-2-allyl-1-(3-(cyclohepta-2,4,6-trien-1-yl)prop-1-en-1-yl)naphthalene (**7m**)

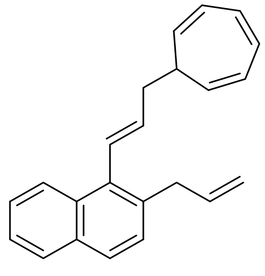

The compound was synthesized using the general procedure for the trapping with tropylium tetrafluoroborate, Method C. The crude compound was purified by column chromatography (SiO_2, pentane), giving **7m** as a white solid (67.1 mg, 75% yield).

^{1}H NMR (400 MHz, $CDCl_3$): δ 1.88-1.96 (m, 1H), 2.76-2.82 (m, 2H), 3.61 (d, *J*= 6.2 Hz, 2H), 4.96-5.03 (m, 1H), 5.04-5.09 (m, 1H), 5.37 (dd, J_1= 5.5 Hz, J_2= 9.0 Hz, 2H), 5.87-6.07 (m, 2H), 6.23-6.30 (m, 2H), 6.67-6.74 (m, 2H), 6.82 (d, *J*= 16.1 Hz, 1H), 7.35 (d, *J*= 8.5 Hz, 1H), 7.41-7.50 (m, 2H), 7.72 (d, *J*= 8.5 Hz, 1H), 7.78-7.84 (m, 1H), 8.10-8.15 (m, 1H) ppm.

$^{13}C\{^{1}H\}$ NMR (101 MHz, $CDCl_3$): δ 36.9, 38.3, 38.6, 115.6, 124.9 (2xC), 125.0, 125.7 (2xC), 126.1 (2xC), 126.9, 127.8, 128.0 (2xC), 131.0 (2xC), 132.4, 134.5 (2xC), 135.0 (2xC), 137.5 ppm.

HRMS(ESI+, M/Z): Calcd. for $C_{23}H_{23}$ $[M+H]^+$ 299.1755, found 299.1793.

7-(4-allylbenzyl)cyclohepta-1,3,5-triene (**7n**)

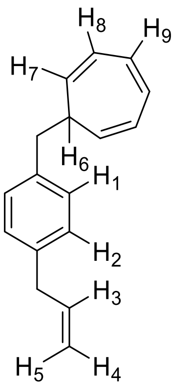

The compound was synthesized using the general procedure for the trapping with tropylium tetrafluoroborate, Method D. The crude compound was purified by column chromatography (SiO_2, pentane), giving **7n** as a white solid (42.7 mg, 64% yield).

^{1}H NMR (400 MHz, $CDCl_3$): δ 1.95-2.04 (m, 1H, H_6), 3.01 (d, *J*= 8.0 Hz, 2H, CH_2 benzylic), 3.37 (d, *J*= 6.8 Hz, 2H, CH_2 allylic), 5.04-5.12 (m, 2H, H_4+H_5), 5.27 (dd, J_1= 5.5 Hz, J_2= 9.2 Hz, 2H, H_7), 5.92-6.04 (m, 1H, H_3), 6.13-6.22 (m, 2H, H_8) 6.62-6.69 (m, 2H, H_9), 7.10-7.17 (m, 4H, H_1+H_2) ppm.

$^{13}C\{^{1}H\}$ NMR (101 MHz, $CDCl_3$): δ 38.6, 39.9, 40.1, 115.7, 124.9 (2xC), 126.2 (2xC), 128.5 (2xC), 129.0 (2xC), 130.9 (2xC), 137.6, 137.8 (2xC) ppm.

HRMS(ESI+, M/Z): Fragmentation observed. Calcd. for C_7H_7 $[M]^+$ 91.0548, found 91.0541.

7-(3-allyl-4-Methyl-benzyl)cyclohepta-1,3,5-triene (7oa) + 7-(2-allyl-4-Methyl-benzyl)cyclohepta-1,3,5-triene (7ob)

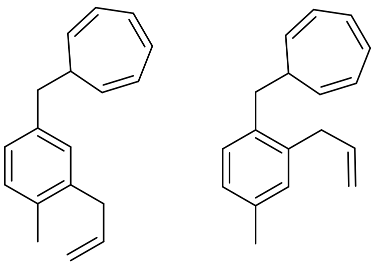

The compound was synthesized using the general procedure for the trapping with tropylium tetrafluoroborate, Method D. The crude compound was purified by column chromatography (SiO_2, pentane), giving a mixture of **7oa** and **7ob** as a white solid (43.9 mg, 62% yield – 6:4 regioisomer ratio).

^{1}H NMR (400 MHz, $CDCl_3$): δ 1.95-2.08 (m, $1H_{major} + 1H_{minor}$), 2.27 (s, $3H_{major}$), 2.32(s, $3H_{minor}$), 2.97-3.04 (m, $2H_{major} + 2H_{minor}$), 3.34-3.40 (m, $2H_{major} + 2H_{minor}$), 4.97-5.10 (m, $2H_{major} + 2H_{minor}$), 5.24-5.31 (m, $2H_{major} + 2H_{minor}$), 5.90-6.02 (m, $1H_{major} + 1H_{minor}$), 6.15-6.22 (m, $2H_{major} + 2H_{minor}$), 6.63-6.70 (m, $2H_{major} + 2H_{minor}$), 9.97-7.12 (m, $3H_{major} + 3H_{minor}$) ppm.

^{13}C{^{1}H} NMR (101 MHz, $CDCl_3$): δ 18.9, 21.0, 35.3, 37.1, 37.7, 38.7, 39.1, 40.1, 115.6, 115.7, 124.8, 124.9, 126.3 (2xC), 126.7, 127.0, 128.3, 128.8, 129.0, 129.1, 129.4, 129.9 (2xC), 130.1 (2xC), 130.4, 130.9 (2xC), 134.0, 134.9, 135.8, 136.7, 137.2, 137.7, 137.9, 138.0 ppm.

HRMS(ESI+, M/Z): Calcd. for $C_{18}H_{21}$ $[M+H]^+$ 237.1638, found 237.1646.

2-((3-allyl-4-methylnaphthalen-1-yl)methyl)benzo[d][1,3]dithiole (8aa) and **2-((2-allyl-4-methylnaphthalen-1-yl)methyl)benzo[d][1,3]dithiole (8ab)**

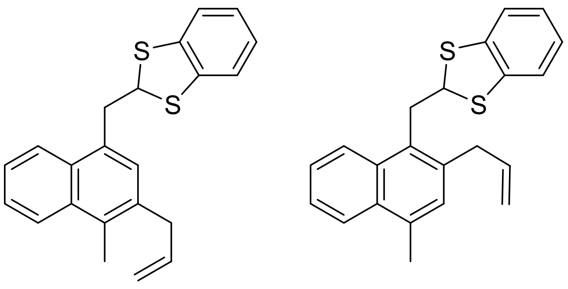

The compound was synthesized using the general procedure for the trapping with 1,3-Benzo dithiolylium tetrafluoroborate, Method E. The crude compound was purified by column chromatography (SiO_2, pentane:CH_2Cl_2 from 90:10 to 85:15), giving a mixture of **8aa** and **8ab** as a colorless sticky oil (92.0 mg, 88% yield – 85:15 regioisomer ratio).

^{1}H NMR (400 MHz, $CDCl_3$): δ 2.63 (s, 3H major), 2.69 (s, 3H minor), 3.60-3.69 (m, 4H major + 2H minor), 3.75 (d, *J*= 7.6 Hz, 2H minor), 4.87-4.98 (m, 1H minor), 4.99-5.14 (m, 2H major + 1H minor), 5.21-5.30 (m, 1H major + 1H minor), 5.92-6.11 (m, 1H major + 1H minor), 7.04-7.13 (m, 2H major + 2H minor), 7.21-7.33 (m, 3H major + 3H minor), 7.48-7.58 (m, 2H major + 2H minor), 7.93-8.01 (m, 1H major + 1H minor), 8.02-8.05 (m, 1H minor), 8.09-8.15 (m, 1H major) ppm.

^{13}C{^{1}H} NMR (101 MHz, $CDCl_3$): δ 17.1 (major), 22.2 (minor), 39.3 (minor), 40.9 (minor), 41.2 (major), 44.6 (major), 57.7 (major), 58.1 (minor), 118.3 (major), 118.6 (minor), 125.3 (major), 125.5 (minor), 126.3 (major), 126.9 (minor), 127.6 (minor), 127.7 (major + minor), 127.8 (major), 128.2 (major), 128.3 (major), 128.4 (minor), 128.6 (minor), 131.3 (minor), 132.0 (minor), 133.3 (major), 133.5 (major), 133.7 (major), 134.0 (major), 134.7 (minor), 135.0 (minor), 136.2 (major), 136.6 (minor), 136.9 (major), 139.2 (minor), 139.4 (major), 139.8 (minor), 140.0 (major), 140.2 (minor) ppm.*

*Presence of regioisomer prevents accurate assignation of symmetric carbon.

HRMS(ESI+, M/Z)**:** Calcd. for $C_{22}H_{20}S_2$ $[M+H]^+$ 349.1079, found: 349.1070.

2-((3-allyl-4-ethylnaphthalen-1-yl)methyl)benzo[d][1,3]dithiole (**8ba**) and **2-((2-allyl-4-ethylnaphthalen-1-yl)methyl)benzo[d][1,3]dithiole** (**8bb**)

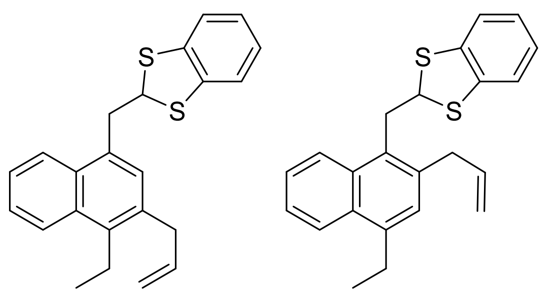

The compound was synthesized using the general procedure for the trapping with 1,3-Benzo dithiolylium tetrafluoroborate, Method E. The crude compound was purified by column

chromatography (SiO_2, pentane:CH_2Cl_2 90:10), giving a mixture of **8ba** and **8bb** as a colorless sticky oil (83.7 mg, 72% yield – 82:18 regioisomer ratio).

^{1}H NMR (400 MHz, $CDCl_3$): δ 1.32 (t, *J*= 7.5 Hz, 2H major), 1.14 (t, *J*= 7.5 Hz, 2H minor), 3.08-3.17 (m, 2H major + 2H minor), 3.60-3.70 (m, 4H major + 2H minor), 3.75 (d, *J*= 7.6 Hz, 2H minor), 4.87-4.94 (m, 1H minor), 5.03-5.15 (m, 2H major + 1H minor), 5.21-5.30 (m, 1H major + 1H minor), 5.93-6.03 (m, 1H minor), 6.04-6.14 (m, 1H major), 7.05-7.14 (m, 2H major + 2H minor), 7.23-7.33 (m, 3H major + 3H minor),7.49-7.58 (m, 2H major + 2H minor), 7.94-8.02 (m, 1H major + 1H minor), 8.08-8.12 (m, 1H minor), 8.13-8.17 (m, 1H major) ppm.

^{13}C{^{1}H} NMR (101 MHz, $CDCl_3$): δ 17.7 (minor), 17.9 (major), 24.1 (major), 28.6 (minor), 39.4 (minor), 40.4 (major), 41.1 (minor), 44.7 (major), 57.6 (major), 58.1 (minor), 118.4 (major), 118.6 (minor), 125.3 (major), 125.5, 126.5 (major), 127.0 (minor), 127.3 (minor), 127.5 (minor), 127.6 (major), 127.8 (major), 128.2 (major), 128.3 (major), 128.4, 128.5 (minor), 130.3 (minor), 131.3 (minor), 133.7 (major + minor), 133.8 (major + minor), 135.2 (major), 135.3 (minor), 136.3 (major + minor), 139.8 (minor), 140.0 (major), 140.1 (major), 140.2 (minor), 142.5 (minor) ppm.*

*Presence of regioisomer prevents accurate assignation of symmetric carbon.

HRMS(ESI+, M/Z): Calcd. for $C_{23}H_{23}S_2$ $[M+H]^+$ 363.1235, found: 363.1237.

2-((4-allyl-3-(4-methoxyphenyl)naphthalen-1-yl)methyl)benzo[d][1,3]dithiole (**8e**)

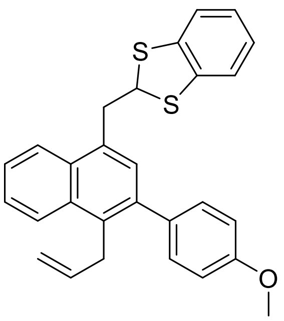

The compound was synthesized using the general procedure for the trapping with 1,3-Benzo dithiolylium tetrafluoroborate, Method E. The crude compound was purified by column chromatography (SiO_2, pentane:CH_2Cl_2 from 90:10 to 85:15), giving **8e** as a white solid (101.8 mg, 77% yield).

^{1}H NMR (400 MHz, $CDCl_3$): δ 3.68 (d, *J*= 7.4 Hz, 2H), 3.78-3.82 (m, 2H), 3.90 (s, 3H), 4.86-4.94 (m, 1H), 5.09-5.15 (m, 1H), 5.26 (t, *J*= 7.4 Hz, 1H), 6.09-6.20 (m, 1H), 6.99-7.10 (m, 4H), 7.24-7.30 (m, 2H), 7.36-7.43 (m, 3H), 7.52-7.60 (m, 2H), 7.99-8.04 (m, 1H), 8.11-8.17 (m, 1H) ppm.

^{13}C{^{1}H} NMR (101 MHz, $CDCl_3$): δ 33.9, 42.1, 54.9, 55.3, 113.5 (2xC), 116.2, 122.7 (2xC), 123.7, 125.6 (2xC), 125.7, 126.0, 126.2, 130.5 (2xC), 131.0, 131.2, 131.5, 132.1, 133.0, 134.6, 137.2 (2xC), 137.8, 138.8, 158.8. ppm.

HRMS(ESI+, M/Z): Calcd. for $C_{28}H_{23}OS_2$ $[M-H]^+$ 439.1190, found: 439.1192.

2-((4-allylnaphthalen-1-yl)methyl)benzo[d][1,3]dithiole (**8f**)

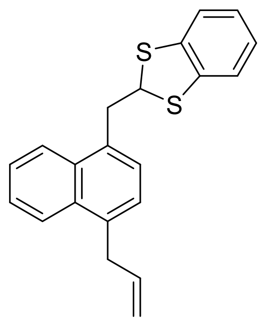

The compound was synthesized using the general procedure for the trapping with 1,3-Benzo dithiolylium tetrafluoroborate, Method E. The crude compound was purified by column chromatography (SiO_2, pentane:CH_2Cl_2 90:10), giving **8f** as a colorless sticky oil (72.2 mg, 72% yield).

^{1}H NMR (400 MHz, $CDCl_3$): δ 3.68 (d, *J*= 7.4 Hz, 2H), 3.86 (d, *J*= 6.3 Hz, 2H), 5.09-5.18 (m, 2H), 5.23 (t, *J*= 7.4 Hz, 1H), 6.08-6.20 (m, 1H), 7.05-7.11 (m, 2H), 7.25-7.31 (m, 2H), 7.32-7.38 (m, 2H), 7.52-7.59 (m, 2H), 7.96-8.03 (m, 1H), 8.08-8.15 (m, 1H) ppm.

^{13}C{^{1}H} NMR (101 MHz, $CDCl_3$): δ 37.4, 42.2, 54.9, 116.4, 122.7 (2xC), 123.9, 125.1, 125.6 (3xC), 125.8, 125.9, 128.1, 131.9, 132.0, 132.4, 136.0, 136.9, 137.2 (2xC) ppm.

HRMS(ESI+, M/Z): Calcd. for $C_{21}H_{17}S_2$ $[M-H]^+$ 333.0850, found: 333.0771.

2-((4-allyl-2-methylnaphthalen-1-yl)methyl)benzo[d][1,3]dithiole (**8g**)

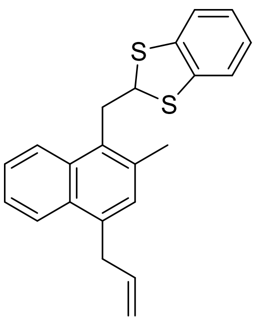

The compound was synthesized using the general procedure for the trapping with 1,3-Benzo dithiolylium tetrafluoroborate, Method E. The crude compound was purified by column chromatography (SiO_2, pentane:CH_2Cl_2 90:10), giving **8g** as a white solid (81.5 mg, 78% yield).

^{1}H NMR (400 MHz, $CDCl_3$): δ 3.50 (s, 3H), 3.74 (d, *J*= 7.7 Hz, 2H), 3.82 (d, *J*= 6.3 Hz, 2H), 5.10-5.17 (m, 2H), 5.36 (t, *J*= 7.7 Hz, 1H), 6.07-6.19 (m, 1H), 7.06-7.12 (m, 2H), 7.23 (s, 1H) 7.27-7.33 (m, 2H), 7.44-7.55 (m, 2H), 7.96 (d, *J*= 8.4 Hz, 1H), 8.05 (d, *J*= 8.4 Hz, 1H) ppm.

^{13}C{^{1}H} NMR (101 MHz, $CDCl_3$): δ 23.7, 39.9, 40.0, 57.9, 118.9, 125.4 (2xC), 126.7, 127.3, 127.5, 128.4 (2xC), 128.6, 131.8, 132.5, 133.7, 135.2, 137.8, 138.0, 139.6, 140.2 ppm.*

*Missing peak carbon due to overlapping signals.

HRMS(ESI+, M/Z): Calcd. for $C_{22}H_{19}S_2$ $[M-H]^+$ 347.0928, found: 347.0926.

2-((4-allyl-6-methoxynaphthalen-1-yl)methyl)benzo[d][1,3]dithiole (**8h**)

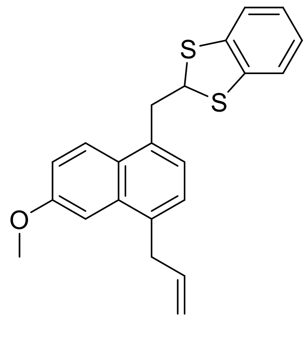

The compound was synthesized using the general procedure for the trapping with 1,3-Benzo dithiolylium tetrafluoroborate, Method E. The crude compound was purified by column chromatography (SiO_2, pentane:CH_2Cl_2 from 90:10 to 85:15), giving **8h** as a white solid (67.8 mg, 62% yield).

^{1}H NMR (400 MHz, $CDCl_3$): δ 3.63 (d, *J*= 7.4 Hz, 2H), 3.80 (d, *J*= 6.3 Hz, 2H), 3.94 (s, 3H), 5.12-5.22 (m, 3H), 6.06-6.18 (m, 1H), 7.04-7.11 (m, 2H), 7.19-7.33 (m, 5H), 7.36 (d, *J*= 2.5 Hz, 1H), 7.90 (d, *J*= 9.3 Hz, 1H) ppm.

^{13}C{^{1}H} NMR (101 MHz, $CDCl_3$): δ 37.8, 42.1, 55.1, 55.3, 103.9, 116.3, 118.0, 122.7 (2xC), 125.5, 125.6 (2xC), 125.7, 126.4, 127.3, 132.0, 133.7, 134.6, 136.7, 137.2 (2xC), 157.3 ppm.

HRMS(ESI+, M/Z): Calcd. for $C_{22}H_{19}OS_2$ $[M-H]^+$ 363.0877, found: 363.0878.

2-((4-allylnaphthalen-1-yl)(phenyl)methyl)benzo[d][1,3]dithiole (**8i**)

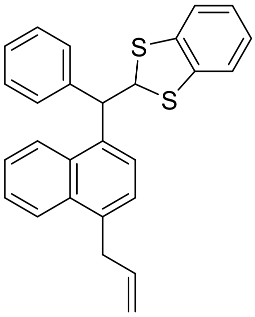

The compound was synthesized using the general procedure for the trapping with 1,3-Benzo dithiolylium tetrafluoroborate, Method E. The crude compound was purified by column chromatography (SiO_2, pentane:CH_2Cl_2 from 90:10 to 85:15), giving **8i** as a white solid (55.4 mg, 45% yield).

^{1}H NMR (400 MHz, $CDCl_3$): δ 3.85 (d, *J*= 6.3 Hz, 2H), 5.11-5.18 (m, 2H), 5.38 (d, *J*= 11.1 Hz, 1H), 6.05 (d, *J*= 11.1 Hz, 1H), 6.08-6.19 (m, 1H), 6.95-7.08 (m, 3H), 7.15-7.24 (m, 2H), 7.27-7.32 (m, 2H), 7.40-7.55 (m, 6H) 8.03-8.09 (m, 1H), 8.14-8.19 (m, 1H) ppm.

^{13}C{^{1}H} NMR (101 MHz, $CDCl_3$): δ 37.4, 53.2, 59.7, 116.5, 122.2 (2xC), 123.6, 124.2, 124.8, 125.4, 125.5, 125.6, 125.7, 126.0, 127.3, 128.4 (2xC), 128.6 (2xC), 132.2, 132.6, 135.9, 136.4, 136.7, 137.4, 137.9, 141.3 ppm.

HRMS(ESI+, M/Z): Fragmentation observed. Calcd. for $C_7H_5S_2$ $[M]^+$ 152.9833, found: 152.9827. Calcd. for $C_{20}H_{17}$ $[M]^+$ 257.1330, found: 257.1324.

2-(1-(4-allylnaphthalen-1-yl)ethyl)benzo[d][1,3]dithiole (**8k**)

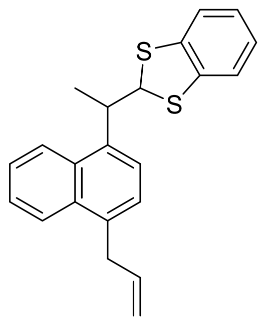

The compound was synthesized using the general procedure for the trapping with 1,3-Benzo dithiolylium tetrafluoroborate, Method E. The crude compound was purified by column chromatography (SiO_2, pentane:CH_2Cl_2 90:10), giving **8k** as a white solid (81.5 mg, 78% yield).

^{1}H NMR (400 MHz, $CDCl_3$): δ 1.56 (d, *J*= 7.0 Hz, 3H), 3.80-3.93 (m, 2H), 4.17 (m, 1H), 5.11-5.20 (m, 2H), 5.41 (d, *J*= 7.0 Hz, 1H), 6.10-6.22 (m, 1H), 6.99-7.06 (m, 2H), 7.10-7.15 (m, 1H), 7.22-7.27 (m, 1H), 7.37-7.44 (m, 2H), 7.51-7.58 (m, 2H), 8.05-5.16 (m, 2H) ppm.

^{13}C{^{1}H} NMR (101 MHz, $CDCl_3$): δ 17.7, 37.4, 41.6, 60.4, 116.4, 121.9, 122.0, 123.5, 123.6, 125.1, 125.3, 125.4, 125.6, 125.9 (2xC), 131.8, 132.4, 135.5, 136.9, 137.8 (2xC), 138.1 ppm.

HRMS(ESI+, M/Z): Calcd. for $C_{22}H_{19}S_2$ $[M-H]^+$ 347.0928, found: 347.0919. Fragmentation observed (**HRMS -** ESI)**:** Calcd. for $C_7H_5S_2$ $[M]^+$ 152.9833, found: 152.9827. Calcd. for $C_{15}H_{15}$ $[M]^+$ 195.1174, found: 195.1166.

(E)-2-(3-(2-allylnaphthalen-1-yl)allyl)benzo[d][1,3]dithiole (**8m**)

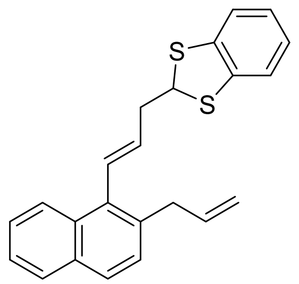

The compound was synthesized using the general procedure for the trapping with 1,3-Benzo dithiolylium tetrafluoroborate, Method E. The crude compound was purified by column chromatography (SiO_2, pentane:CH_2Cl_2 95:5), giving **8m** as a colorless sticky oil (72.5 mg, 67% yield).

^{1}H NMR (400 MHz, $CDCl_3$): δ 2.99 (d, J_1= 1.4 Hz J_2= 7.0 Hz, 2H), 3.61 (d, J= 6.2 Hz, 2H), 4.99-5.13 (m, 3H), 5.80-5.90 (m, 1H), 5.98-6.09 (m, 1H), 6.86 (d, J= 16.0 Hz, 1H), 7.04-7.09 (m, 2H), 7.26-7.31 (m, 2H), 7.37 (d, J= 8.4 Hz, 1H), 7.44-7.52 (m, 2H), 7.74 (d, J= 8.4 Hz, 1H), 7.81-7.85 (m, 1H), 8.08-8.14 (m, 1H) ppm.

^{13}C{^{1}H} NMR (101 MHz, $CDCl_3$): δ 38.3, 43.0, 53.9, 115.8, 122.6 (2xC), 125.1, 125.6 (2xC), 125.7, 126.0, 127.3, 128.0, 128.1, 130.5, 131.9, 132.2, 132.4, 133.9, 134.6, 137.2 (2xC), 137.4 ppm.

HRMS(ESI+, M/Z): Calcd. for $C_{23}H_{21}S_2$ $[M+H]^+$ 361.1075, found: 361.1089.

2-(4-allylbenzyl)benzo[d][1,3]dithiole (**8n**)

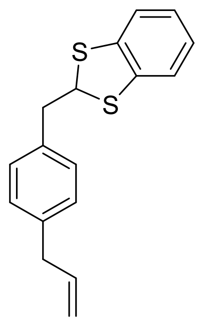

The compound was synthesized using the general procedure for the trapping with 1,3-Benzo dithiolylium tetrafluoroborate, Method F. The crude compound was purified by column chromatography (SiO_2, pentane:CH_2Cl_2 90:10), giving **8n** as a colorless sticky oil (55.5 mg, 65% yield).

^{1}H NMR (400 MHz, $CDCl_3$): δ 3.18 (d, J= 7.5 Hz, 2H), 3.38 (d, J= 6.7 Hz, 2H), 4.99 (t, J= 7.5 Hz, 1H), 5.05-5.11 (m, 2H), 5.91-6.06 (m, 1H), 7.01-7.06 (m, 2H), 7.12-7.18 (m, 4H), 7.20-7.25 (m, 2H) ppm.

^{13}C{^{1}H} NMR (101 MHz, $CDCl_3$): δ 39.9, 44.6, 55.7, 115.9, 122.6 (2xC), 125.5 (2xC), 128.6 (2xC), 129.5 (2xC), 135.2, 137.0, 137.3, 138.9 ppm.*

*missing peak due to overlapping signals, it’s not possible to distinguish which signal belong to the two symmetric quaternary aromatic carbons bonded to Sulphur.

HRMS(ESI+, M/Z): Calcd. for $C_{17}H_{15}S_2$ $[M-H]^+$ 283.0615, found: 283.0605. Fragmentation observed **HRMS**(ESI+, M/Z): Calcd. for $C_7H_5S_2$ $[M]^+$ 152.9833, found: 152.9825. Calcd. for $C_{10}H_{11}$ $[M]^+$ 131.0861, found: 131.0852.

2-((4-allylnaphthalen-1-yl)methyl)-2-methylbenzo[d][1,3]dithiole (**9fa**)

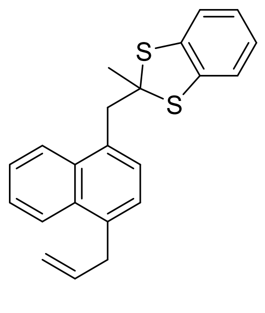

The compound was synthesized using the general procedure for alkylation (using MeI). The crude compound was purified by column chromatography (SiO_2, pentane:CH_2Cl_2 95:5), giving **9fa** as a colorless sticky oil (127.2 mg, 73% yield).

^{1}H NMR (400 MHz, $CDCl_3$): δ 1.87 (s, 3H), 3.86 (d, *J* = 6.3 Hz, 2H), 3.92 (s, 2H), 5.08-5.19 (m, 2H), 6.05-6.25 (m, 1H), 7.02-7.10 (m, 2H), 7.19-7.25 (m, 2H), 7.31-7.41 (m, 1H), 7.47-7.56 (m, 3H), 8.05-8.23 (m, 2H) ppm.

^{13}C{^{1}H} NMR (101 MHz, $CDCl_3$): δ 29.0, 37.4, 43.8, 70.6, 116.3, 122.8 (2xC), 124.7, 125.3, 125.4 (3xC), 125.5, 125.5, 129.3, 131.7, 132.3, 133.1, 136.0, 136.9, 138.4 (2xC) ppm.

HRMS(ESI+, M/Z): Fragmentation observed. Calcd. for $C_8H_7S_2$ $[M]^+$ 166.9989, found: 166.9984. Calcd. for $C_{14}H_{13}$ $[M]^+$ 181.1017, found: 181.1011.

2-((4-allylnaphthalen-1-yl)methyl)-2-hexylbenzo[d][1,3]dithiole (**9fb**)

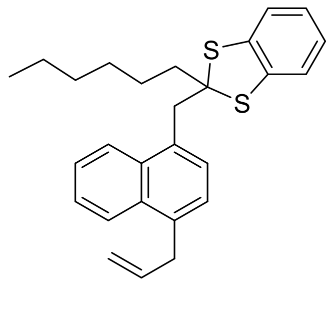

The compound was synthesized using the general procedure for alkylation (using HexylI). The crude compound was purified by column chromatography (SiO_2, pentane:CH_2Cl_2 95:5), giving **9fb** as a colorless sticky oil (175.8 mg, 84% yield).

^{1}H NMR (400 MHz, $CDCl_3$): δ 0.87 (d, *J* = 6.8 Hz, 3H), 1.23-1.35 (m, 6H), 1.63-1.73 (m, 2H), 2.07-2.14 (m, 2H), 3.81-3.86 (m, 4H), 5.07-5.16 (m, 2H), 6.06-6.18 (m, 1H), 6.91-7.05 (m, 2H), 7.10-7.15 (m, 2H), 7.30 (d, *J* = 7.3 Hz, 1H), 7.43 (d, *J* = 7.3 Hz, 1H), 7.45-7.53 (m, 2H), 8.02-8.07 (m, 1H), 8.09-8.14 (m, 1H) ppm.

^{13}C{^{1}H} NMR (101 MHz, $CDCl_3$): δ 14.1, 22.6, 26.8, 29.3, 31.7, 37.4, 40.3, 42.6, 75.4, 116.3, 122.5 (2xC), 124.5, 125.0, 125.3 (3xC), 125.4, 125.5, 129.3, 131.5, 132.2, 133.5, 135.8, 136.9, 138.4 (2xC) ppm.

HRMS(ESI+, M/Z): Fragmentation observed. Calcd. for $C_{13}H_{17}S_2$ $[M]^+$ 237.0772, found: 237.0776. Calcd. for $C_{14}H_{13}$ $[M]^+$ 181.1017, found: 181.1013.

2-((4-allylnaphthalen-1-yl)methyl)-2-benzylbenzo[d][1,3]dithiole (**9fc**)

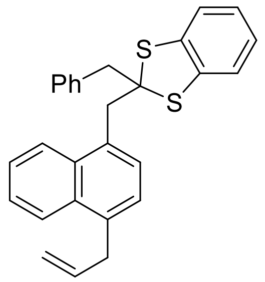

The compound was synthesized using the general procedure for alkylation (using BnBr). The crude compound was purified by column chromatography (SiO_2, pentane:CH_2Cl_2 95:5), giving **9fc** as a white solid (154.9 mg, 73% yield).

^{1}H NMR (400 MHz, $CDCl_3$): δ 3.51 (s, 2H), 3.80-3.89 (m, 4H), 5.07-5.18 (m, 2H), 6.07-6.19 (m, 1H), 6.83-6.89 (m, 2H), 6.96-7.01 (m, 2H), 7.26-7.35 (m, 4H), 7.37-7.42 (m, 2H), 7.44-7.53 (m, 3H), 7.96 (d, *J* = 8.1 Hz, 1H), 8.05 (d, *J* = 8.1 Hz, 1H) ppm.

^{13}C{^{1}H} NMR (101 MHz, $CDCl_3$): δ 37.4, 41.5, 48.3, 75.4, 116.3, 122.3 (2xC), 124.6, 125.1 (2xC), 125.2 (2xC), 125.3, 125.5, 127.1, 127.7 (2xC), 129.7, 131.4 (2xC), 131.5, 132.2, 133.2, 135.8, 136.2, 136.9, 138.2 (2xC) ppm.

HRMS(ESI+, M/Z): Fragmentation observed. Calcd. for $C_{14}H_{11}S_2$ $[M]^+$ 243.0302, found: 243.0308. Calcd. for $C_{14}H_{13}$ $[M]^+$ 181.1017, found: 181.1011.

2-(1-(4-allylnaphthalen-1-yl)ethyl)-2-methylbenzo[d][1,3]dithiole (**9ka**)

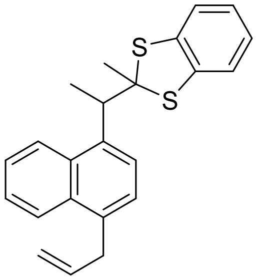

.

The compound was synthesized using the general procedure for alkylation (using MeI). The crude compound was purified by column chromatography (SiO_2, pentane:CH_2Cl_2 95:5), giving **9ka** as a colorless sticky oil (134.1 mg, 74% yield).

^{1}H NMR (400 MHz, $CDCl_3$): δ 1.70 (d, *J* = 6.9 Hz, 3H), 1.84 (s, 3H), 3.86 (d, *J* = 6.4 Hz, 2H), 4.53 (q, *J* = 6.9 Hz, 1H), 5.09-5.17 (m, 2H), 6.07-6.20 (m, 1H), 6.97-7.04 (m, 2H), 7.08-7.13 (m, 1H), 7.17-7.21 (m, 1H), 7.37 (d, *J* = 7.4 Hz, 1H), 7.48-7.55 (m, 2H), 7.62 (d, *J* = 7.4 Hz, 1H), 8.05-8.12 (m, 1H), 8.22-8.28 (m, 1H) ppm.

^{13}C{^{1}H} NMR (101 MHz, $CDCl_3$): δ 19.6, 28.6, 37.5, 42.2, 74.9, 116.3, 122.3, 122.4, 124.5, 124.8, 124.9, 125.2, 125.3 (2xC), 125.4, 125.6, 132.2, 132.6, 135.5, 136.9, 137.2, 138.3, 138.4 ppm.

HRMS(ESI+, M/Z): Fragmentation observed. Calcd. for $C_8H_7S_2$ $[M]^+$ 166.9989, found: 166.9984. Calcd. for $C_{15}H_{15}$ $[M]^+$ 195.1174, found: 195.1171.

2-(1-(4-allylnaphthalen-1-yl)ethyl)-2-hexylbenzo[d][1,3]dithiole (**9kb**)

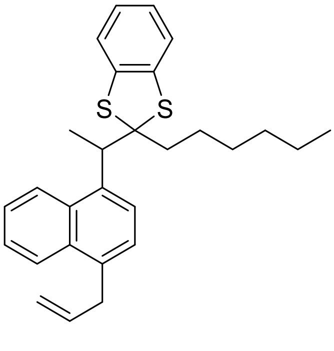

The compound was synthesized using the general procedure for alkylation (using HexyI). The crude compound was purified by column chromatography (SiO_2, pentane:CH_2Cl_2 95:5), giving **9kb** as a colorless sticky oil (168.7 mg, 78% yield).

^{1}H NMR (400 MHz, $CDCl_3$): δ 0.80 (d, *J* = 6.9 Hz, 3H), 1.08-1.22 (m, 6H), 1.54-1.65 (m, 2H), 1.71 (d, *J* = 6.8 Hz, 3H), 1.93-2.10 (m, 2H), 3.84 (d, *J* = 6.3 Hz, 2H), 4.37 (q, *J* = 6.8 Hz, 1H), 5.08-5.18 (m, 2H), 6.08-6.20 (m, 1H), 6.91-7.01 (m, 2H), 7.04-7.08 (m, 1H), 7.10-7.14 (m, 1H), 7.34 (d, *J* = 7.5 Hz, 1H), 7.50-7.56 (m, 2H), 7.74 (d, *J* = 7.5 Hz, 1H), 8.05-8.12 (m, 1H), 8.17-8.24 (m, 1H) ppm.

^{13}C{^{1}H} NMR (101 MHz, $CDCl_3$): δ 14.0, 19.7, 22.5, 26.2, 29.2, 31.5, 37.5, 41.1, 42.6, 79.6, 116.3, 121.4, 121.6, 124.2, 124.8, 124.9, 125.0, 125.2, 125.3 (2xC), 125.7, 132.2, 132.7, 135.1, 136.9, 137.6, 138.5, 138.9 ppm.

HRMS(ESI+, M/Z): Fragmentation observed. Calcd. for $C_{13}H_{17}S_2$ $[M]^+$ 237.0772, found: 237.0769. Calcd. for $C_{15}H_{15}$ $[M]^+$ 195.1174, found: 195.1170.

1,4-dipropylnaphthalene (**10fa**)[21]

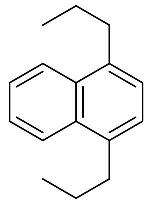

The compound was synthesized using the general procedure for reductive removal of benzothiol group. The crude compound was purified by column chromatography (SiO_2, pentane), giving **10fa** as a colorless oil (16.2 mg, 76% yield).

^{1}H NMR (400 MHz, $CDCl_3$): δ 1.03 (t, *J* = 7.3 Hz, 6H), 1.78 (m, 4H), 3.02 (t, *J* = 7.3 Hz, 4H), 7.24 (s, 2H), 7.46-7.53 (m, 2H), 8.04-8.10 (m, 2H) ppm.

^{13}C{^{1}H} NMR (101 MHz, $CDCl_3$): δ 14.3 (2xC), 23.9 (2xC), 35.2 (2xC), 124.6 (2xC), 125.0 (2xC), 125.6 (2xC), 132.2 (2xC), 136.8 (2xC) ppm.

1-propyl-4-octyl-naphthalene (**10fb**)

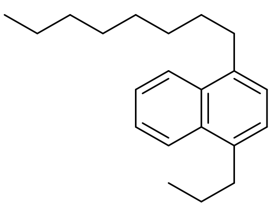

The compound was synthesized using the general procedure for reductive removal of benzothiol group. The crude compound was purified by column chromatography (SiO_2, pentane), giving **10fb** as a colorless oil (24.8 mg, 88% yield).

^{1}H NMR (400 MHz, $CDCl_3$): δ 0.88 (t, *J* = 6.7 Hz, 3H), 1.03 (t, *J* = 7.3 Hz, 3H), 1.24-1.38 (m, 8H), 1.39-1.49 (m, 2H), 1.39-1.83 (m, 4H), 2.99-3.06 (m, 4H), 7.24 (s, 2H), 7.47-7.53 (m, 2H), 8.04-8.10 (m, 2H) ppm.

^{13}C{^{1}H} NMR (101 MHz, $CDCl_3$): δ 14.1, 14.3, 22.7, 23.9, 29.3, 29.5, 29.9, 30.9, 31.9, 33.2, 35.2, 124.6 (2xC), 125.0 (2xC), 125.5, 125.6, 132.2, 132.2, 136.7, 137.1 ppm.

HRMS (MALDI - TOF): Calcd. for $C_{21}H_{31}$ $[M]^+$ 283.2426, found: 283.2428.

1-(3-phenylpropyl)-4-propyl-naphthalene (**10fc**)

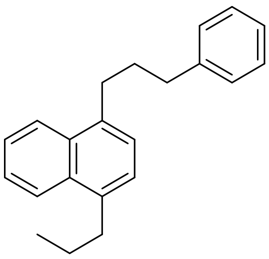

The compound was synthesized using the general procedure for reductive removal of benzothiol group. The crude compound was purified by column chromatography (SiO_2, pentane:CH_2Cl_2 99:1), giving **10fc** as a colorless oil (24.2 mg, 84% yield).

^{1}H NMR (400 MHz, $CDCl_3$): δ 1.05 (t, *J* = 7.3 Hz, 3H), 1.79 (m, 2H), 2.11 (m, 2H), 2.78 (t, *J* = 7.3 Hz, 2H), 3.04 (t, *J* = 7.8 Hz, 2H), 3.10 (t, *J* = 7.8 Hz, 2H), 7.18-7.28 (m, 5H), 7.29-7.34 (m, 2H), 7.49-7.54 (m, 2H), 7.98-8.04 (m, 1H), 5.05-8.12 (m, 1H) ppm.

^{13}C{^{1}H} NMR (101 MHz, $CDCl_3$): δ 14.3, 23.9, 32.3, 32.6, 35.2, 35.9, 124.5, 124.6, 125.1, 125.2, 125.6, 125.6, 125.8, 128.3 (2xC), 128.5 (2xC), 132.2, 132.3, 136.5, 137.0, 142.3 ppm.

HRMS (MALDI - TOF): Calcd. for $C_{22}H_{25}$ $[M]^+$ 289.1956, found: 289.1950.

1-(1-methyl-propyl)-4-propylnaphthalene (**10ka**)

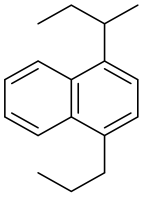

The compound was synthesized using the general procedure for reductive removal of benzothiol group. The crude compound was purified by column chromatography (SiO_2, pentane), giving **10ka** as a colorless oil (19.2 mg, 85% yield).

^{1}H NMR (400 MHz, $CDCl_3$): δ 0.94 (t, *J* = 7.4 Hz, 3H), 1.04 (t, *J* = 7.4 Hz, 3H), 1.37 (d, *J* = 6.9 Hz, 3H), 1.64-1.92 (m, 4H), 3.03 (t, *J* = 7.4 Hz, 2H), 3.50 (m, 1H), 7.30 (s, 2H), 7.47-7.53 (m, 2H), 8.06-8.11 (m, 1H), 8.13-8.19 (m, 1H) ppm.

^{13}C{^{1}H} NMR (101 MHz, $CDCl_3$): δ 12.3, 14.4, 21.2, 23.9, 30.5, 35.1, 35.3, 122.1, 123.9, 124.7, 124.9, 125.0, 125.7, 132.1, 132.3, 136.3, 141.7 ppm.

HRMS (MALDI - TOF): Calcd. for $C_{17}H_{23}$ $[M]^+$ 227.1800, found: 227.1799.

1-(1-methyl-octyl)-4-propylnaphthalene (**10kb**)

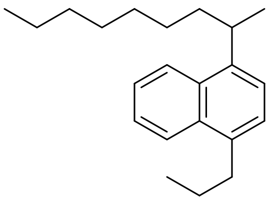

The compound was synthesized using the general procedure for reductive removal of benzothiol group. The crude compound was purified by column chromatography (SiO_2, pentane), giving **10kb** as a colorless oil (24.0 mg, 89% yield).

^{1}H NMR (400 MHz, $CDCl_3$): δ 0.88 (t, *J* = 6.7 Hz, 3H), 1.05 (t, *J* = 7.4 Hz, 3H), 1.19-1.42 (m, 13H), 1.59-1.71 (m, 1H), 1.74-1.87 (m, 3H), 3.04 (t, *J* = 7.7 Hz, 3H), 3.57 (m, 1H), 7.32 (s, 2H), 7.48-7.54 (m, 2H), 8.07-8.12 (m, 1H), 8.15-8.20 (m, 1H) ppm.

$^{13}C\{^{1}H\}$ **NMR** (101 MHz, $CDCl_3$): δ 14.1, 14.4, 21.7, 22.7, 23.9, 27.9, 29.3, 29.9, 31.9, 33.5, 35.3, 37.9, 122.1, 123.8, 124.7, 124.9, 125.0, 125.8, 132.0, 132.3, 136.3, 142.1 ppm.

HRMS (MALDI - TOF): Calcd. for $C_{22}H_{33}$ $[M]^+$ 297.2582, found: 297.2580.

1-(4-propylnaphthalen-1-yl)propan-2-one (**11**)

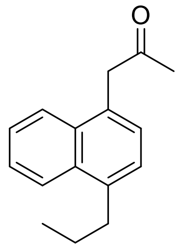

The compound was synthesized using the general procedure for oxidative removal of benzothiol group. The crude compound was purified by column chromatography (SiO_2, pentane:CH_2Cl_2 50:50), giving **11** as a colorless oil (37.6 mg, 83% yield).

^{1}H **NMR** (400 MHz, $CDCl_3$): δ 1.04 (t, *J* = 7.7 Hz, 3H), (m, 2H), 2.11 (s, 3H), 3.05 (t, *J* = 7.7 Hz, 2H), 4.09 (s, 2H), 7.28-7.34 (m, 2H), 7.49-7.56 (m, 2H), 7.87-7.92 (m, 1H), 8.07-8.12 (m, 1H) ppm.

$^{13}C\{^{1}H\}$ **NMR** (101 MHz, $CDCl_3$): δ 14.3, 23.9, 28.9, 35.2, 49.4, 124.5, 124.7, 125.6, 125.7, 126.0, 128.0, 129.2, 132.4, 132.5, 138.8, 207.4 ppm.

HRMS (MALDI - TOF): Calcd. for $C_{16}H_{19}O$ $[M+H]^+$ 227.1413, found: 227.1415.

10. NMR spectra

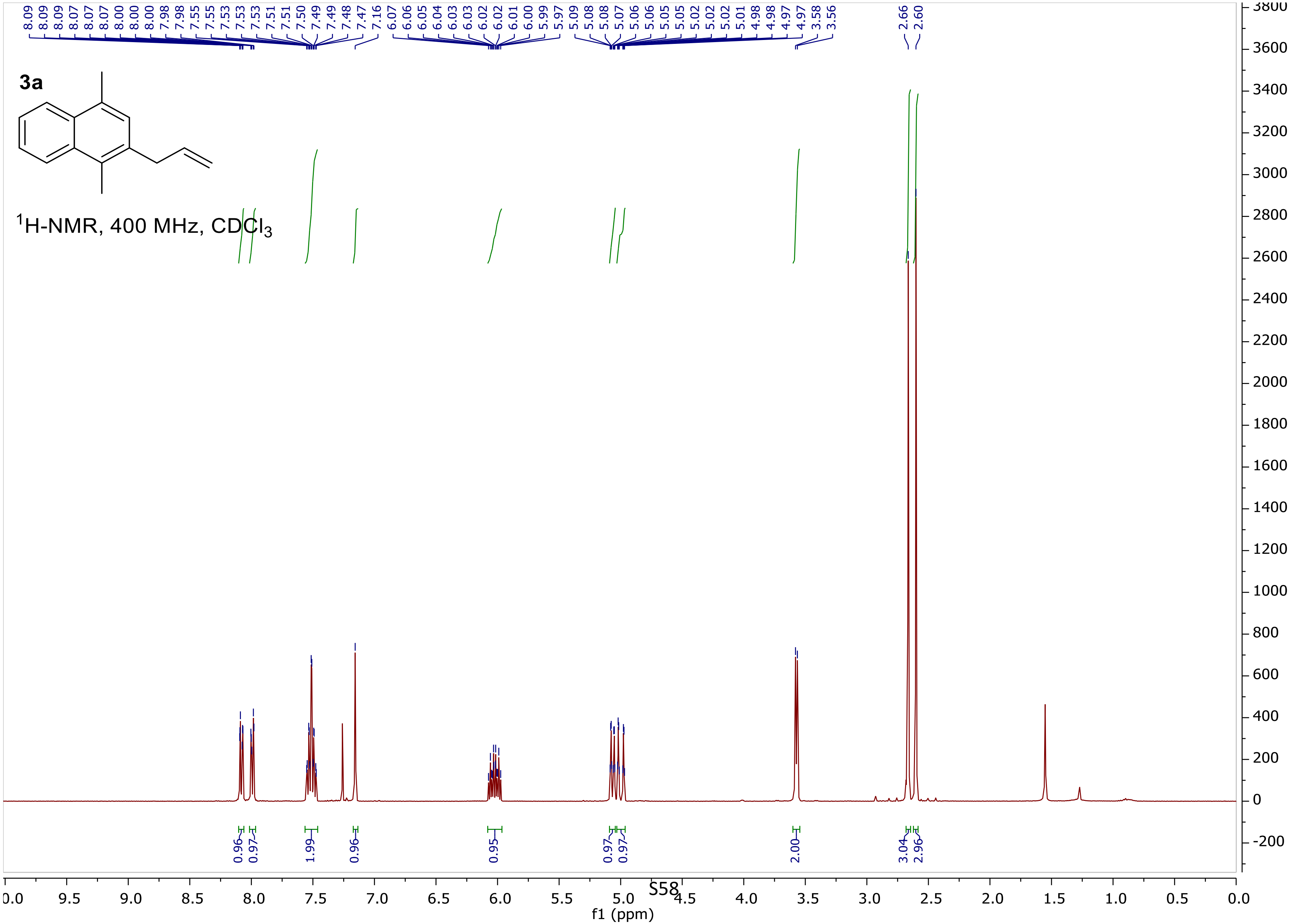
3a
1H-NMR, 400 MHz, CDCl3
8.09 8.09 8.07 8.07 8.07 8.00 8.00 8.00 7.98 7.98 7.55 7.55 7.53 7.53 7.53 7.51 7.51 7.50 7.49 7.49 7.48 7.47 7.16 6.07 6.06 6.05 6.04 6.03 6.03 6.02 6.02 6.01 6.00 5.99 5.97 5.09 5.08 5.08 5.07 5.06 5.06 5.05 5.02 5.02 5.02 5.01 4.98 4.98 4.97 4.97 3.58 3.56 2.66 2.60
0.96 0.97 1.99 0.96 0.95 0.97 0.97 2.00 3.04 2.96
f1 (ppm)

3a

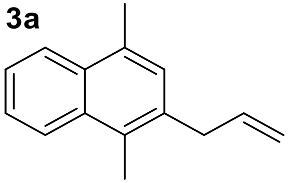

$^{13}C\{^{1}H\}$-NMR, 101 MHz, $CDCl_3$

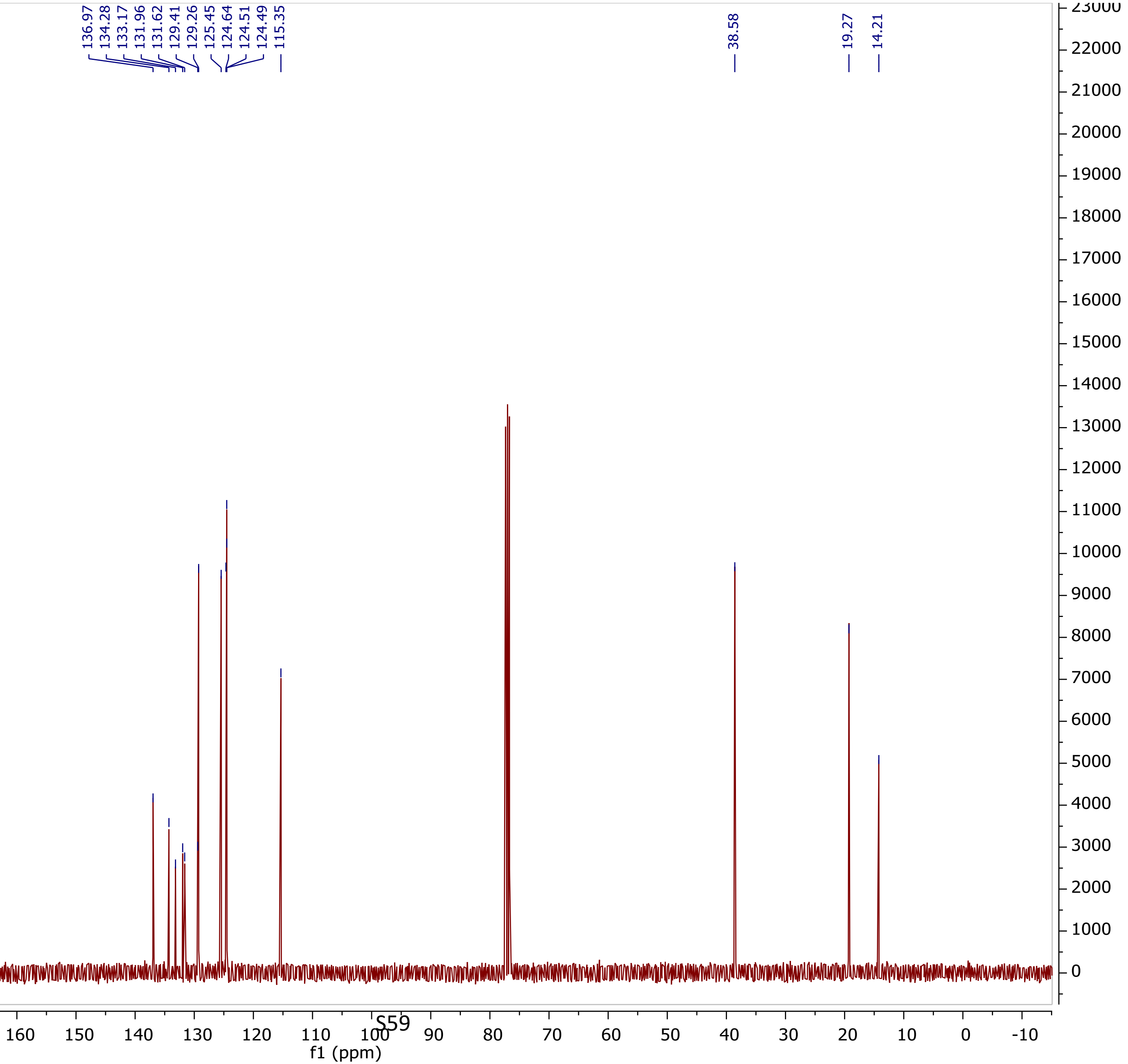

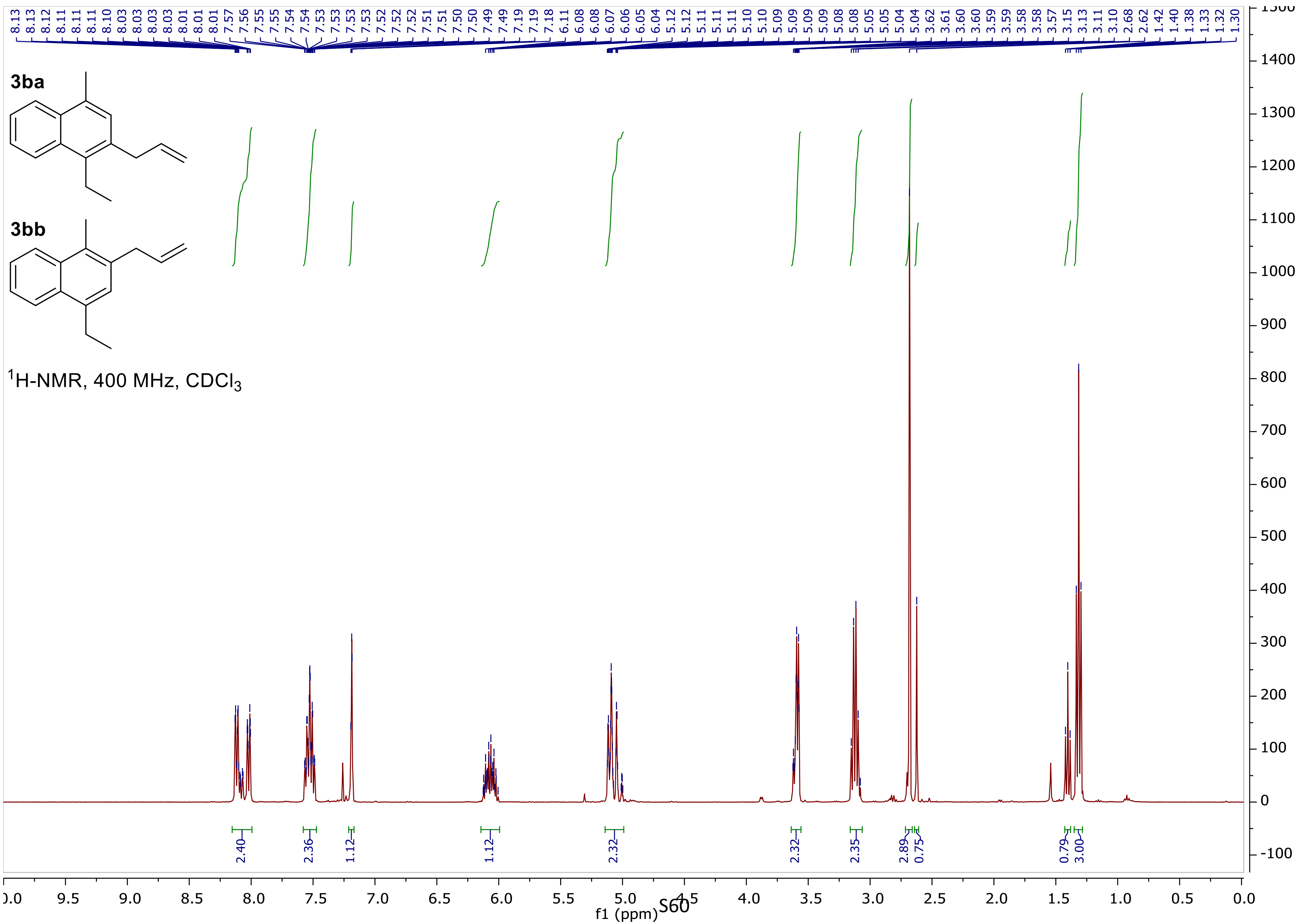
3ba
3bb
1H-NMR, 400 MHz, CDCl3
f1 (ppm)

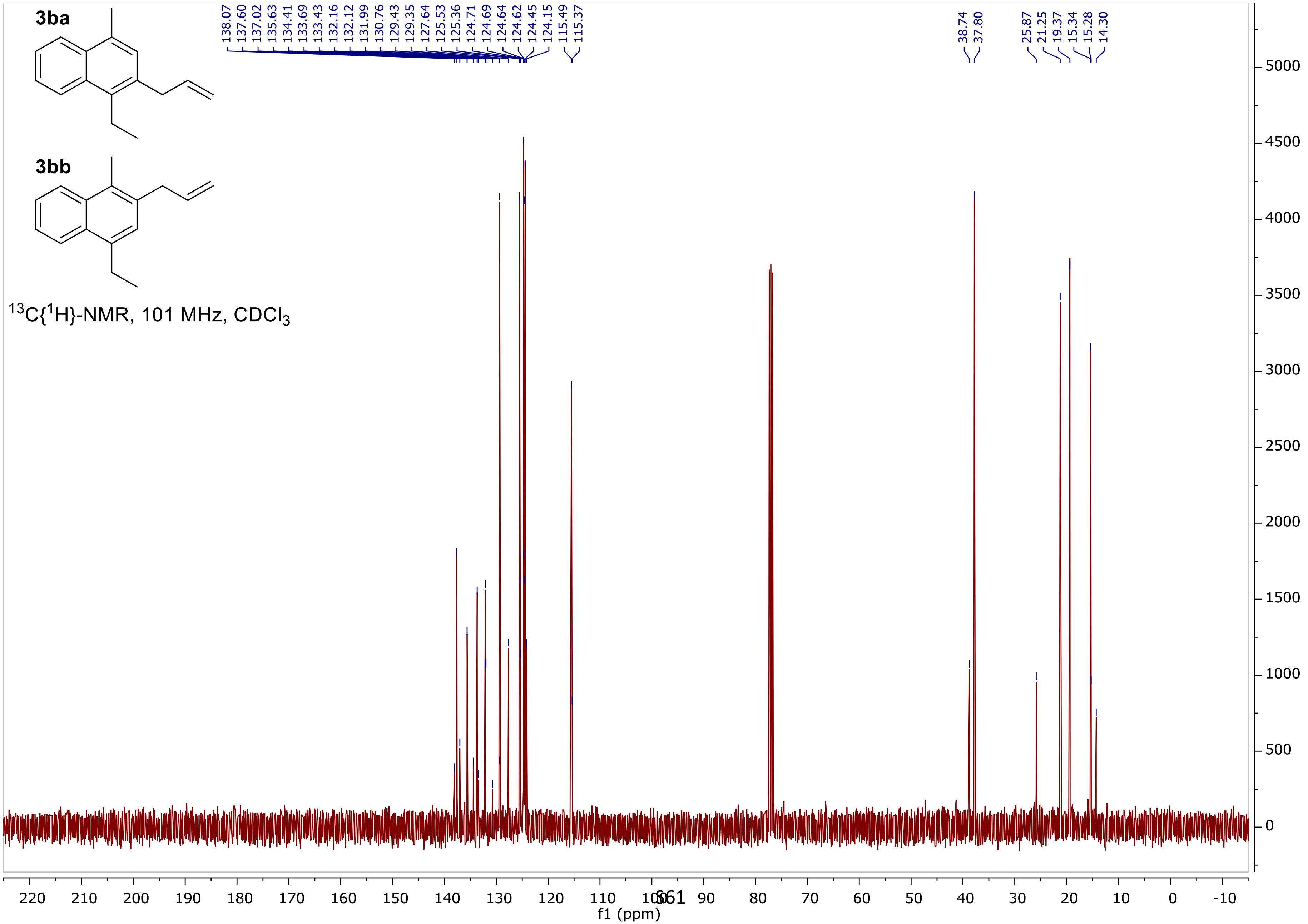
3ba
3bb
$^{13}C\{^1H\}$-NMR, 101 MHz, $CDCl_3$
138.07 137.60 137.02 135.63 134.41 133.69 133.43 132.16 132.12 131.99 130.76 129.43 129.35 127.64 125.53 125.36 124.71 124.69 124.64 124.62 124.45 124.15 115.49 115.37
38.74 37.80
25.87 21.25 19.37 15.34 15.28 14.30
5000 4500 4000 3500 3000 2500 2000 1500 1000 500 0
220 210 200 190 180 170 160 150 140 130 120 110 100 90 80 70 60 50 40 30 20 10 0 -10
f1 (ppm)

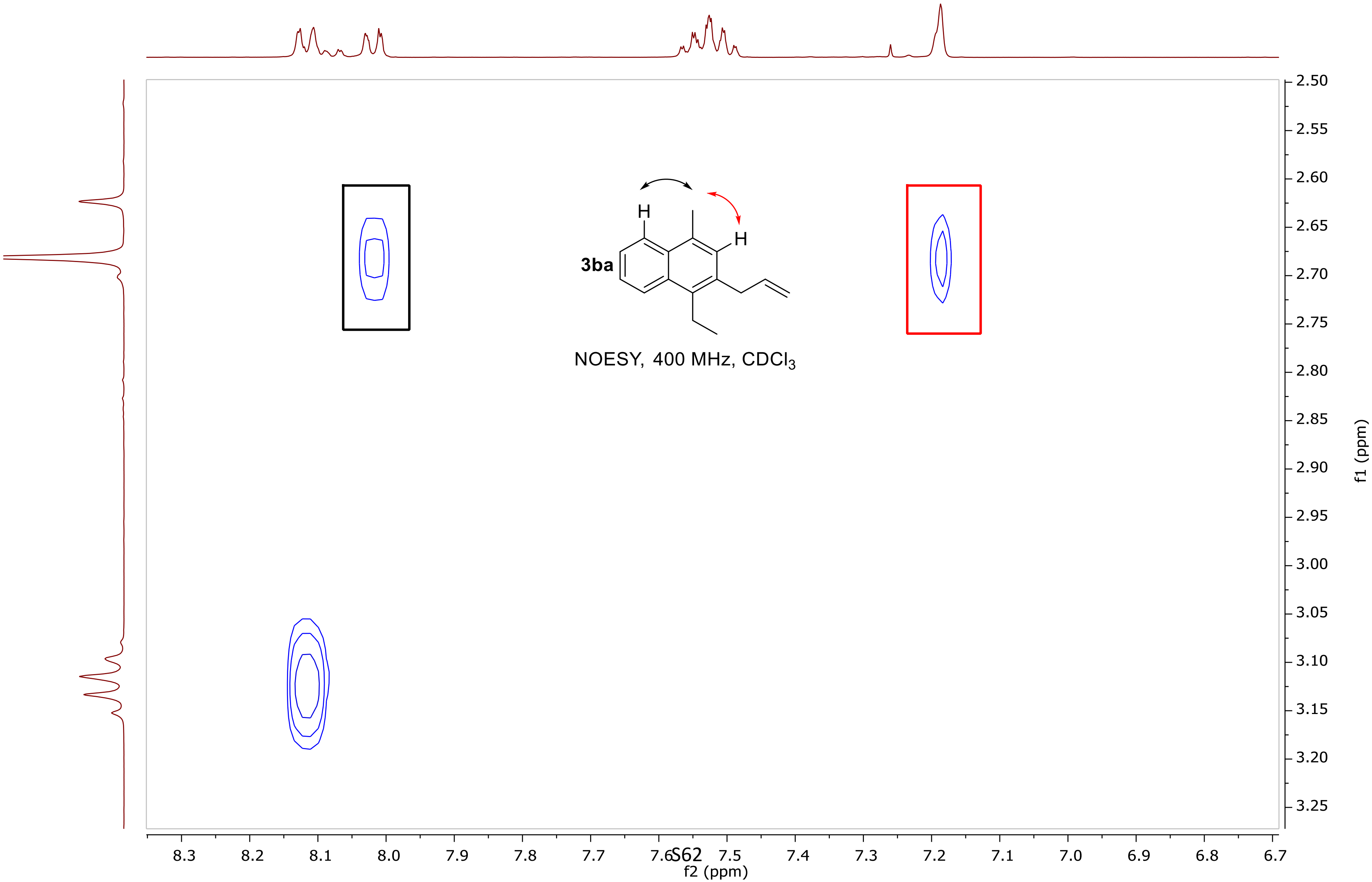

3ba
NOESY, 400 MHz, CDCl3
f2 (ppm)
f1 (ppm)

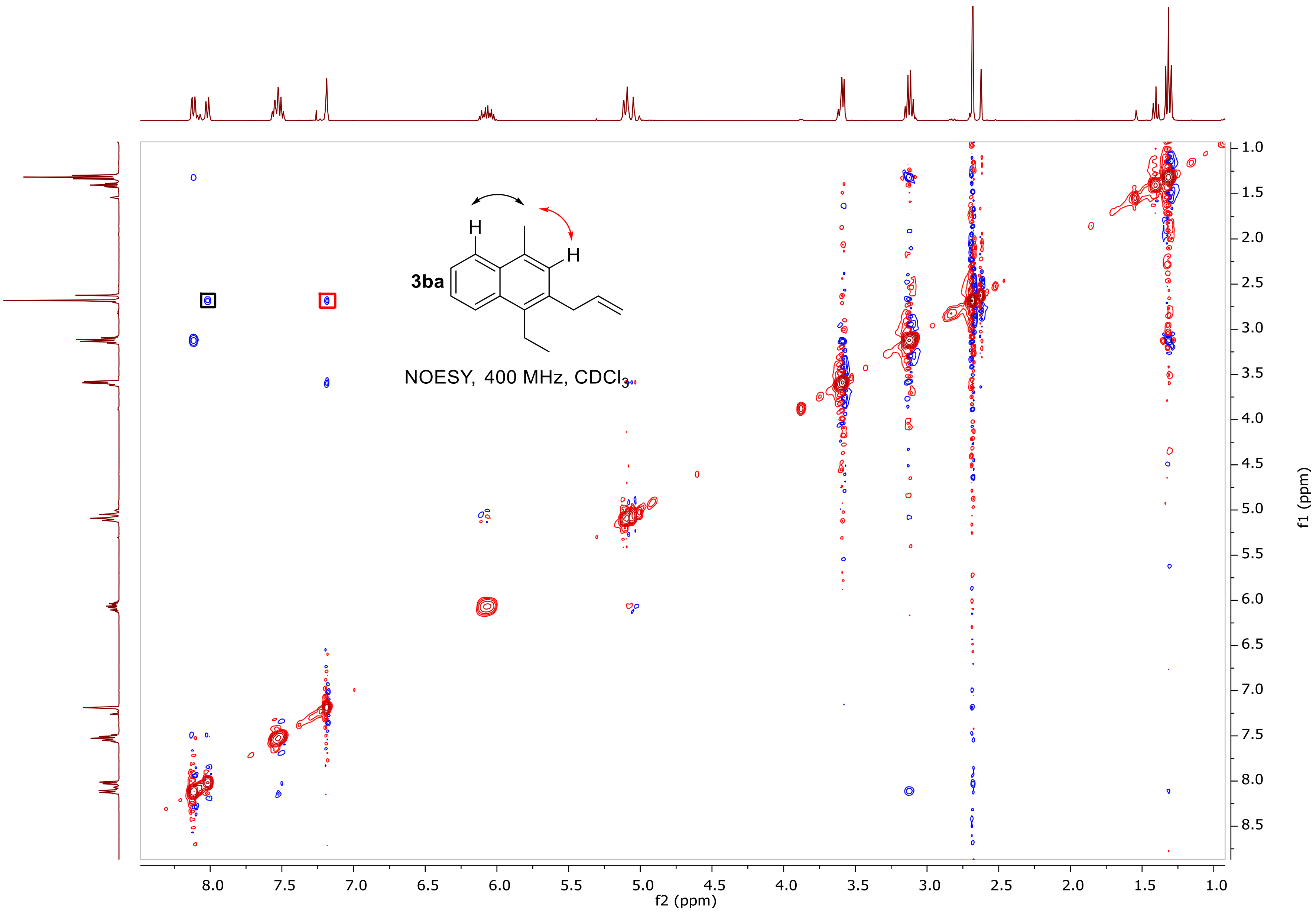

3ba
NOESY, 400 MHz, CDCl3
f2 (ppm)
f1 (ppm)

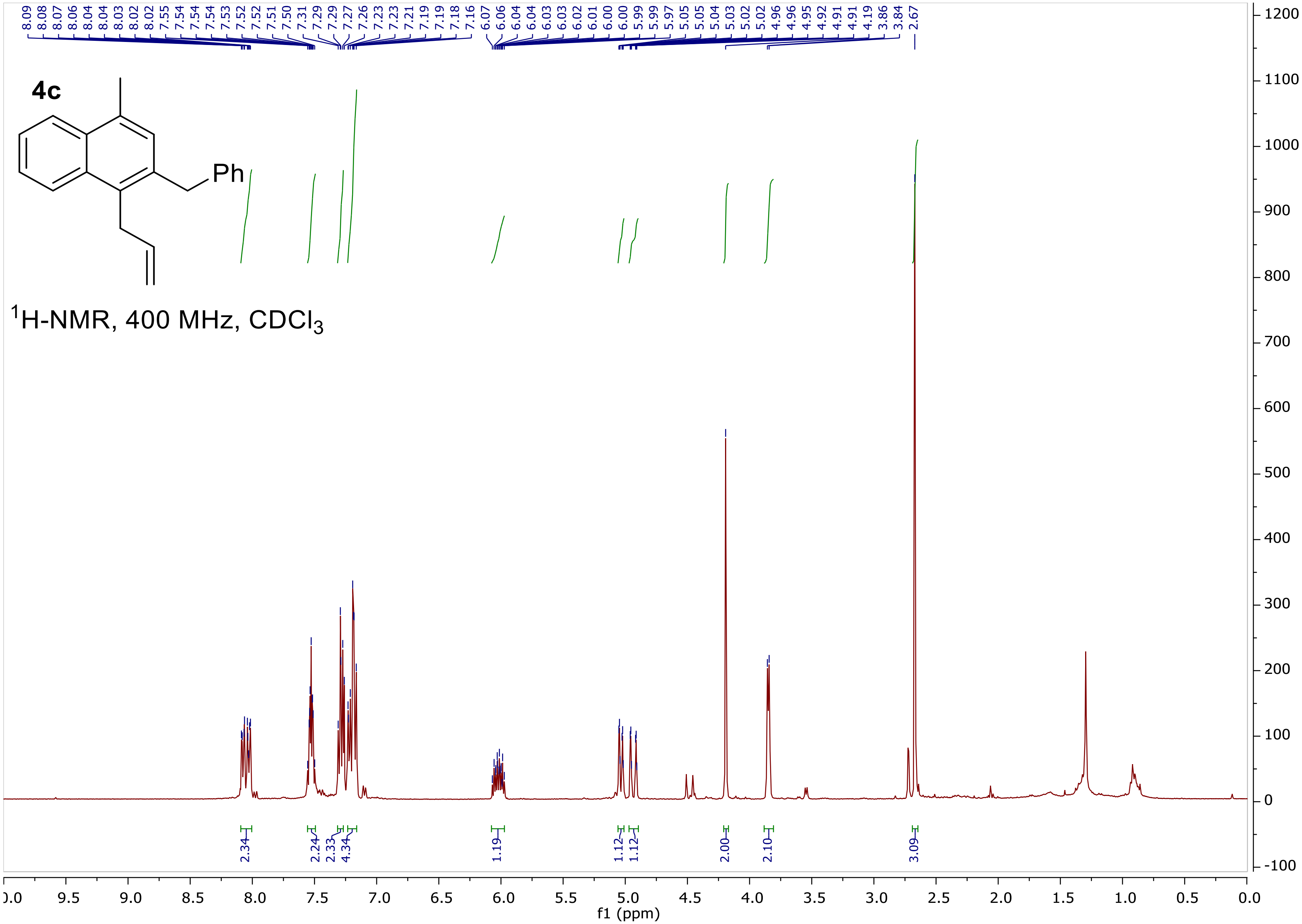

4c
Ph
¹H-NMR, 400 MHz, CDCl3
f1 (ppm)

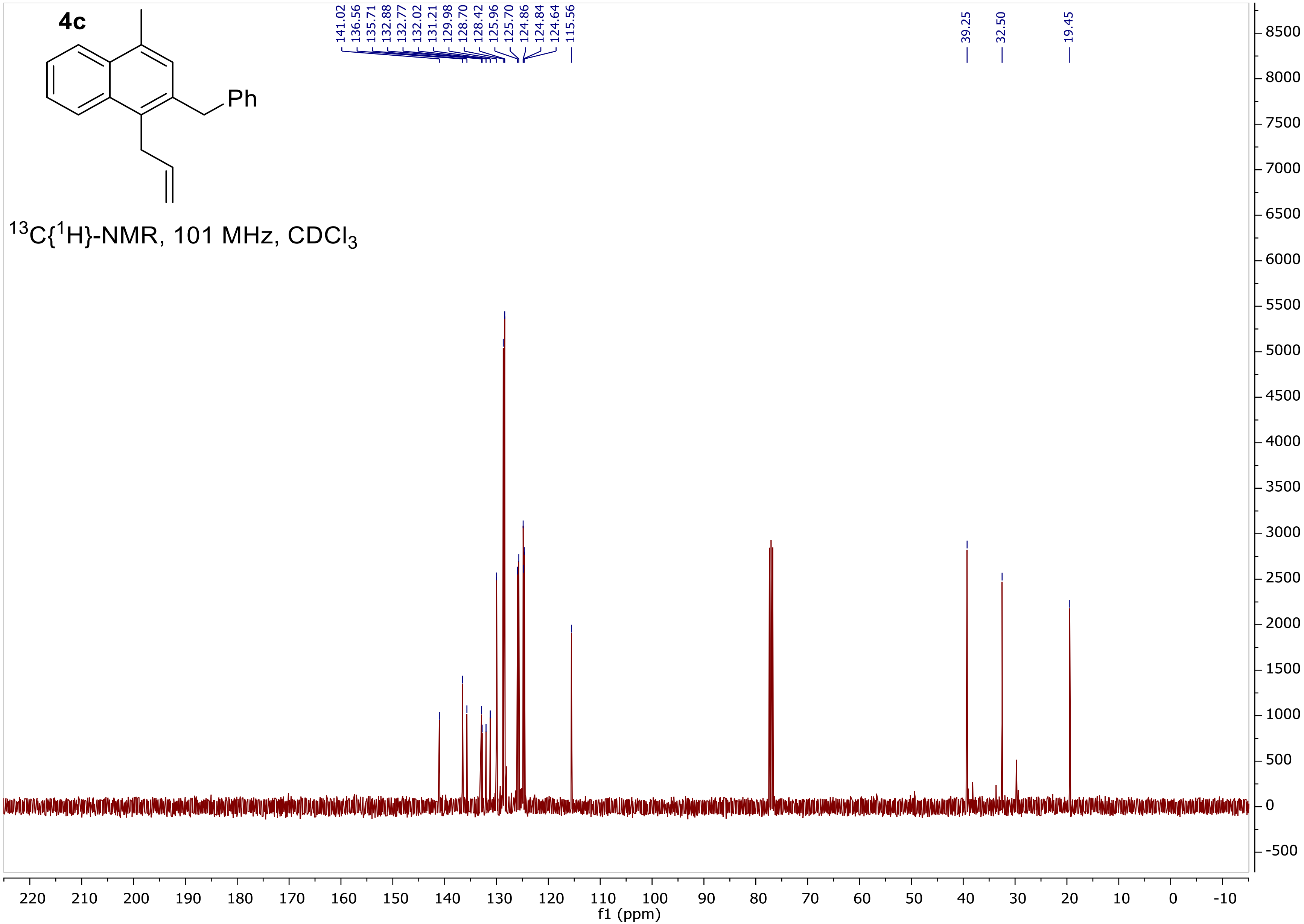
4c
Ph
13C{1H}-NMR, 101 MHz, CDCl3
141.02
136.56
135.71
132.88
132.77
132.02
131.21
129.98
128.70
128.42
125.96
125.70
124.86
124.84
124.64
115.56
39.25
32.50
19.45
f1 (ppm)

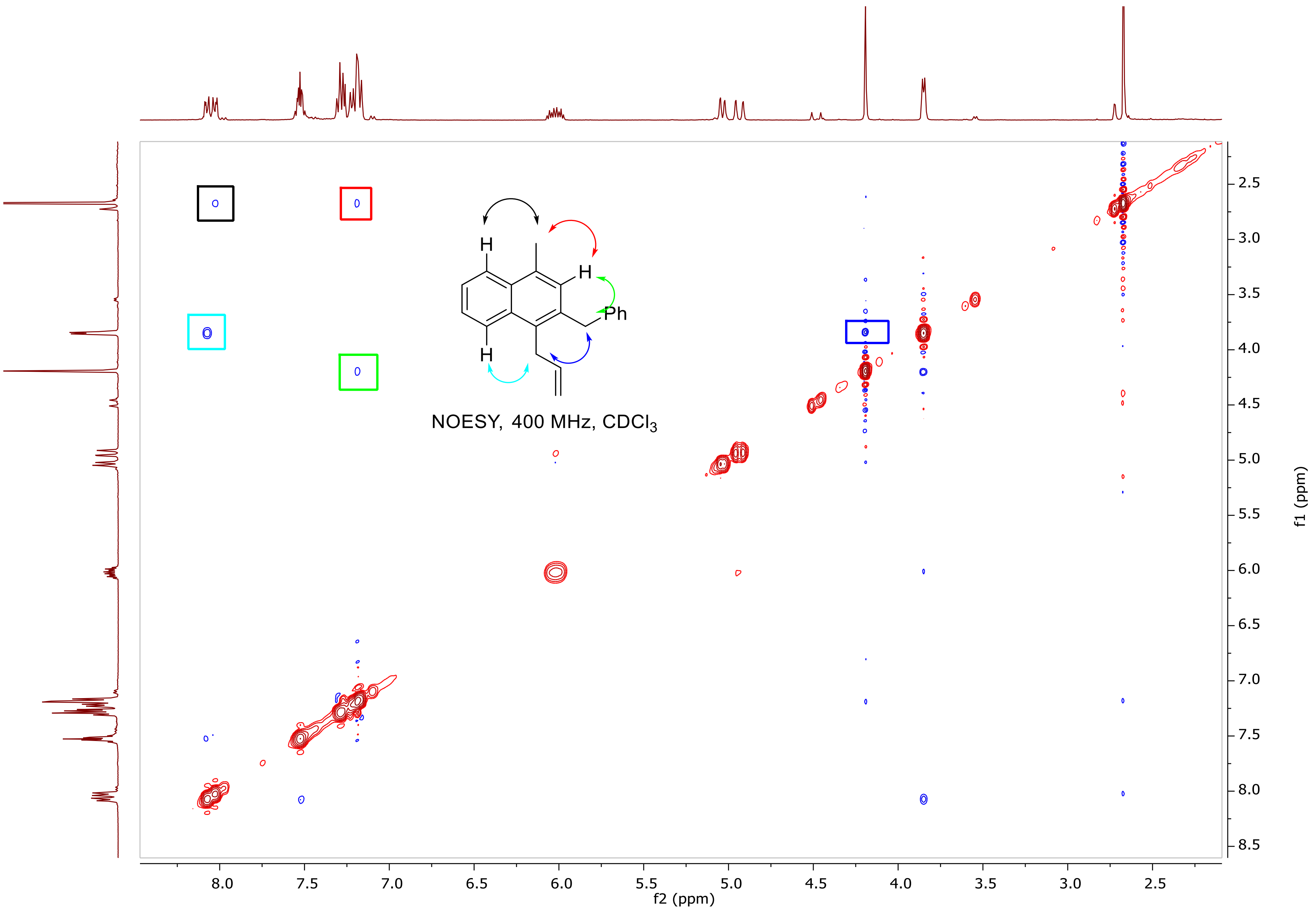

H
H
H
Ph
NOESY, 400 MHz, CDCl3
f2 (ppm)
f1 (ppm)

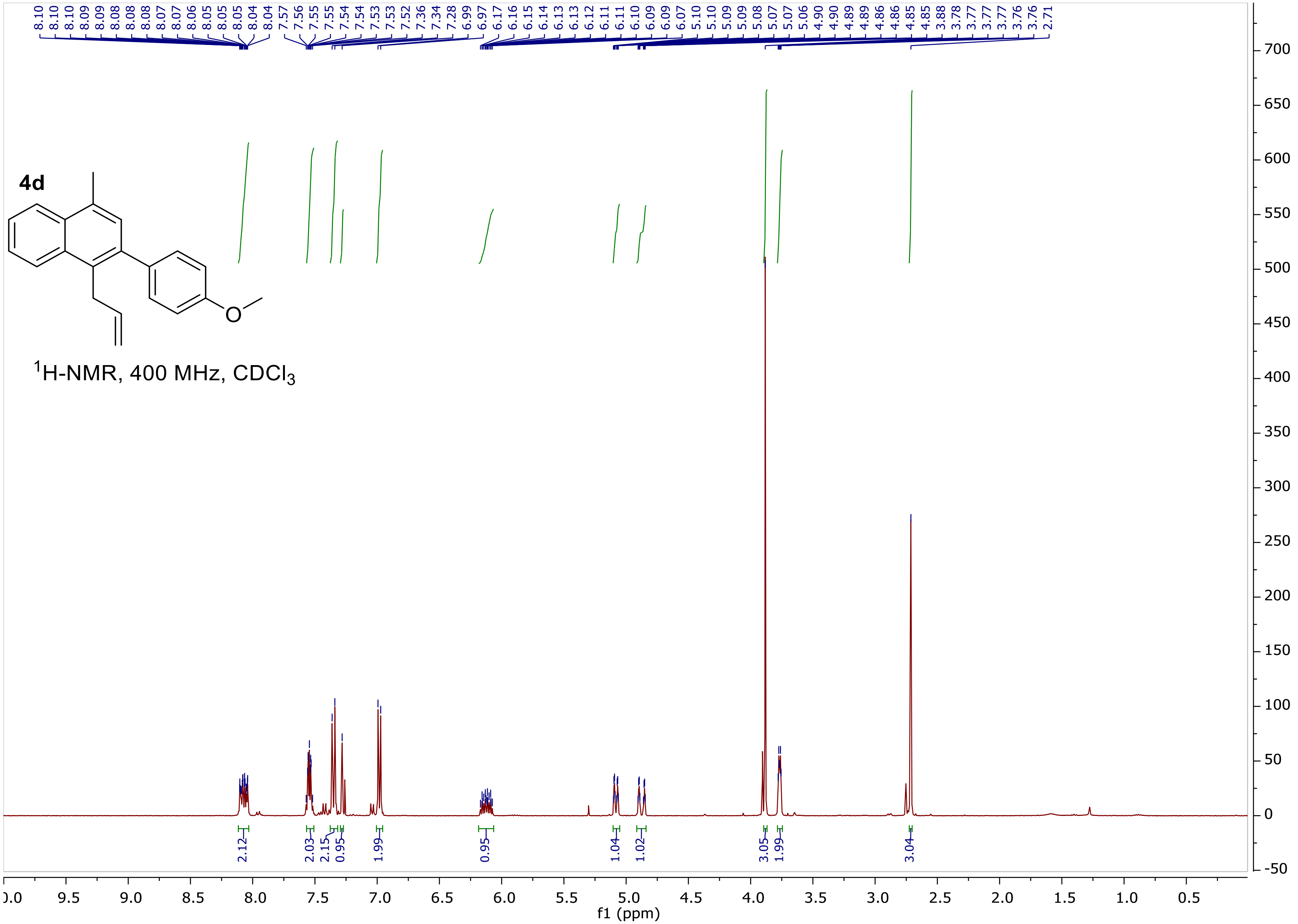
4d
O
1H-NMR, 400 MHz, CDCl3
f1 (ppm)

4d

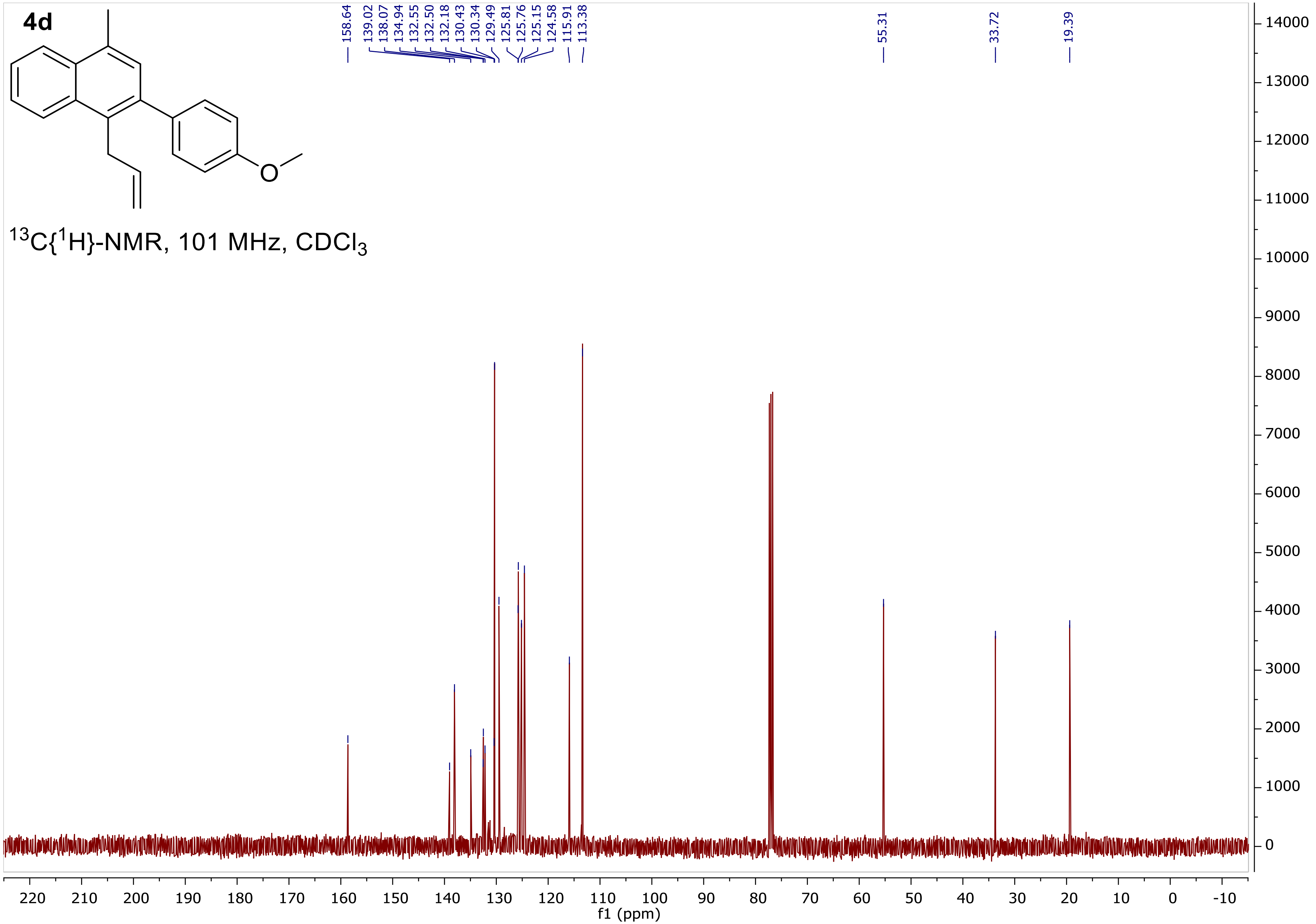

$^{13}C\{^{1}H\}$-NMR, 101 MHz, $CDCl_3$

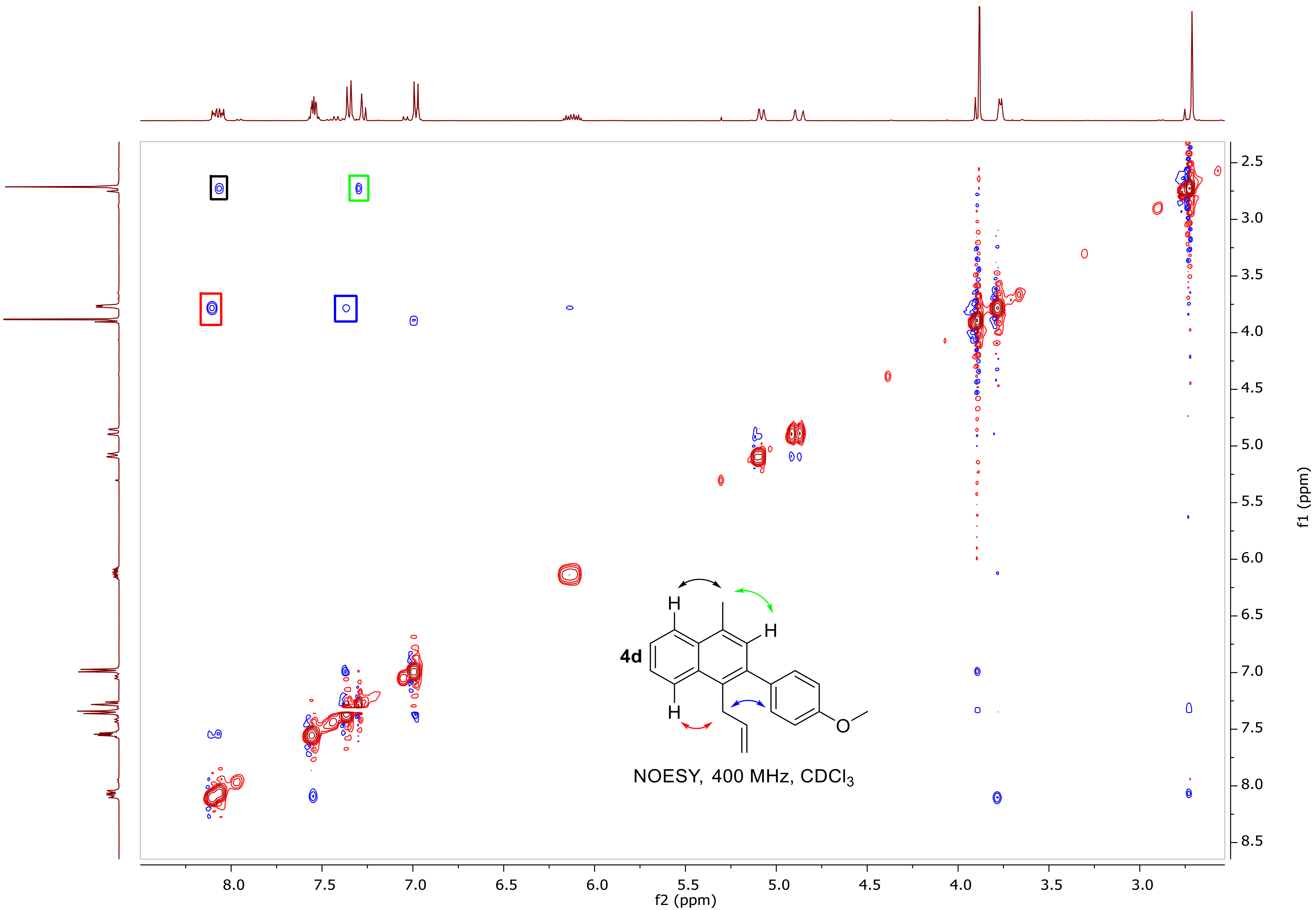
4d
H
H
H
O
NOESY, 400 MHz, CDCl3
f2 (ppm)
f1 (ppm)
8.0
7.5
7.0
6.5
6.0
5.5
5.0
4.5
4.0
3.5
3.0
2.5
8.5

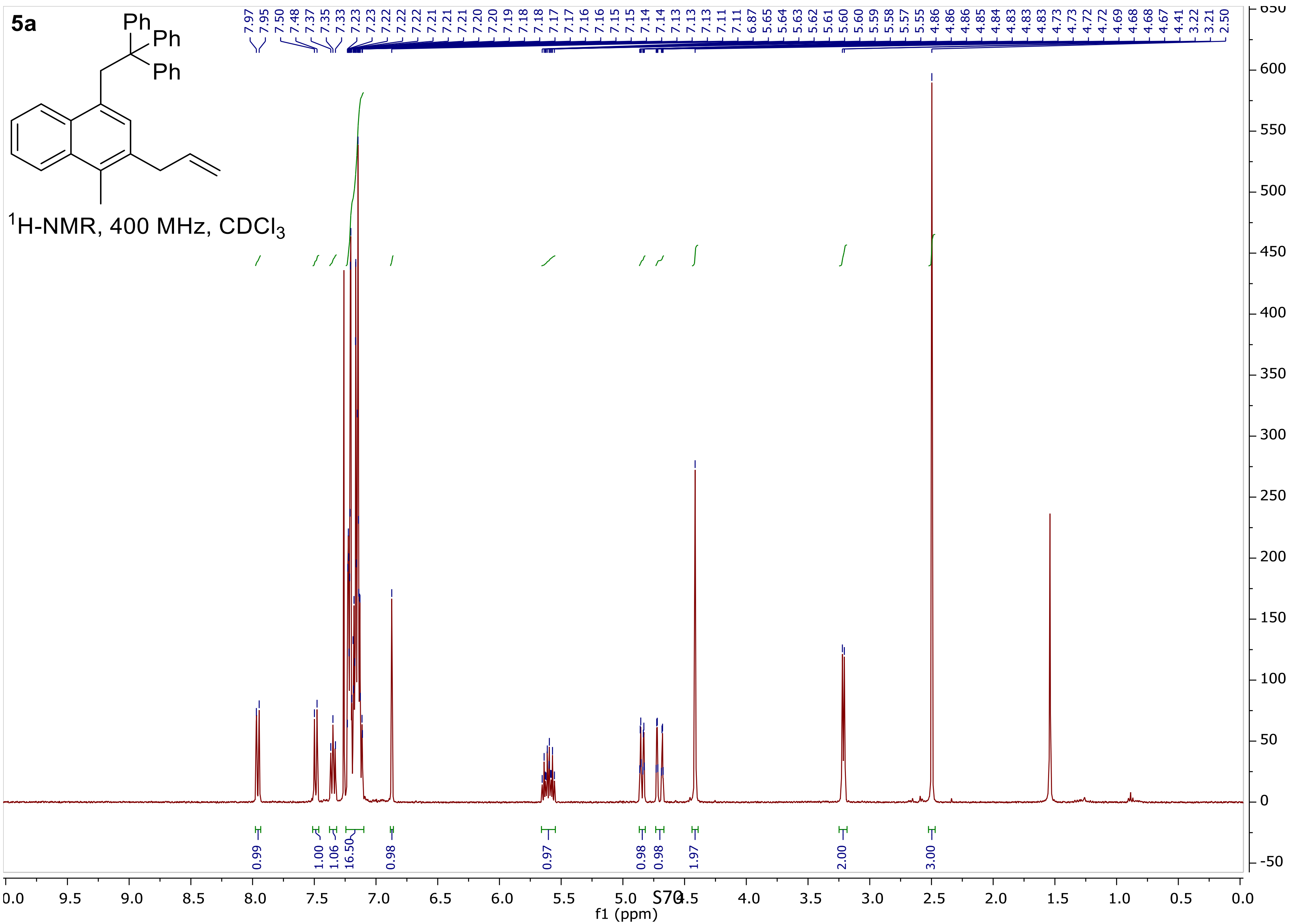

5a
Ph
Ph
Ph
¹H-NMR, 400 MHz, CDCl3
7.97
7.95
7.50
7.48
7.37
7.35
7.33
7.23
7.23
7.22
7.22
7.22
7.21
7.21
7.21
7.20
7.20
7.19
7.18
7.18
7.17
7.17
7.16
7.15
7.15
7.14
7.14
7.13
7.13
7.13
7.11
7.11
6.87
5.65
5.64
5.63
5.62
5.61
5.60
5.60
5.59
5.58
5.57
5.55
4.86
4.86
4.86
4.85
4.84
4.83
4.83
4.83
4.73
4.73
4.72
4.72
4.69
4.68
4.67
4.41
3.22
3.21
2.50
0.99
1.00
1.06
16.50
0.98
0.97
0.98
0.98
1.97
2.00
3.00
f1 (ppm)

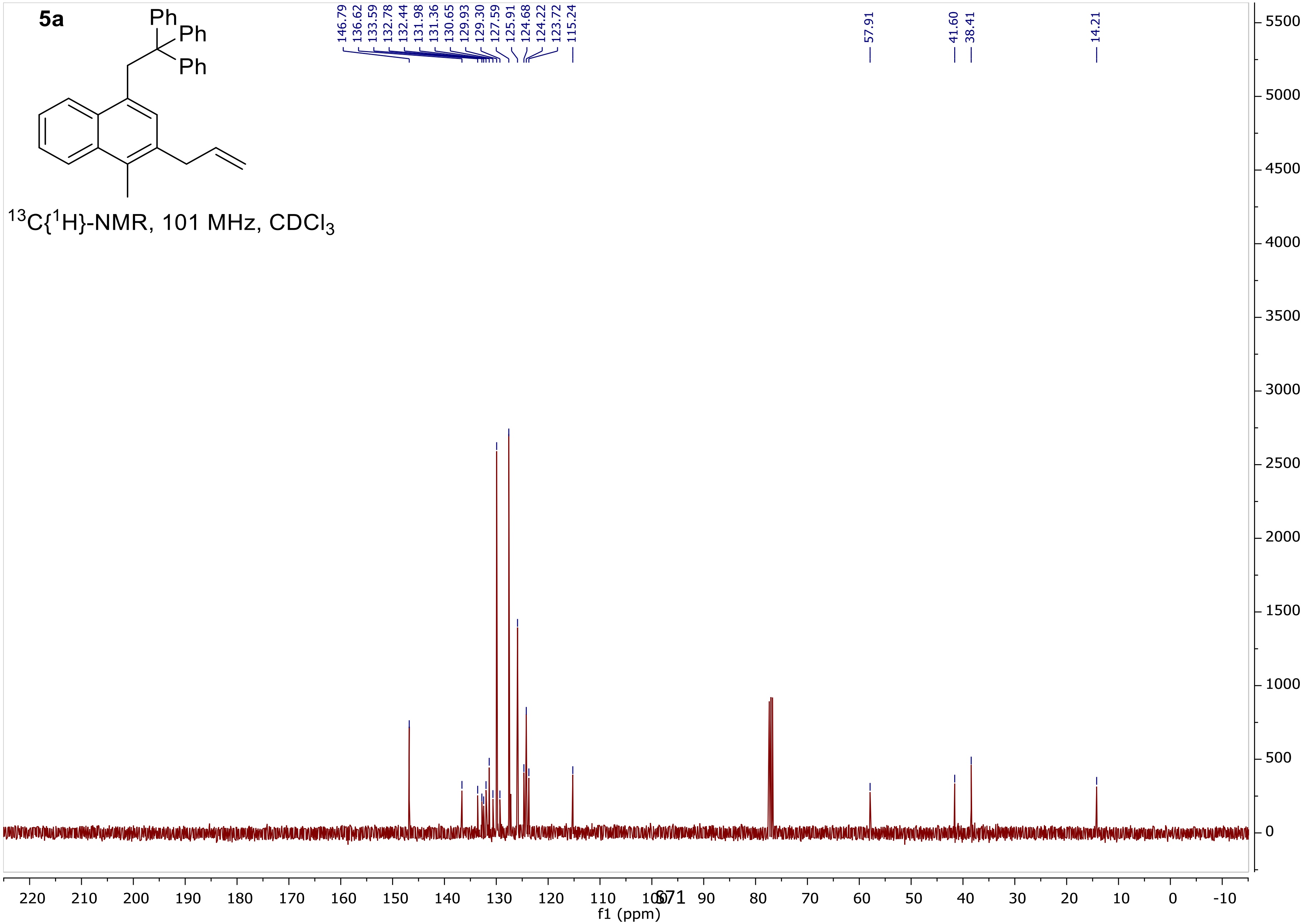
5a
Ph
Ph
Ph
$^{13}C\{^{1}H\}$-NMR, 101 MHz, $CDCl_3$
146.79
136.62
133.59
132.78
132.44
131.98
131.36
130.65
129.93
129.30
127.59
125.91
124.68
124.22
123.72
115.24
57.91
41.60
38.41
14.21
f1 (ppm)

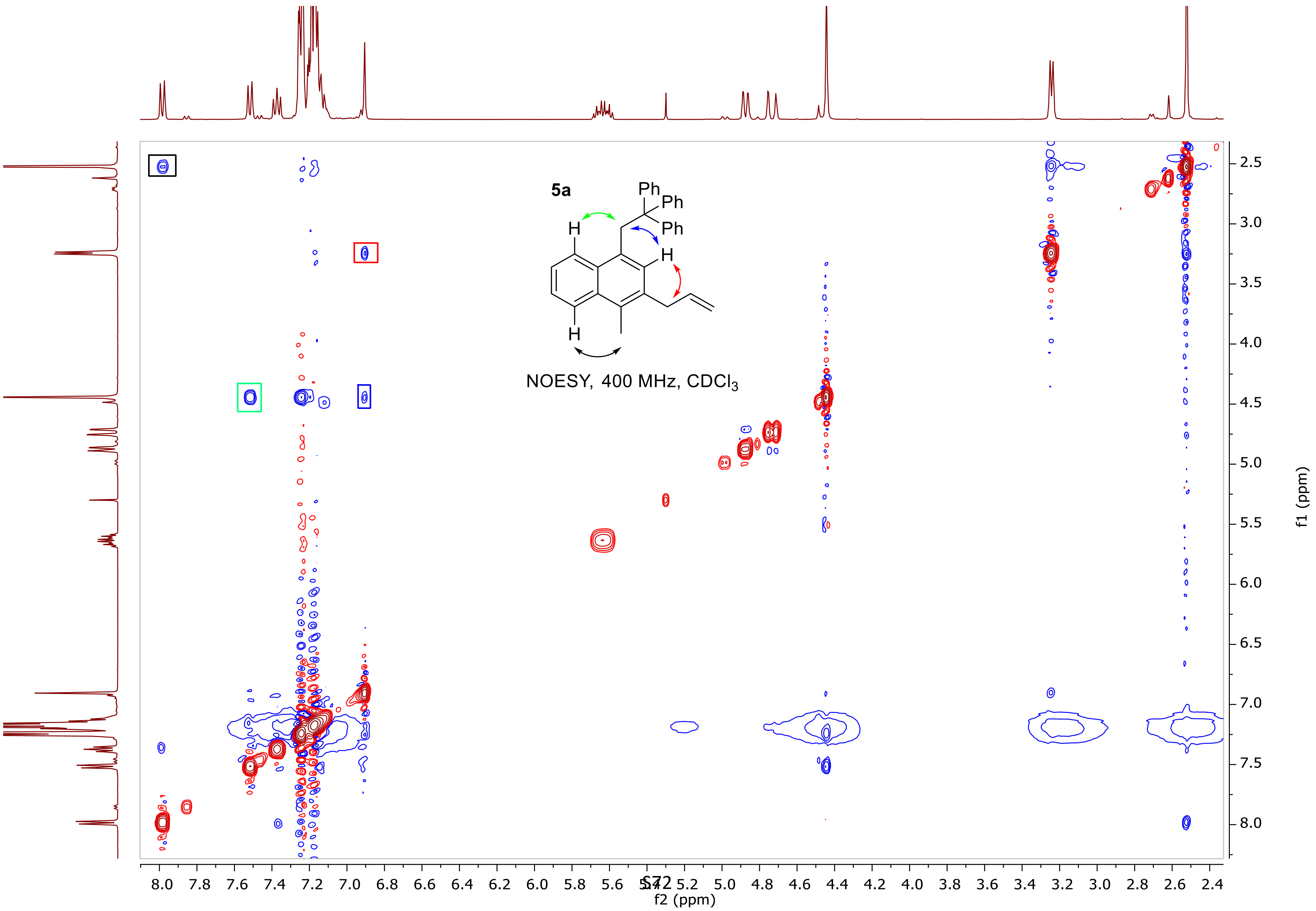
5a
Ph
Ph
Ph
H
H
H
NOESY, 400 MHz, CDCl3
f2 (ppm)
f1 (ppm)

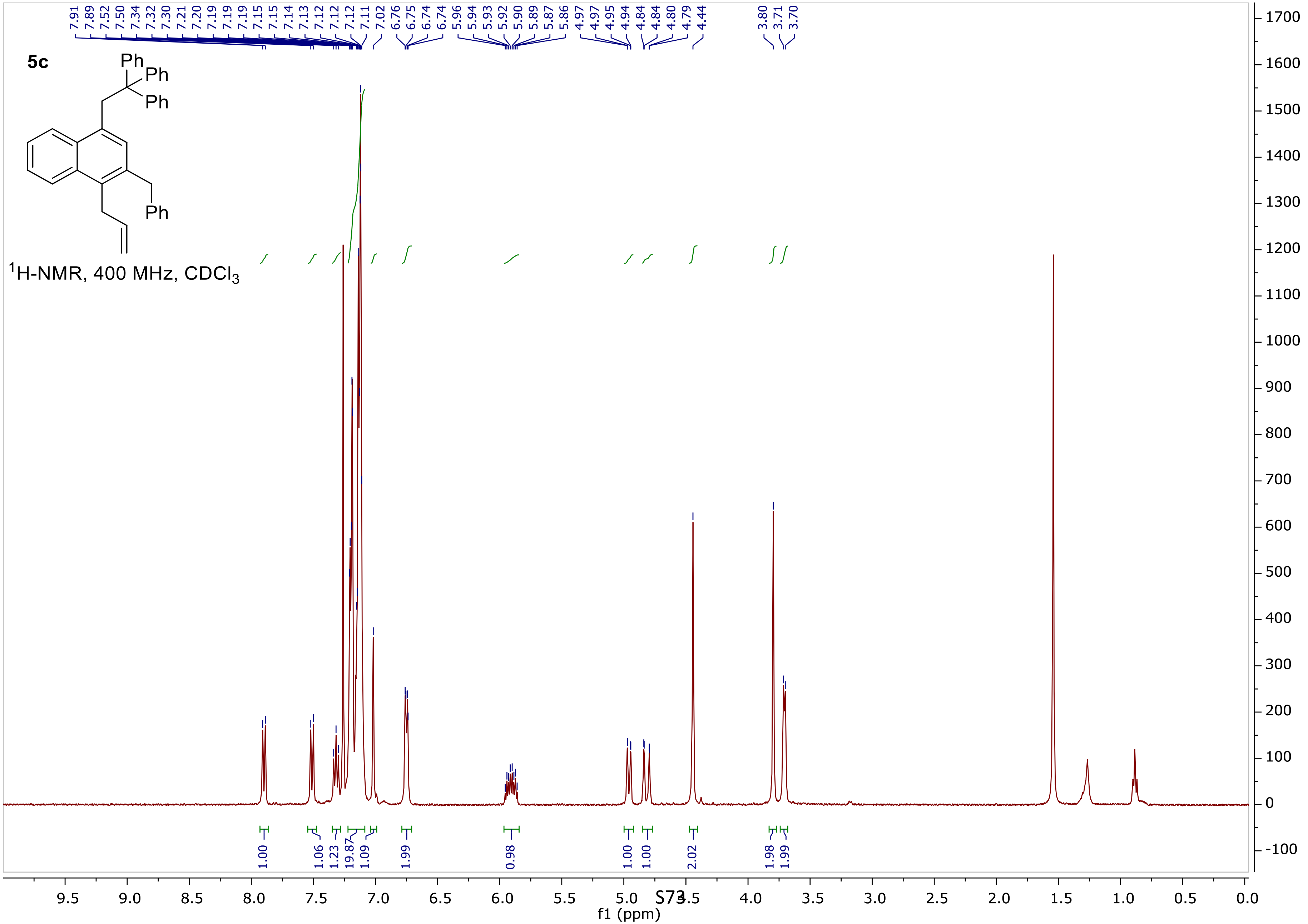
5c
Ph
Ph
Ph
Ph
1H-NMR, 400 MHz, CDCl3
7.91
7.89
7.52
7.50
7.34
7.32
7.30
7.21
7.20
7.19
7.19
7.19
7.15
7.15
7.14
7.13
7.12
7.12
7.12
7.11
7.02
6.76
6.75
6.74
6.74
5.96
5.94
5.93
5.92
5.90
5.89
5.87
5.86
4.97
4.97
4.95
4.94
4.84
4.84
4.80
4.79
4.44
3.80
3.71
3.70
1.00
1.06
1.23
19.87
1.09
1.99
0.98
1.00
1.00
2.02
1.98
1.99
f1 (ppm)

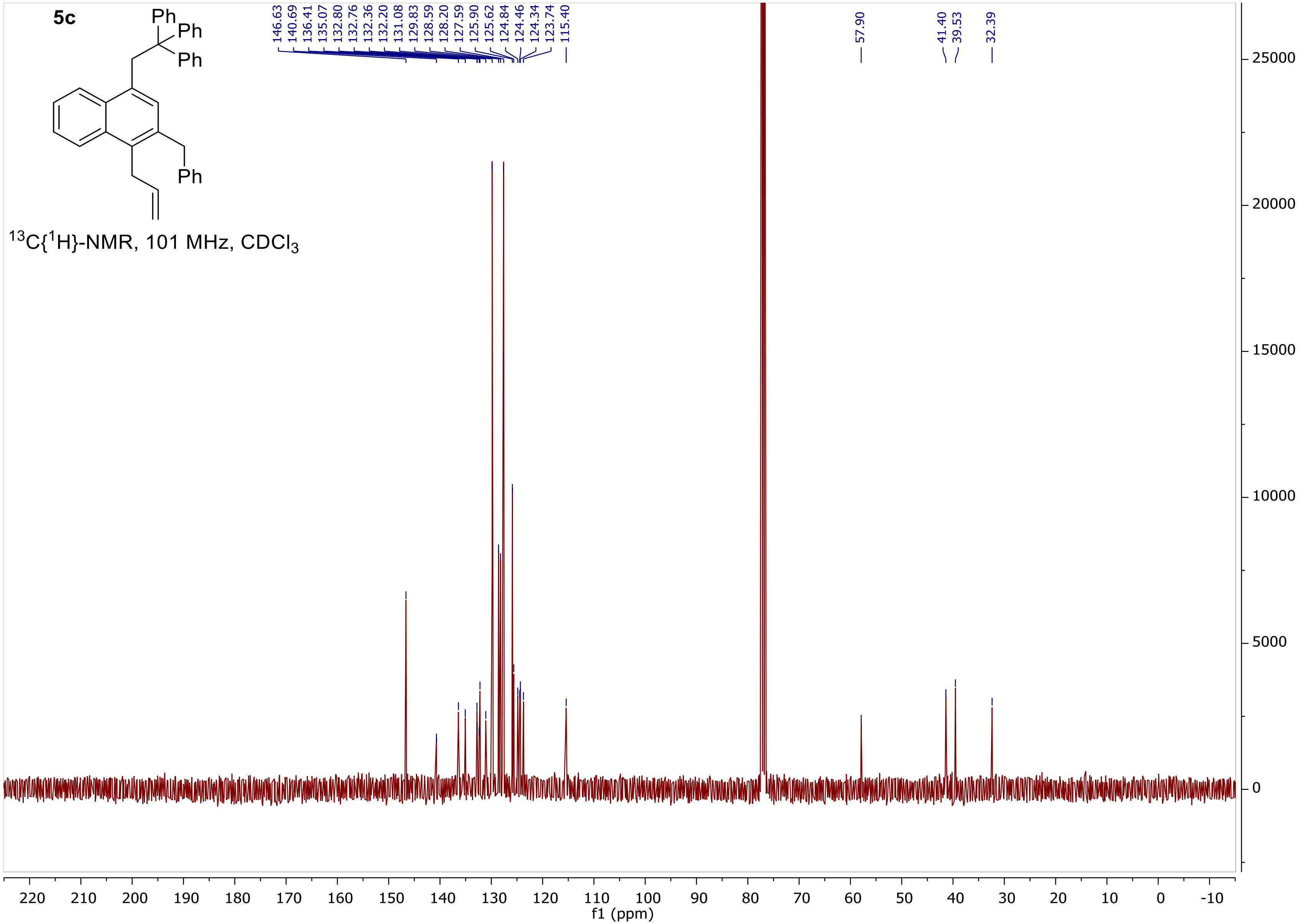

5c
Ph
Ph
Ph
Ph
$^{13}C\{^{1}H\}$-NMR, 101 MHz, CDCl$_3$
146.63
140.69
136.41
135.07
132.80
132.76
132.36
132.20
131.08
129.83
128.59
128.20
127.59
125.90
125.62
124.84
124.46
124.34
123.74
115.40
57.90
41.40
39.53
32.39
25000
20000
15000
10000
5000
0
220 210 200 190 180 170 160 150 140 130 120 110 100 90 80 70 60 50 40 30 20 10 0 -10
f1 (ppm)

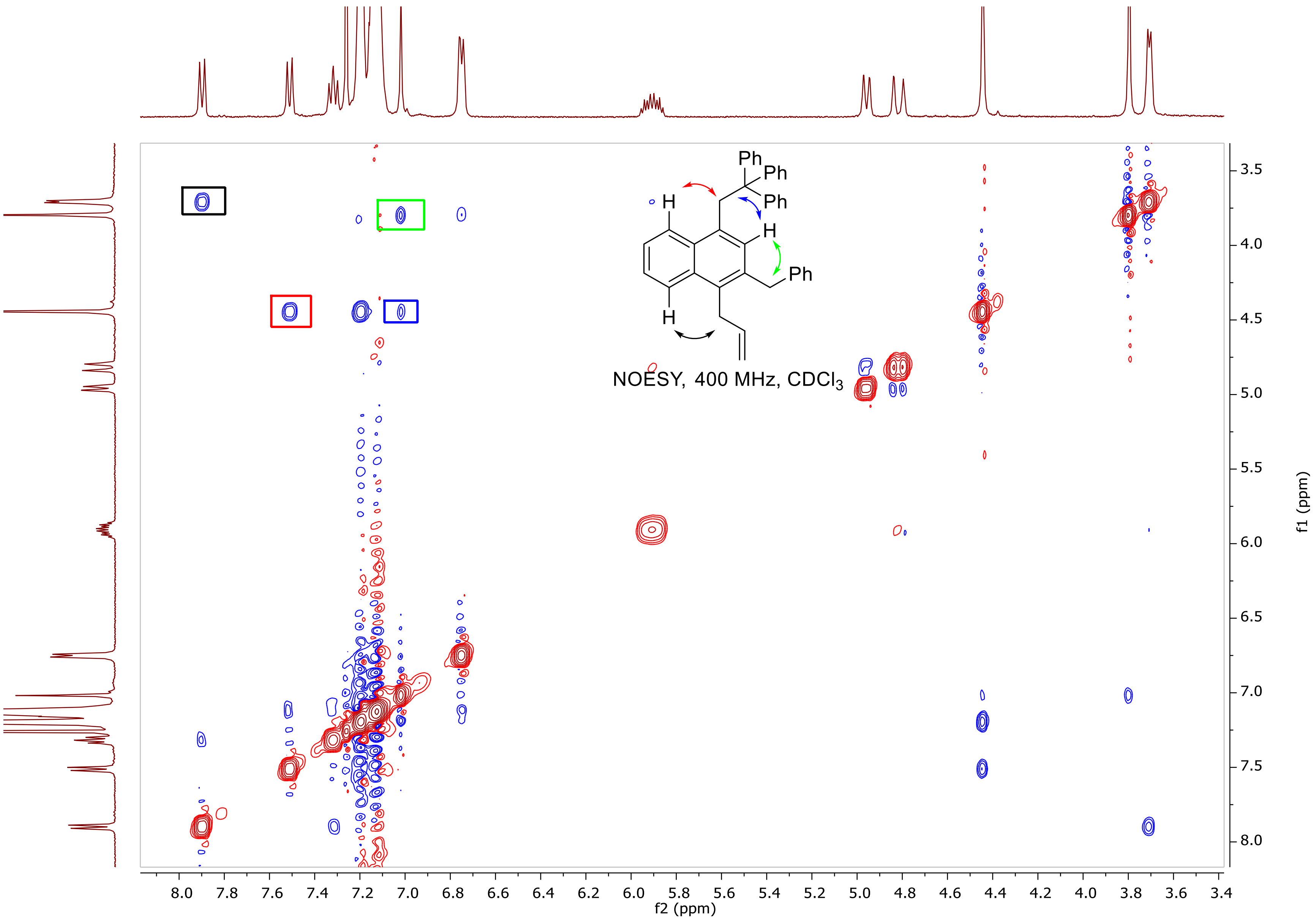

Ph
Ph
Ph
H
H
H
Ph
NOESY, 400 MHz, $CDCl_3$
f2 (ppm)
f1 (ppm)

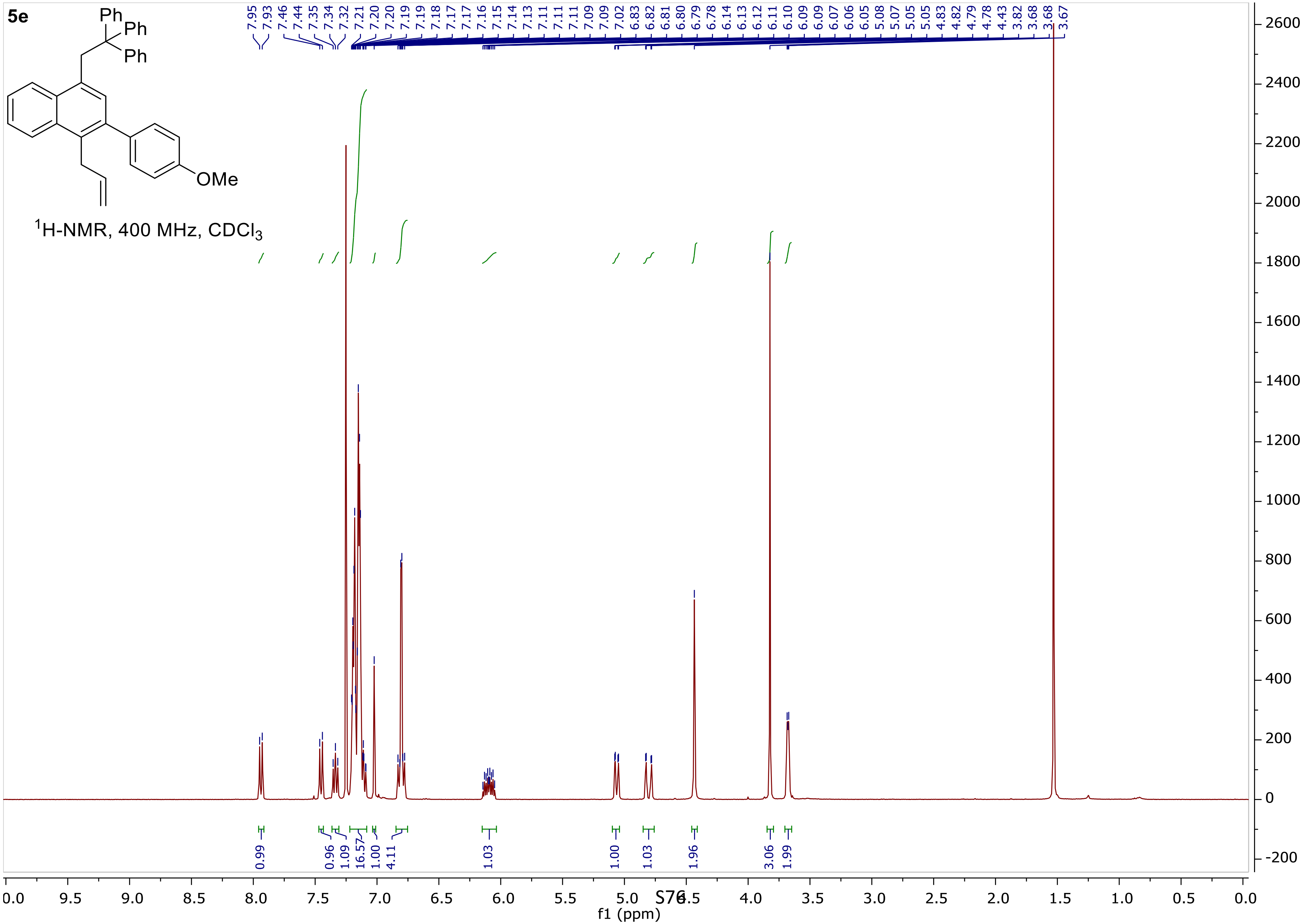
5e
Ph
Ph
Ph
OMe
1H-NMR, 400 MHz, CDCl3
f1 (ppm)

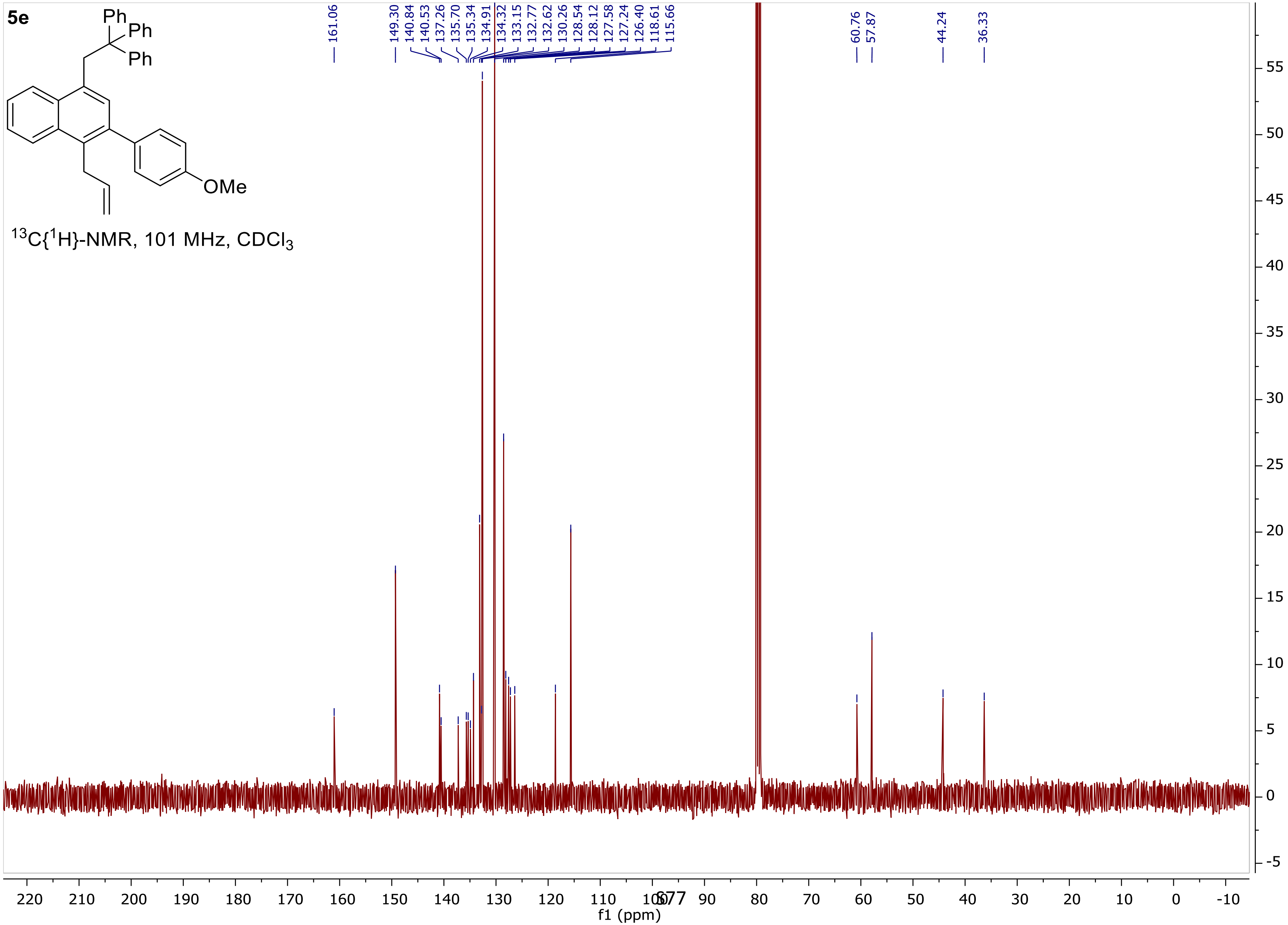
5e
Ph
Ph
Ph
OMe
$^{13}C\{^{1}H\}$-NMR, 101 MHz, $CDCl_3$
161.06
149.30
140.84
140.53
137.26
135.70
135.34
134.91
134.32
133.15
132.77
132.62
130.26
128.54
128.12
127.58
127.24
126.40
118.61
115.66
60.76
57.87
44.24
36.33
f1 (ppm)

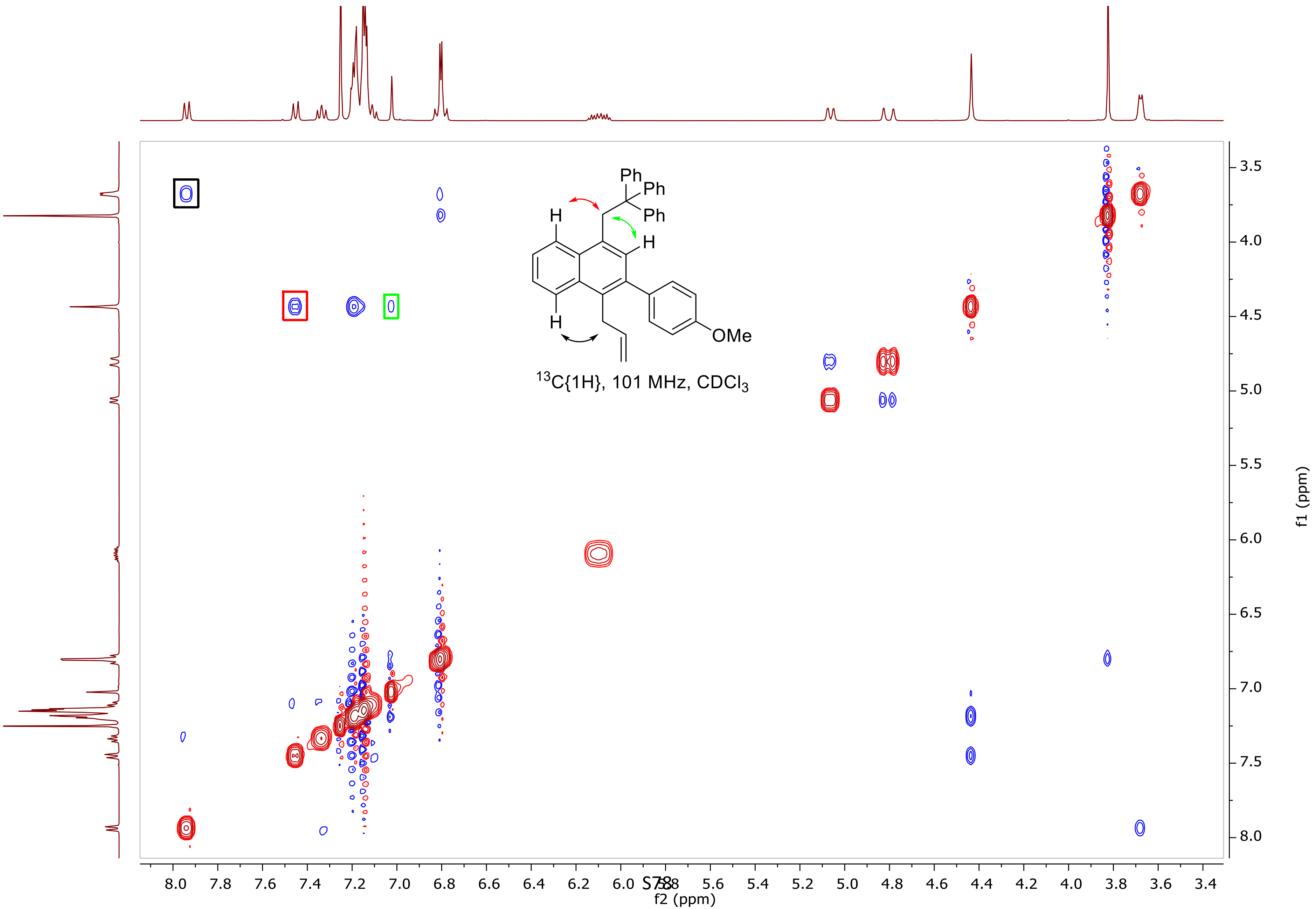
Ph
Ph
Ph
H
H
H
OMe
13C{1H}, 101 MHz, CDCl3
f1 (ppm)
f2 (ppm)

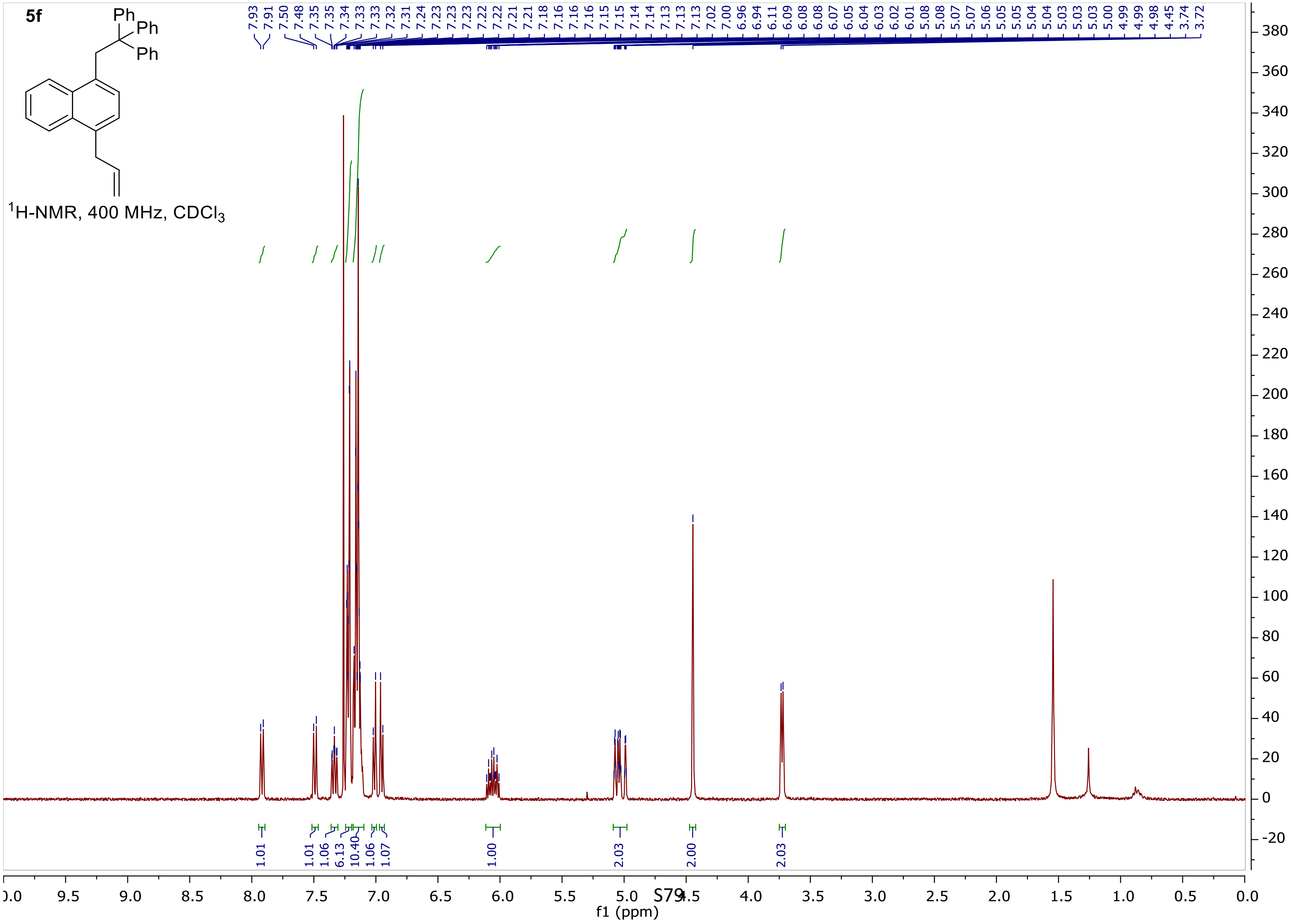

5f
Ph
Ph
Ph
^{1}H-NMR, 400 MHz, CDCl3
7.93 7.91 7.50 7.48 7.35 7.35 7.34 7.33 7.33 7.32 7.31 7.24 7.23 7.23 7.23 7.22 7.22 7.21 7.18 7.16 7.16 7.15 7.15 7.14 7.14 7.13 7.13 7.13 7.02 7.00 6.96 6.94 6.11 6.09 6.08 6.07 6.05 6.04 6.03 6.02 6.01 5.08 5.08 5.07 5.07 5.06 5.05 5.05 5.04 5.04 5.03 5.03 5.03 5.00 4.99 4.99 4.98 4.45 3.74 3.72
1.01 1.01 1.06 6.13 10.40 1.06 1.07 1.00 2.03 2.00 2.03
f1 (ppm)

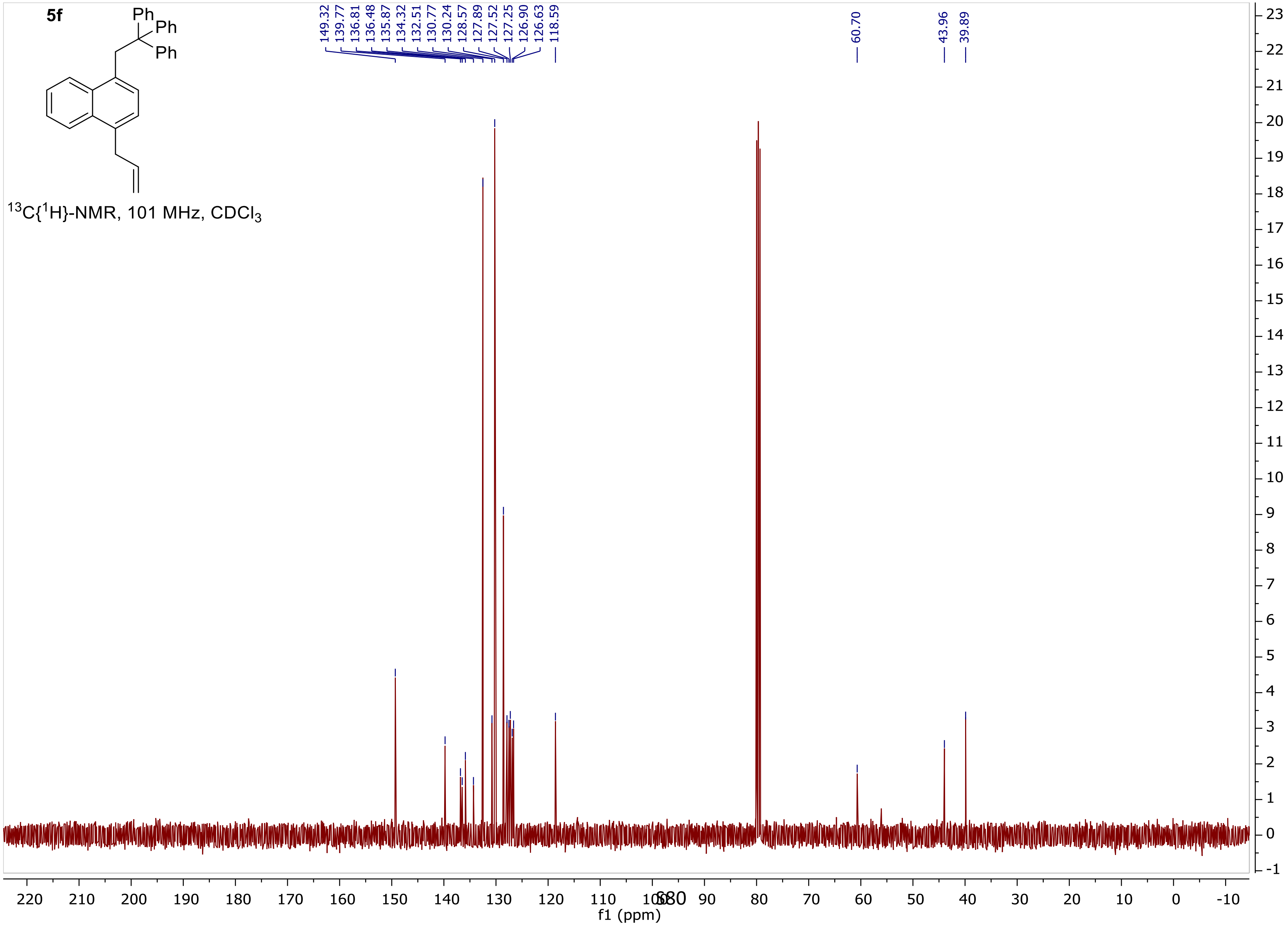
5f
Ph
Ph
Ph
13C{1H}-NMR, 101 MHz, CDCl3
149.32
139.77
136.81
136.48
135.87
134.32
132.51
130.77
130.24
128.57
127.89
127.52
127.25
126.90
126.63
118.59
60.70
43.96
39.89
f1 (ppm)

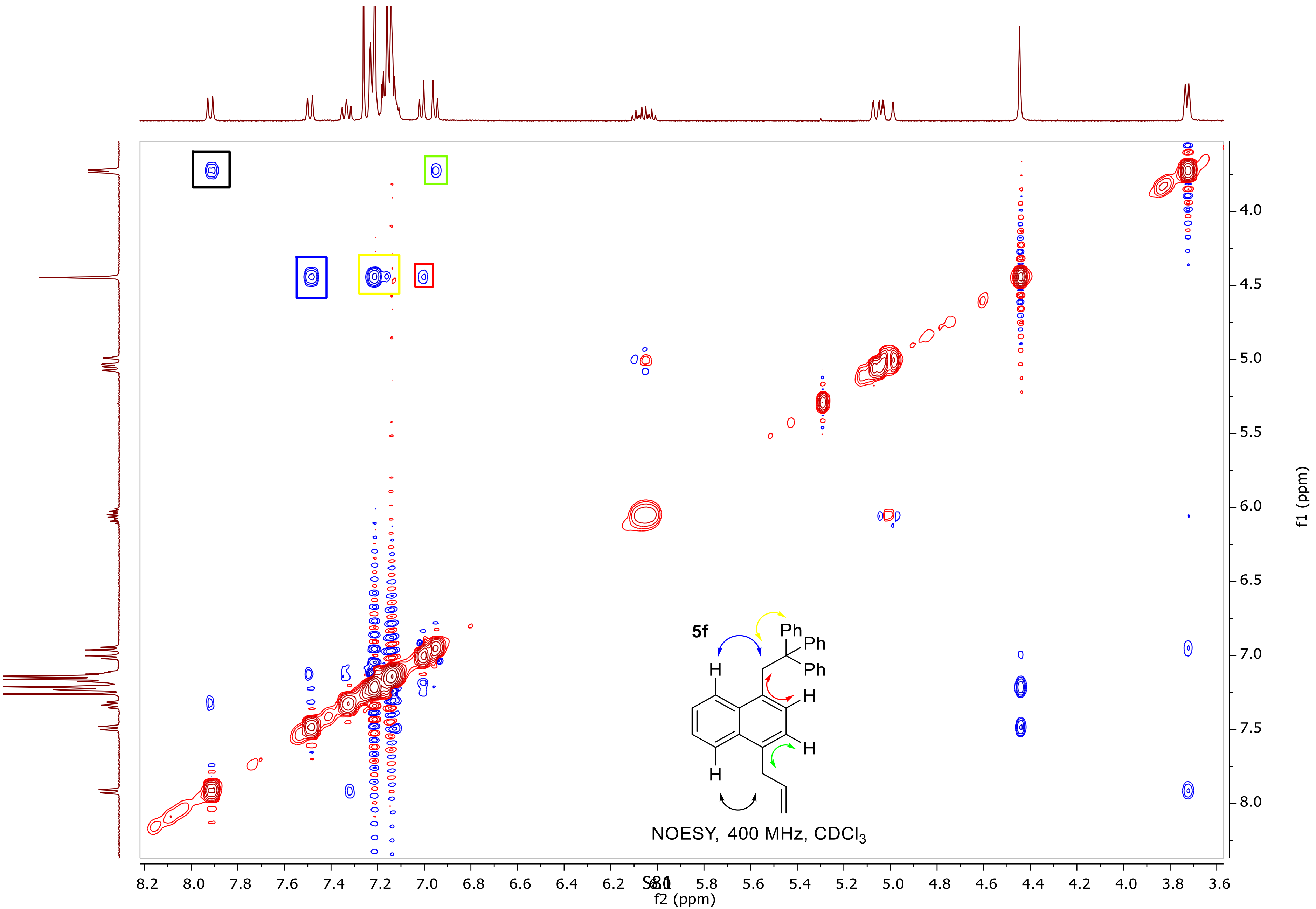
5f
Ph
Ph
Ph
H
H
H
H
NOESY, 400 MHz, $CDCl_3$
f2 (ppm)
f1 (ppm)

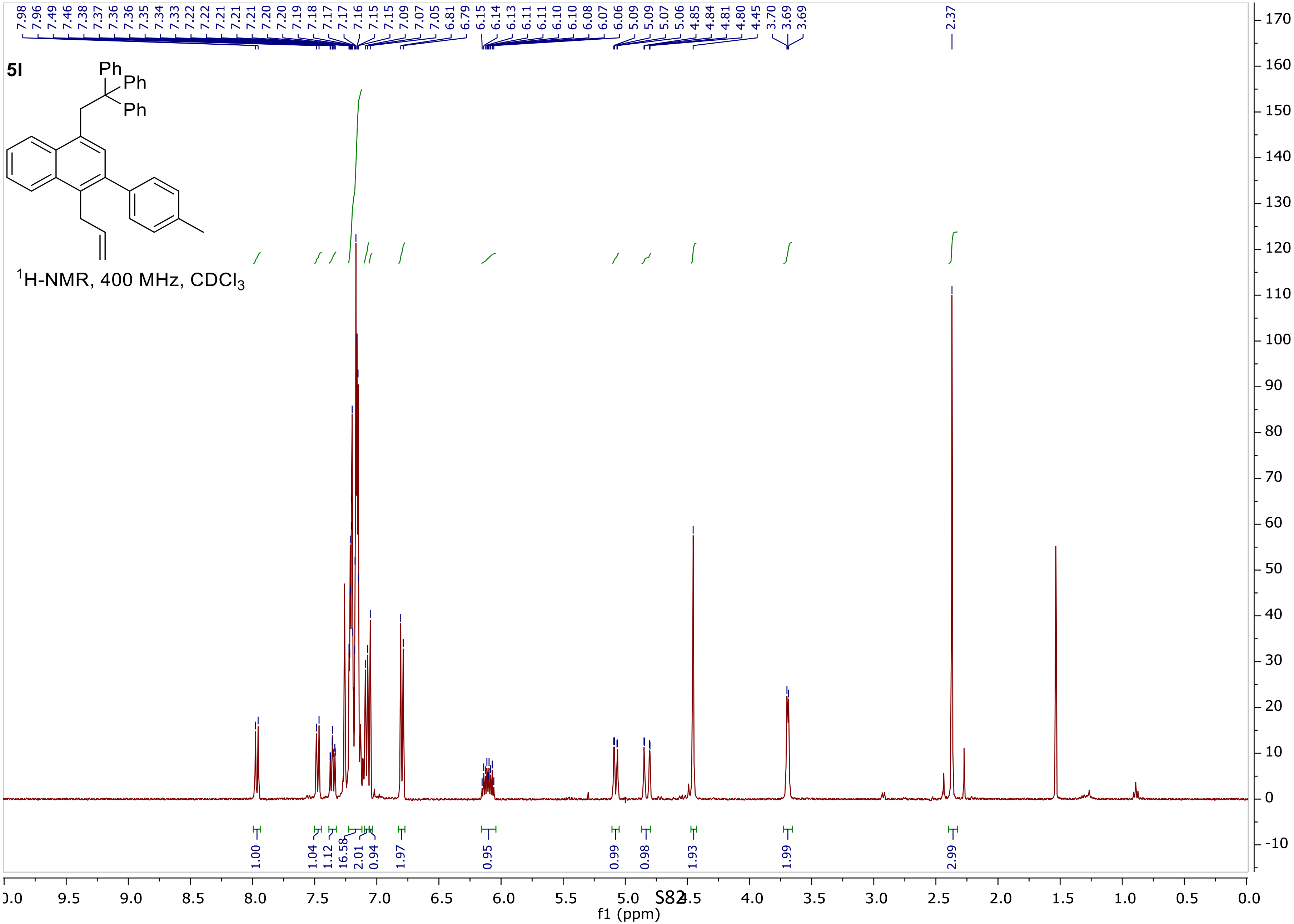
5l
Ph
Ph
Ph
1H-NMR, 400 MHz, CDCl3
f1 (ppm)

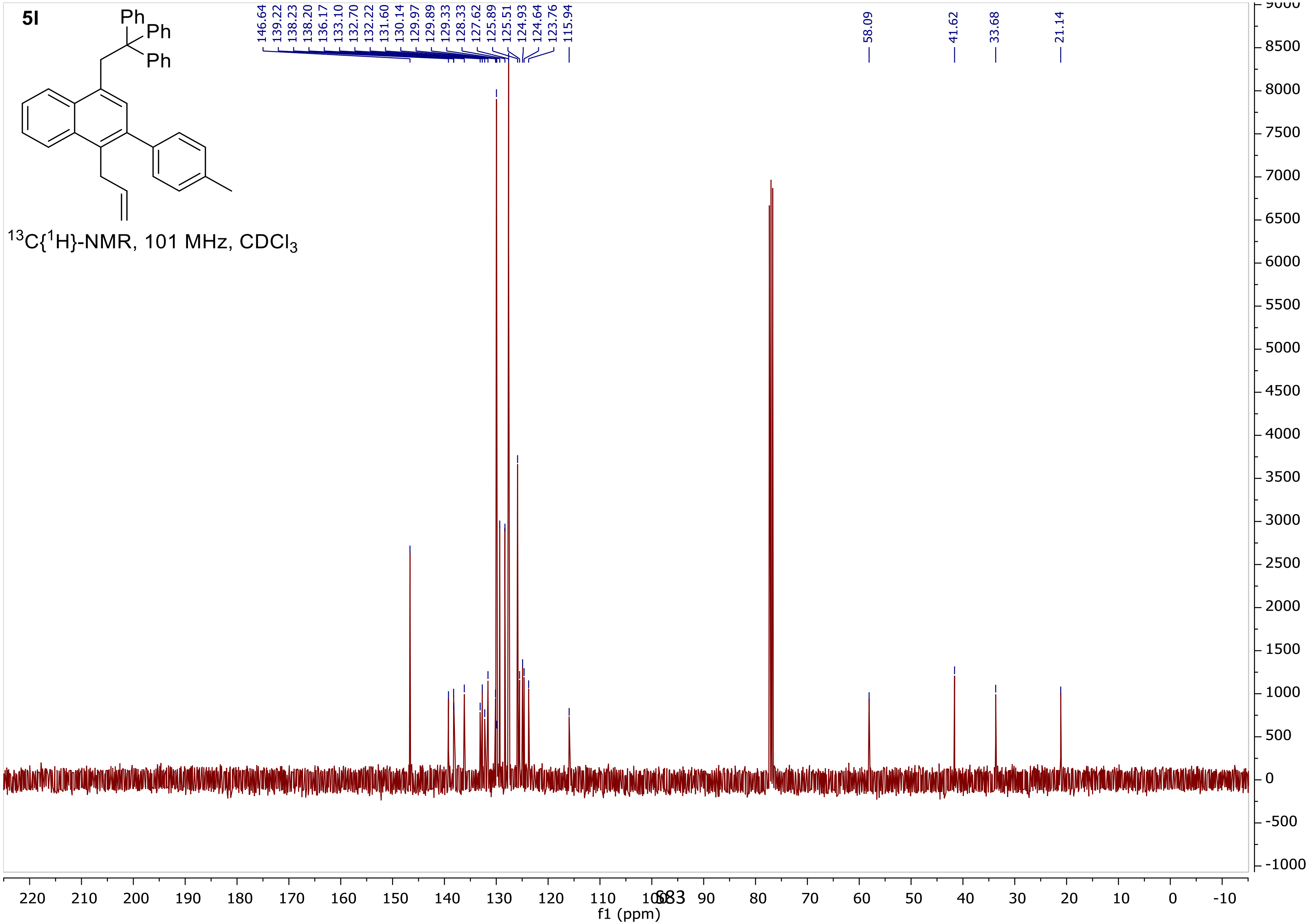

5l
Ph
Ph
Ph
$^{13}C\{^1H\}$-NMR, 101 MHz, $CDCl_3$
146.64
139.22
138.23
138.20
136.17
133.10
132.70
132.22
131.60
130.14
129.97
129.89
129.33
128.33
127.62
125.89
125.51
124.93
124.64
123.76
115.94
58.09
41.62
33.68
21.14
f1 (ppm)

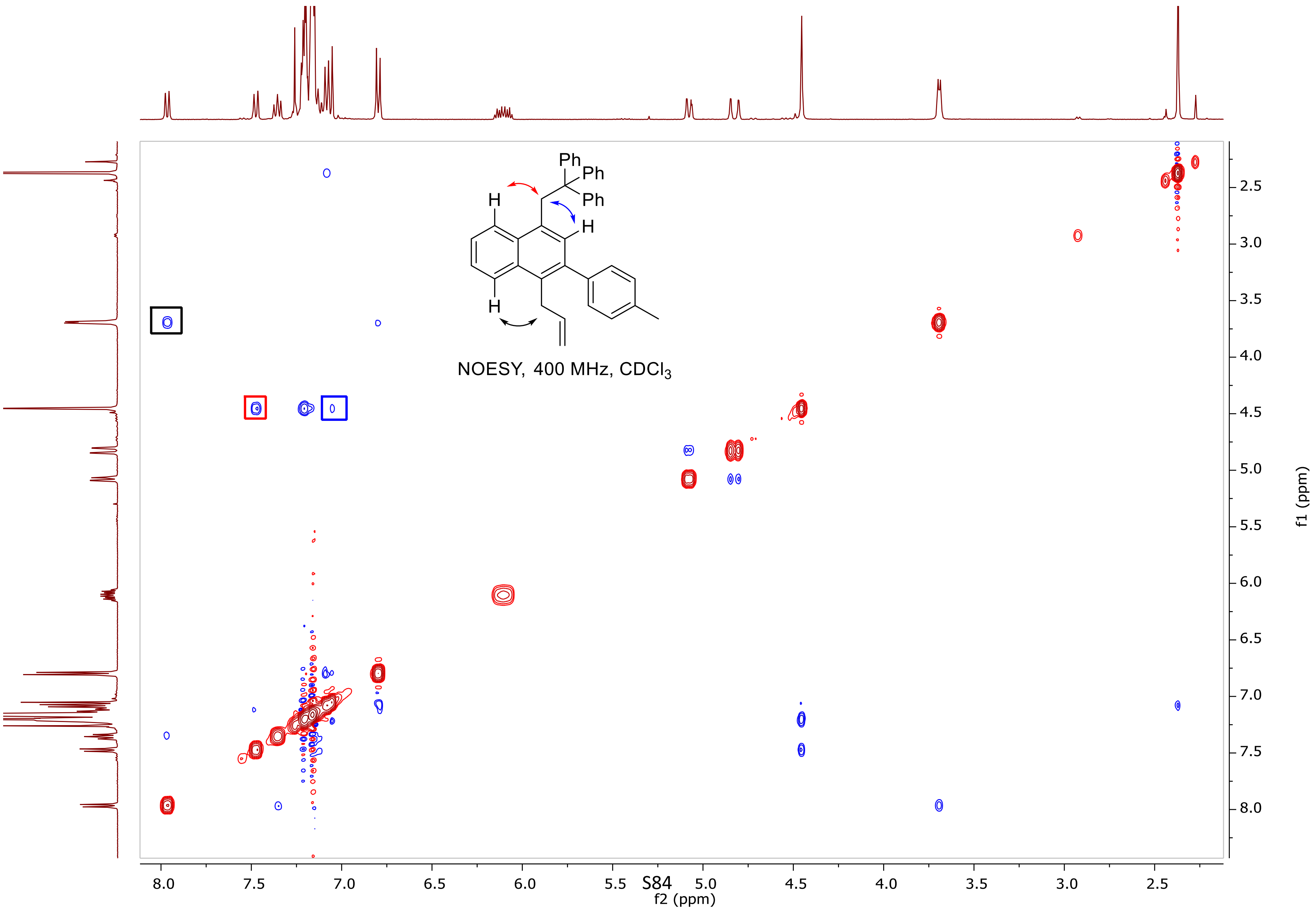

Ph
Ph
Ph
H
H
H
NOESY, 400 MHz, $CDCl_3$
f1 (ppm)
f2 (ppm)

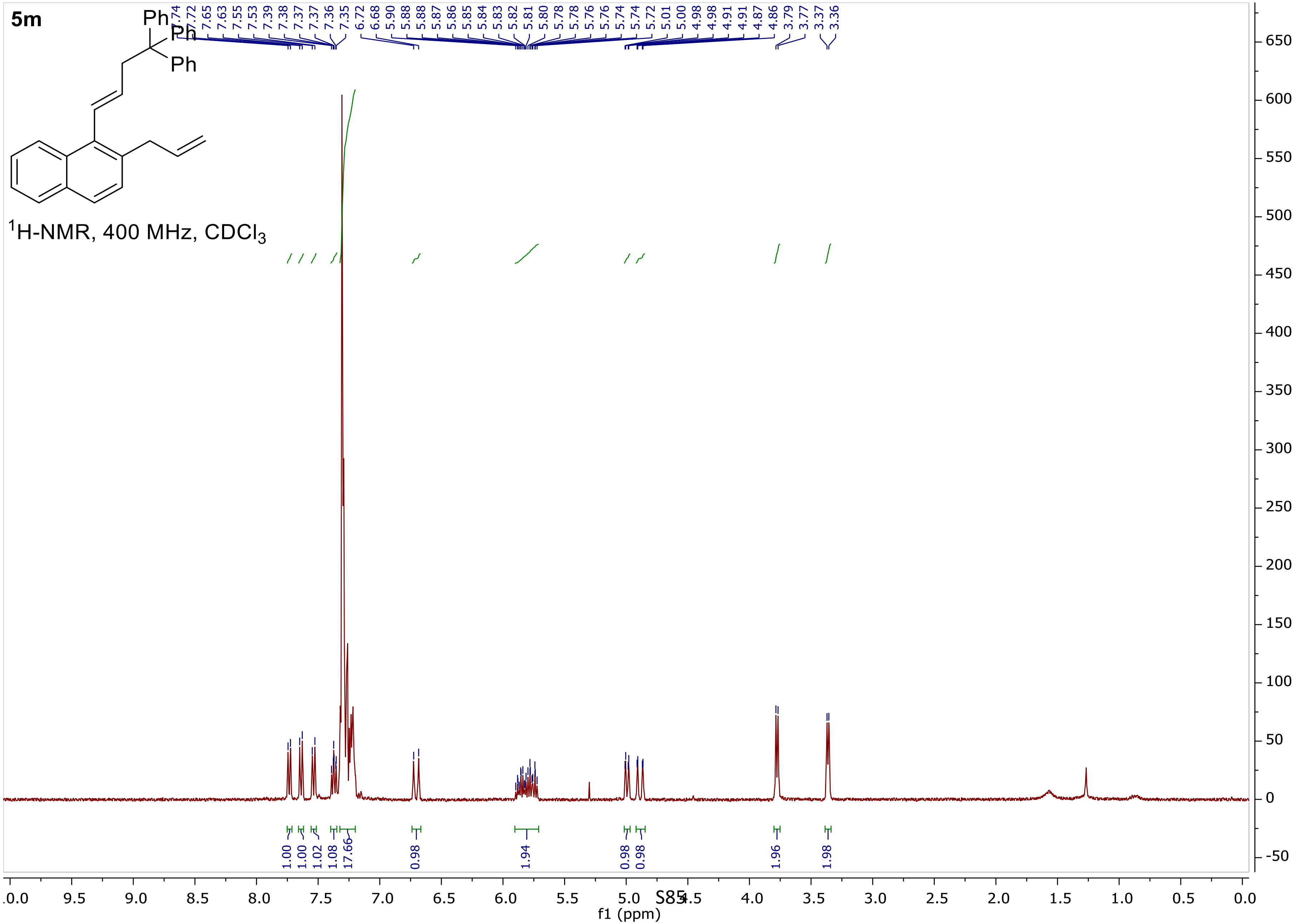

5m
Ph
Ph
Ph
¹H-NMR, 400 MHz, CDCl3
7.74 7.72 7.65 7.63 7.55 7.53 7.39 7.38 7.37 7.37 7.36 7.35 6.72 6.68 5.90 5.88 5.88 5.87 5.86 5.85 5.84 5.83 5.82 5.81 5.80 5.78 5.78 5.76 5.76 5.74 5.74 5.72 5.01 5.00 4.98 4.98 4.91 4.87 4.86 3.79 3.77 3.37 3.36
1.00 1.00 1.02 1.08 17.66 0.98 1.94 0.98 0.98 1.96 1.98
f1 (ppm)

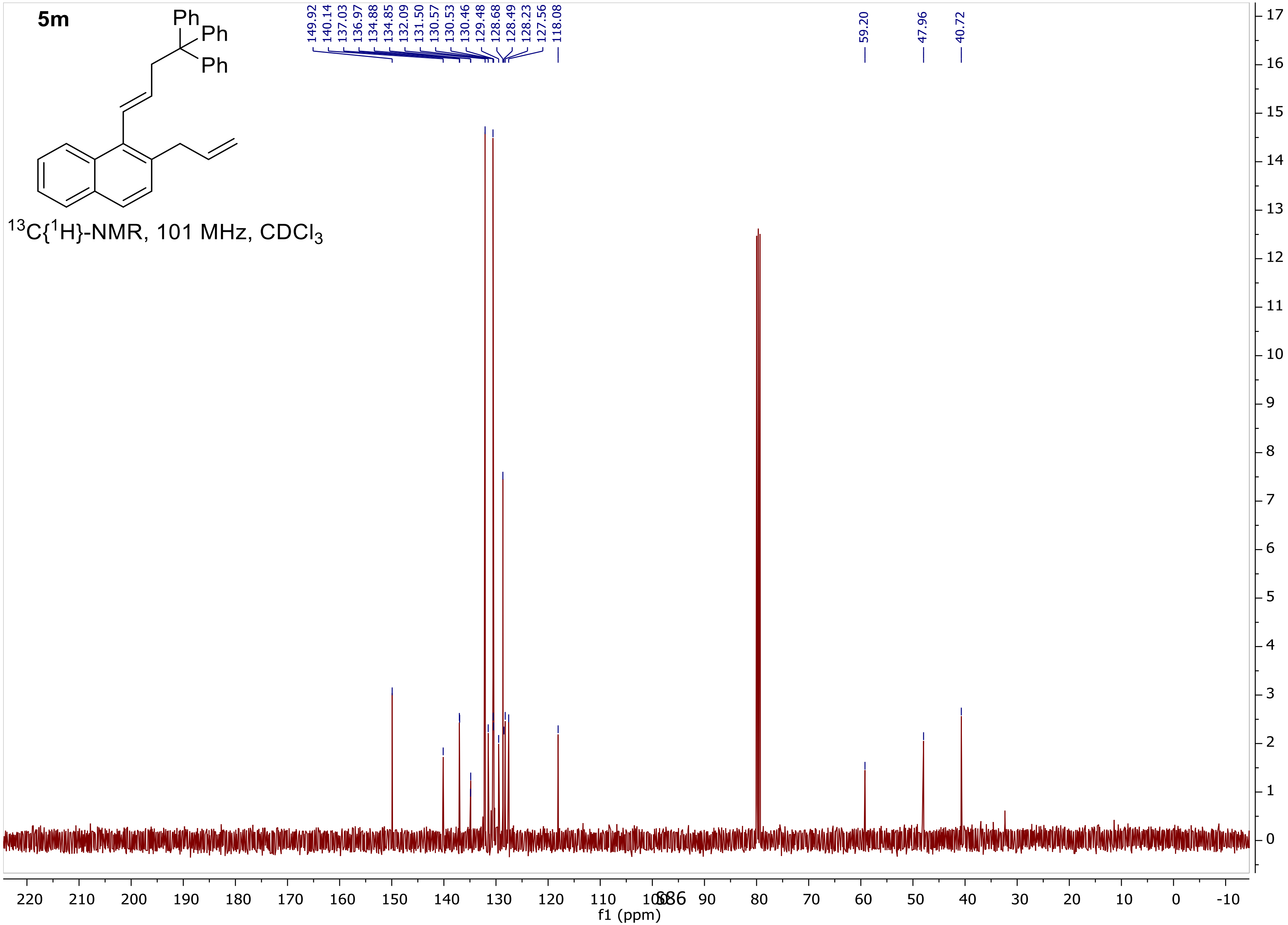
5m
Ph
Ph
Ph
13C{1H}-NMR, 101 MHz, CDCl3
149.92
140.14
137.03
136.97
134.88
134.85
132.09
131.50
130.57
130.53
130.46
129.48
128.68
128.49
128.23
127.56
118.08
59.20
47.96
40.72
f1 (ppm)

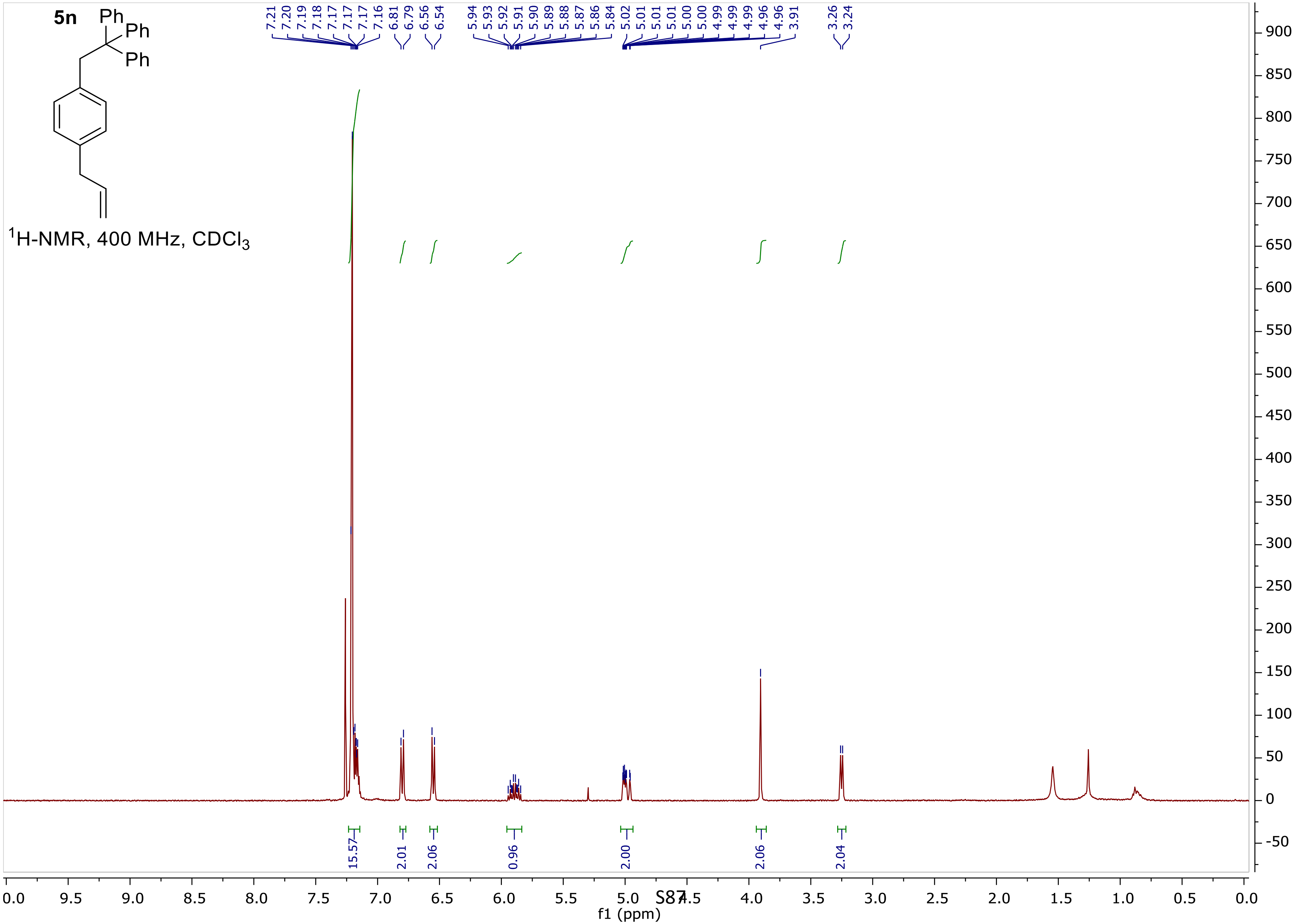
5n
Ph
Ph
Ph
1H-NMR, 400 MHz, CDCl3
7.21
7.20
7.19
7.18
7.17
7.17
7.17
7.16
6.81
6.79
6.56
6.54
5.94
5.93
5.92
5.91
5.90
5.89
5.88
5.87
5.86
5.84
5.02
5.01
5.01
5.01
5.00
5.00
4.99
4.99
4.99
4.96
4.96
3.91
3.26
3.24
15.57
2.01
2.06
0.96
2.00
2.06
2.04
f1 (ppm)

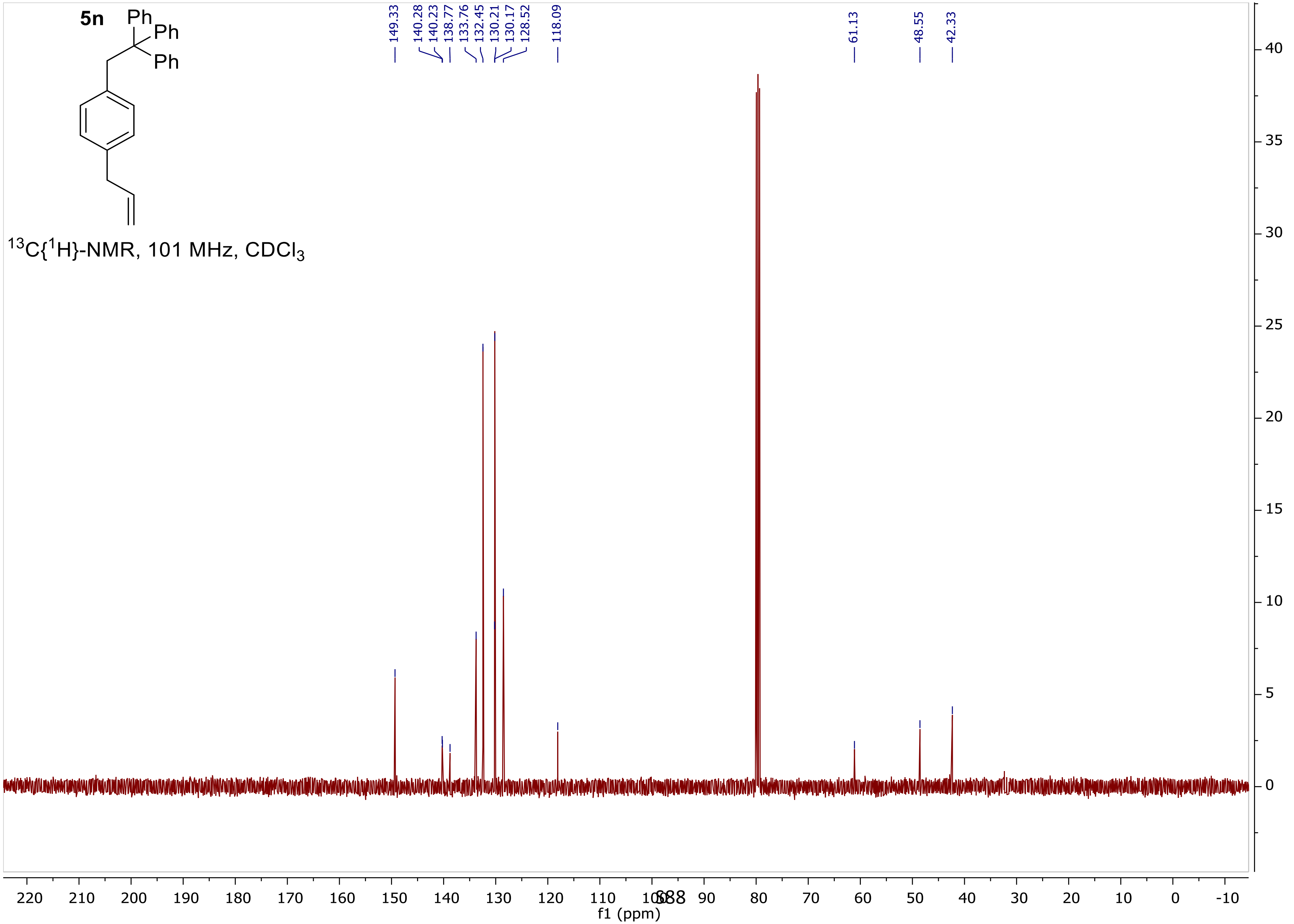
5n
Ph
Ph
Ph
$^{13}C\{^1H\}$-NMR, 101 MHz, $CDCl_3$
149.33
140.28
140.23
138.77
133.76
132.45
130.21
130.17
128.52
118.09
61.13
48.55
42.33
f1 (ppm)

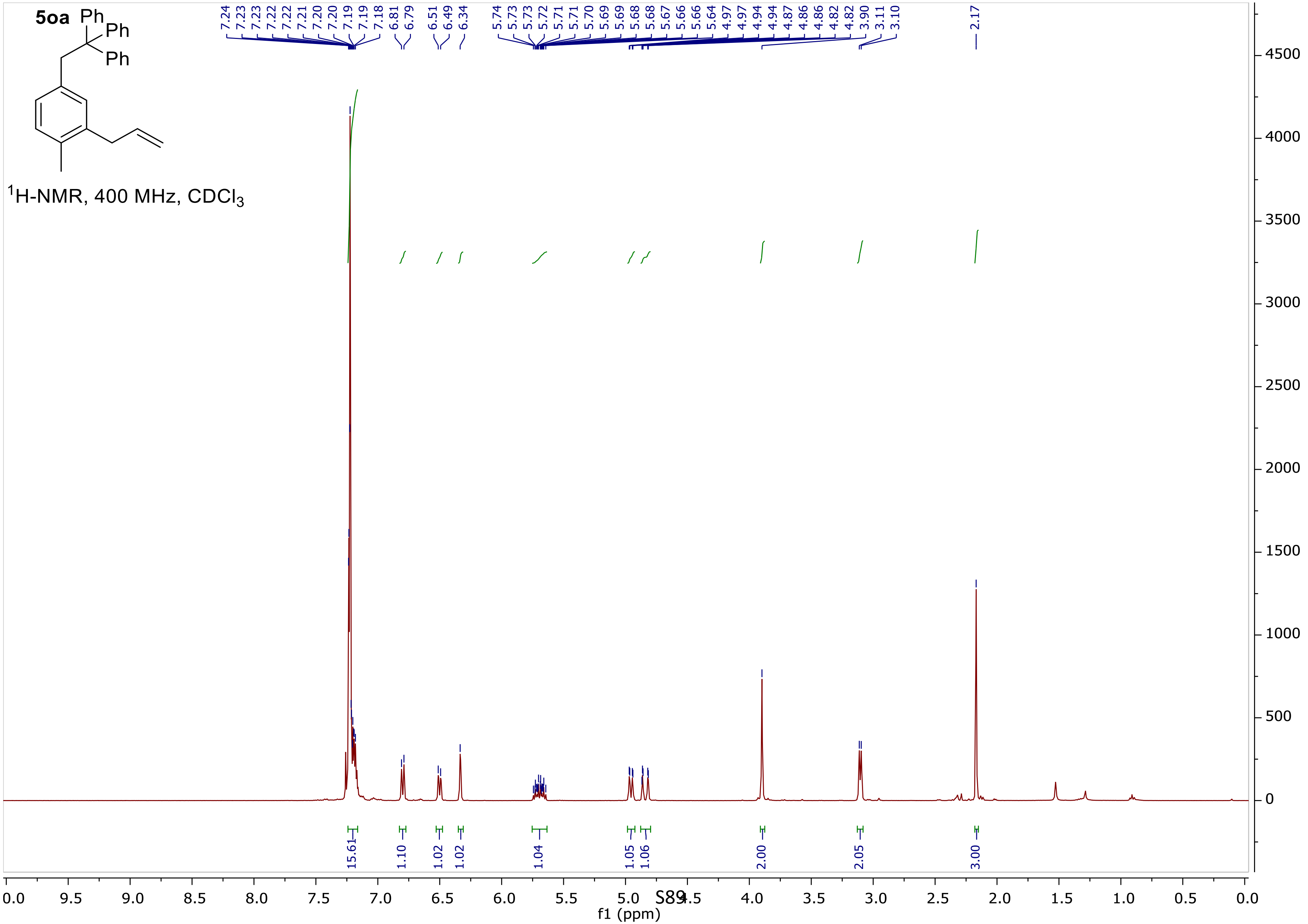
5oa
Ph
Ph
Ph
1H-NMR, 400 MHz, CDCl3
7.24 7.23 7.23 7.22 7.22 7.21 7.20 7.20 7.19 7.19 7.18 6.81 6.79 6.51 6.49 6.34 5.74 5.73 5.73 5.72 5.71 5.71 5.70 5.69 5.69 5.68 5.68 5.68 5.67 5.66 5.66 5.64 4.97 4.94 4.94 4.87 4.86 4.86 4.82 4.82 3.90 3.11 3.10 2.17
15.61 1.10 1.02 1.02 1.04 1.05 1.06 2.00 2.05 3.00
f1 (ppm)

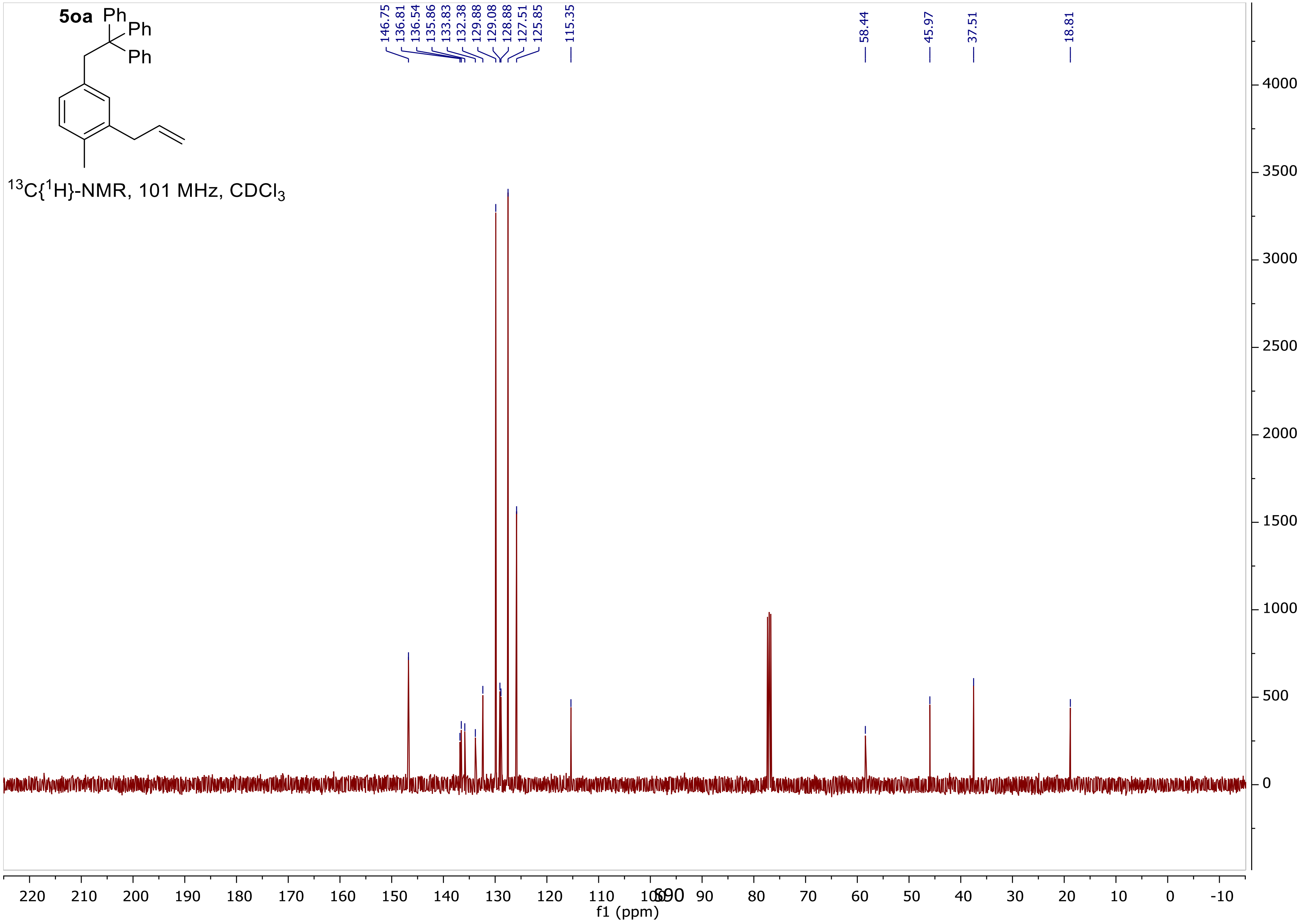
5oa
Ph
Ph
Ph
$^{13}C\{^{1}H\}$-NMR, 101 MHz, $CDCl_3$
146.75
136.81
136.54
135.86
133.83
132.38
129.88
129.08
128.88
127.51
125.85
115.35
58.44
45.97
37.51
18.81
f1 (ppm)

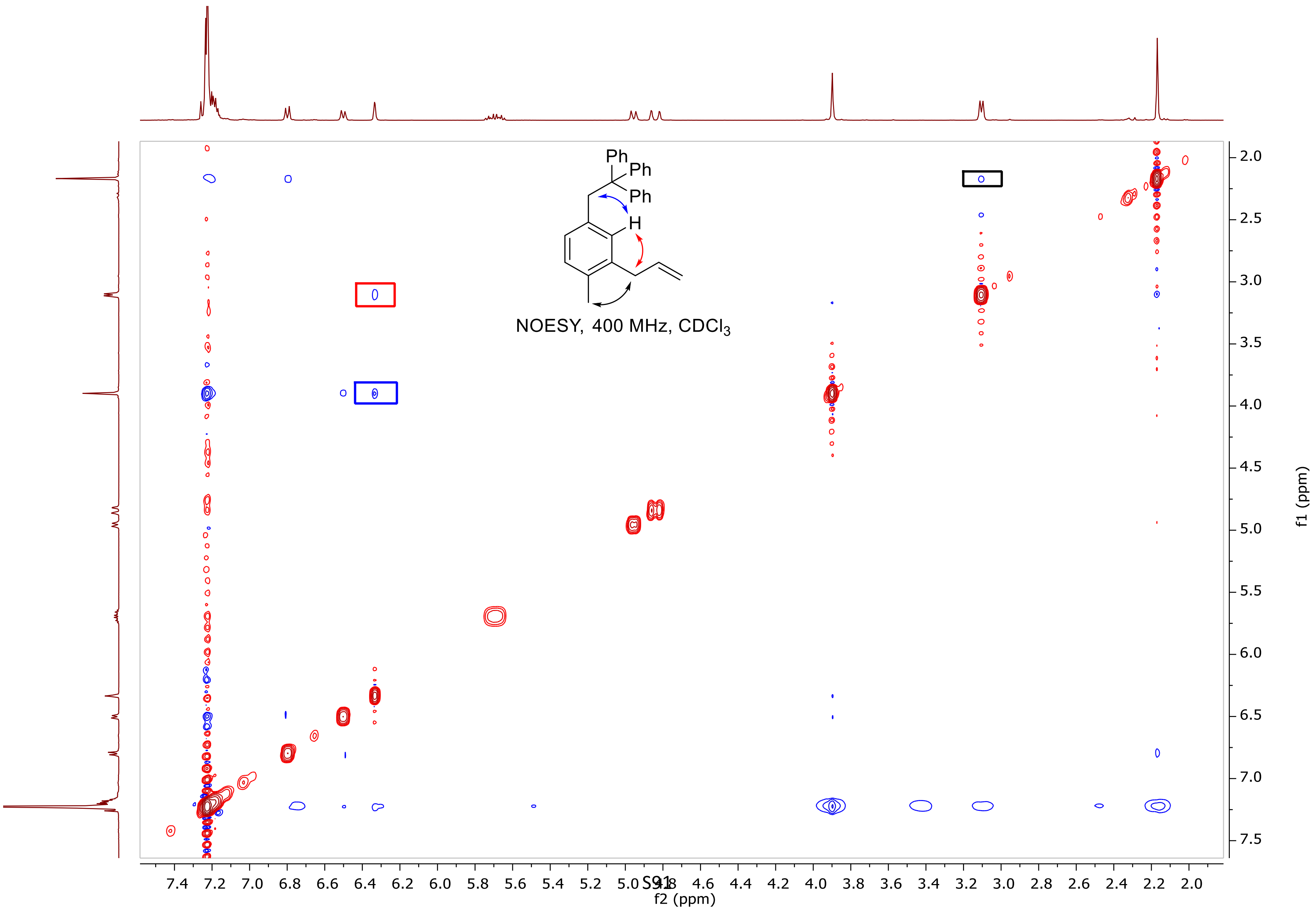
Ph
Ph
Ph
H
NOESY, 400 MHz, CDCl3
f2 (ppm)
f1 (ppm)

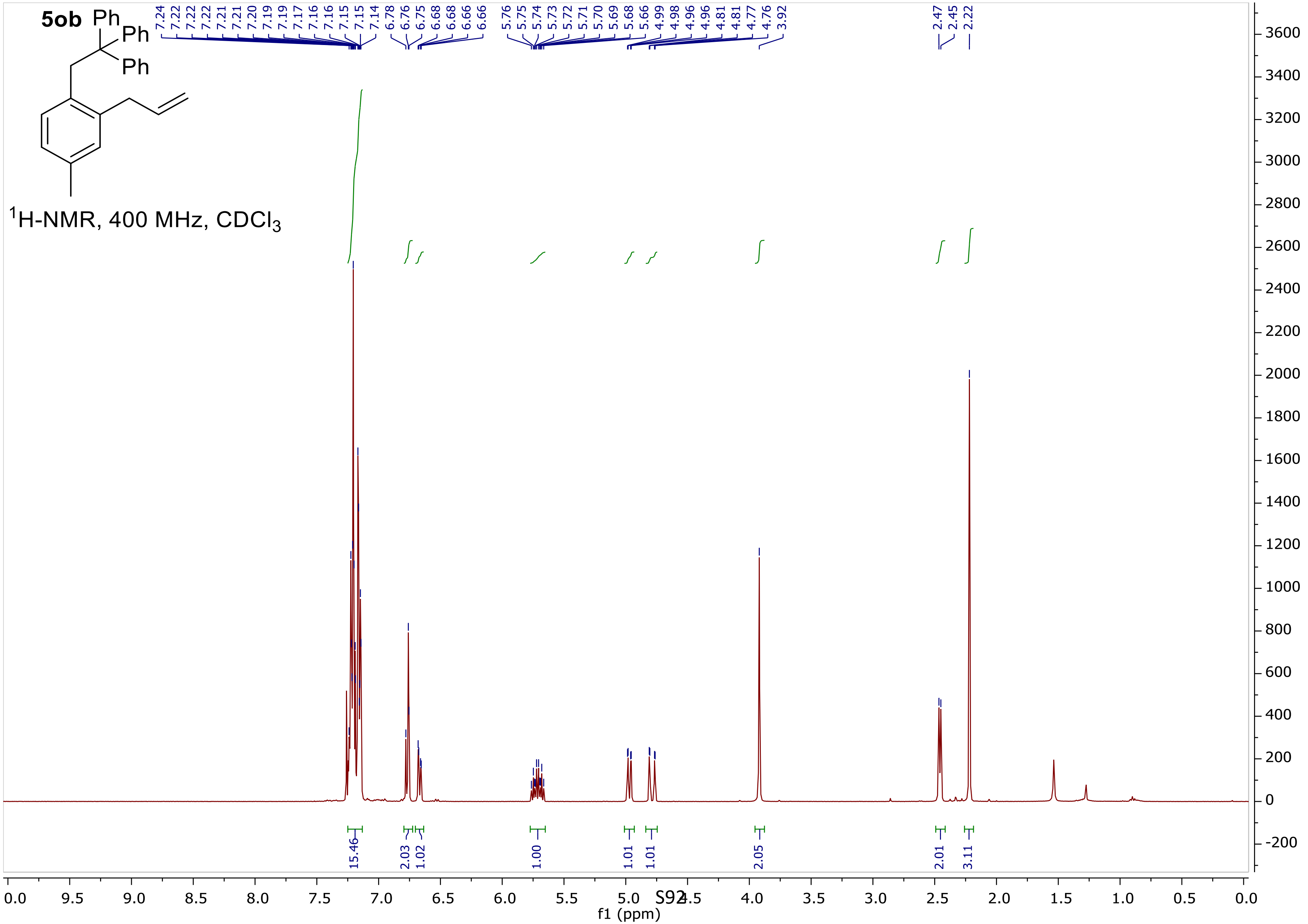

5ob
Ph
Ph
Ph
^{1}H-NMR, 400 MHz, CDCl$_3$
f1 (ppm)

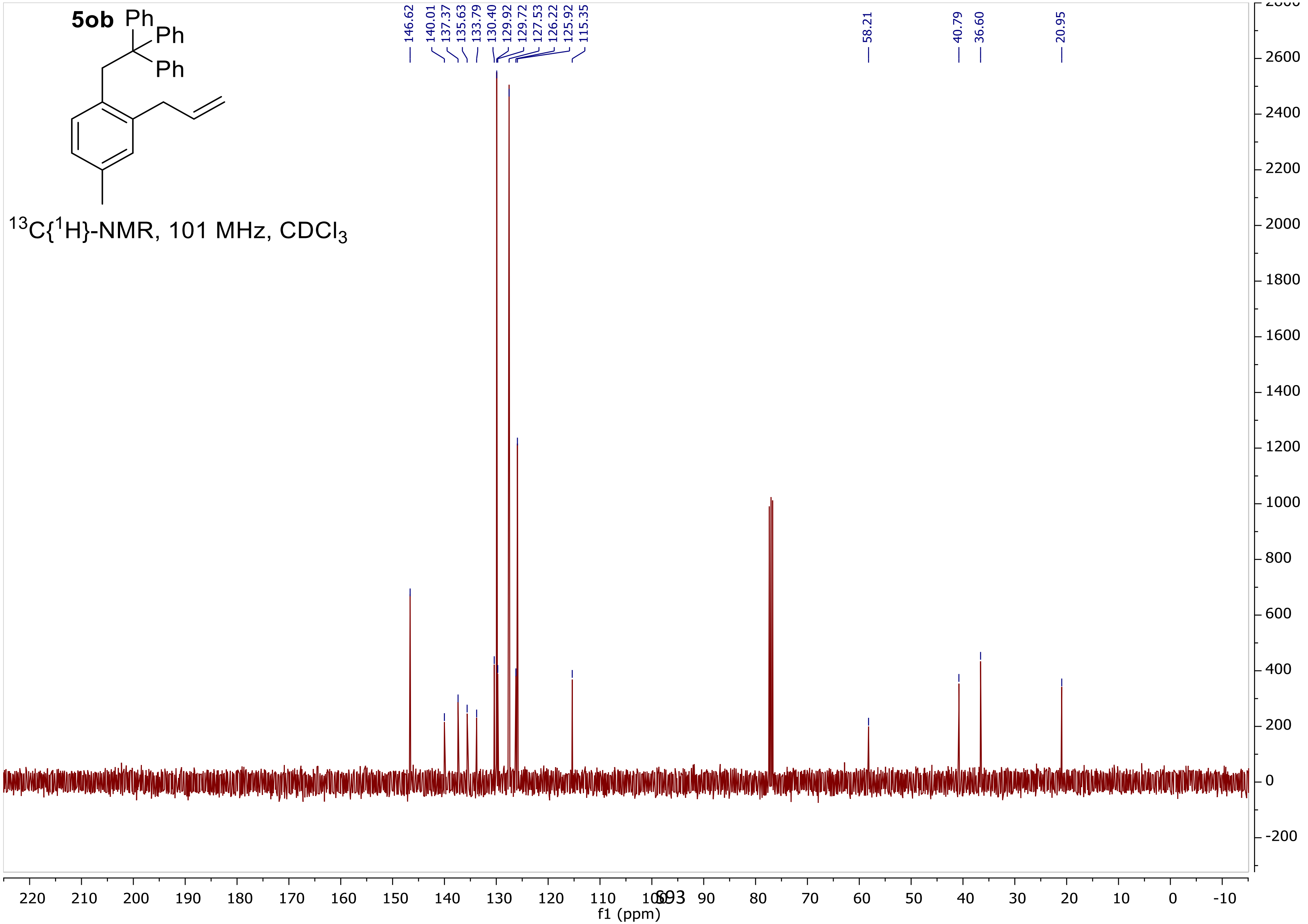

5ob
Ph
Ph
Ph
13C{1H}-NMR, 101 MHz, CDCl3
146.62
140.01
137.37
135.63
133.79
130.40
129.92
129.72
127.53
126.22
125.92
115.35
58.21
40.79
36.60
20.95
f1 (ppm)

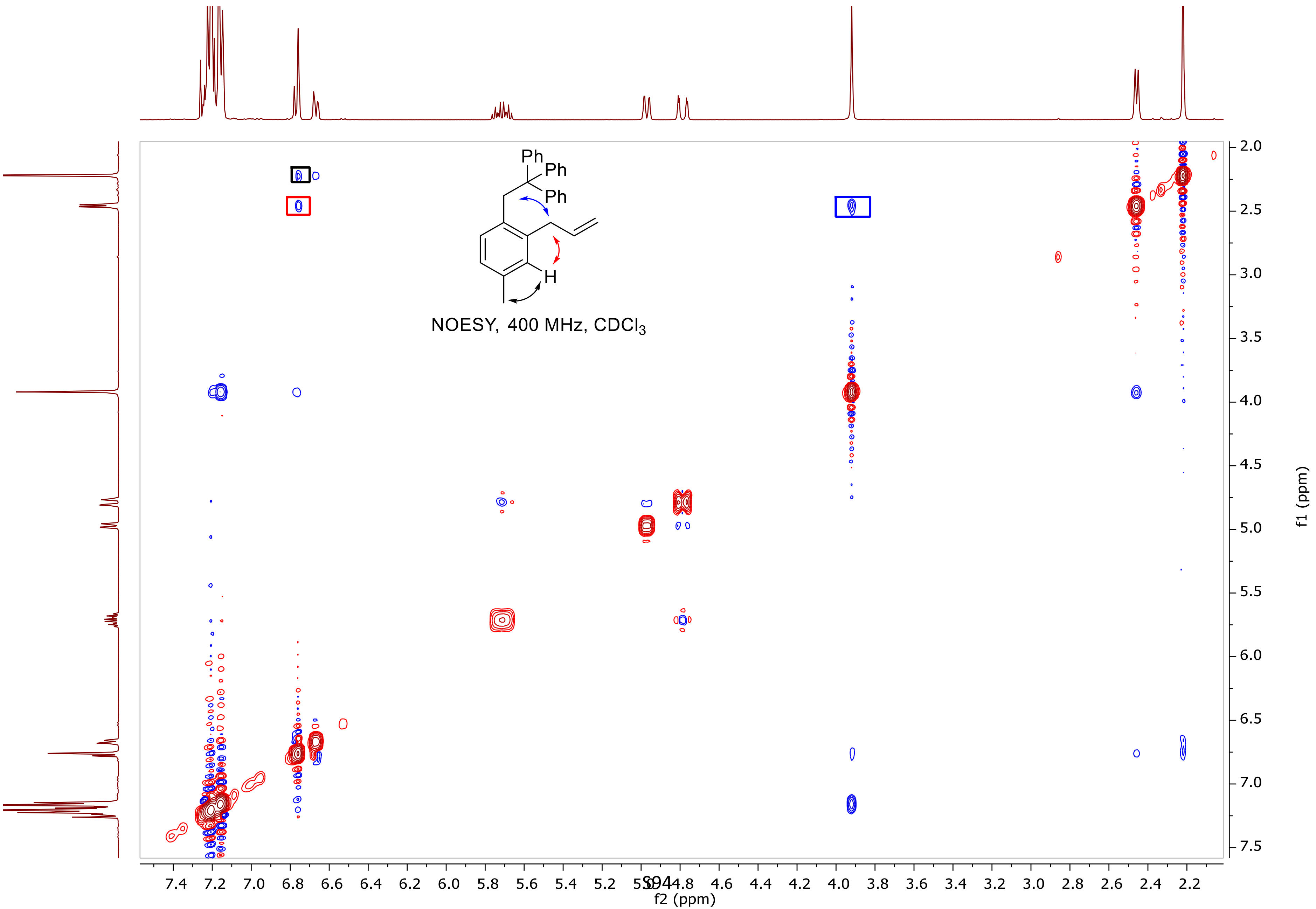
Ph
Ph
Ph
H
NOESY, 400 MHz, CDCl3
f2 (ppm)
f1 (ppm)

7a

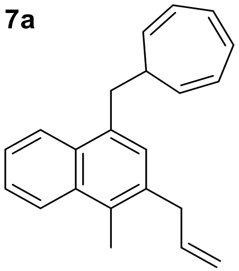

^{1}H-NMR, 400 MHz, $CDCl_3$

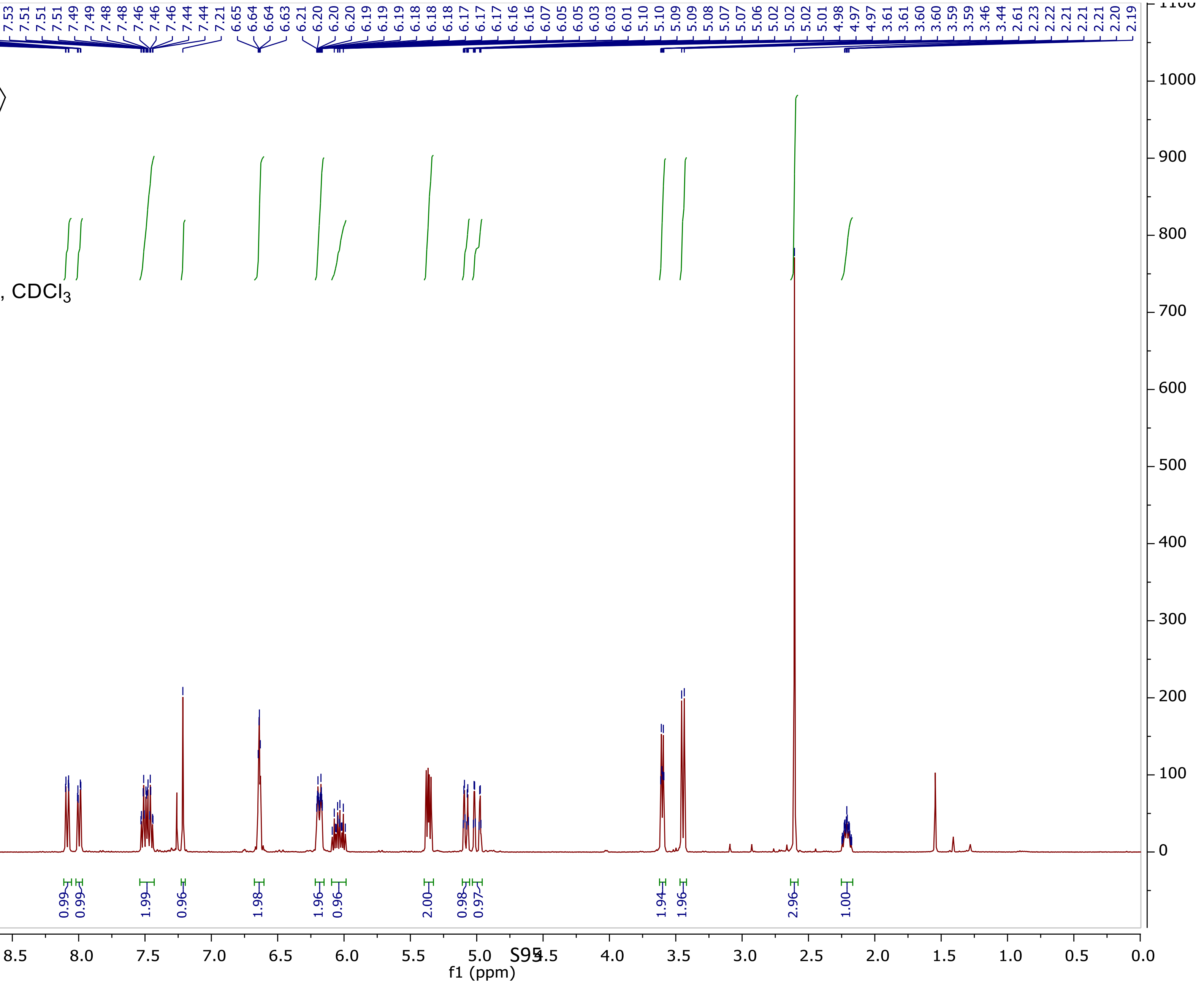

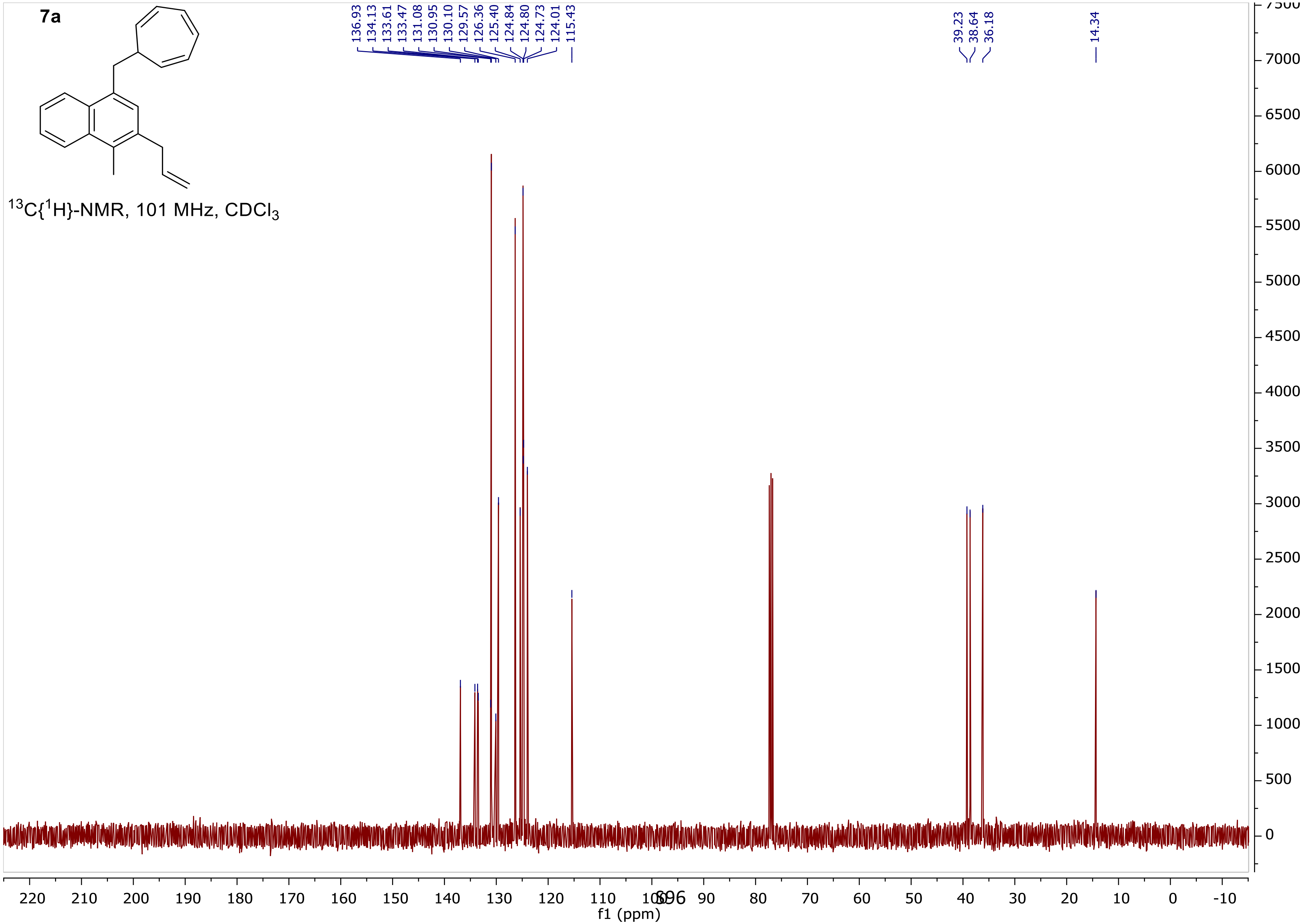
7a
13C{1H}-NMR, 101 MHz, CDCl3
136.93
134.13
133.61
133.47
131.08
130.95
130.10
129.57
126.36
125.40
124.84
124.80
124.73
124.01
115.43
39.23
38.64
36.18
14.34
f1 (ppm)

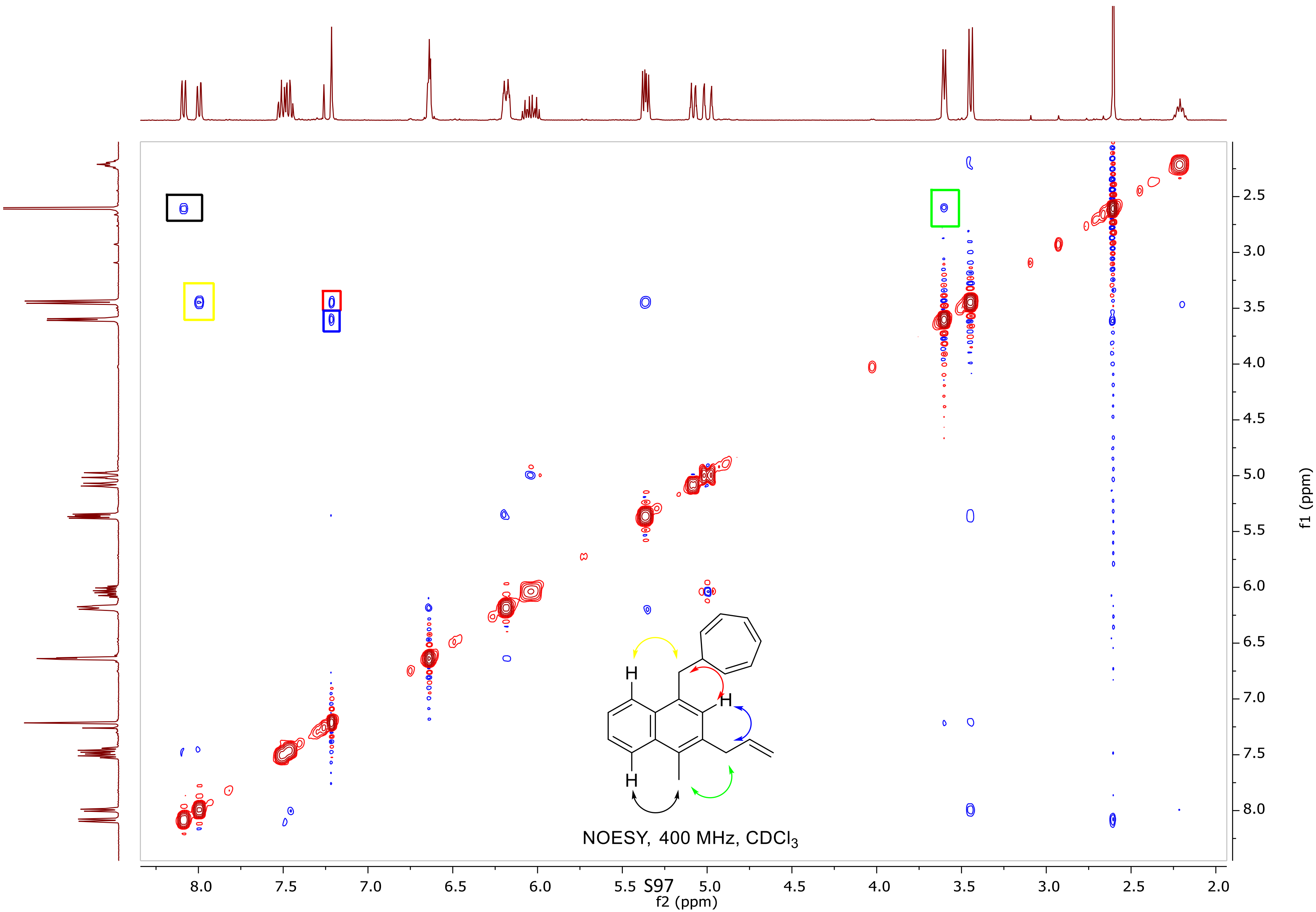

NOESY, 400 MHz, CDCl$_3$
f2 (ppm)
f1 (ppm)
H
H
H

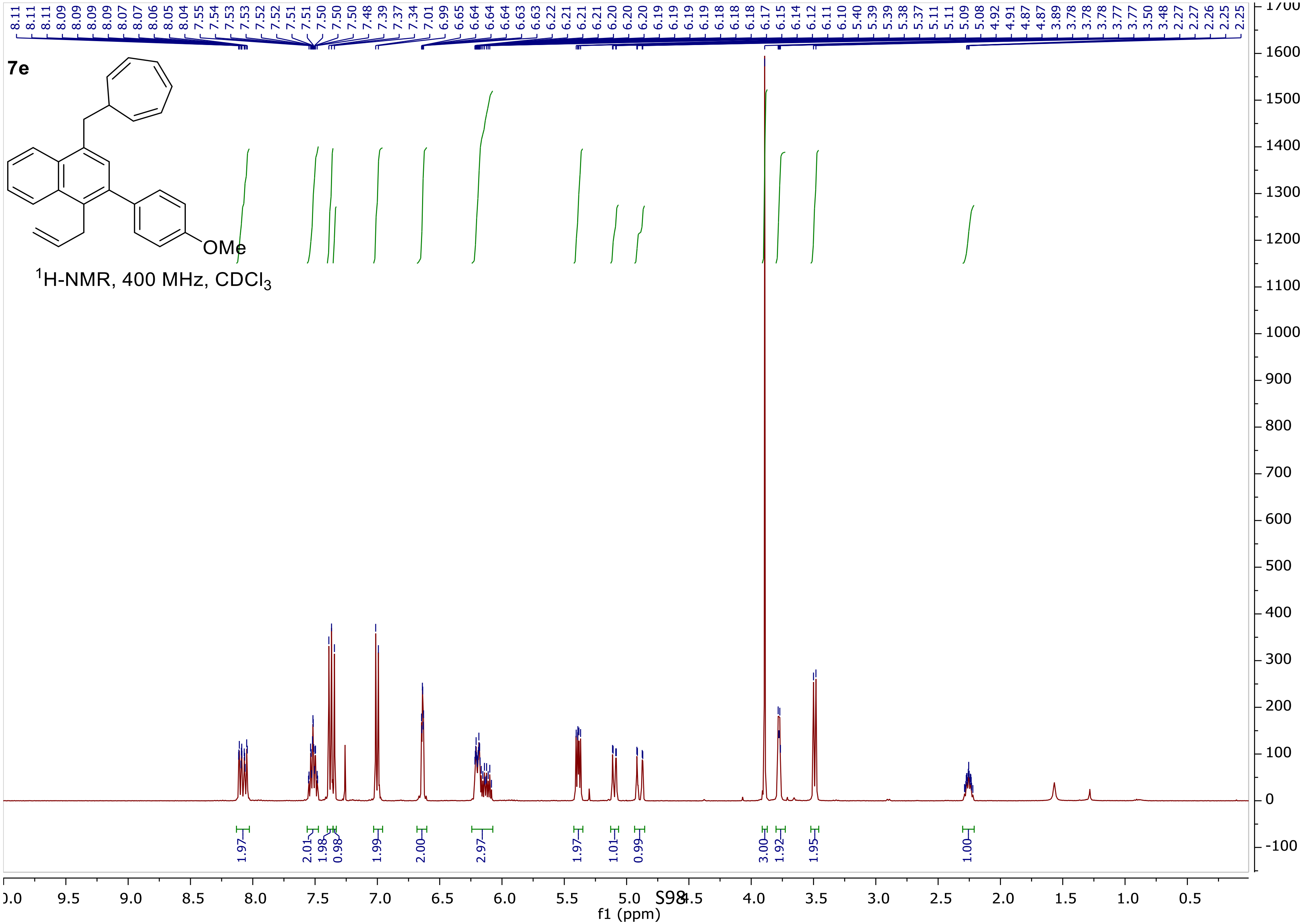

7e
OMe
1H-NMR, 400 MHz, CDCl3
f1 (ppm)

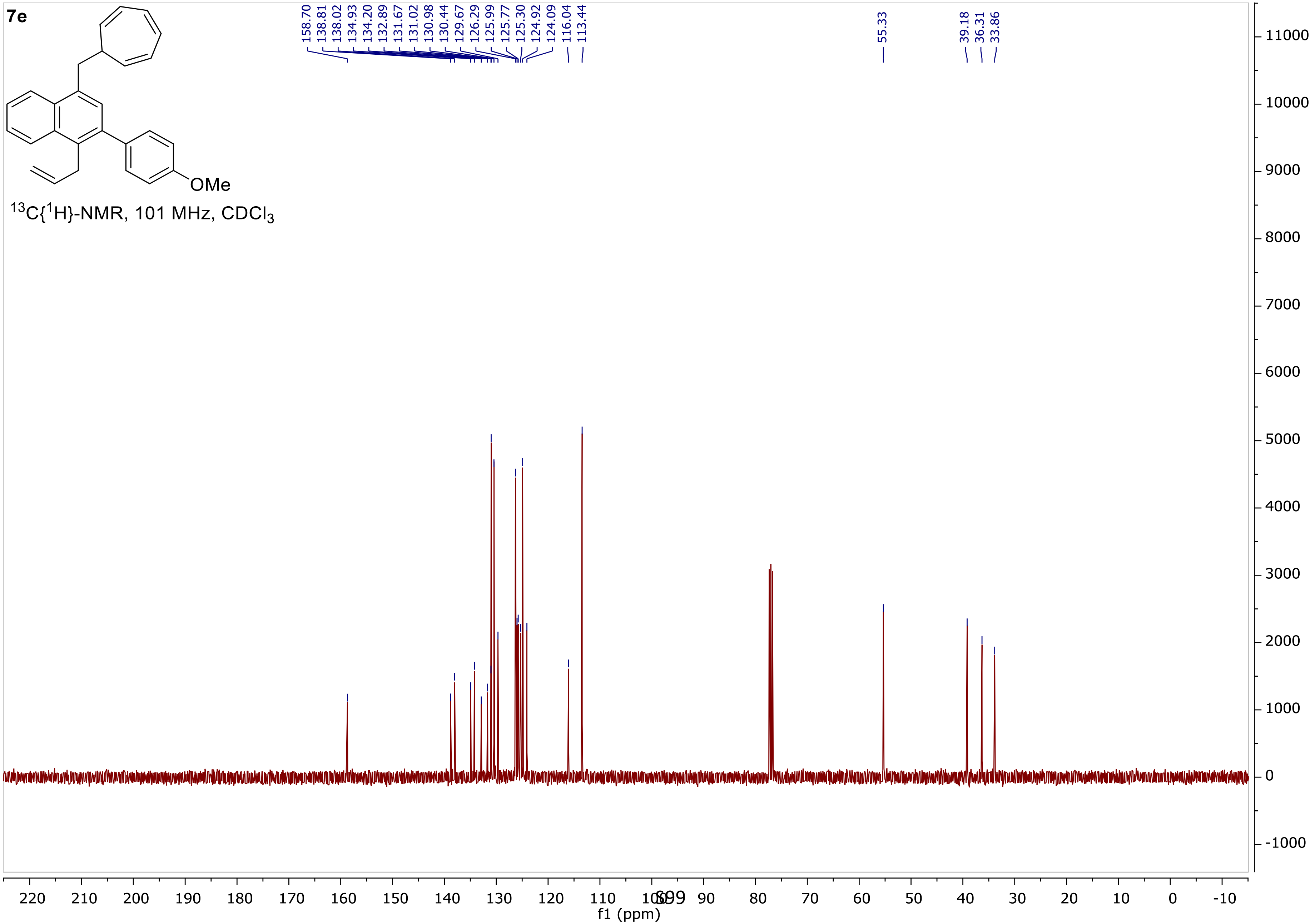

7e
OMe
$^{13}C\{^{1}H\}$-NMR, 101 MHz, $CDCl_3$
158.70
138.81
138.02
134.93
134.20
132.89
131.67
131.02
130.98
130.44
129.67
126.29
125.99
125.77
125.30
124.92
124.09
116.04
113.44
55.33
39.18
36.31
33.86
f1 (ppm)

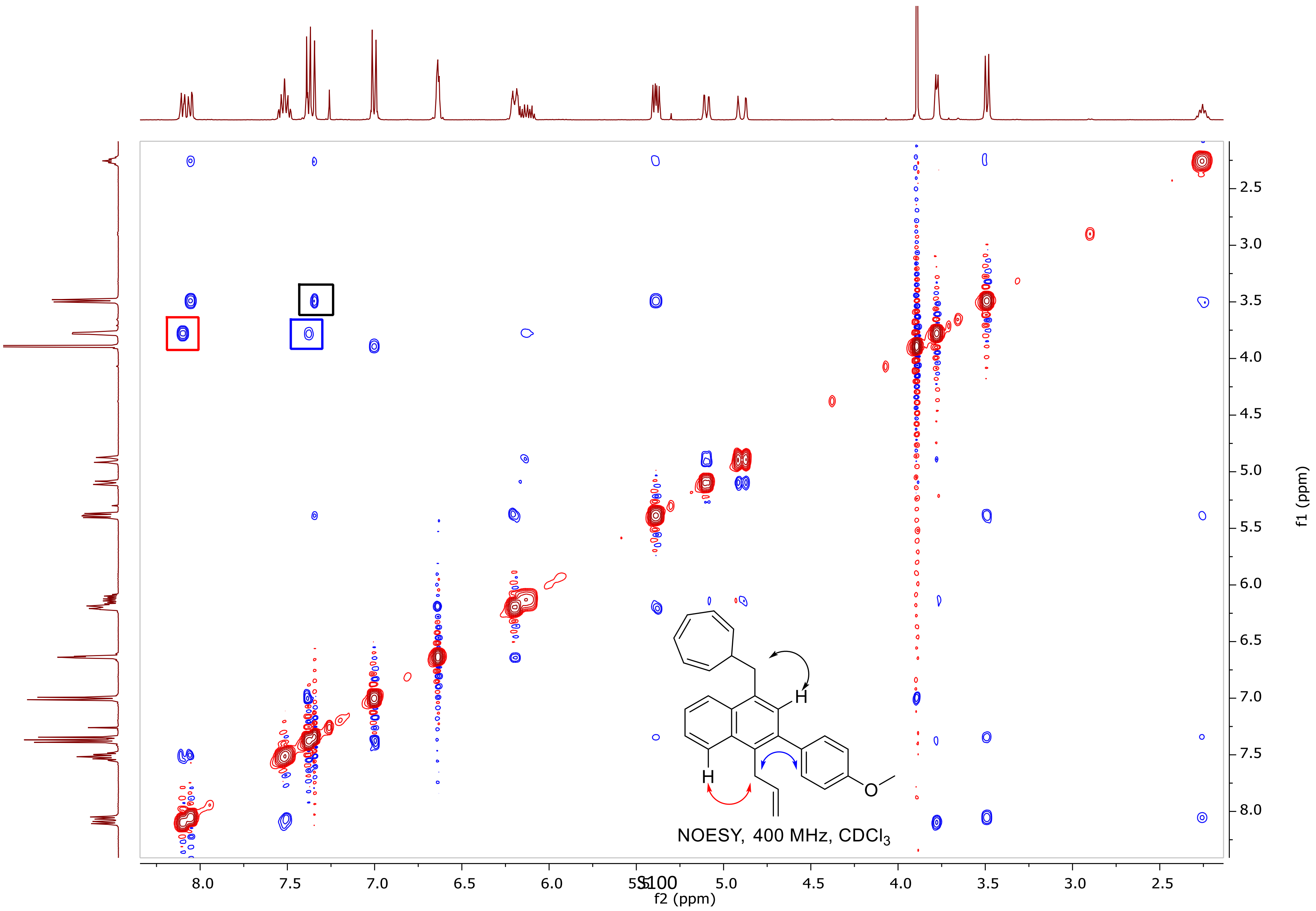

NOESY, 400 MHz, $CDCl_3$
f2 (ppm)
f1 (ppm)
H
H
O

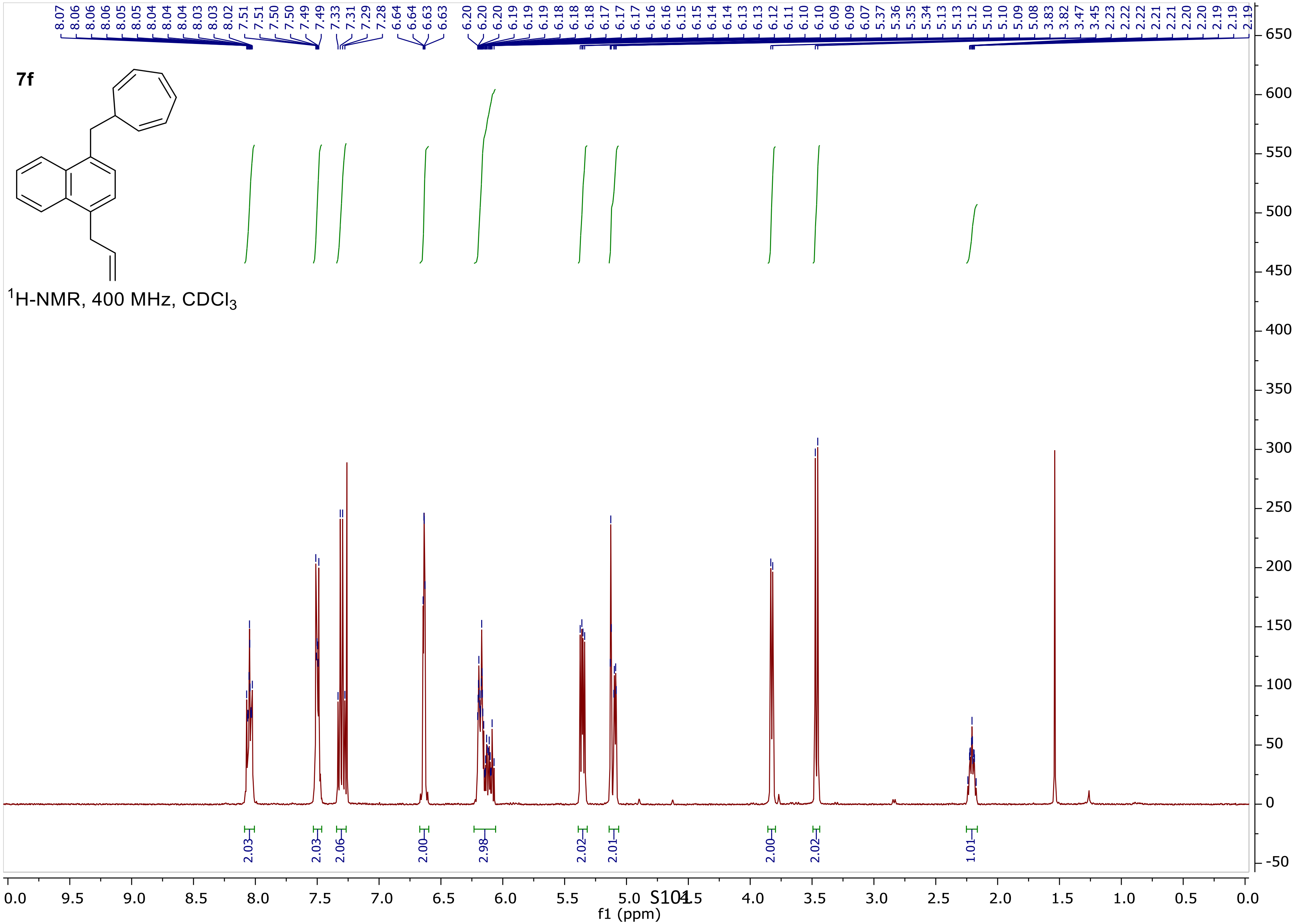
7f
¹H-NMR, 400 MHz, CDCl₃
8.07 8.06 8.06 8.06 8.05 8.04 8.04 8.04 8.03 8.02 7.51 7.51 7.50 7.50 7.49 7.49 7.33 7.31 7.29 7.28 6.64 6.64 6.63 6.63 6.20 6.20 6.20 6.19 6.19 6.19 6.18 6.18 6.17 6.17 6.17 6.16 6.15 6.15 6.14 6.13 6.13 6.12 6.11 6.10 6.10 6.09 6.09 6.07 5.37 5.36 5.35 5.34 5.13 5.13 5.12 5.10 5.10 5.09 5.08 3.83 3.82 3.47 3.45 2.23 2.22 2.22 2.21 2.21 2.20 2.20 2.19 2.19 2.19
2.03 2.03 2.06 2.00 2.98 2.02 2.01 2.00 2.02 1.01
f1 (ppm)

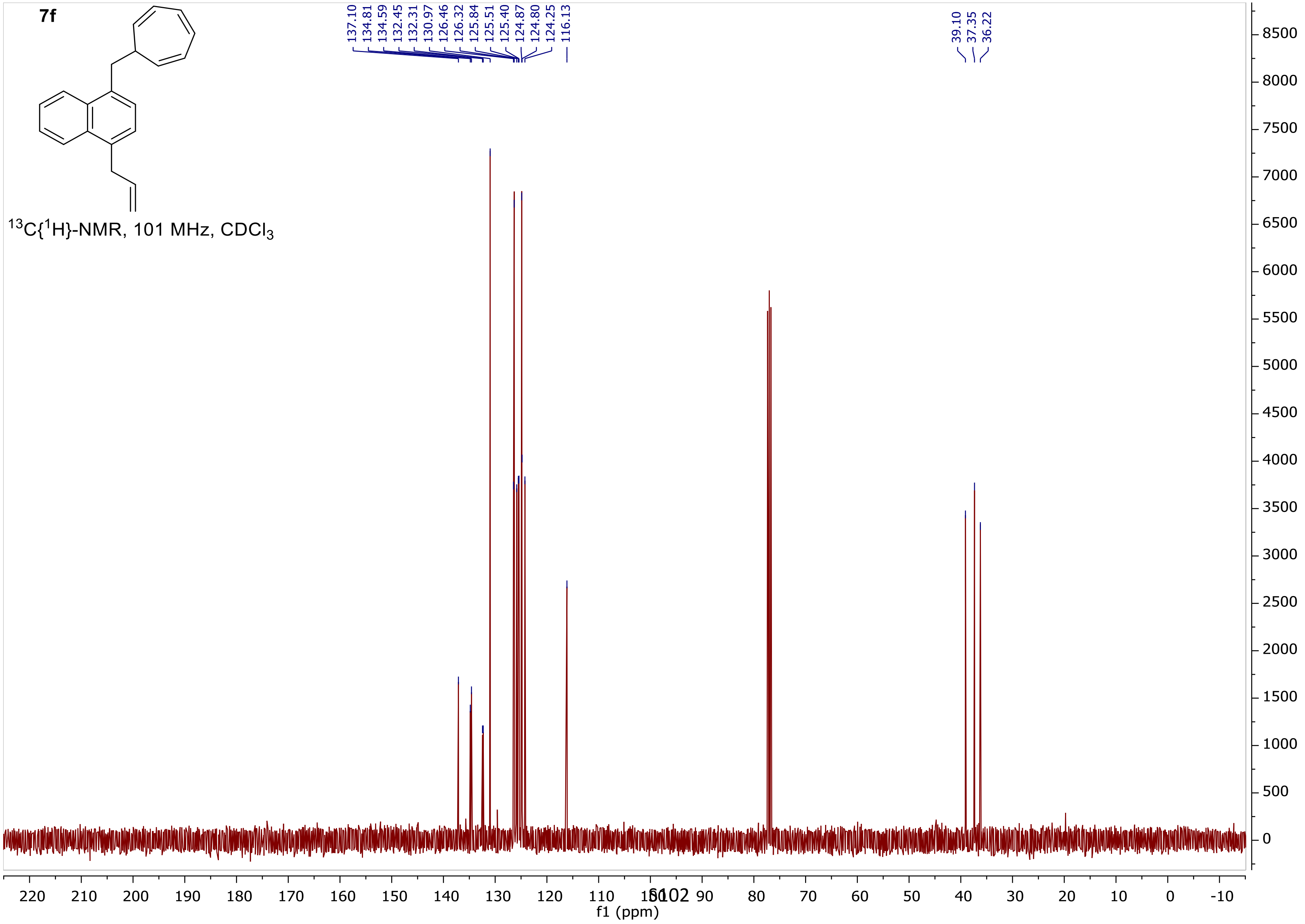
7f
$^{13}C\{^1H\}$-NMR, 101 MHz, $CDCl_3$
137.10
134.81
134.59
132.45
132.31
130.97
126.46
126.32
125.84
125.51
125.40
124.87
124.80
124.25
116.13
39.10
37.35
36.22
f1 (ppm)

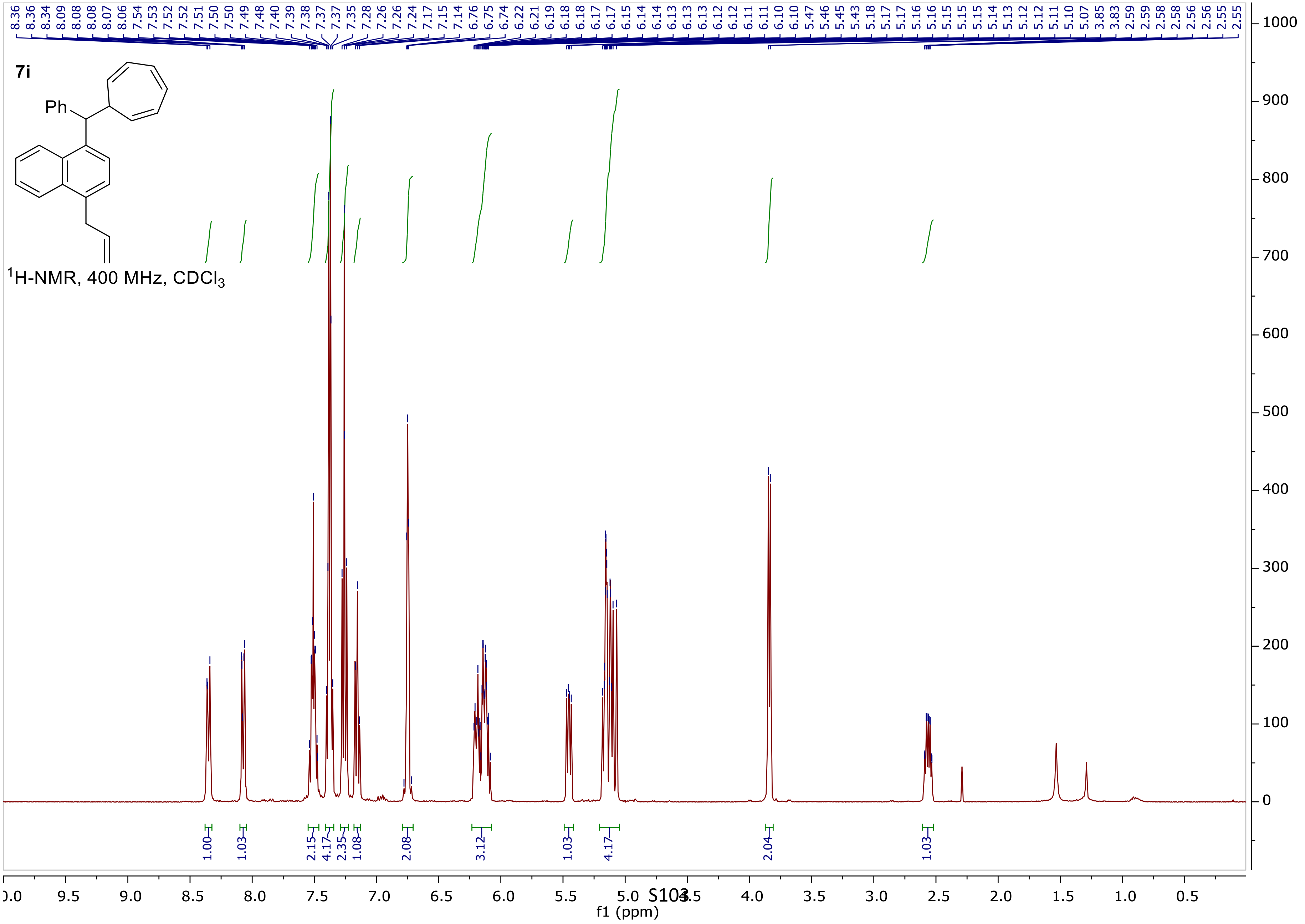
7i
Ph
1H-NMR, 400 MHz, CDCl3
8.36 8.36 8.34 8.09 8.08 8.08 8.07 8.06 7.54 7.53 7.52 7.52 7.51 7.50 7.50 7.49 7.48 7.40 7.39 7.38 7.37 7.37 7.35 7.28 7.26 7.26 7.24 7.17 7.15 7.14 6.76 6.75 6.74 6.22 6.21 6.19 6.18 6.18 6.17 6.17 6.15 6.14 6.14 6.13 6.13 6.13 6.12 6.12 6.11 6.11 6.10 6.10 5.47 5.46 5.45 5.43 5.18 5.17 5.17 5.16 5.16 5.15 5.15 5.15 5.14 5.13 5.12 5.11 5.10 5.07 3.85 3.83 2.59 2.59 2.58 2.58 2.56 2.55 2.55
1.00 1.03 2.15 4.17 2.35 1.08 2.08 3.12 1.03 4.17 2.04 1.03
f1 (ppm)

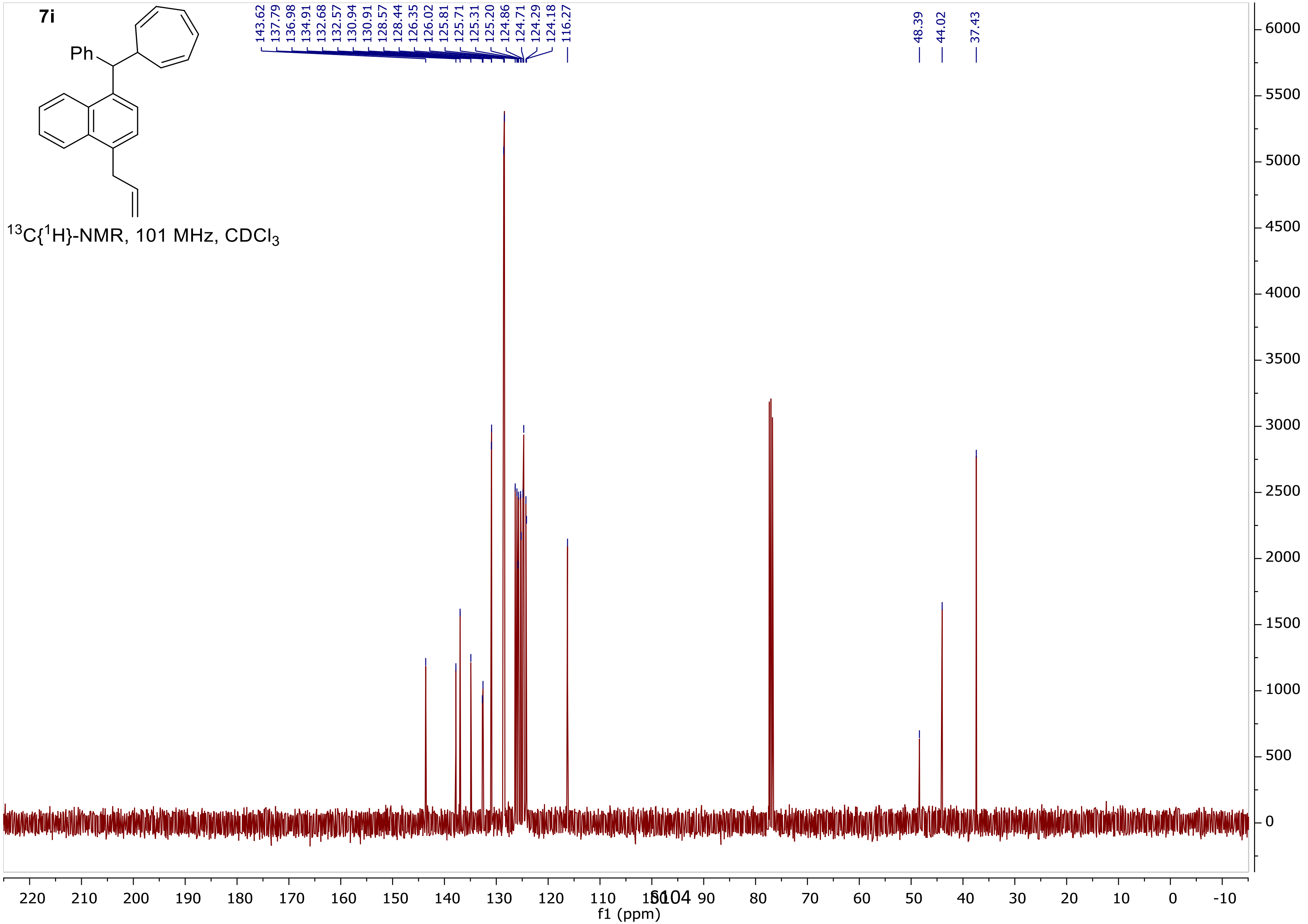

7i
Ph
$^{13}C\{^{1}H\}$-NMR, 101 MHz, $CDCl_3$
143.62
137.79
136.98
134.91
132.68
132.57
130.94
130.91
128.57
128.44
126.35
126.02
125.81
125.71
125.31
125.20
124.86
124.71
124.29
124.18
116.27
48.39
44.02
37.43
f1 (ppm)

7j

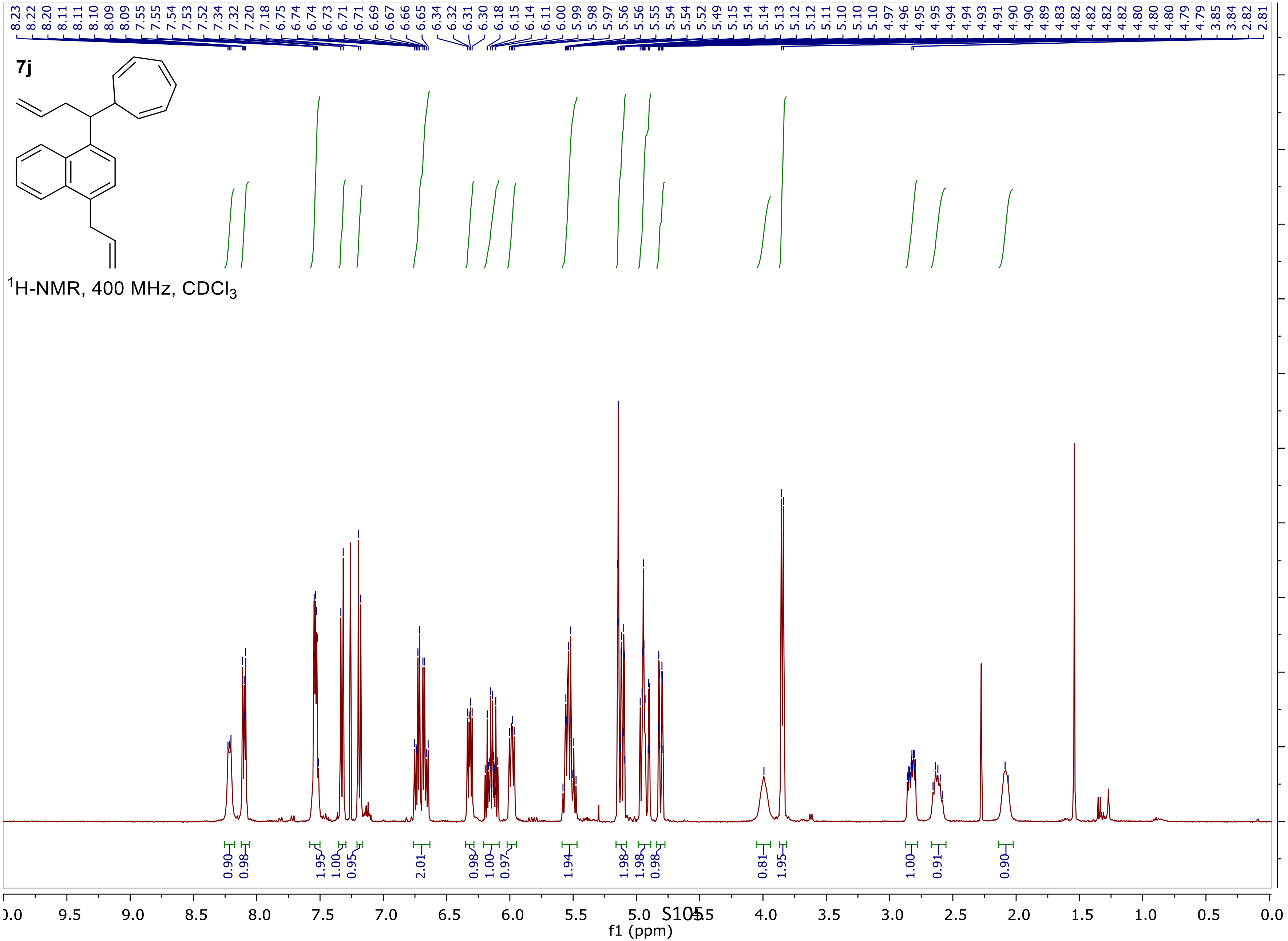

^{1}H-NMR, 400 MHz, $CDCl_3$

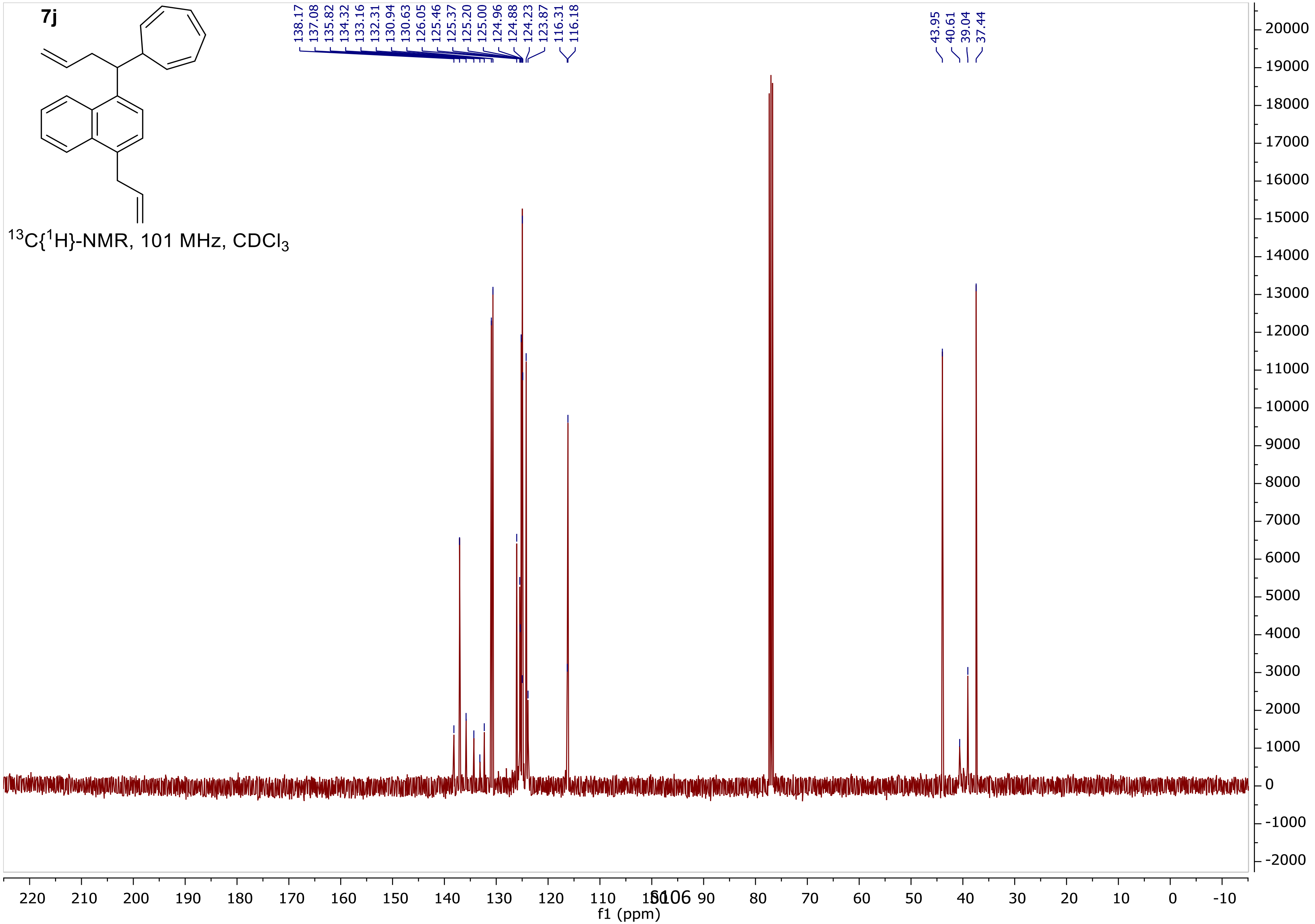
7j
13C{1H}-NMR, 101 MHz, CDCl3
138.17
137.08
135.82
134.32
133.16
132.31
130.94
130.63
126.05
125.46
125.37
125.20
125.00
124.96
124.88
124.23
123.87
116.31
116.18
43.95
40.61
39.04
37.44
f1 (ppm)

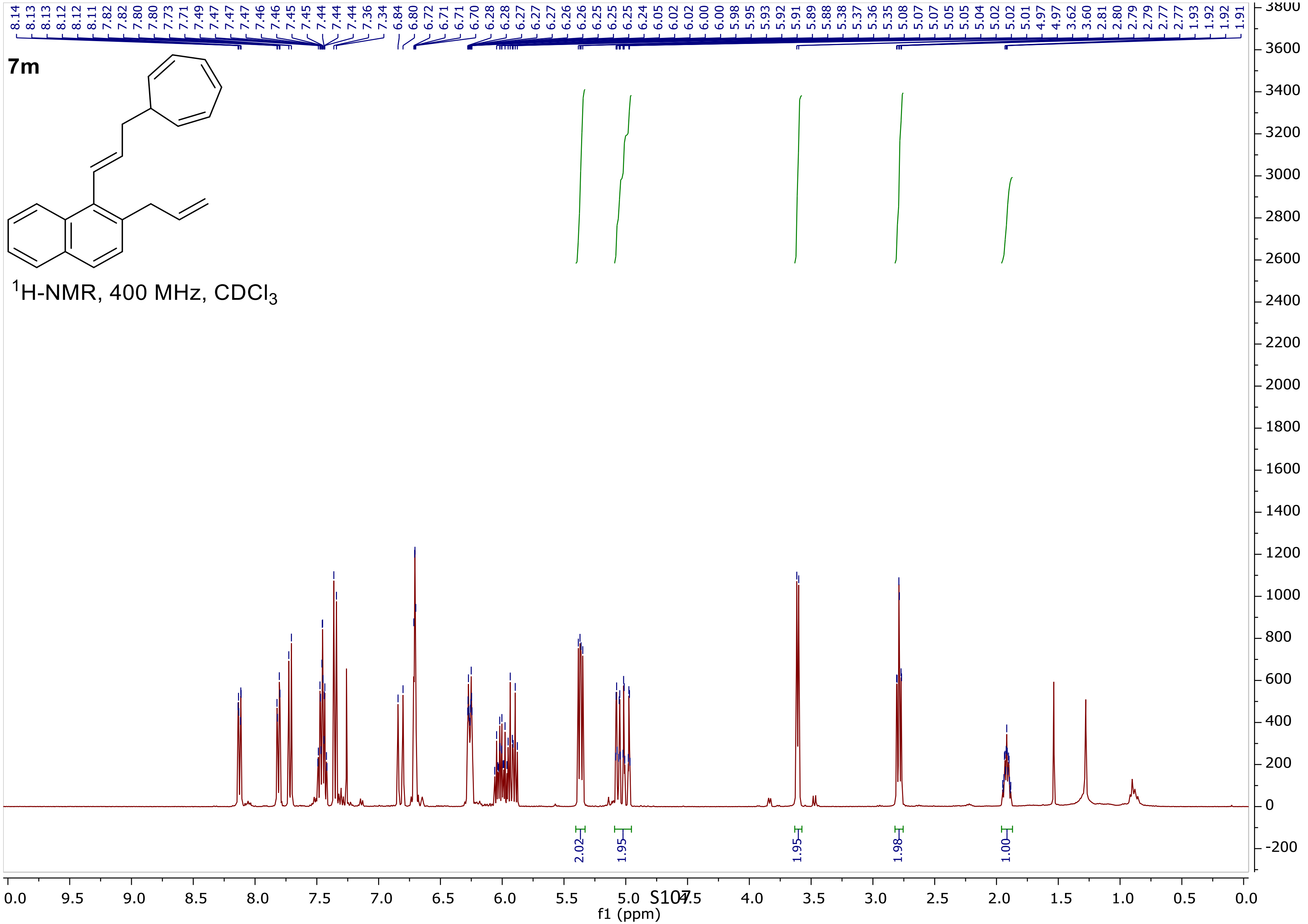
7m
1H-NMR, 400 MHz, CDCl3
8.14 8.13 8.13 8.12 8.12 8.11 7.82 7.82 7.80 7.80 7.73 7.71 7.49 7.47 7.47 7.47 7.46 7.45 7.45 7.44 7.44 7.44 7.36 7.34 6.84 6.80 6.72 6.71 6.71 6.70 6.28 6.28 6.27 6.27 6.26 6.26 6.25 6.25 6.24 6.05 6.02 6.02 6.00 6.00 5.98 5.95 5.93 5.92 5.91 5.89 5.88 5.38 5.37 5.36 5.35 5.08 5.07 5.07 5.05 5.05 5.04 5.02 5.02 5.01 4.97 3.62 3.60 2.81 2.80 2.79 2.79 2.77 2.77 1.93 1.92 1.92 1.91
2.02
1.95
1.95
1.98
1.00
f1 (ppm)

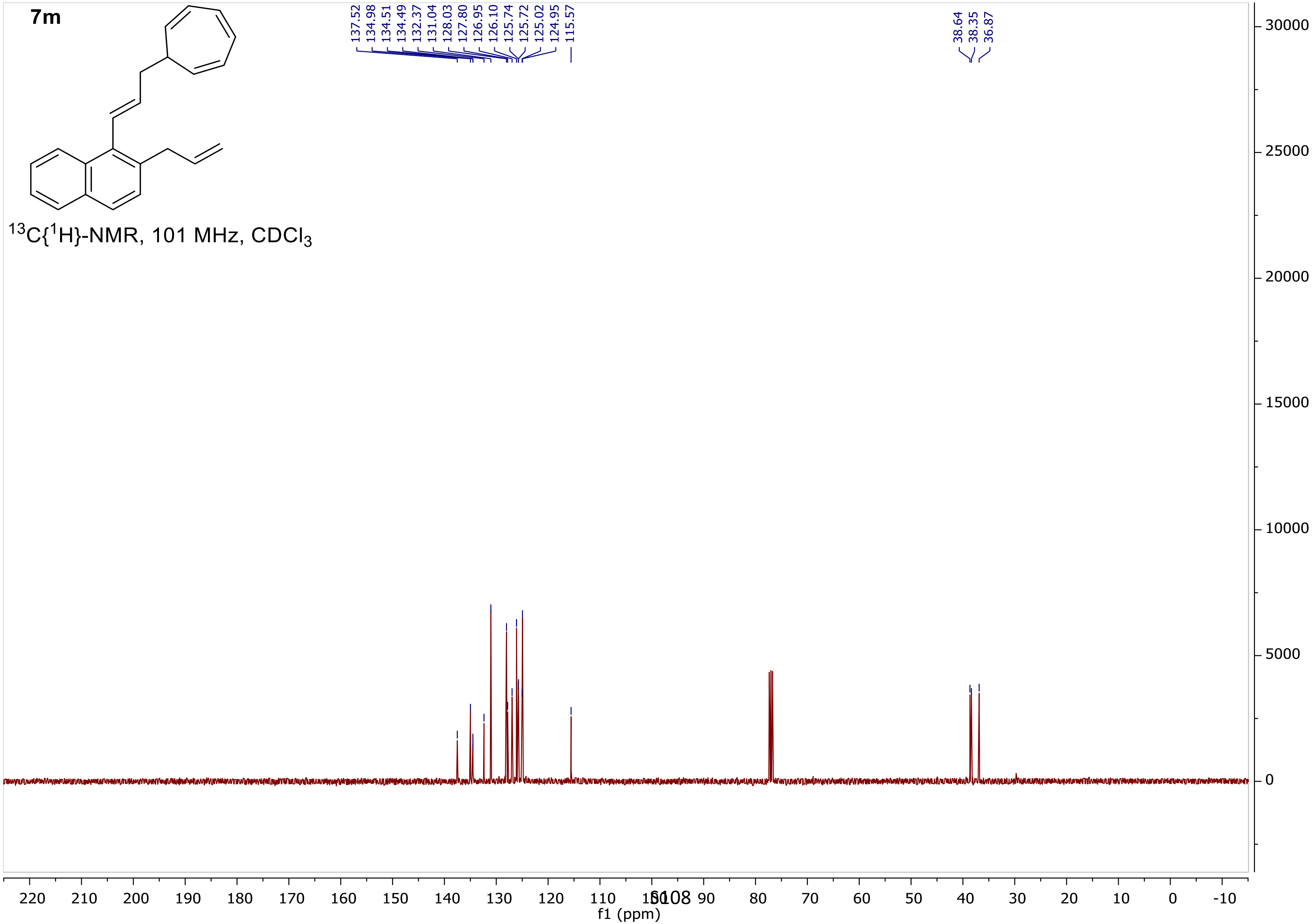
7m
$^{13}C\{^1H\}$-NMR, 101 MHz, $CDCl_3$
137.52
134.98
134.51
134.49
132.37
131.04
128.03
127.80
126.95
126.10
125.74
125.72
125.02
124.95
115.57
38.64
38.35
36.87
30000
25000
20000
15000
10000
5000
0
220 210 200 190 180 170 160 150 140 130 120 110 100 90 80 70 60 50 40 30 20 10 0 -10
f1 (ppm)

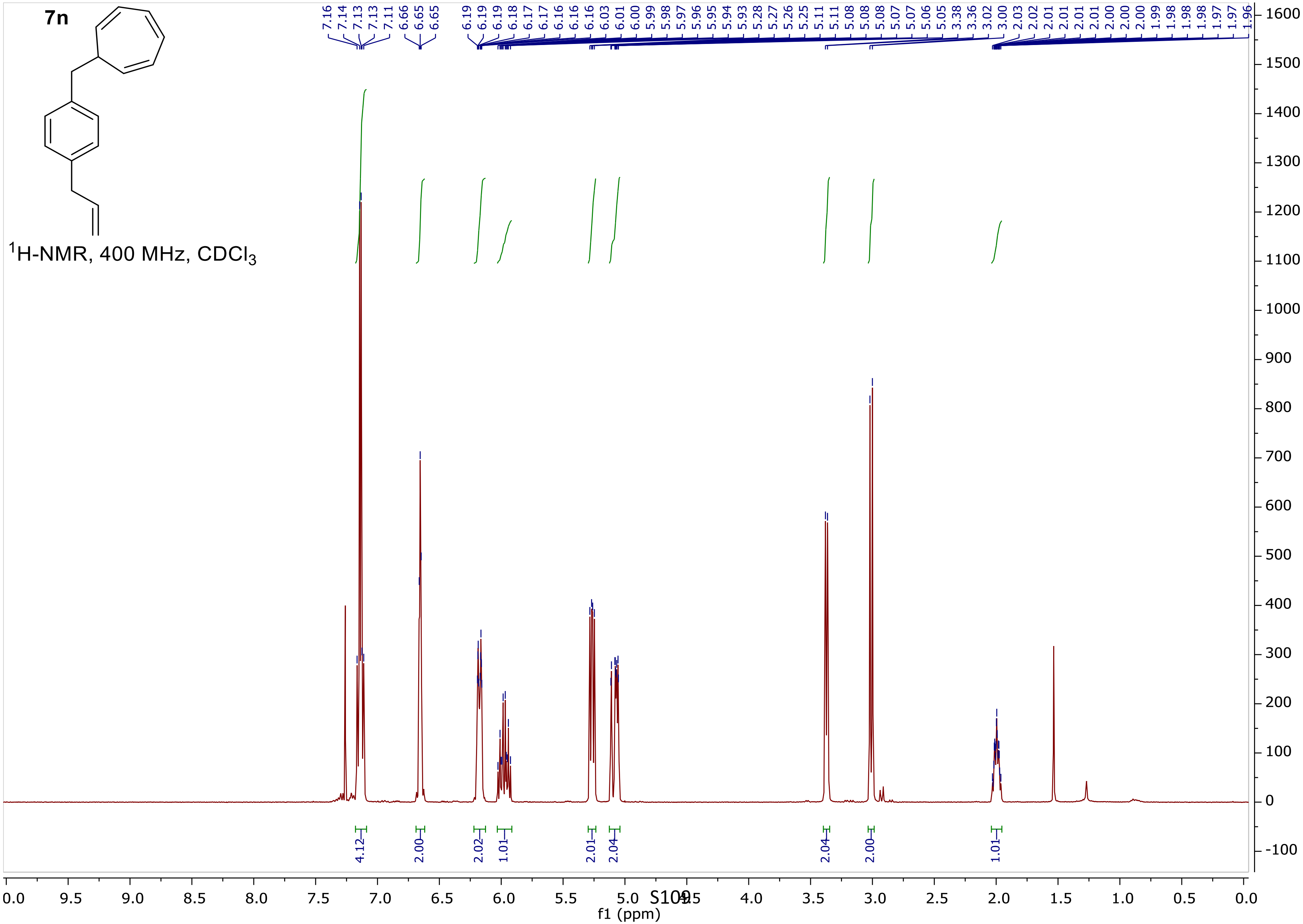
7n
^{1}H-NMR, 400 MHz, CDCl$_3$
f1 (ppm)

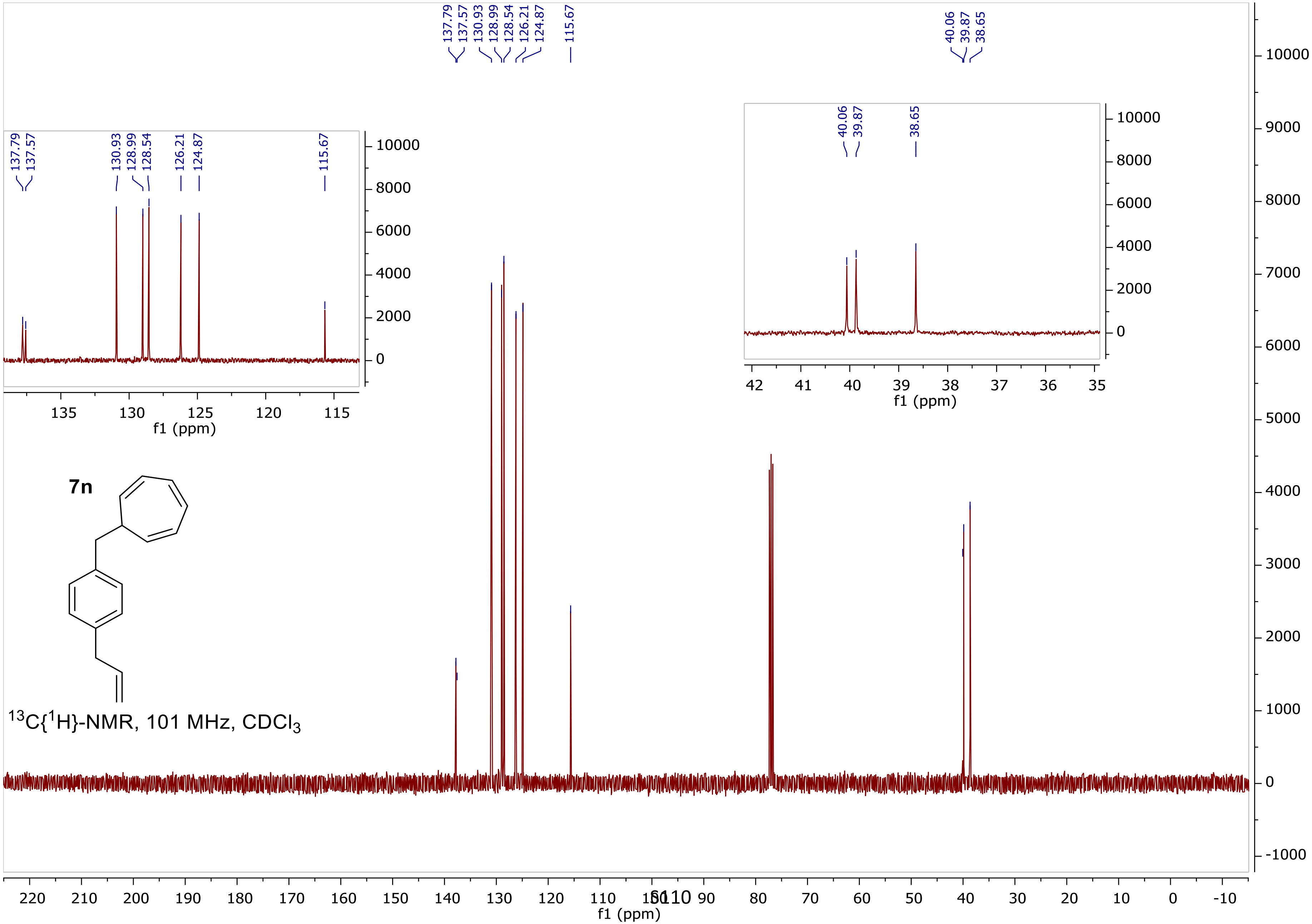
137.79
137.57
130.93
128.99
128.54
126.21
124.87
115.67
40.06
39.87
38.65
f1 (ppm)
7n
$^{13}C\{^{1}H\}$-NMR, 101 MHz, $CDCl_3$

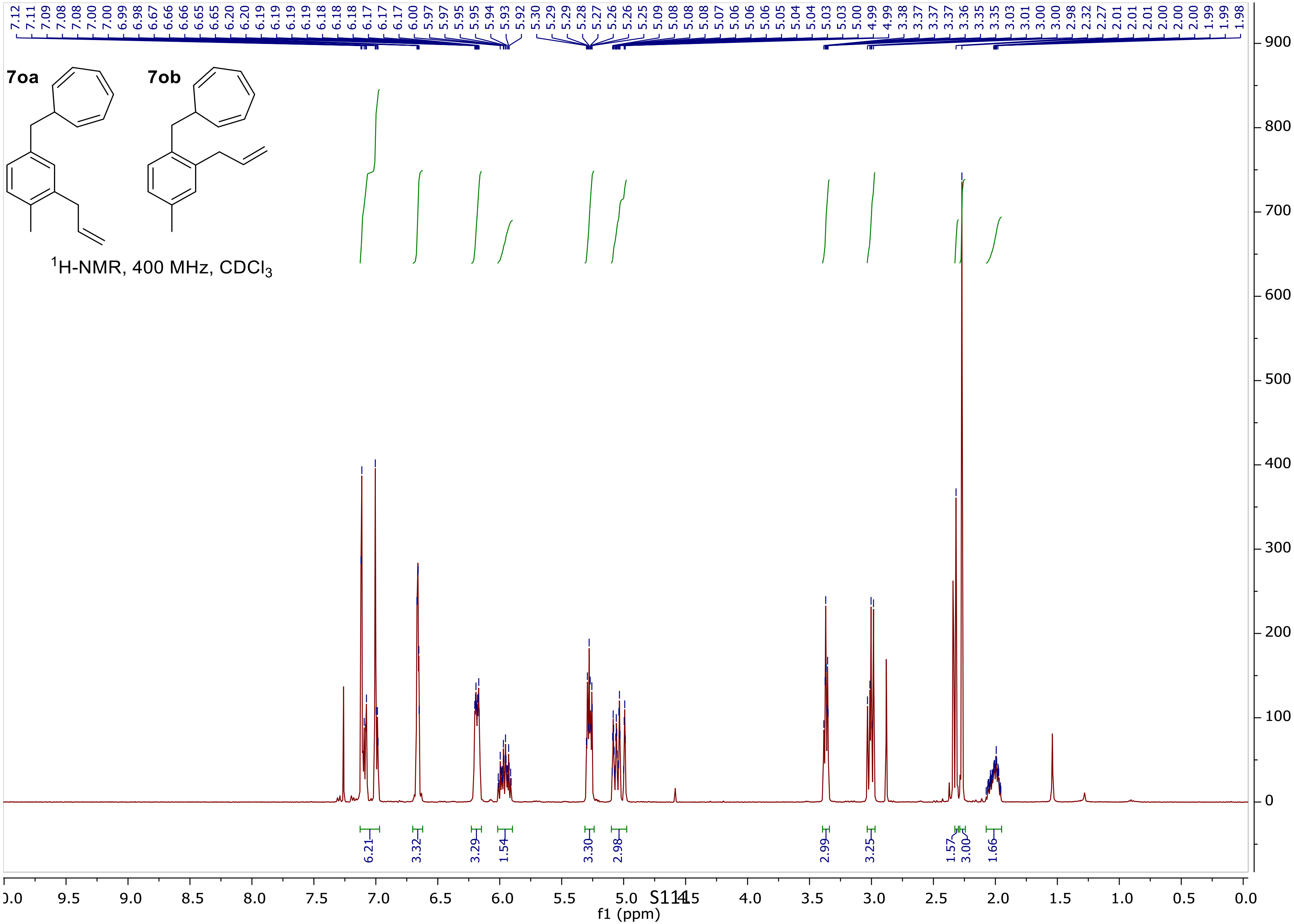
7oa
7ob
^{1}H-NMR, 400 MHz, $CDCl_3$
f1 (ppm)

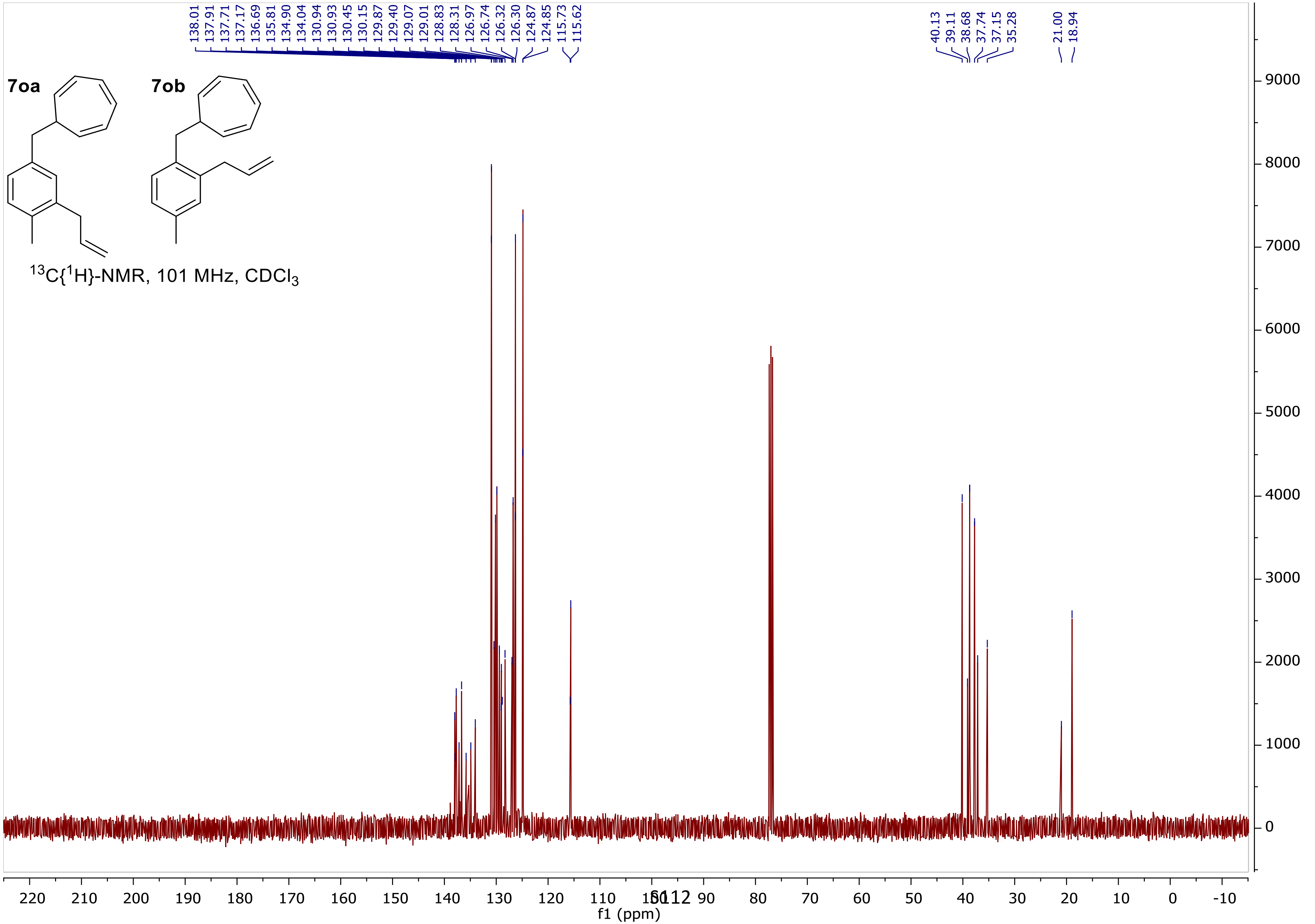

138.01
137.91
137.71
137.17
136.69
135.81
134.90
134.04
130.94
130.93
130.45
130.15
129.87
129.40
129.07
129.01
128.83
128.31
126.97
126.74
126.32
126.30
124.87
124.85
115.73
115.62
40.13
39.11
38.68
37.74
37.15
35.28
21.00
18.94
7oa
7ob
$^{13}C\{^1H\}$-NMR, 101 MHz, $CDCl_3$
f1 (ppm)

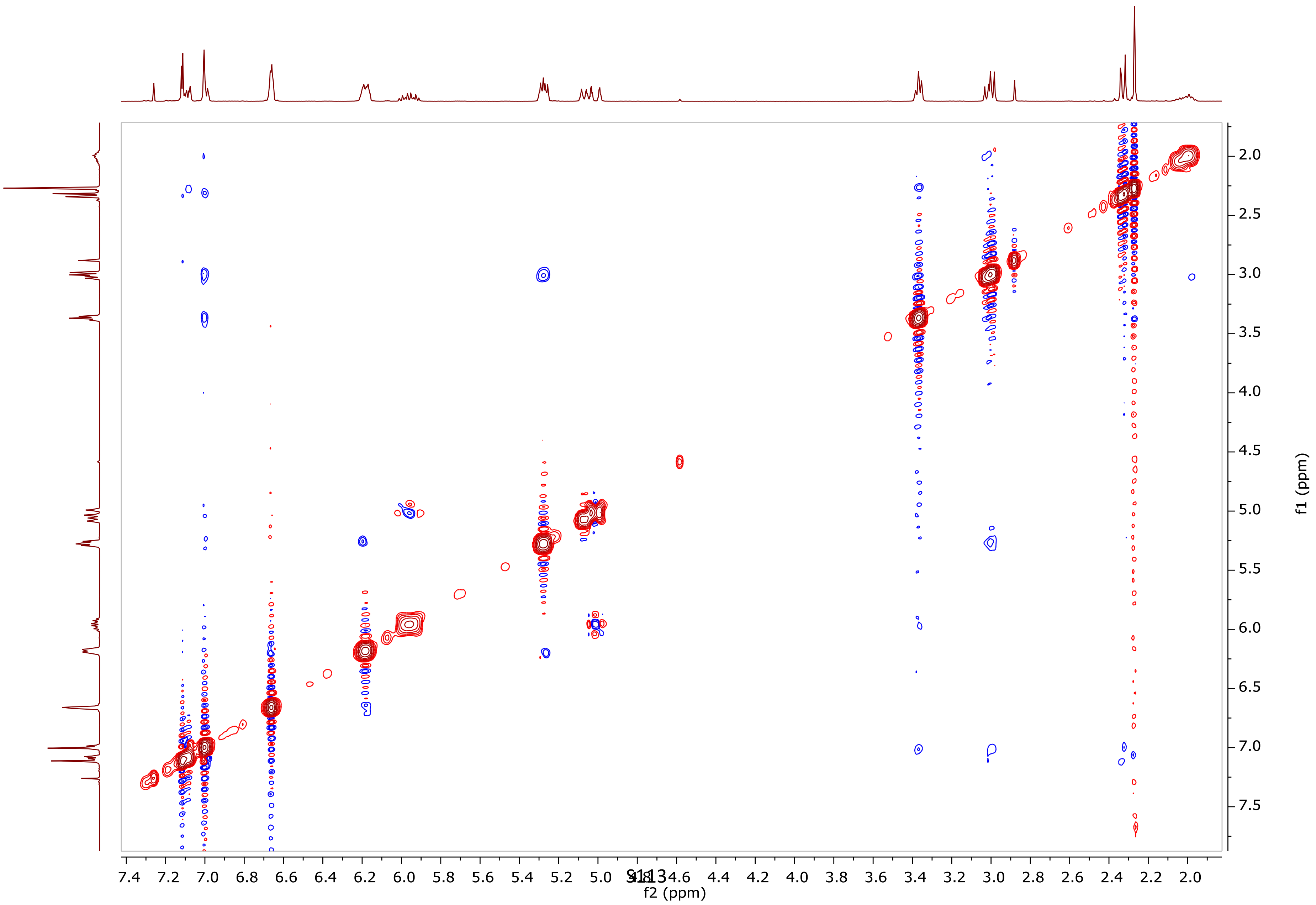
7.4 7.2 7.0 6.8 6.6 6.4 6.2 6.0 5.8 5.6 5.4 5.2 5.0 4.6 4.4 4.2 4.0 3.8 3.6 3.4 3.2 3.0 2.8 2.6 2.4 2.2 2.0
f2 (ppm)
2.0 2.5 3.0 3.5 4.0 4.5 5.0 5.5 6.0 6.5 7.0 7.5
f1 (ppm)

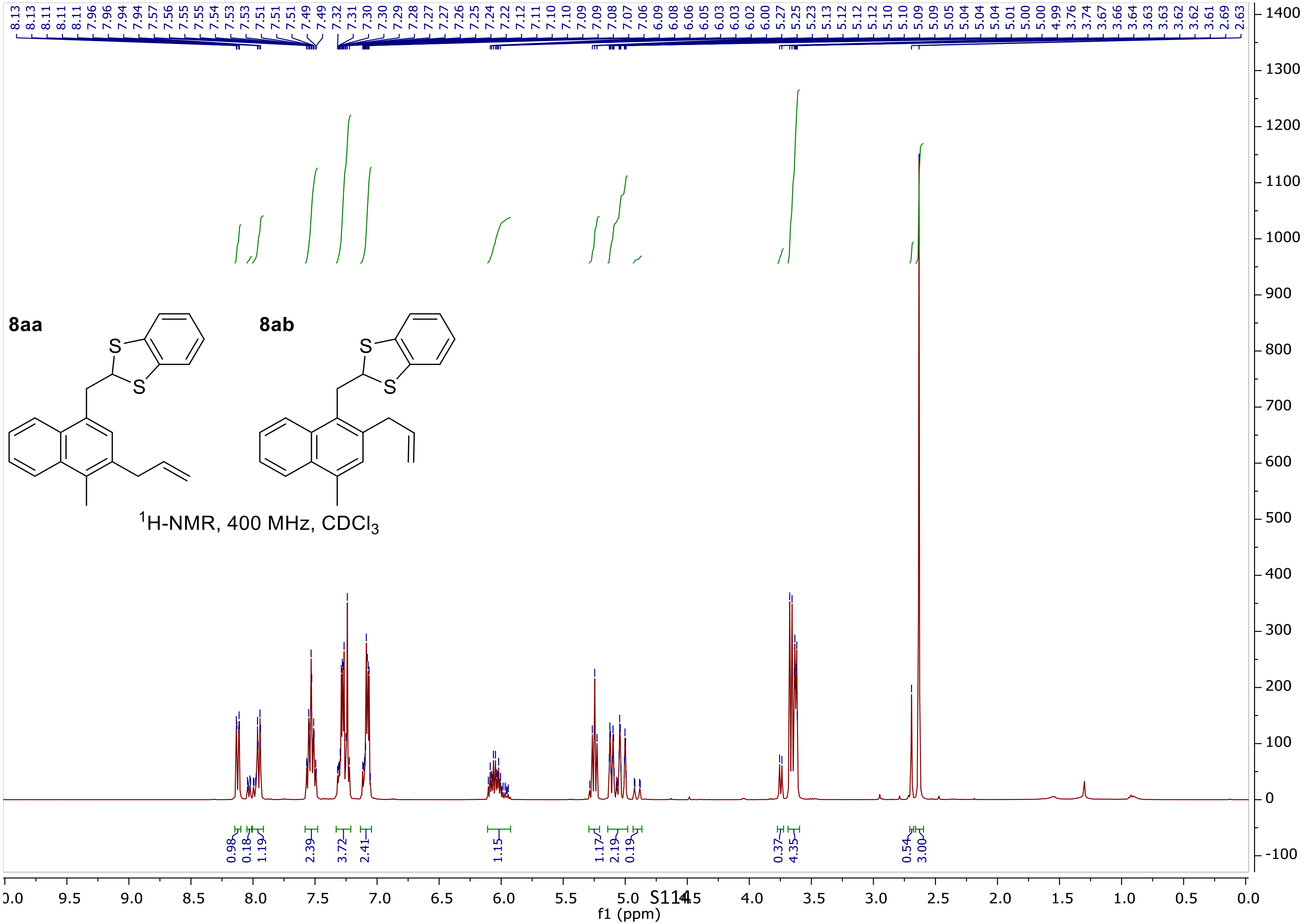
8aa
8ab
S
S
^{1}H-NMR, 400 MHz, CDCl$_3$
f1 (ppm)

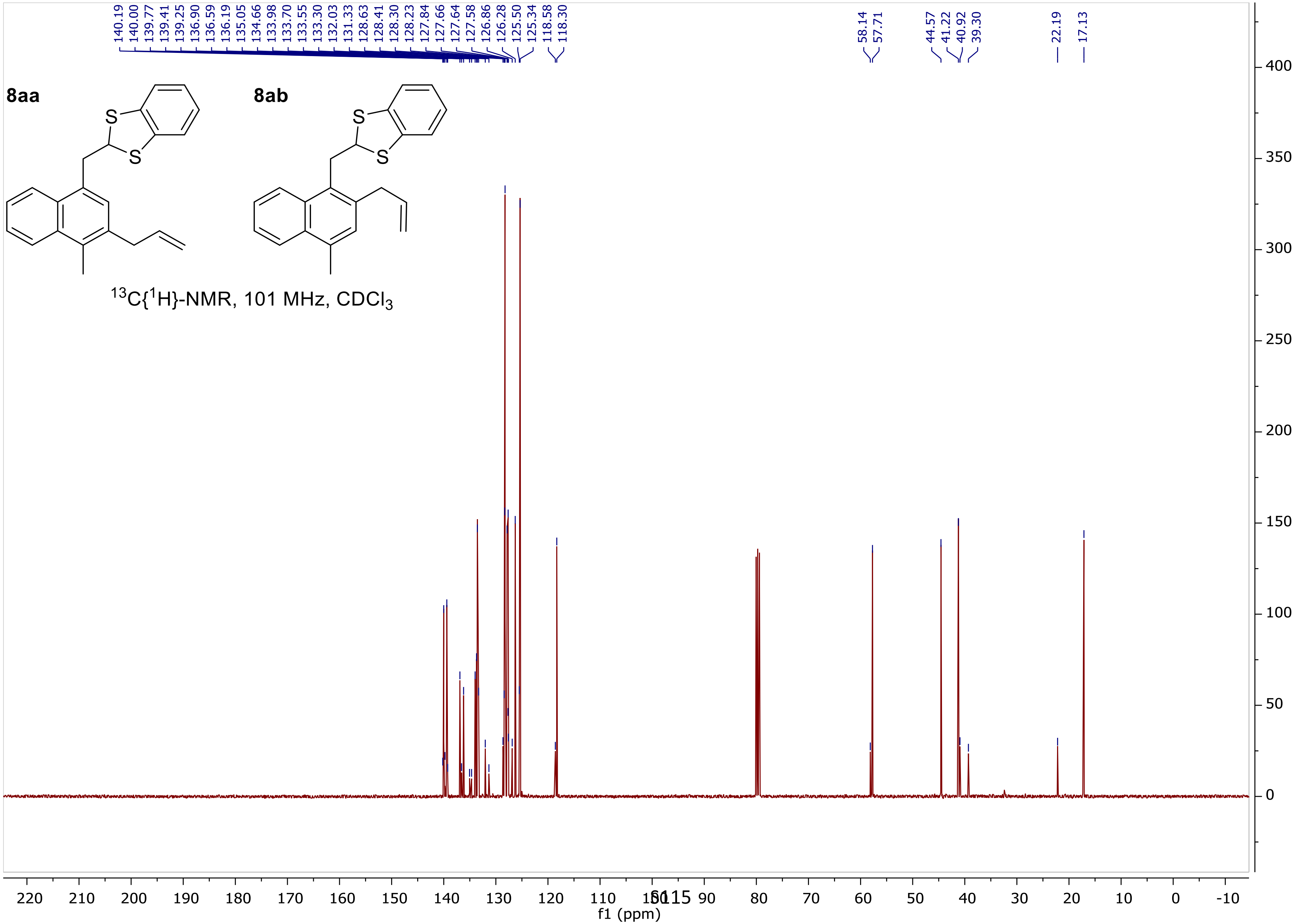
140.19
140.00
139.77
139.41
139.25
136.90
136.59
136.19
135.05
134.66
133.98
133.70
133.55
133.30
132.03
131.33
128.63
128.41
128.30
128.23
127.84
127.66
127.64
127.58
126.86
126.28
125.50
125.34
118.58
118.30
58.14
57.71
44.57
41.22
40.92
39.30
22.19
17.13
8aa
8ab
S
S
$^{13}C\{^1H\}$-NMR, 101 MHz, $CDCl_3$
f1 (ppm)

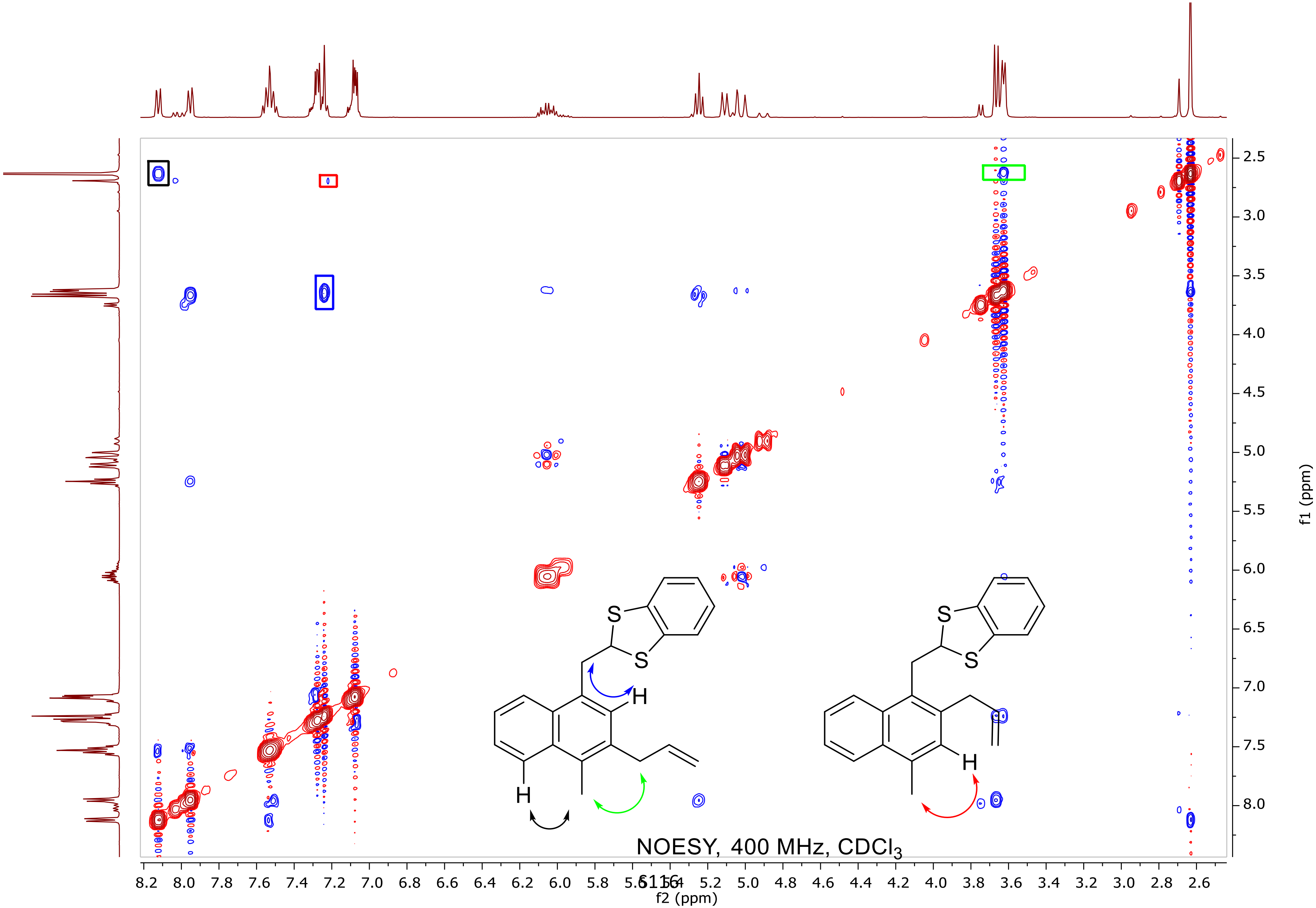
NOESY, 400 MHz, $CDCl_3$
f1 (ppm)
f2 (ppm)
S
S
H
H
S
S
H
8.2 8.0 7.8 7.6 7.4 7.2 7.0 6.8 6.6 6.4 6.2 6.0 5.8 5.6 5.4 5.2 5.0 4.8 4.6 4.4 4.2 4.0 3.8 3.6 3.4 3.2 3.0 2.8 2.6
2.5 3.0 3.5 4.0 4.5 5.0 5.5 6.0 6.5 7.0 7.5 8.0

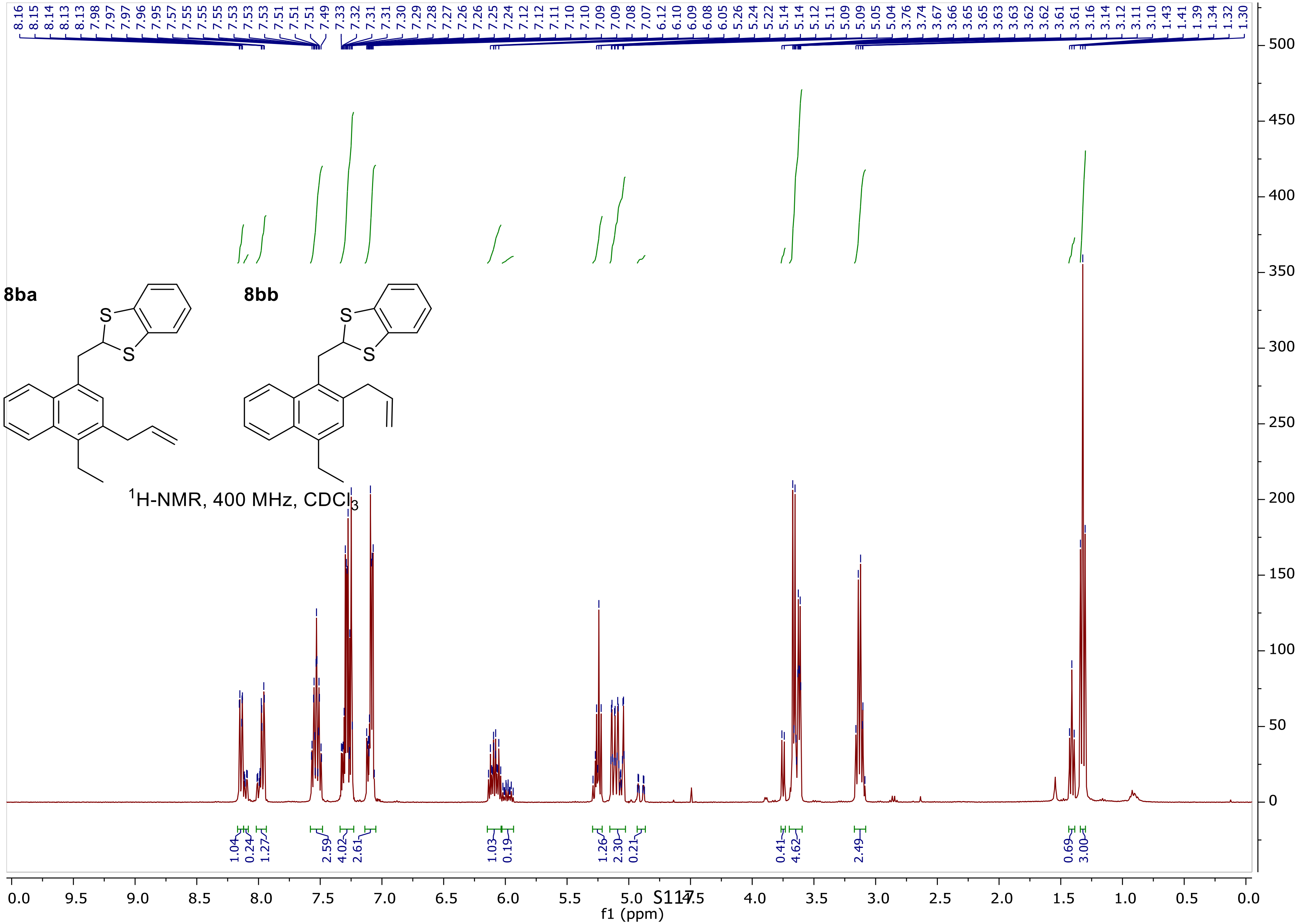
8ba
8bb
S
S
1H-NMR, 400 MHz, CDCl3
f1 (ppm)

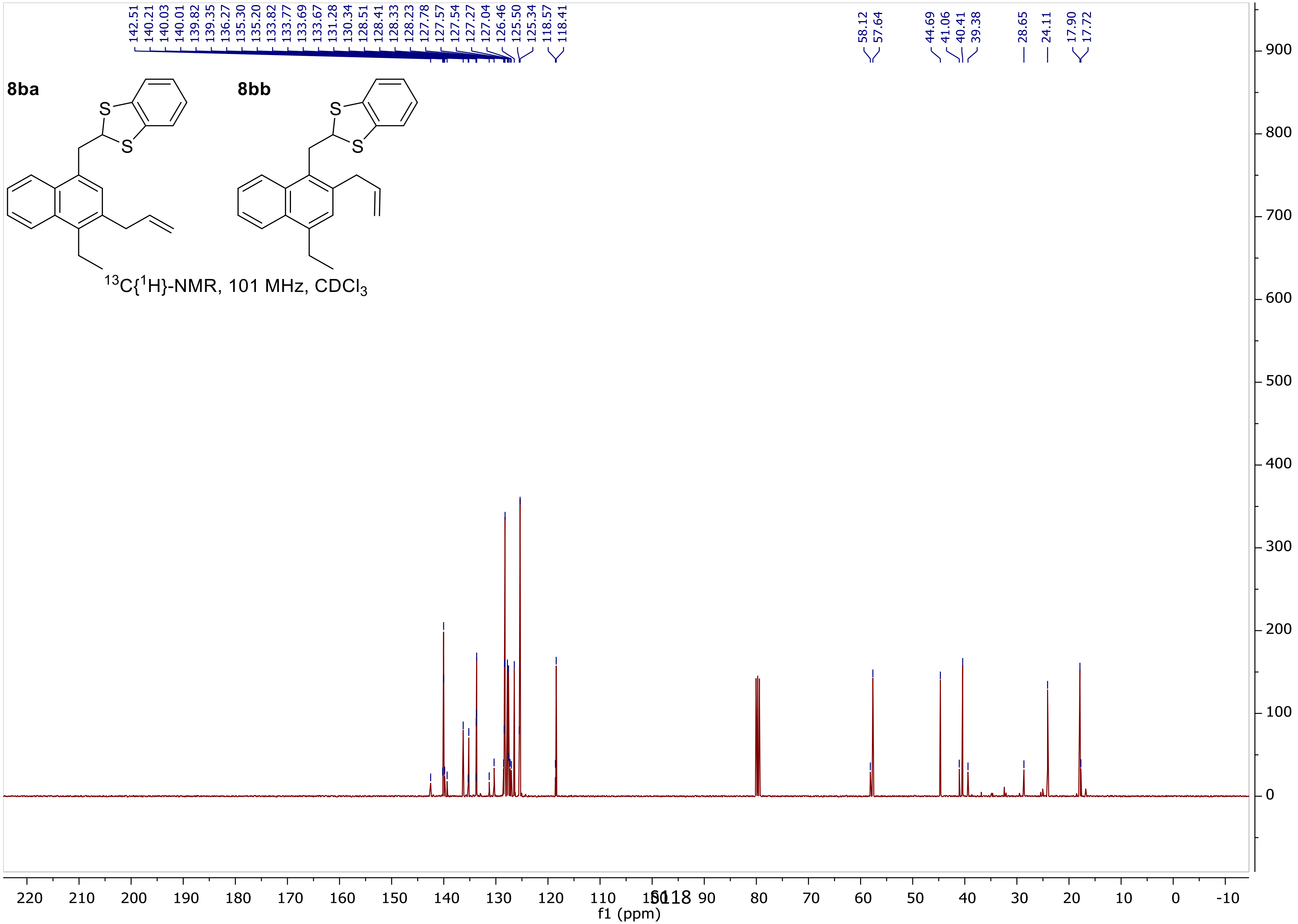

142.51
140.21
140.03
140.01
139.82
139.35
136.27
135.30
135.20
133.82
133.77
133.69
133.67
131.28
130.34
128.51
128.41
128.33
128.23
127.78
127.57
127.54
127.27
127.04
126.46
125.50
125.34
118.57
118.41
58.12
57.64
44.69
41.06
40.41
39.38
28.65
24.11
17.90
17.72
8ba
8bb
S
S
13C{1H}-NMR, 101 MHz, CDCl3
f1 (ppm)

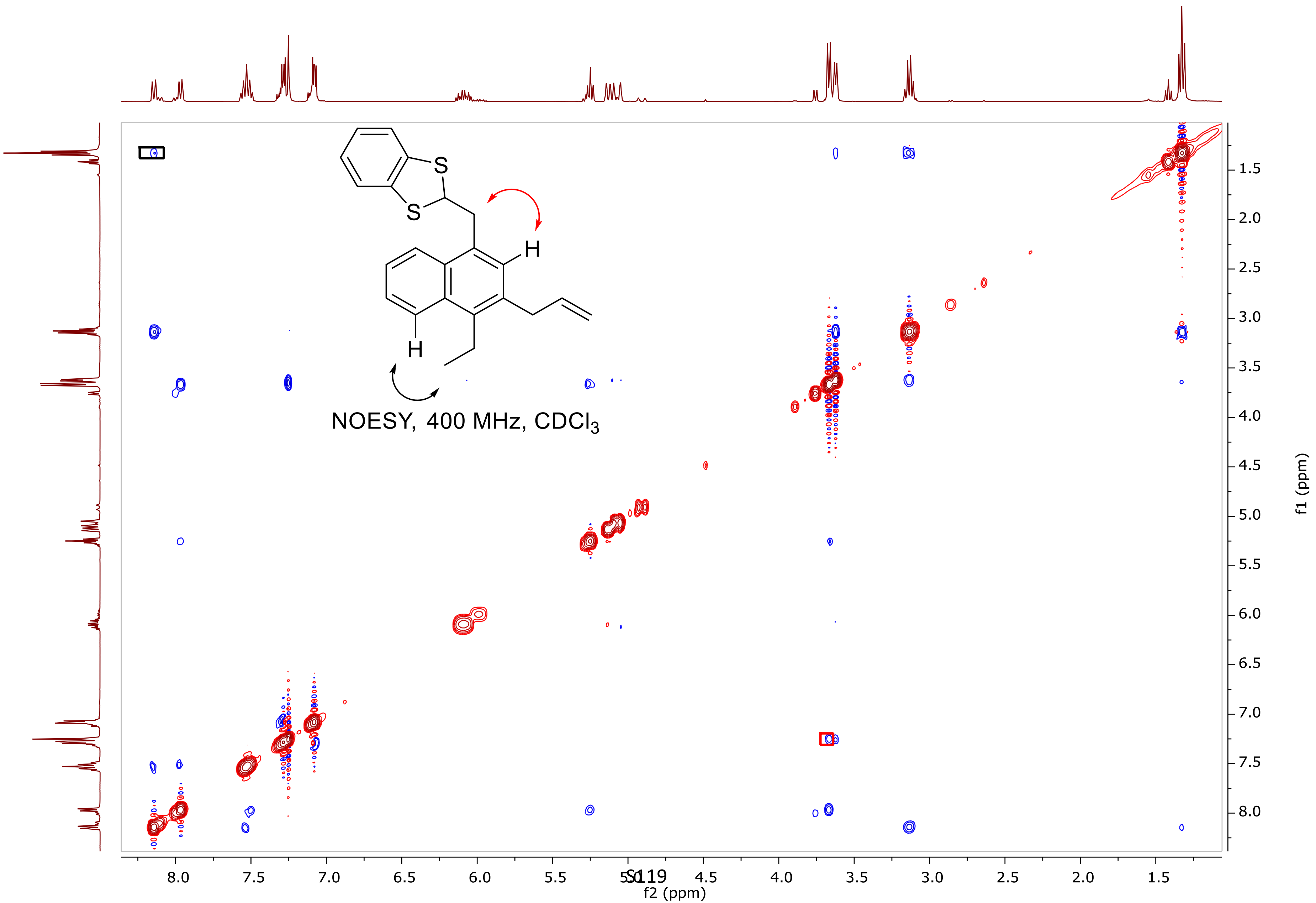

S
S
H
H
NOESY, 400 MHz, CDCl3
f1 (ppm)
f2 (ppm)

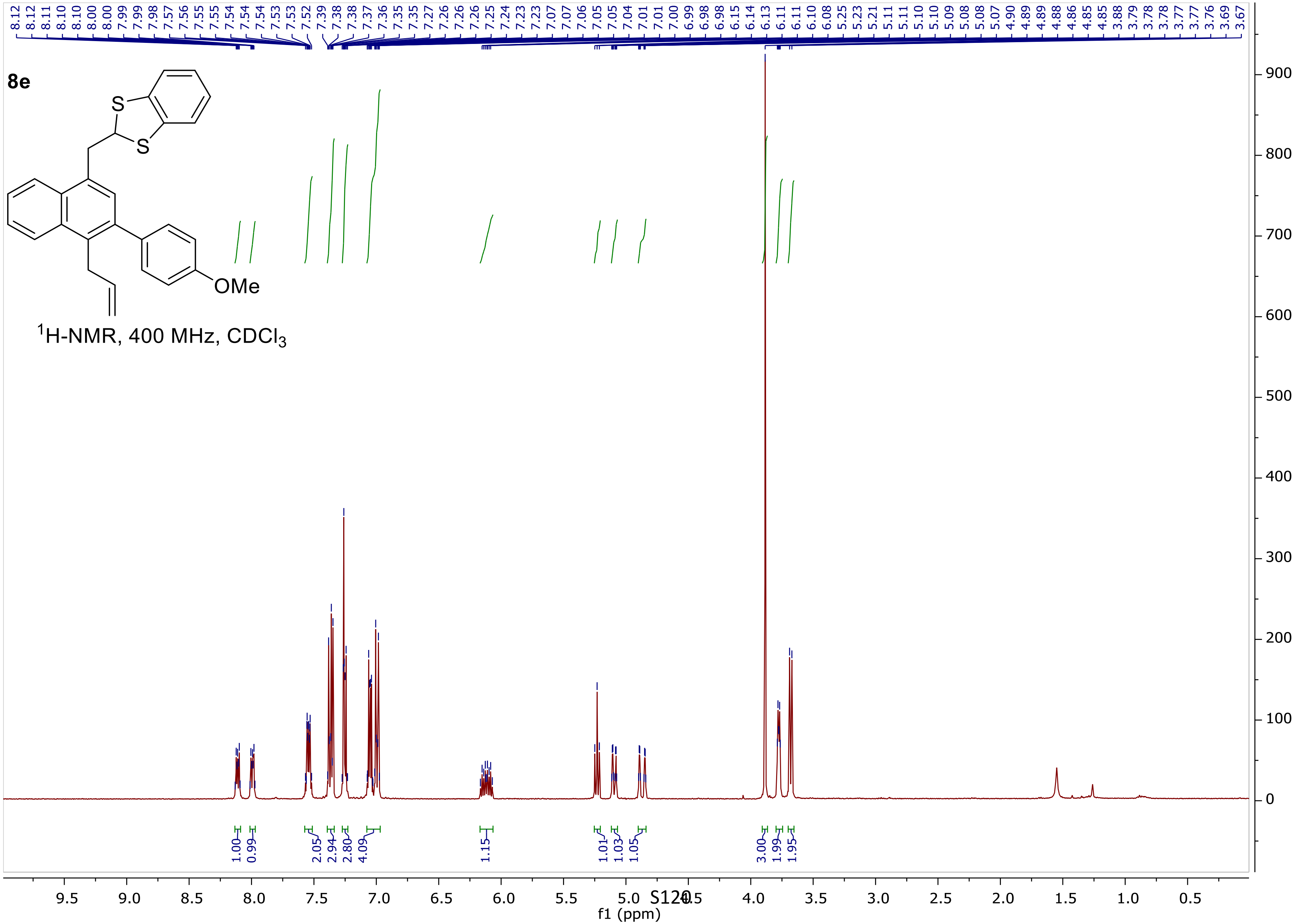
8e
S
S
OMe
1H-NMR, 400 MHz, CDCl3
f1 (ppm)

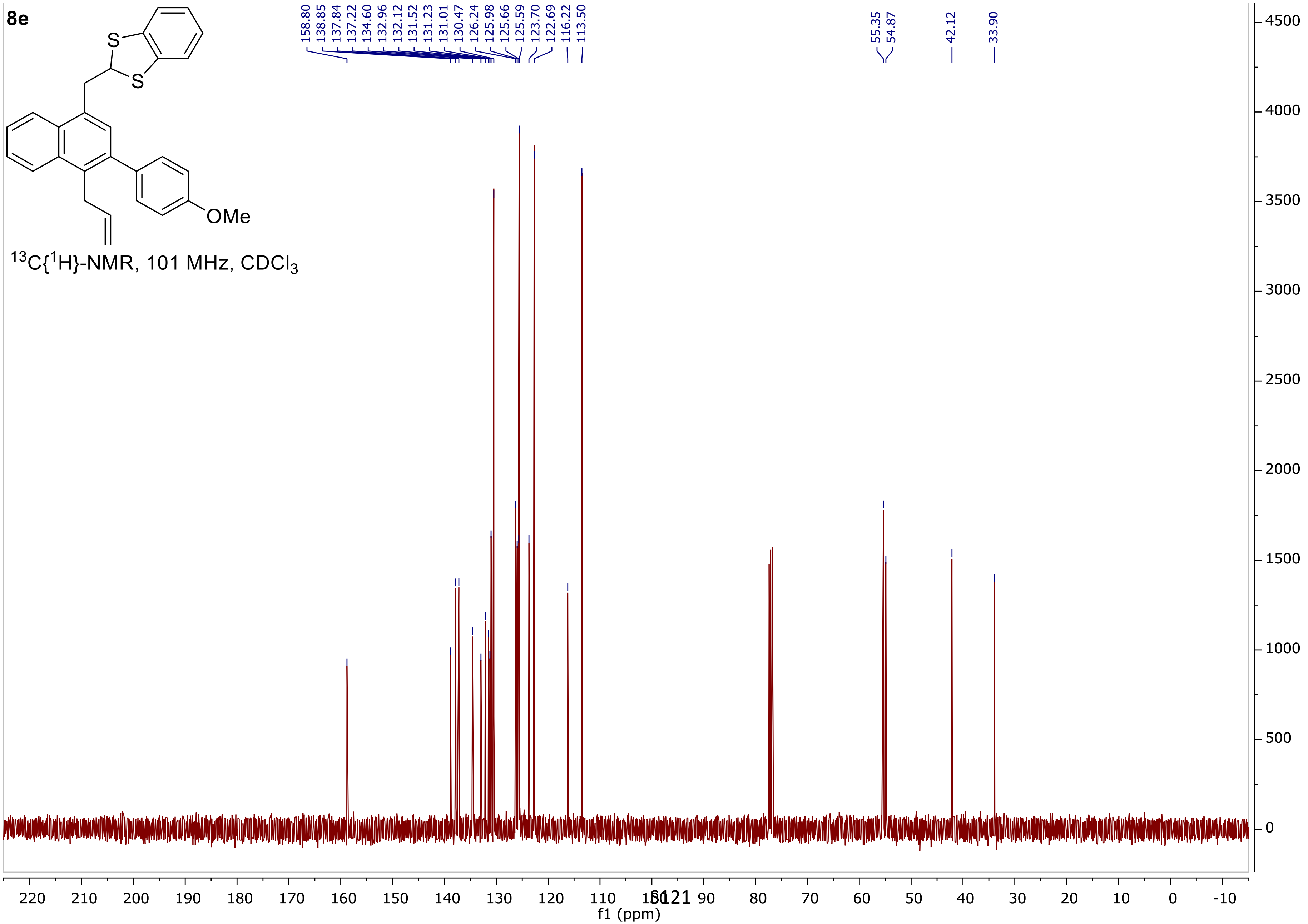
8e
S
S
OMe
$^{13}C\{^{1}H\}$-NMR, 101 MHz, CDCl$_3$
158.80
138.85
137.84
137.22
134.60
132.96
132.12
131.52
131.23
131.01
130.47
126.24
125.98
125.66
125.59
123.70
122.69
116.22
113.50
55.35
54.87
42.12
33.90
f1 (ppm)

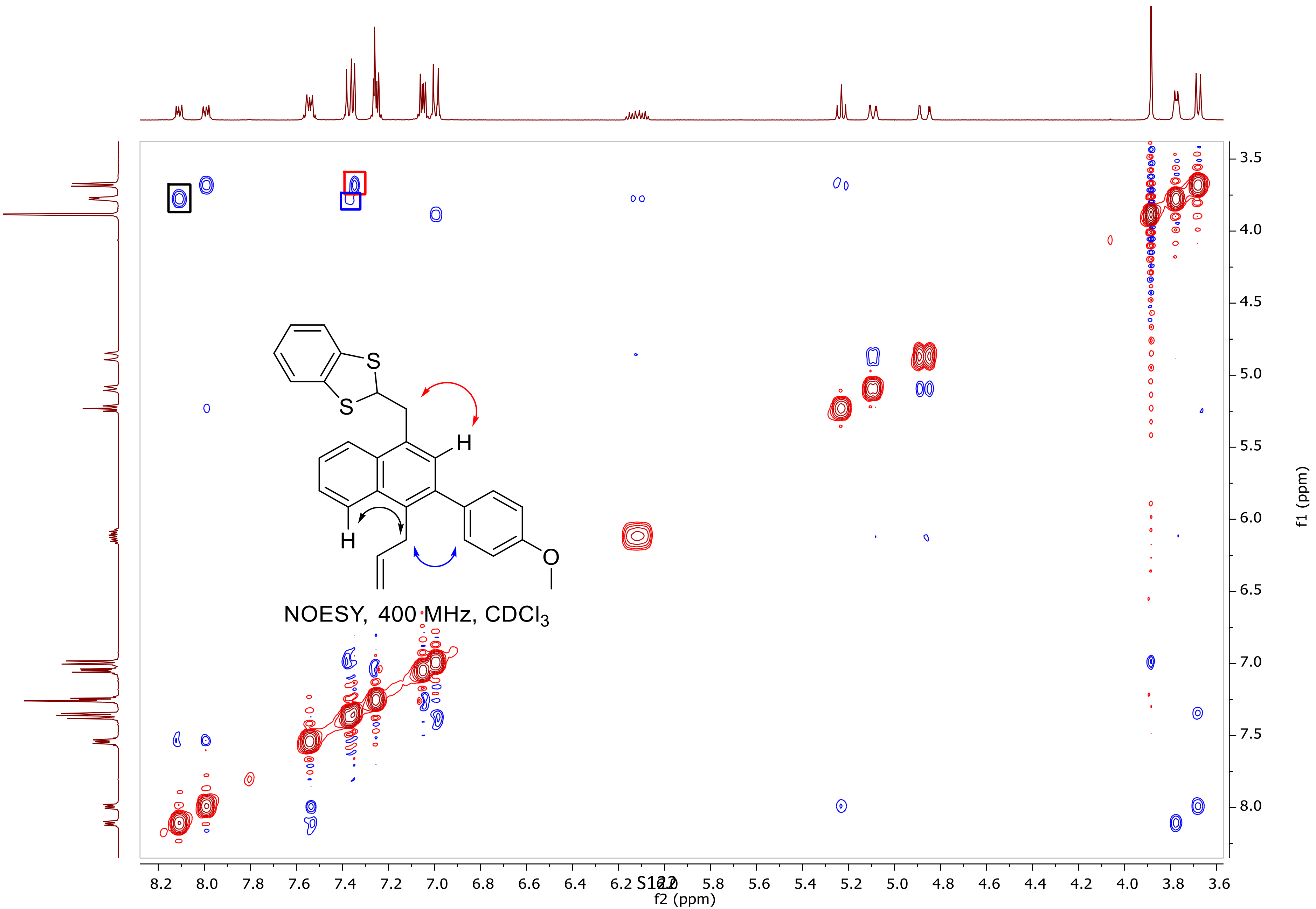

S
S
H
H
O
NOESY, 400 MHz, CDCl3
f2 (ppm)
f1 (ppm)

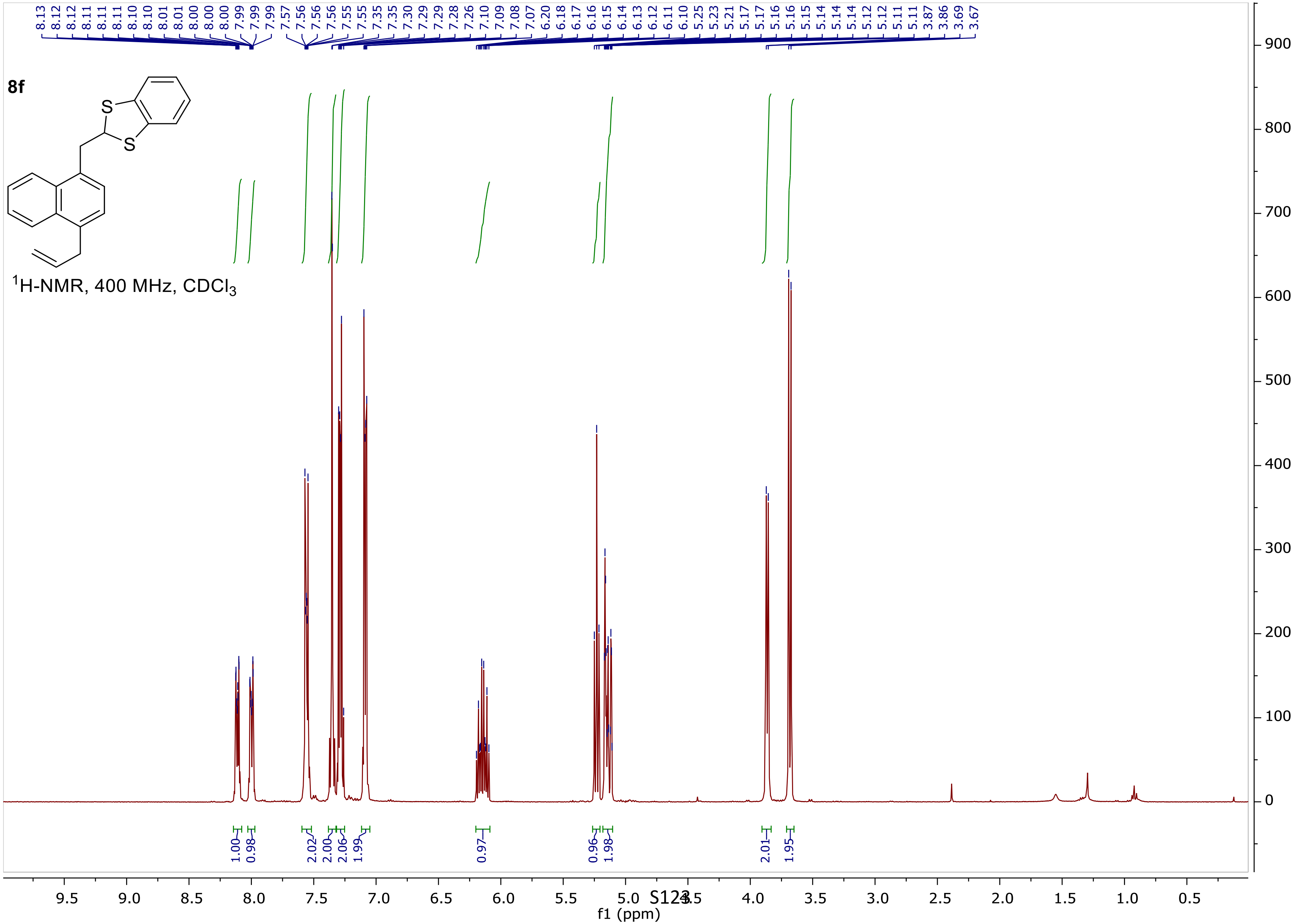
8f
S
S
^{1}H-NMR, 400 MHz, CDCl$_3$
8.13 8.12 8.12 8.11 8.11 8.10 8.10 8.01 8.01 8.00 8.00 7.99 7.99 7.57 7.56 7.56 7.55 7.55 7.35 7.35 7.30 7.29 7.29 7.28 7.26 7.10 7.09 7.08 7.07 6.20 6.18 6.17 6.16 6.15 6.14 6.13 6.12 6.11 6.10 5.25 5.23 5.21 5.17 5.17 5.16 5.16 5.15 5.14 5.14 5.14 5.12 5.11 5.11 3.87 3.86 3.69 3.67
1.00 0.98 2.02 2.00 2.06 1.99 0.97 0.96 1.98 2.01 1.95
f1 (ppm)

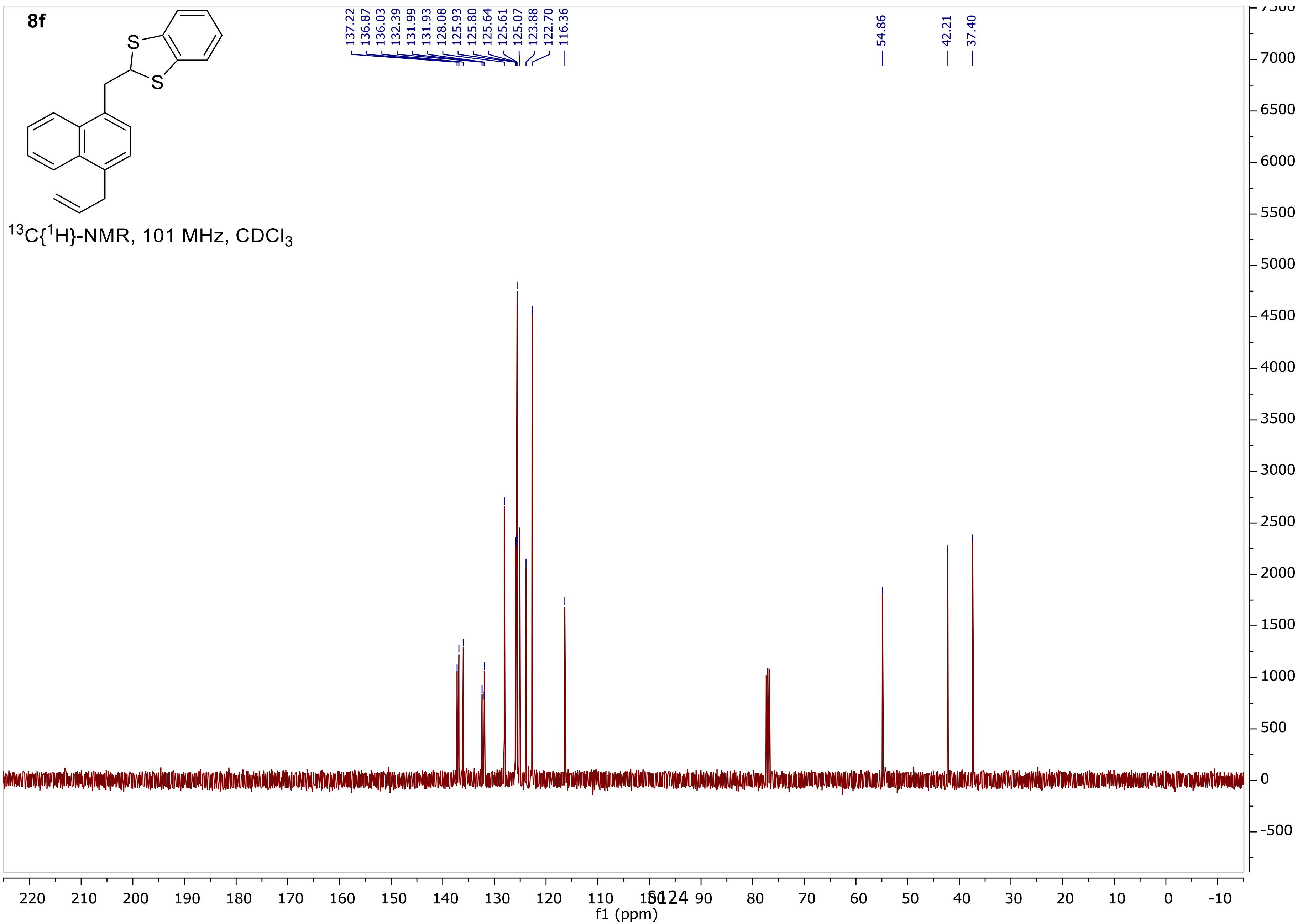
8f
S
S
$^{13}C\{^{1}H\}$-NMR, 101 MHz, $CDCl_3$
137.22
136.87
136.03
132.39
131.99
131.93
128.08
125.93
125.80
125.64
125.61
125.07
123.88
122.70
116.36
54.86
42.21
37.40
7500
7000
6500
6000
5500
5000
4500
4000
3500
3000
2500
2000
1500
1000
500
0
-500
220 210 200 190 180 170 160 150 140 130 120 110 100 90 80 70 60 50 40 30 20 10 0 -10
f1 (ppm)

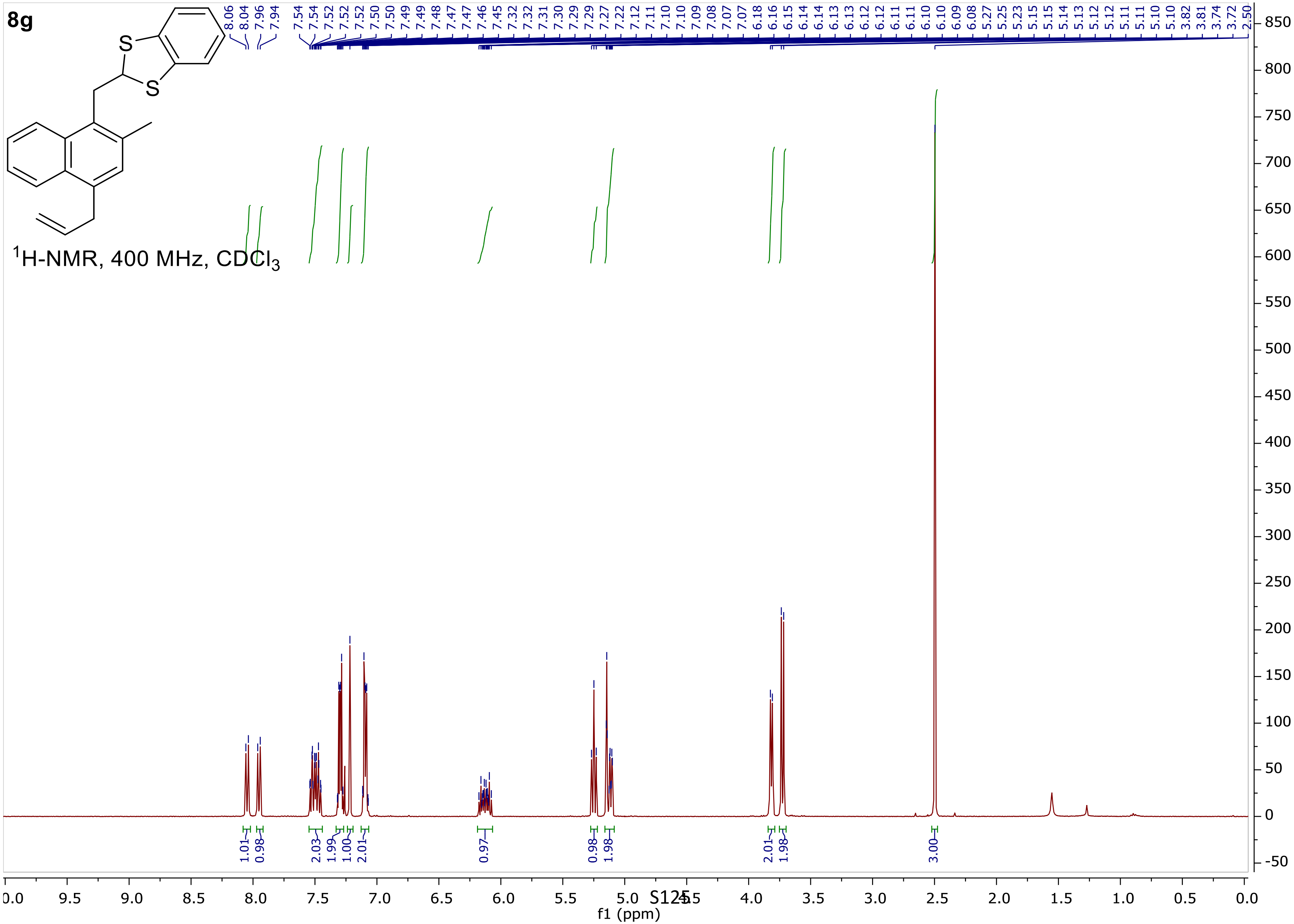

8g
1H-NMR, 400 MHz, CDCl3
f1 (ppm)

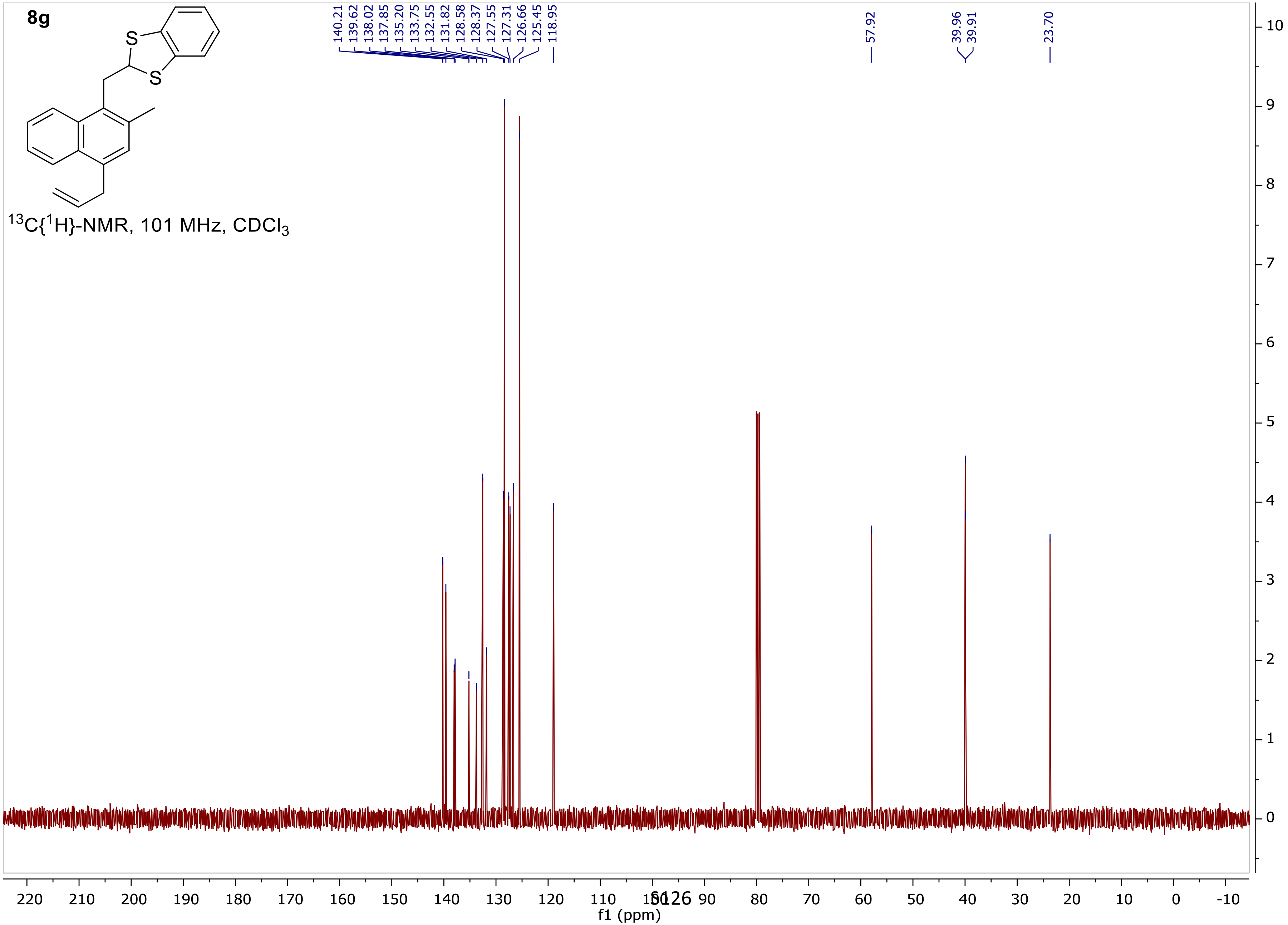
8g
S
S
13C{1H}-NMR, 101 MHz, CDCl3
140.21
139.62
138.02
137.85
135.20
133.75
132.55
131.82
128.58
128.37
127.55
127.31
126.66
125.45
118.95
57.92
39.96
39.91
23.70
f1 (ppm)

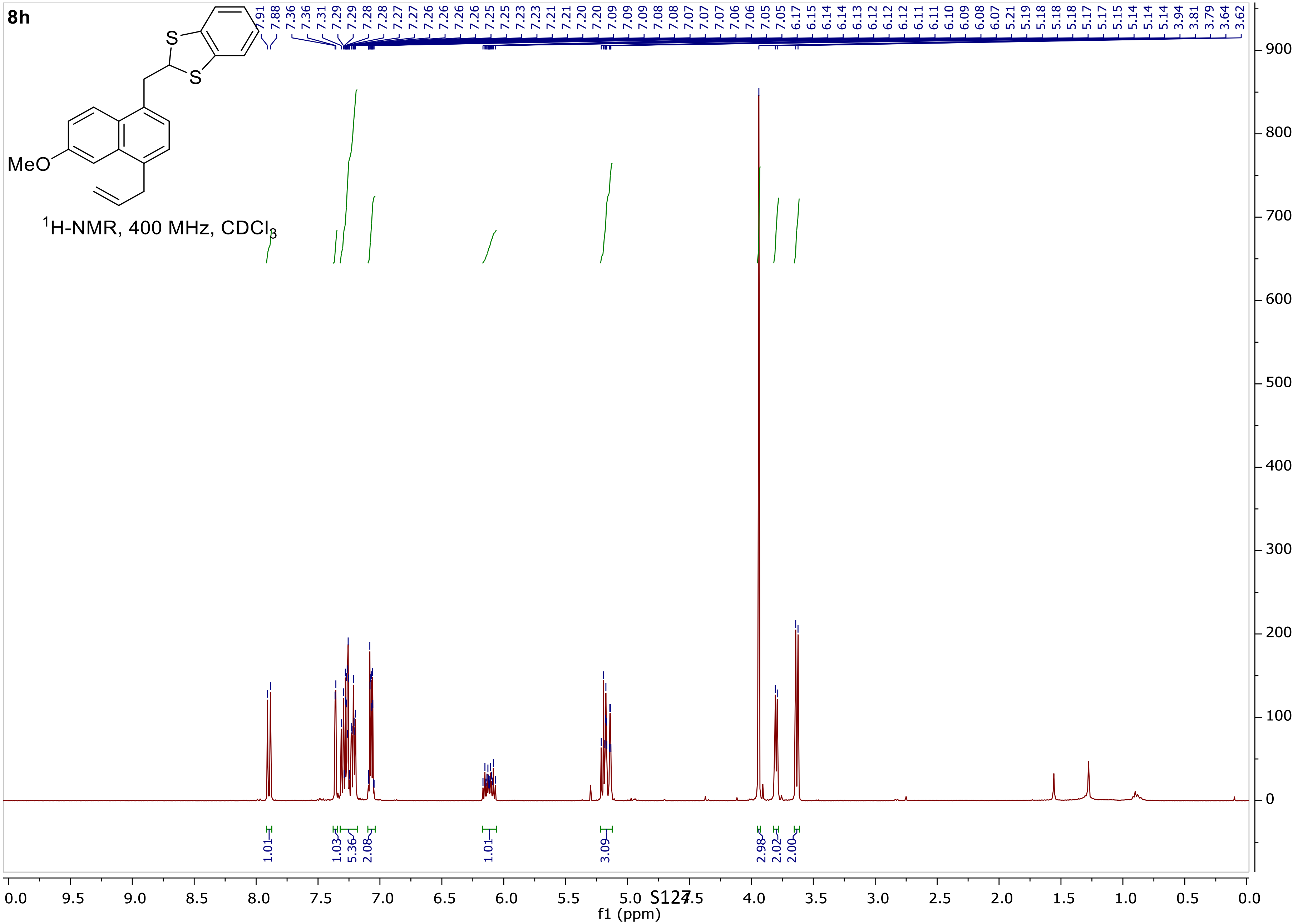

8h
MeO
S
S
1H-NMR, 400 MHz, CDCl3
f1 (ppm)

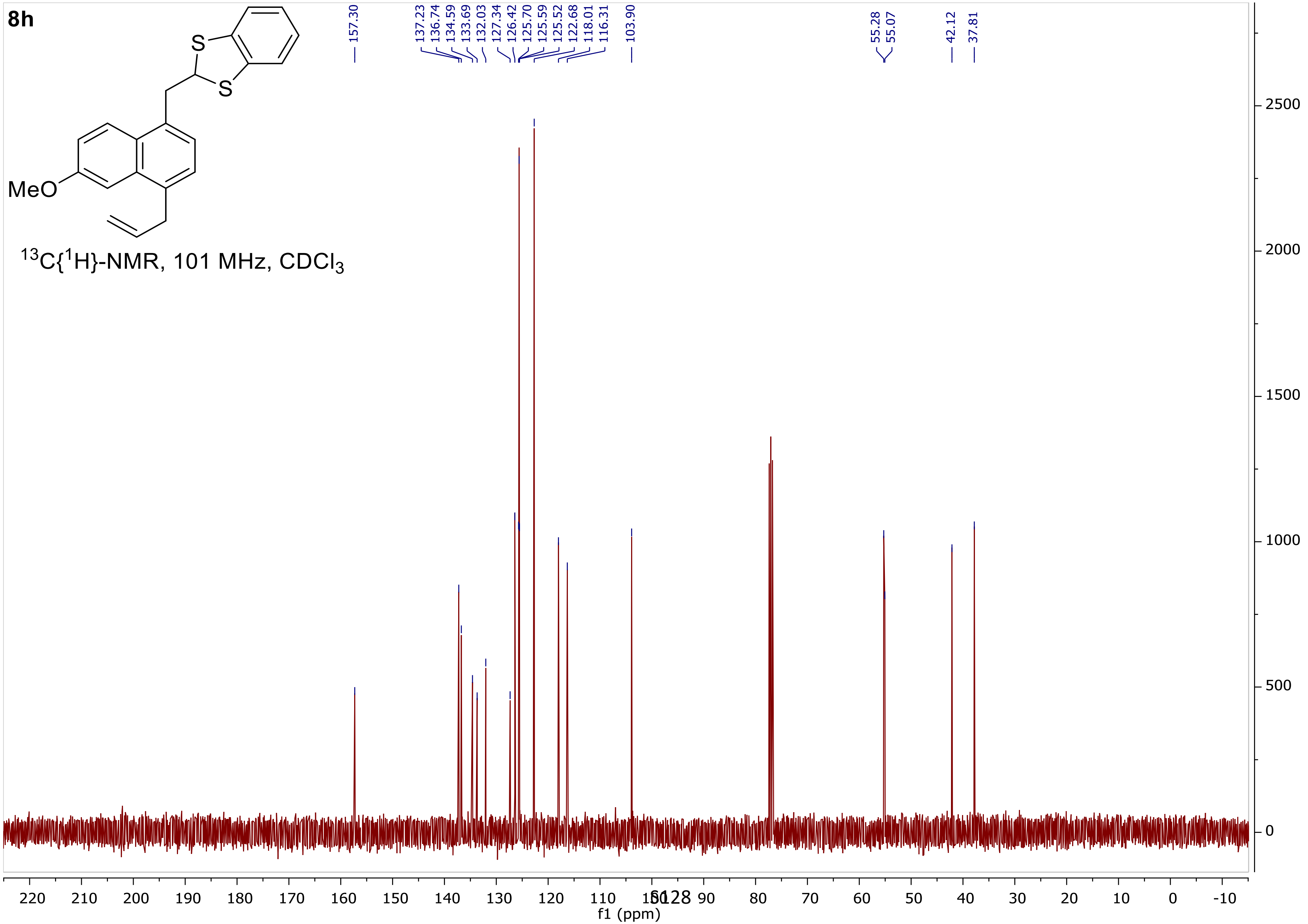
8h
MeO
S
S
13C{1H}-NMR, 101 MHz, CDCl3
157.30
137.23
136.74
134.59
133.69
132.03
127.34
126.42
125.70
125.59
125.52
122.68
118.01
116.31
103.90
55.28
55.07
42.12
37.81
2500
2000
1500
1000
500
0
220 210 200 190 180 170 160 150 140 130 120 110 100 90 80 70 60 50 40 30 20 10 0 -10
f1 (ppm)

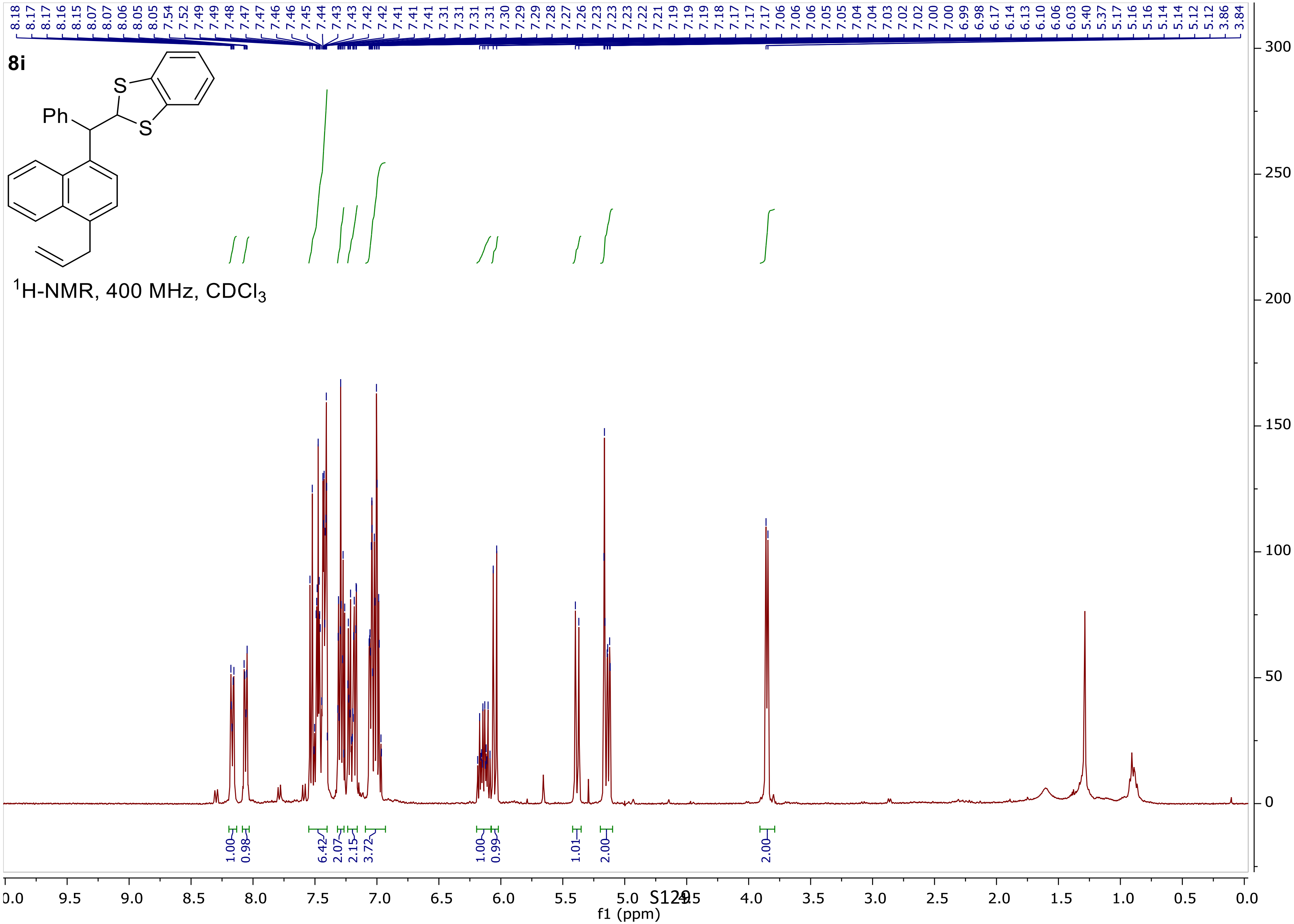
8.18 8.17 8.17 8.16 8.15 8.07 8.07 8.06 8.05 8.05 7.54 7.52 7.49 7.49 7.48 7.47 7.47 7.46 7.46 7.45 7.44 7.43 7.43 7.42 7.42 7.41 7.41 7.41 7.31 7.31 7.31 7.31 7.30 7.29 7.29 7.28 7.27 7.26 7.23 7.23 7.22 7.21 7.19 7.19 7.18 7.17 7.17 7.17 7.06 7.06 7.06 7.05 7.05 7.04 7.04 7.03 7.02 7.02 7.00 7.00 6.99 6.98 6.17 6.14 6.13 6.10 6.06 6.03 5.40 5.37 5.17 5.16 5.16 5.14 5.14 5.12 5.12 3.86 3.84
8i
Ph
S
S
1H-NMR, 400 MHz, CDCl3
1.00 0.98 6.42 2.07 2.15 3.72 1.00 0.99 1.01 2.00 2.00
f1 (ppm)

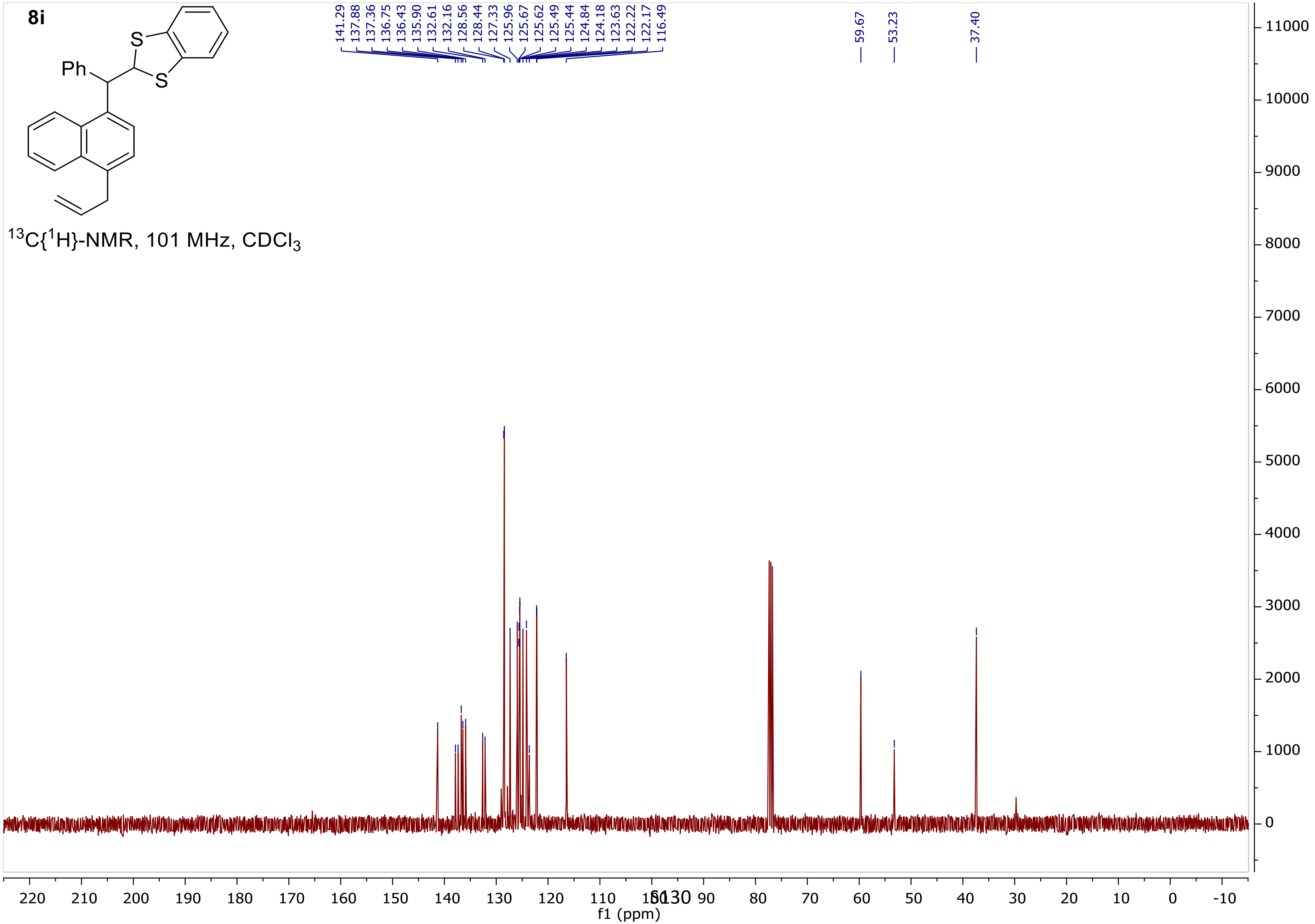
8i
Ph
S
S
13C{1H}-NMR, 101 MHz, CDCl3
141.29 137.88 137.36 136.75 136.43 135.90 132.61 132.16 128.56 128.44 127.33 125.96 125.67 125.62 125.49 125.44 124.84 124.18 123.63 122.22 122.17 116.49
59.67
53.23
37.40
f1 (ppm)

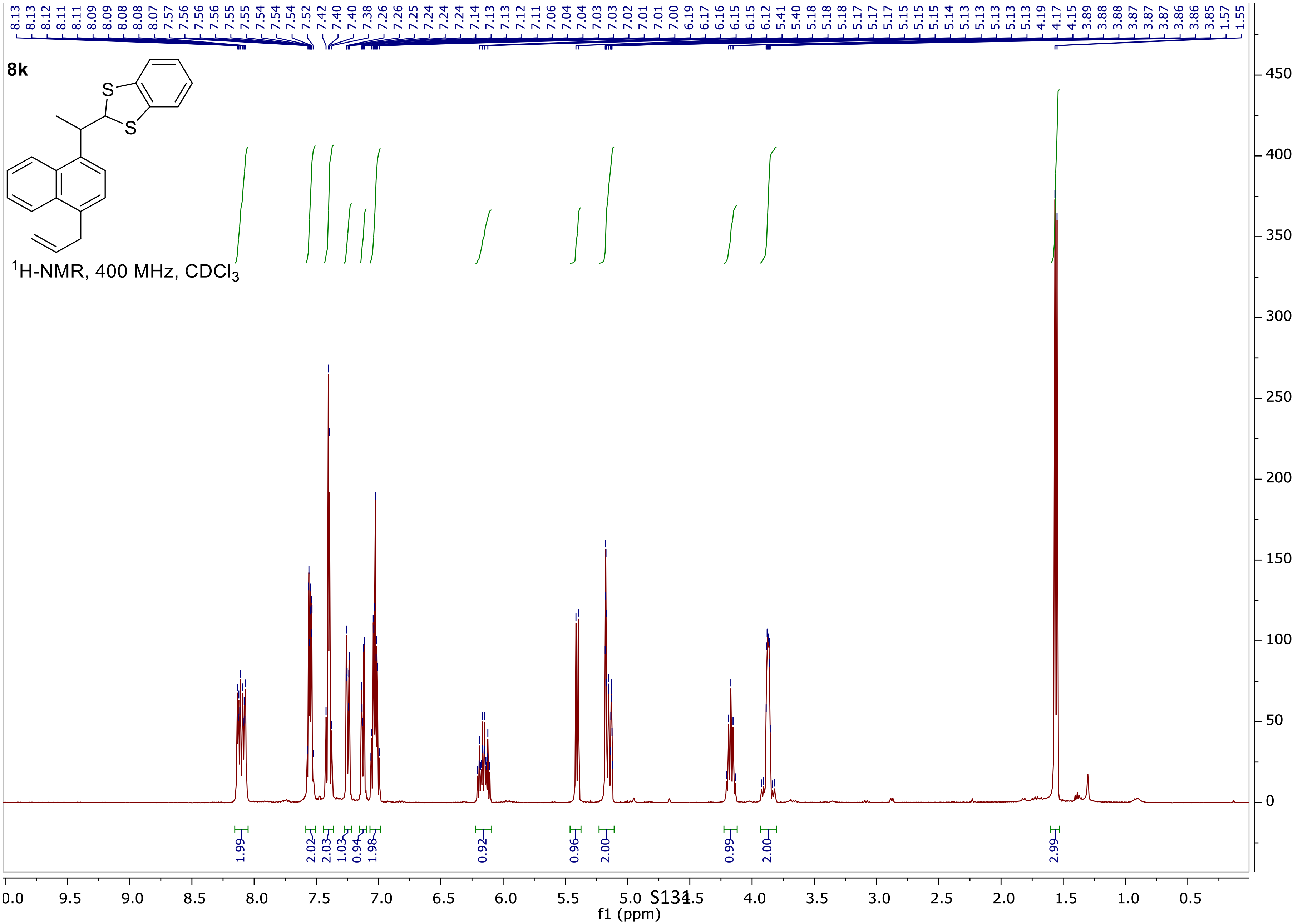
8k
S
S
^{1}H-NMR, 400 MHz, CDCl$_3$
1.99
2.02
2.03
1.03
0.94
1.98
0.92
0.96
2.00
0.99
2.00
2.99
f1 (ppm)

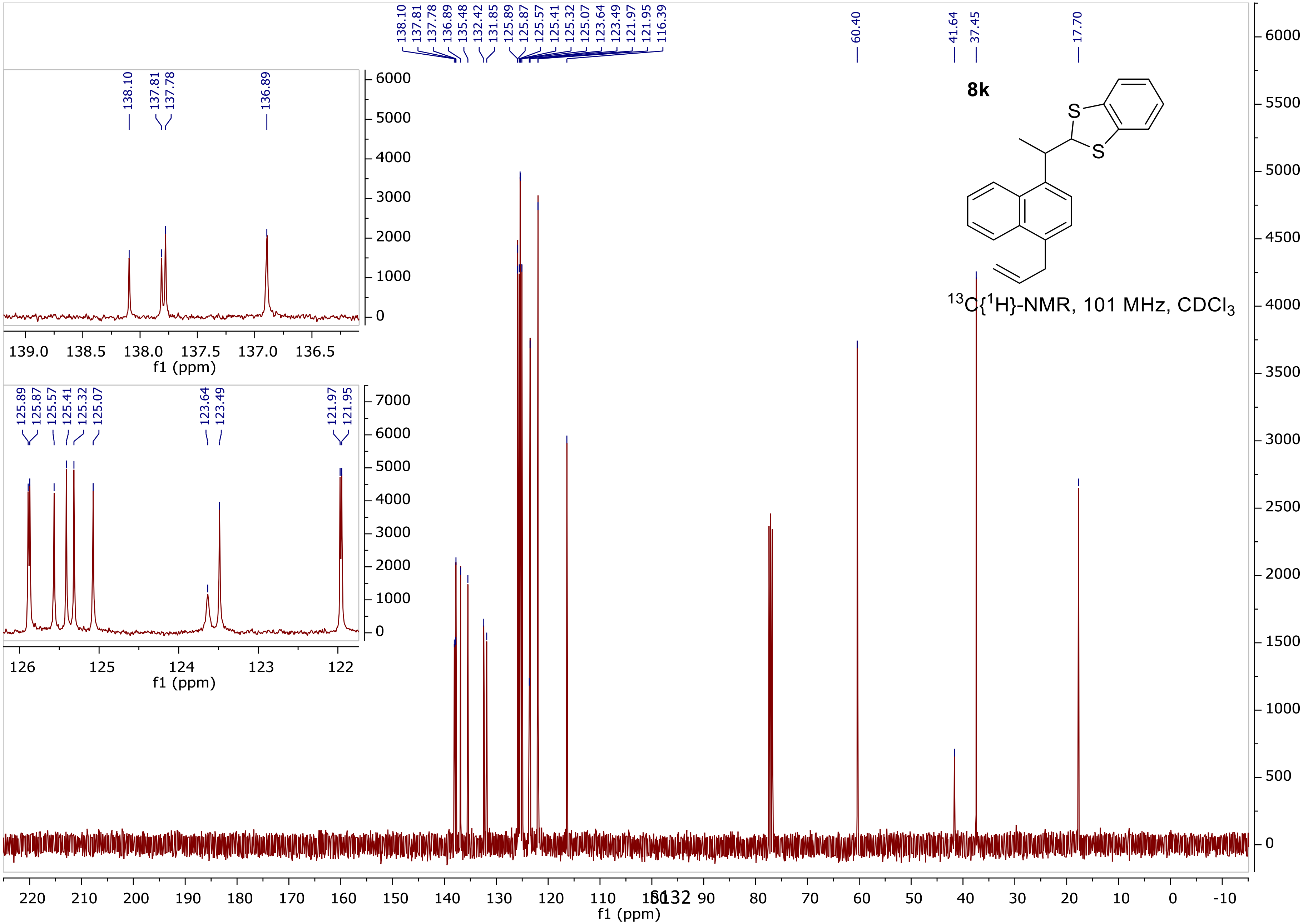

138.10
137.81
137.78
136.89
135.48
132.42
131.85
125.89
125.87
125.57
125.41
125.32
125.07
123.64
123.49
121.97
121.95
116.39
60.40
41.64
37.45
17.70
8k
S
S
$^{13}C\{^1H\}$-NMR, 101 MHz, $CDCl_3$
f1 (ppm)

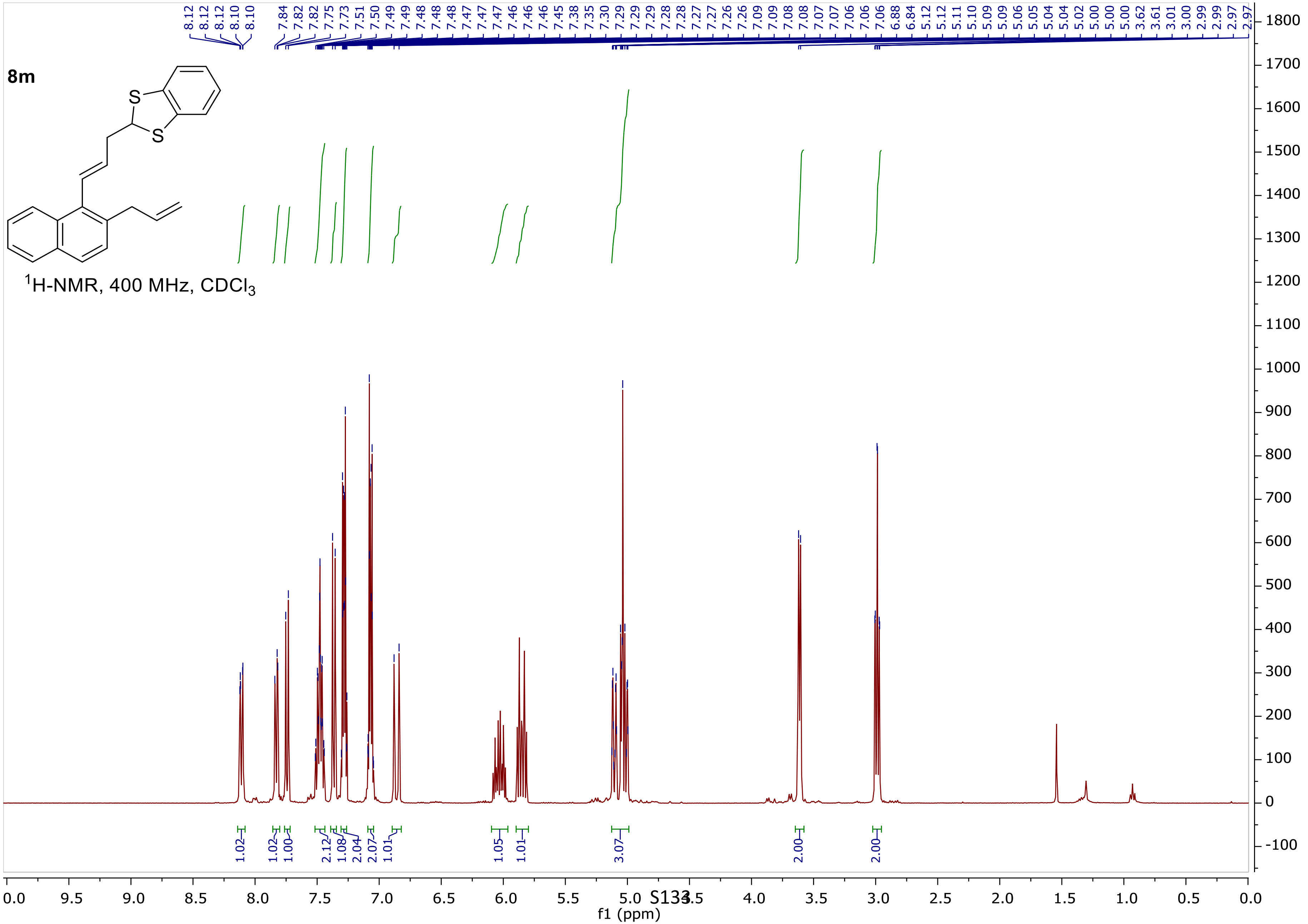
8m
^{1}H-NMR, 400 MHz, CDCl$_3$
f1 (ppm)

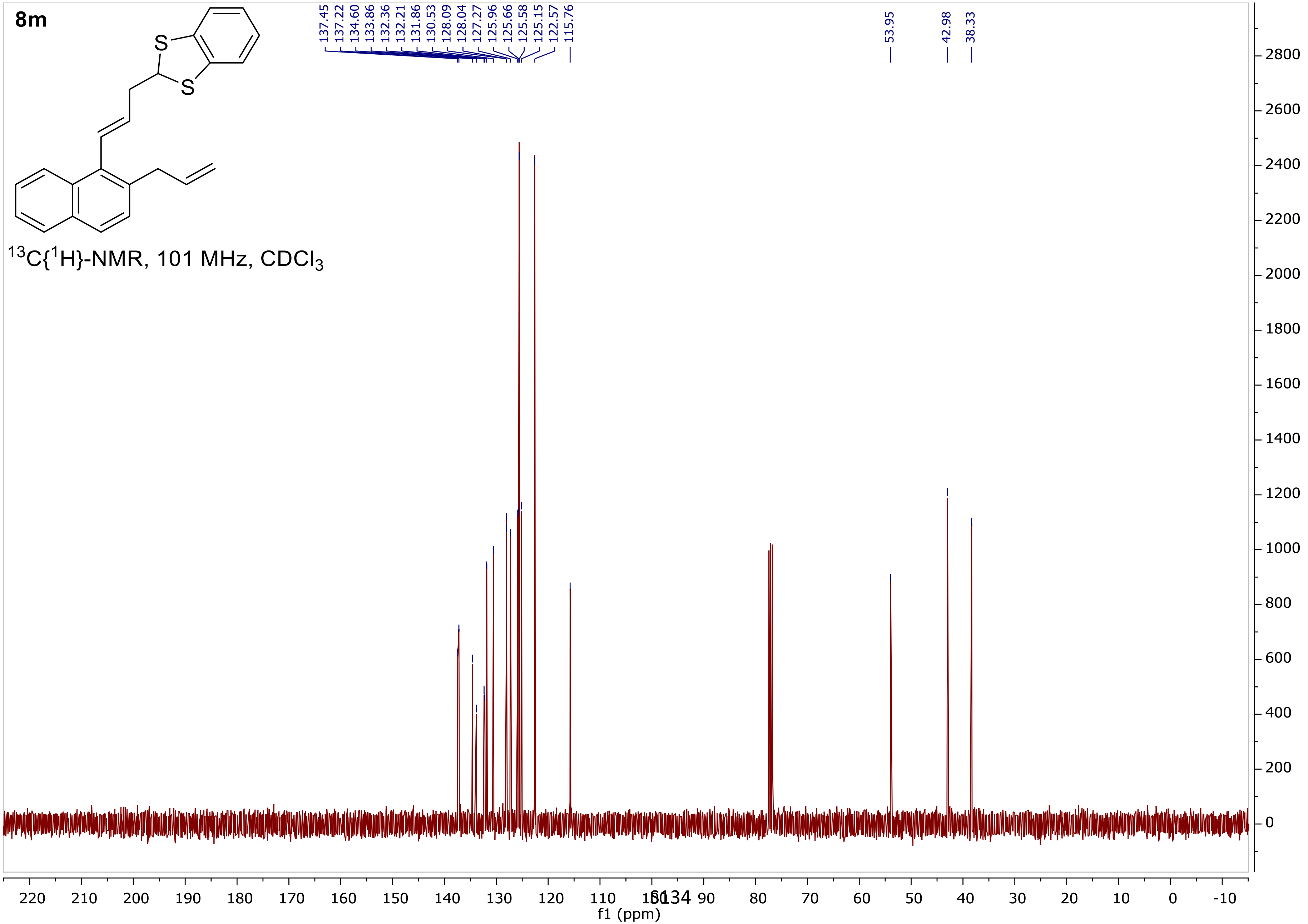
8m
S
S
13C{1H}-NMR, 101 MHz, CDCl3
137.45
137.22
134.60
133.86
132.36
132.21
131.86
130.53
128.09
128.04
127.27
125.96
125.66
125.58
125.15
122.57
115.76
53.95
42.98
38.33
f1 (ppm)

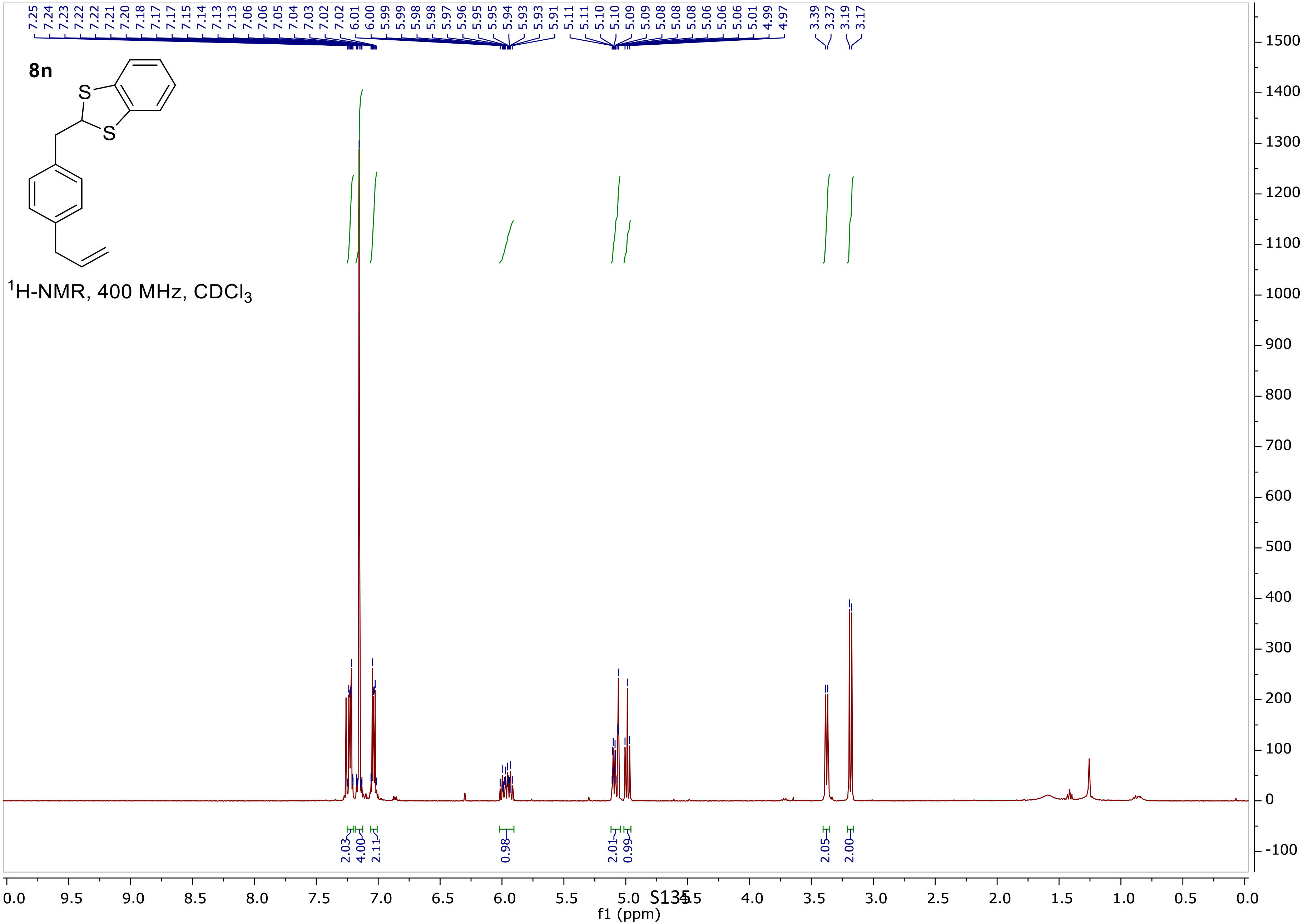

8n
S
S
1H-NMR, 400 MHz, CDCl3
f1 (ppm)

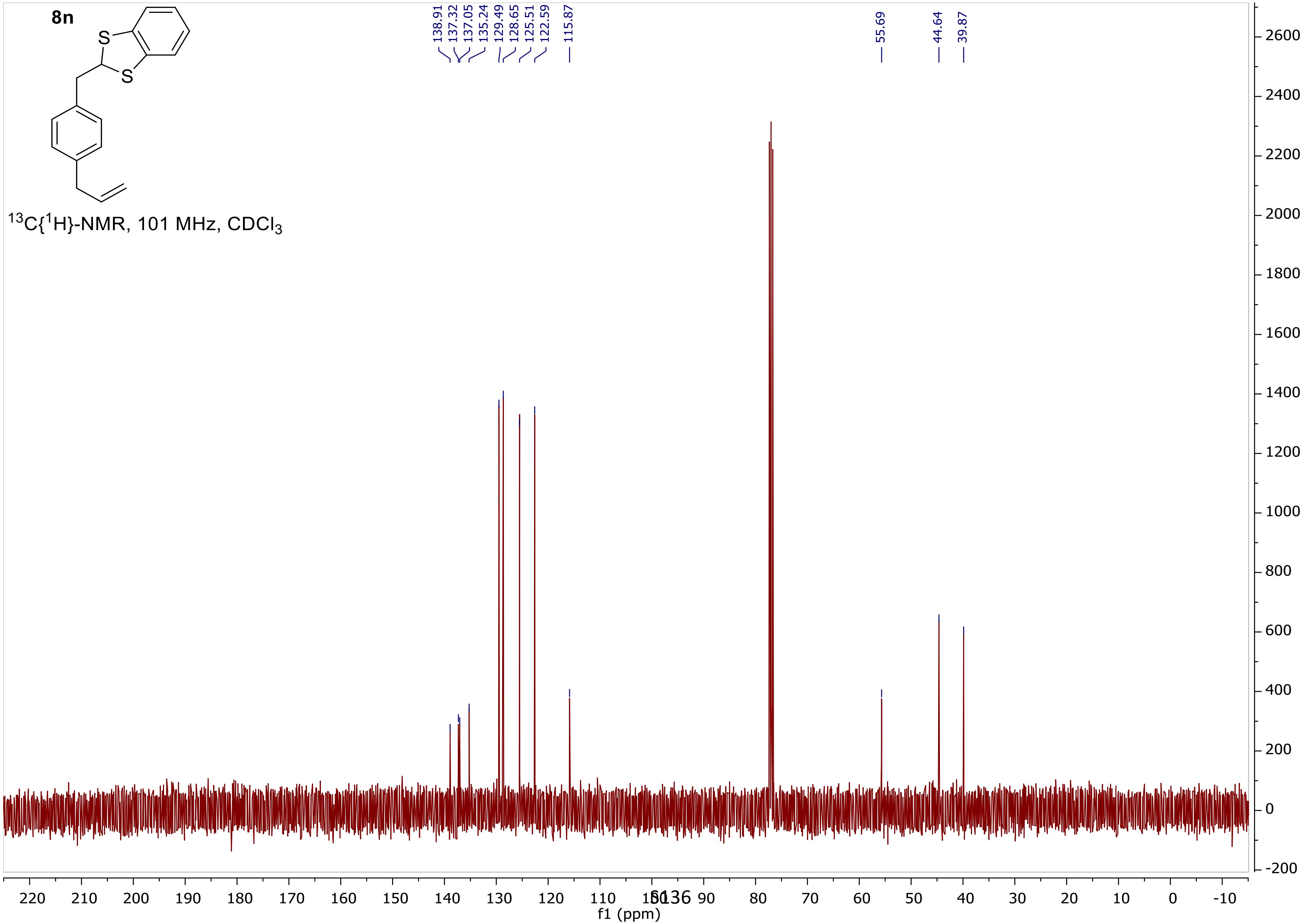
8n
S
S
13C{1H}-NMR, 101 MHz, CDCl3
138.91
137.32
137.05
135.24
129.49
128.65
125.51
122.59
115.87
55.69
44.64
39.87
2600
2400
2200
2000
1800
1600
1400
1200
1000
800
600
400
200
0
-200
220 210 200 190 180 170 160 150 140 130 120 110 100 90 80 70 60 50 40 30 20 10 0 -10
f1 (ppm)

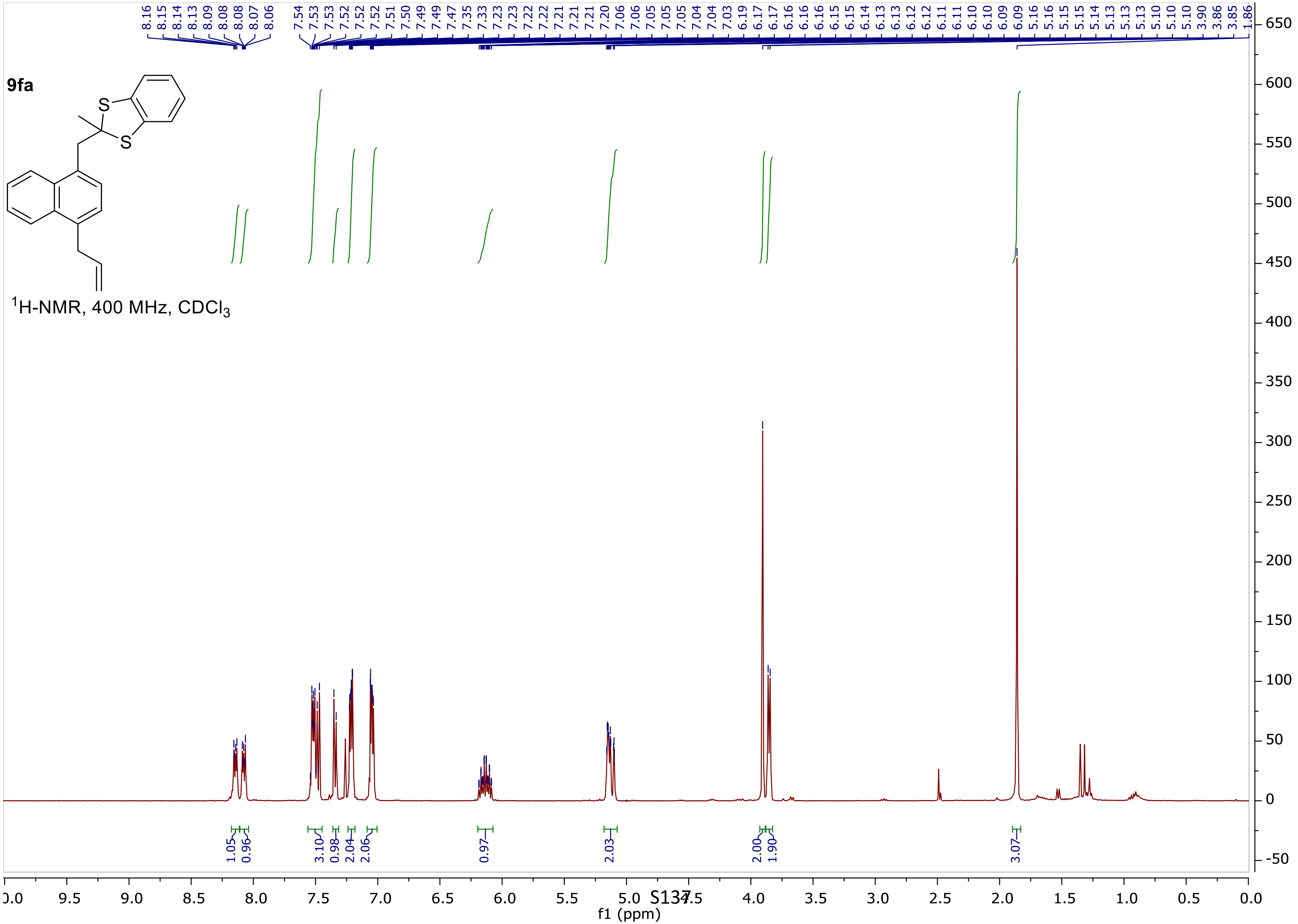

9fa

^{1}H-NMR, 400 MHz, $CDCl_3$

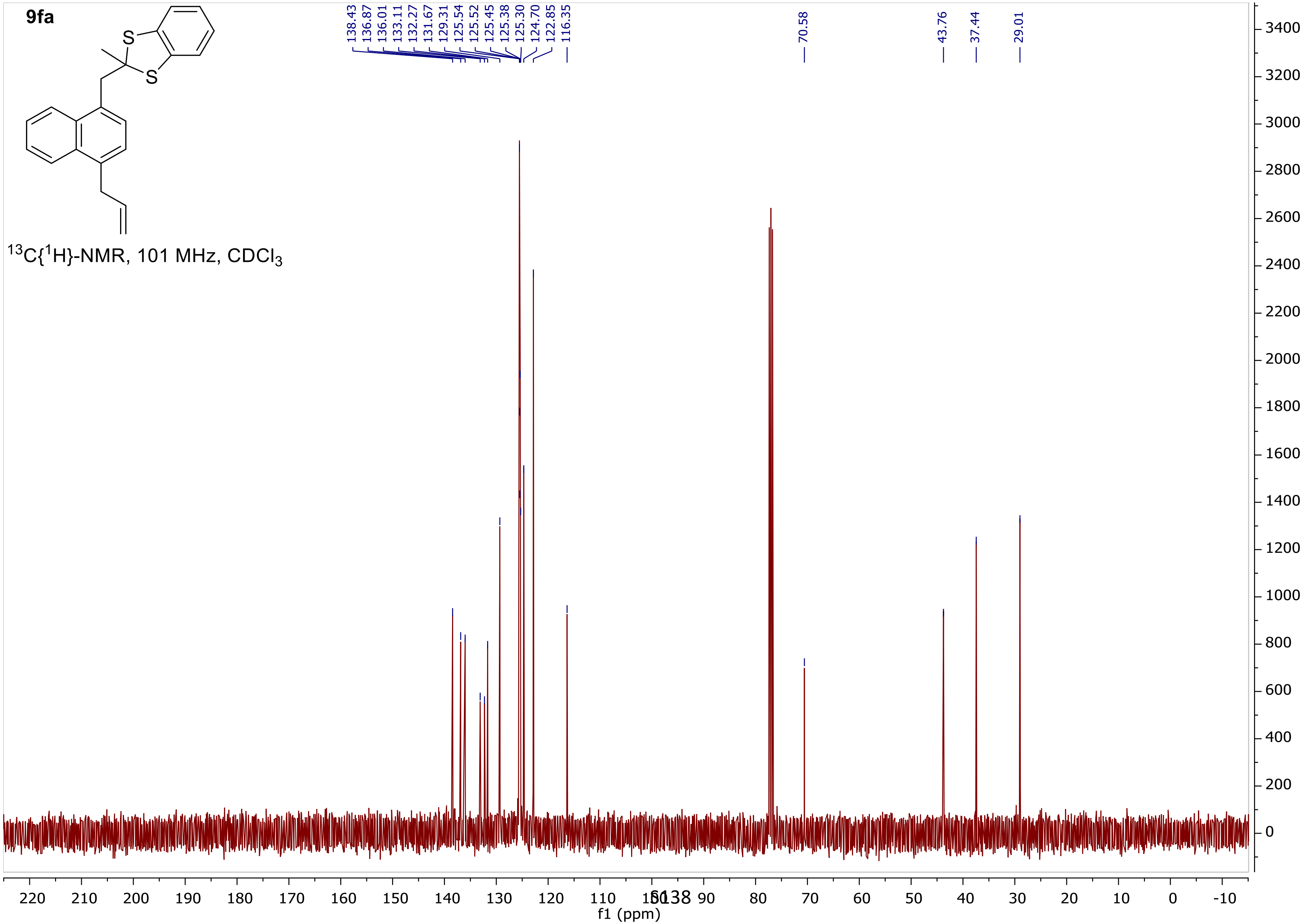
9fa
S
S
13C{1H}-NMR, 101 MHz, CDCl3
138.43
136.87
136.01
133.11
132.27
131.67
129.31
125.54
125.52
125.45
125.38
125.30
124.70
122.85
116.35
70.58
43.76
37.44
29.01
f1 (ppm)

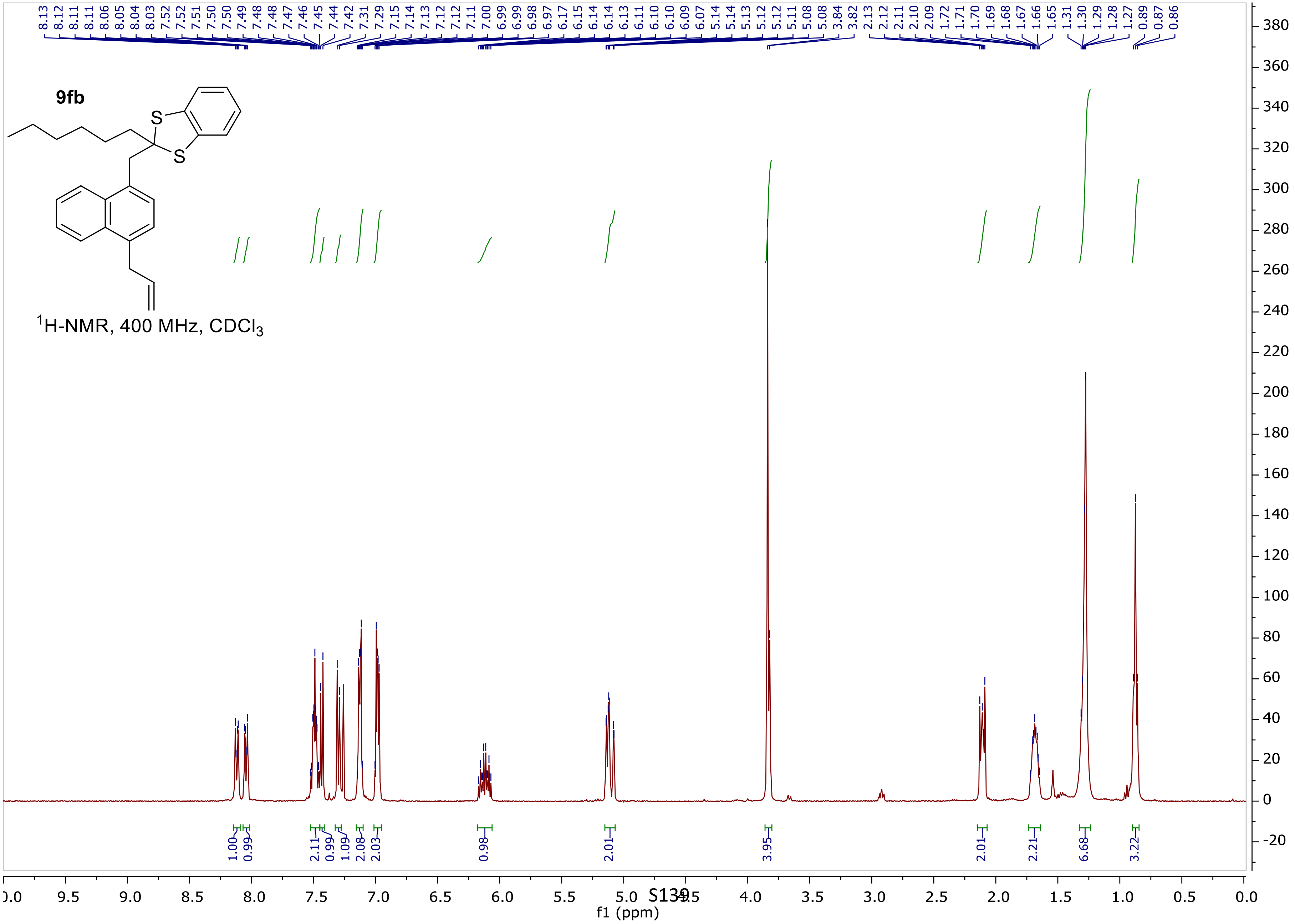
9fb
S
S
1H-NMR, 400 MHz, CDCl3
8.13 8.12 8.11 8.11 8.06 8.05 8.04 8.03 7.52 7.52 7.51 7.50 7.50 7.49 7.48 7.48 7.47 7.46 7.45 7.44 7.42 7.31 7.29 7.15 7.14 7.13 7.12 7.12 7.11 7.00 6.99 6.99 6.98 6.97 6.17 6.15 6.14 6.14 6.13 6.11 6.10 6.10 6.09 6.07 5.14 5.14 5.13 5.12 5.12 5.11 5.08 5.08 3.84 3.82 2.13 2.12 2.11 2.10 2.09 1.72 1.71 1.70 1.69 1.68 1.67 1.66 1.65 1.31 1.30 1.29 1.28 1.27 0.89 0.87 0.86
1.00 0.99 2.11 0.99 1.09 2.08 2.03 0.98 2.01 3.95 2.01 2.21 6.68 3.22
f1 (ppm)

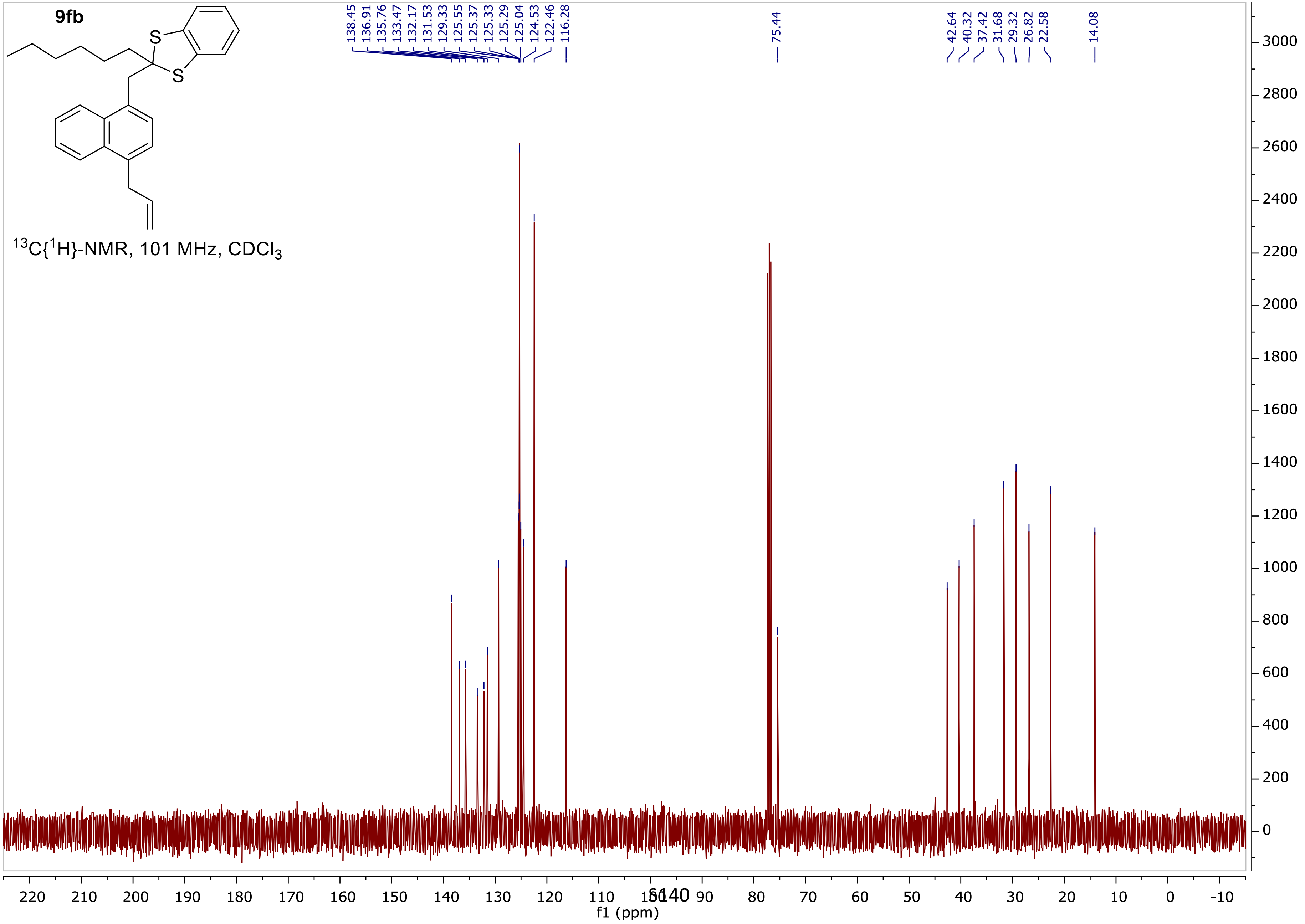
9fb
S
S
13C{1H}-NMR, 101 MHz, CDCl3
138.45
136.91
135.76
133.47
132.17
131.53
129.33
125.55
125.37
125.33
125.29
125.04
124.53
122.46
116.28
75.44
42.64
40.32
37.42
31.68
29.32
26.82
22.58
14.08
f1 (ppm)

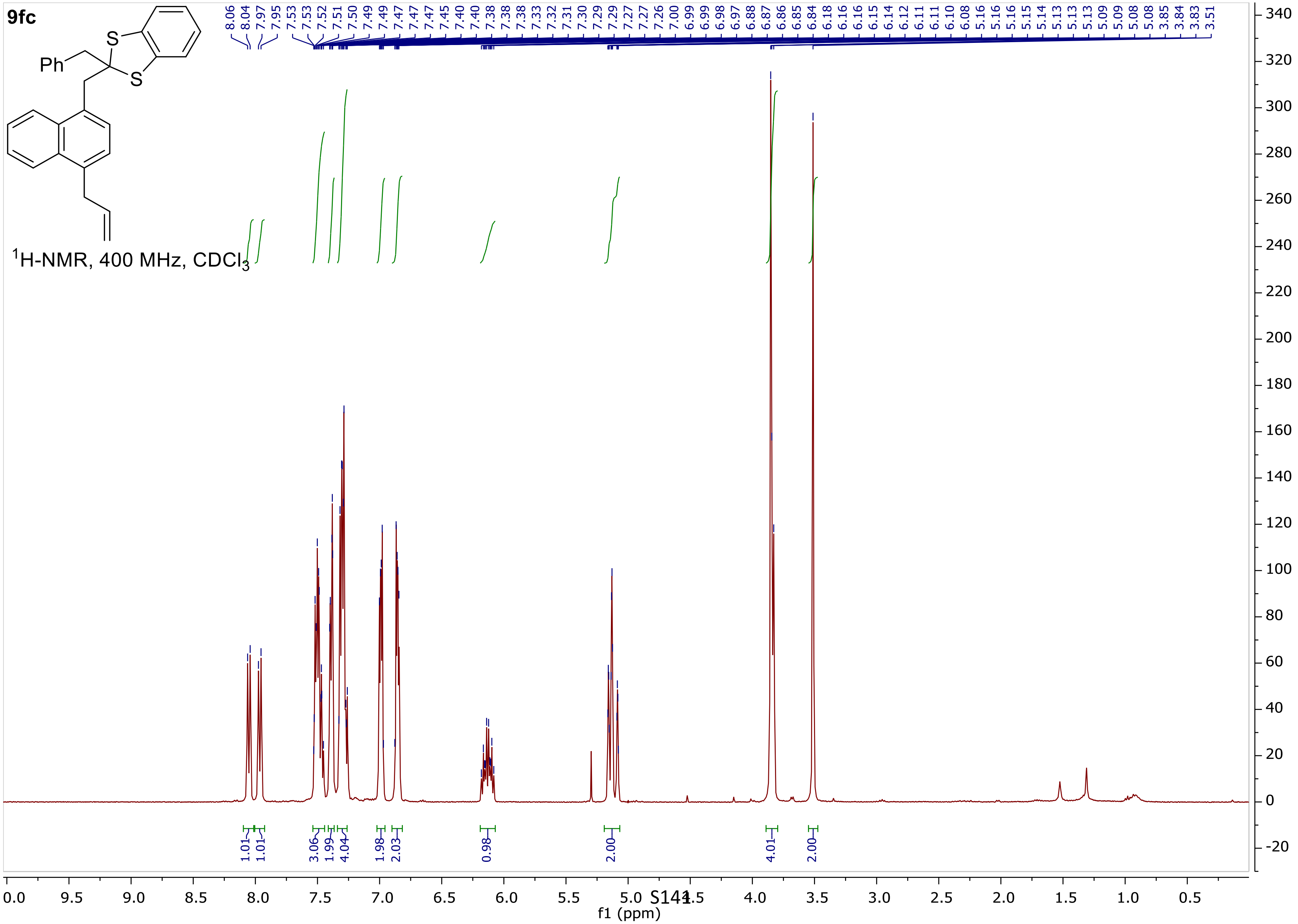
9fc
Ph
S
S
1H-NMR, 400 MHz, CDCl3
8.06 8.04 7.97 7.95 7.53 7.53 7.52 7.51 7.50 7.49 7.49 7.47 7.47 7.45 7.40 7.38 7.38 7.33 7.32 7.31 7.30 7.29 7.29 7.27 7.26 7.00 6.99 6.98 6.97 6.88 6.87 6.86 6.85 6.84 6.18 6.16 6.16 6.15 6.14 6.12 6.11 6.11 6.10 6.08 5.16 5.16 5.15 5.14 5.13 5.13 5.13 5.09 5.09 5.08 5.08 3.85 3.84 3.83 3.51
1.01 1.01 3.06 1.99 4.04 1.98 2.03 0.98 2.00 4.01 2.00
f1 (ppm)

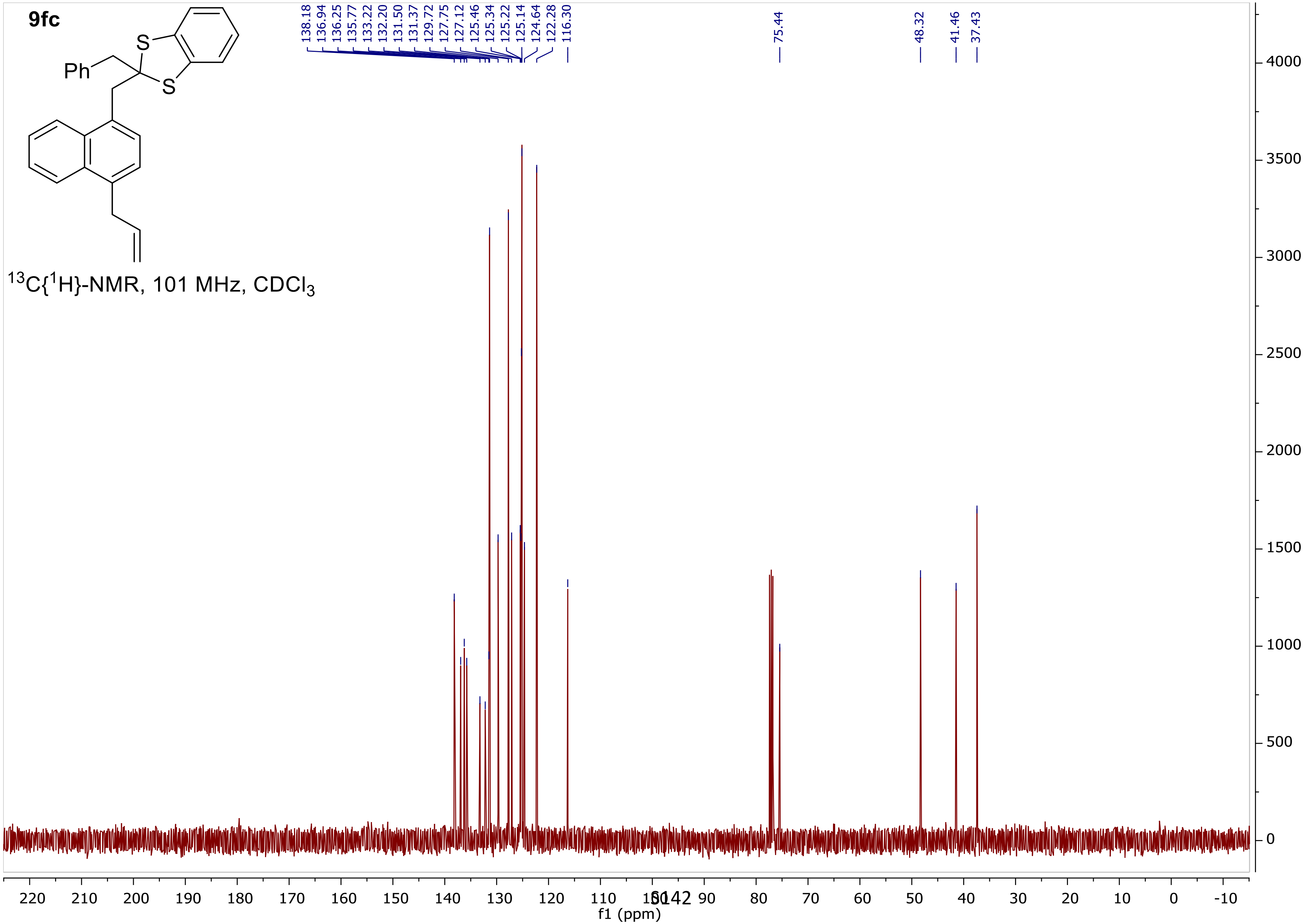
9fc
Ph
S
S
13C{1H}-NMR, 101 MHz, CDCl3
138.18
136.94
136.25
135.77
133.22
132.20
131.50
131.37
129.72
127.75
127.12
125.46
125.34
125.22
125.14
124.64
122.28
116.30
75.44
48.32
41.46
37.43
f1 (ppm)

9ka

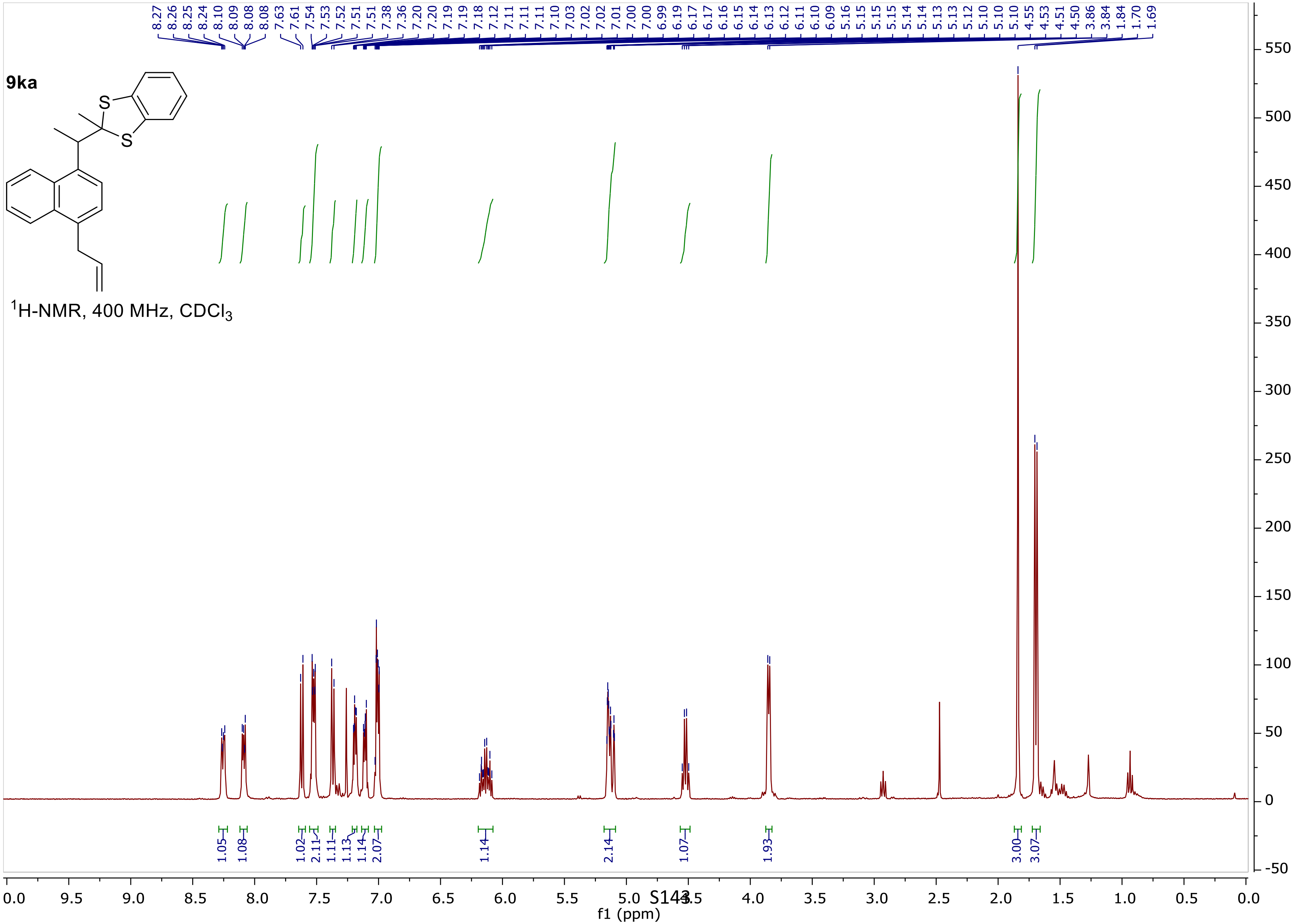

^{1}H-NMR, 400 MHz, $CDCl_3$

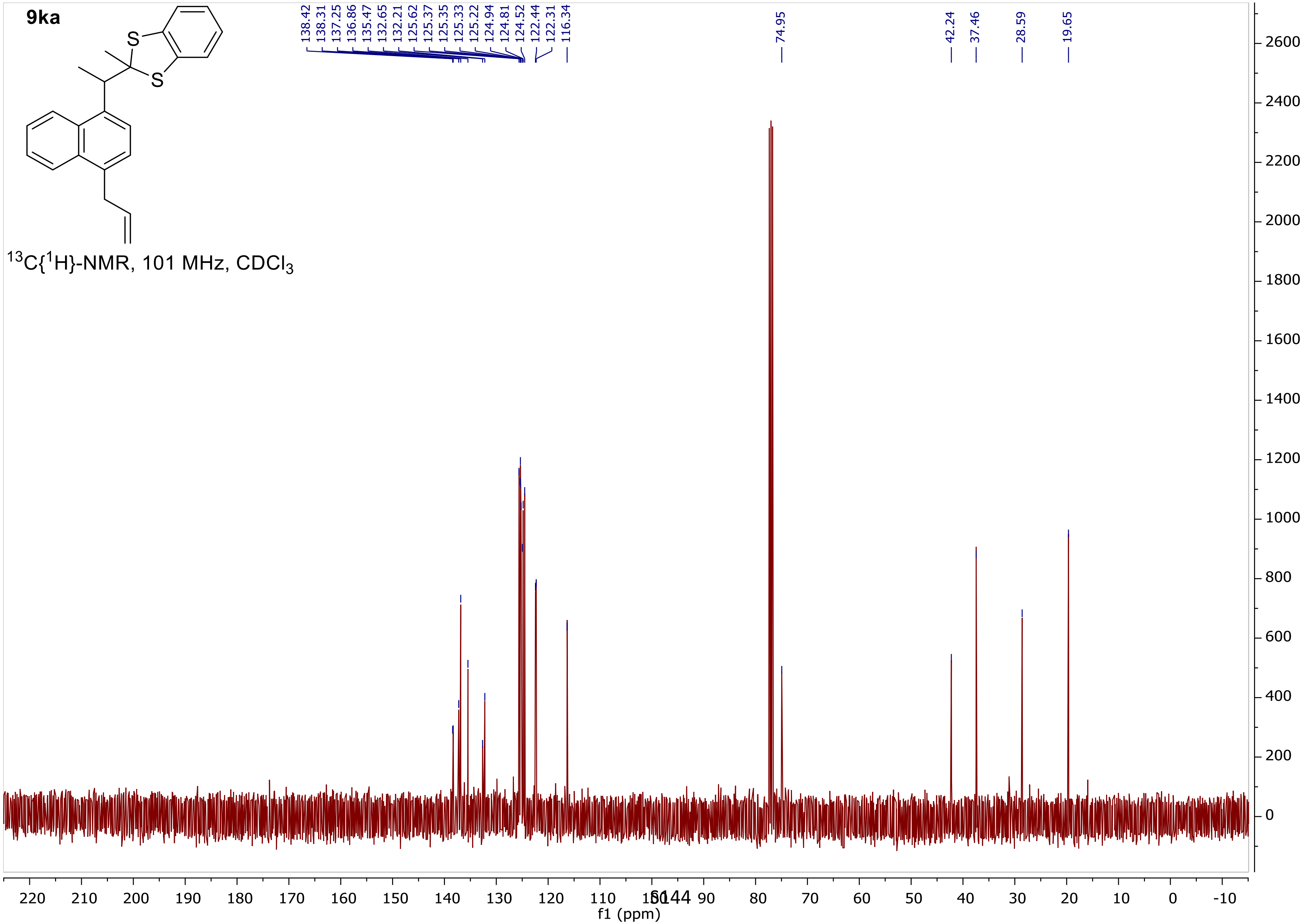
9ka
13C{1H}-NMR, 101 MHz, CDCl3
138.42
138.31
137.25
136.86
135.47
132.65
132.21
125.62
125.37
125.35
125.33
125.22
124.94
124.81
124.52
122.44
122.31
116.34
74.95
42.24
37.46
28.59
19.65
f1 (ppm)

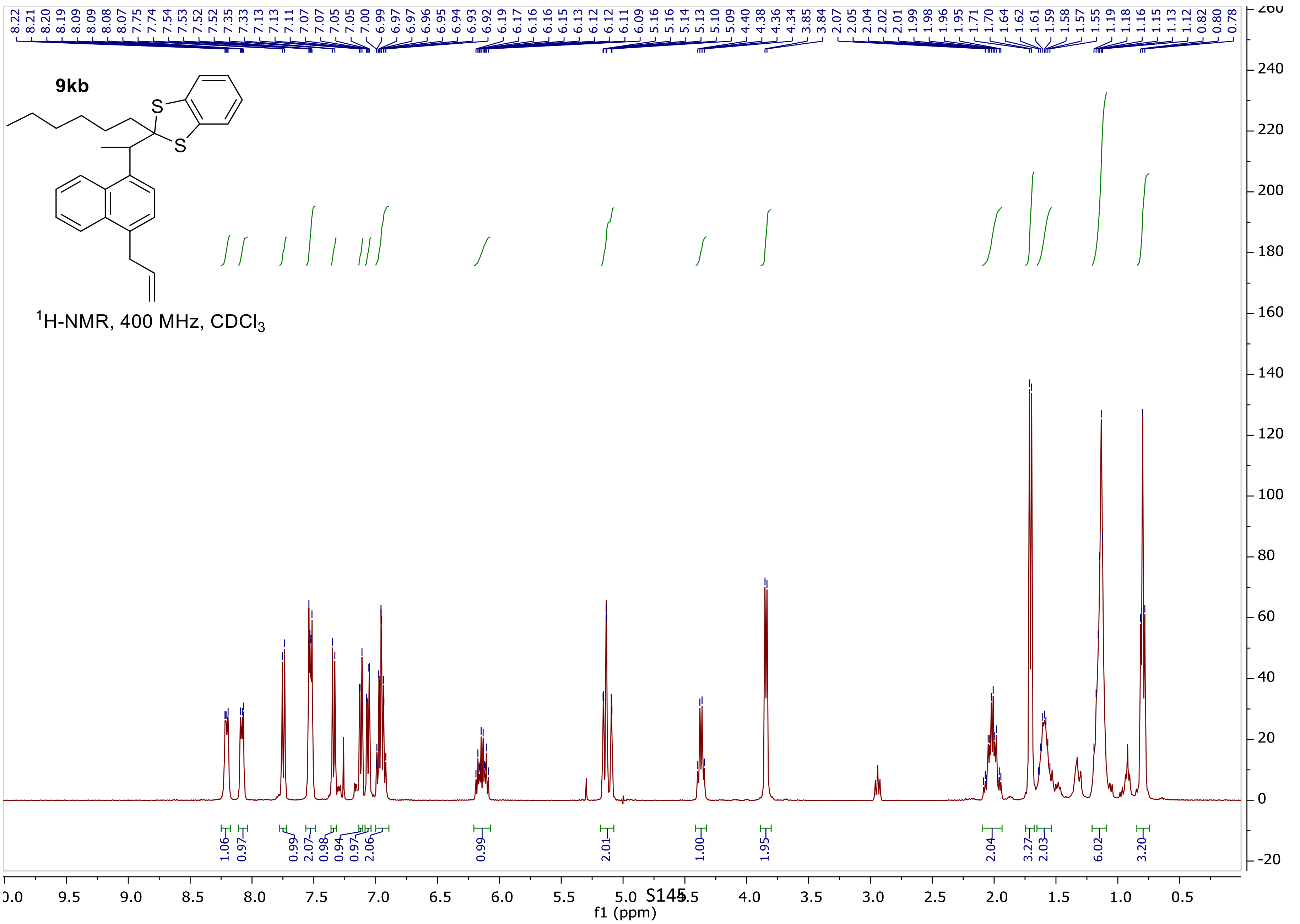
9kb
S
S
¹H-NMR, 400 MHz, CDCl₃
f1 (ppm)

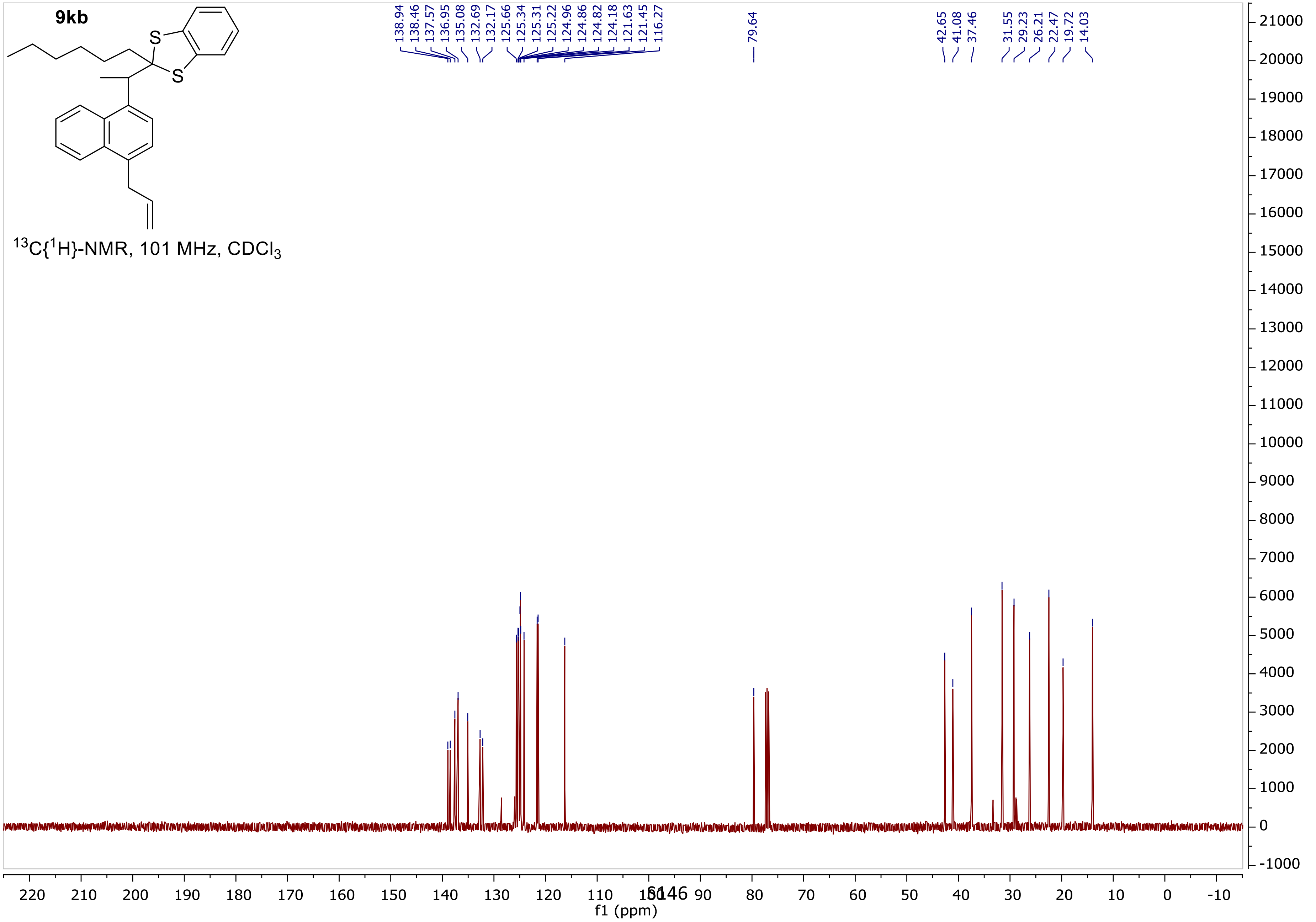
9kb
S
S
$^{13}C\{^1H\}$-NMR, 101 MHz, $CDCl_3$
138.94
138.46
137.57
136.95
135.08
132.69
132.17
125.66
125.34
125.31
125.22
124.96
124.86
124.82
124.18
121.63
121.45
116.27
79.64
42.65
41.08
37.46
31.55
29.23
26.21
22.47
19.72
14.03
f1 (ppm)

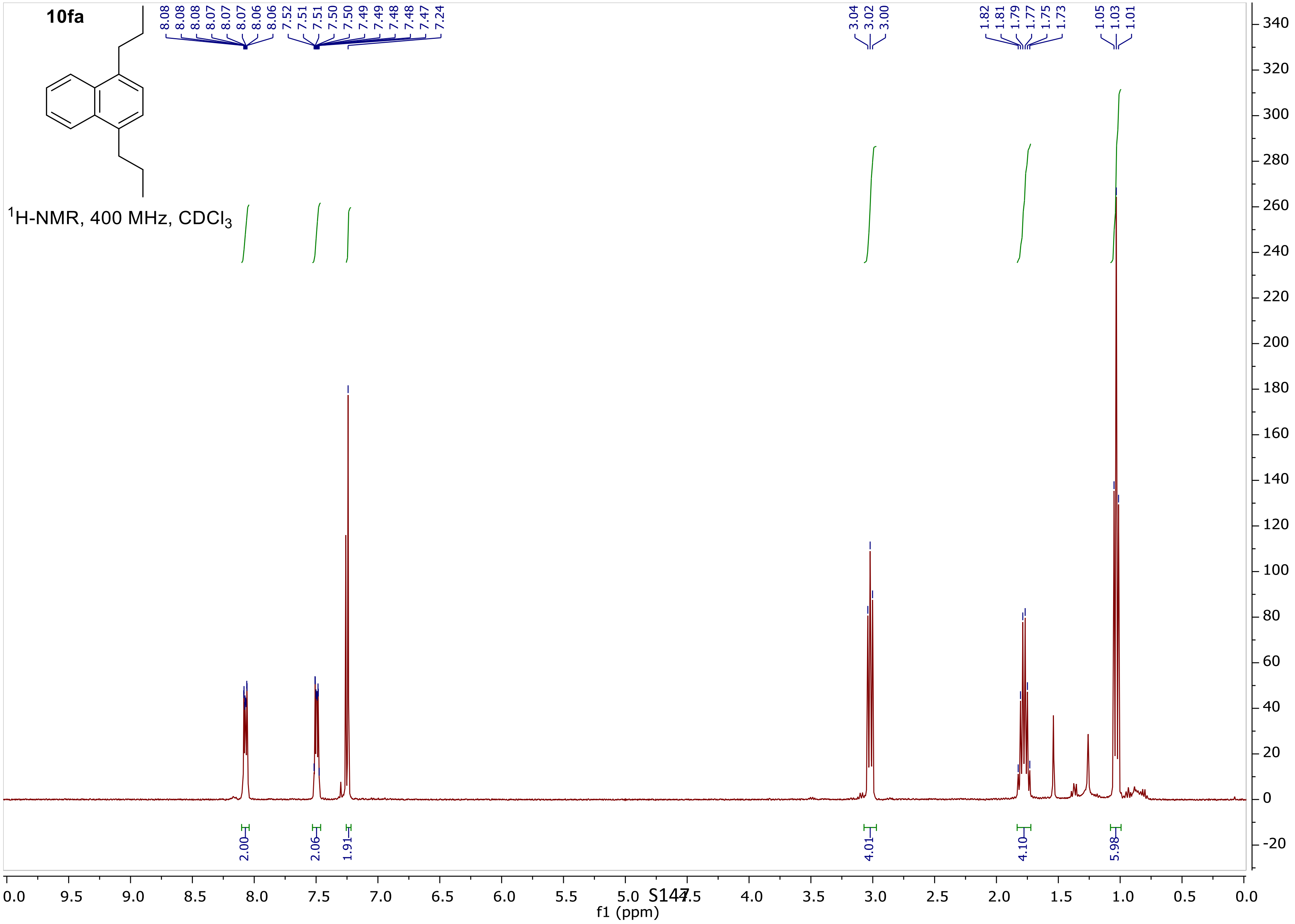

10fa
1H-NMR, 400 MHz, CDCl3
8.08 8.08 8.08 8.07 8.07 8.07 8.06 8.06 7.52 7.51 7.51 7.50 7.50 7.49 7.49 7.48 7.48 7.47 7.24
3.04 3.02 3.00
1.82 1.81 1.79 1.77 1.75 1.73
1.05 1.03 1.01
2.00 2.06 1.91 4.01 4.10 5.98
f1 (ppm)

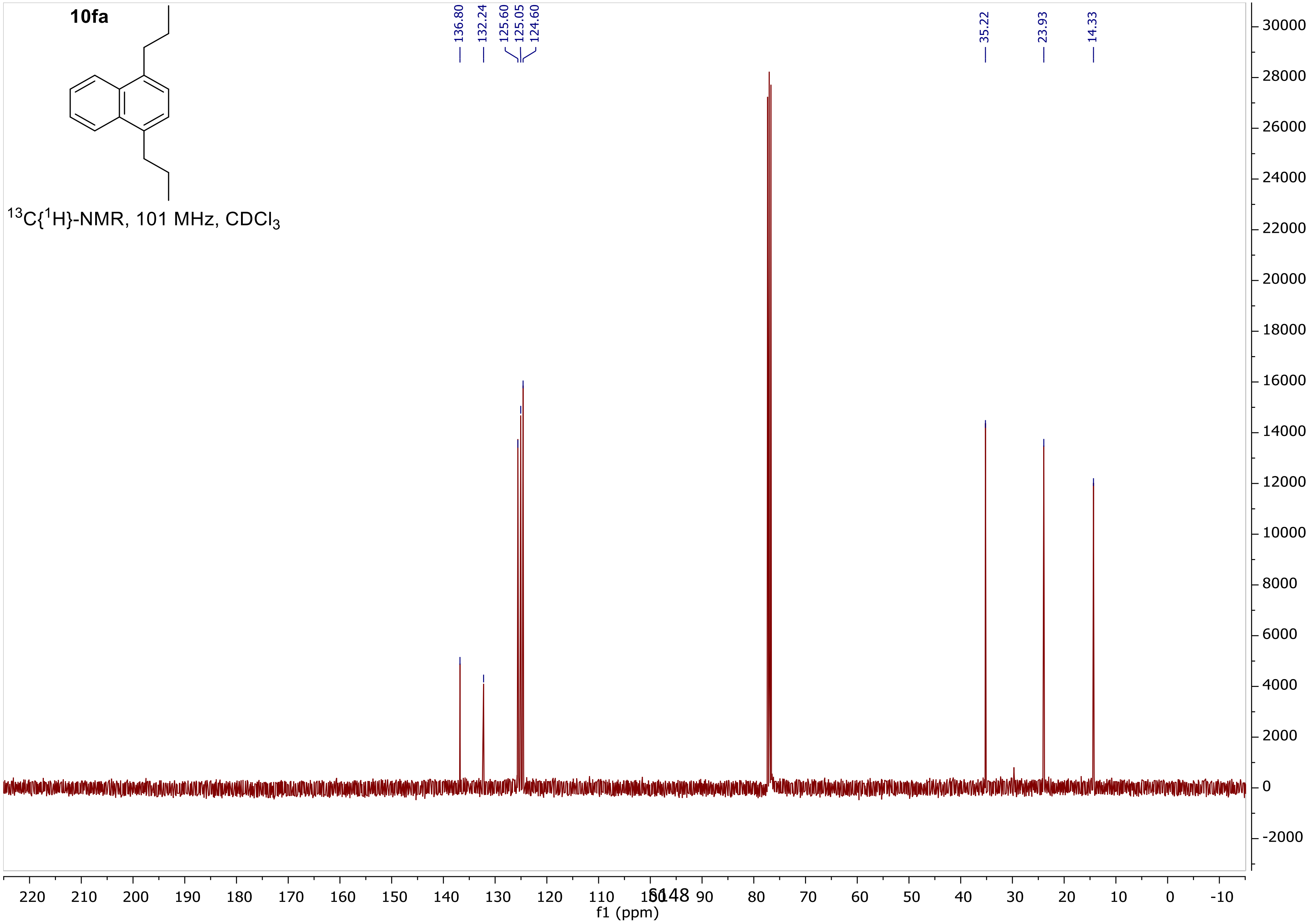
10fa
$^{13}C\{^{1}H\}$-NMR, 101 MHz, $CDCl_3$
136.80
132.24
125.60
125.05
124.60
35.22
23.93
14.33
30000
28000
26000
24000
22000
20000
18000
16000
14000
12000
10000
8000
6000
4000
2000
0
-2000
220 210 200 190 180 170 160 150 140 130 120 110 100 90 80 70 60 50 40 30 20 10 0 -10
f1 (ppm)

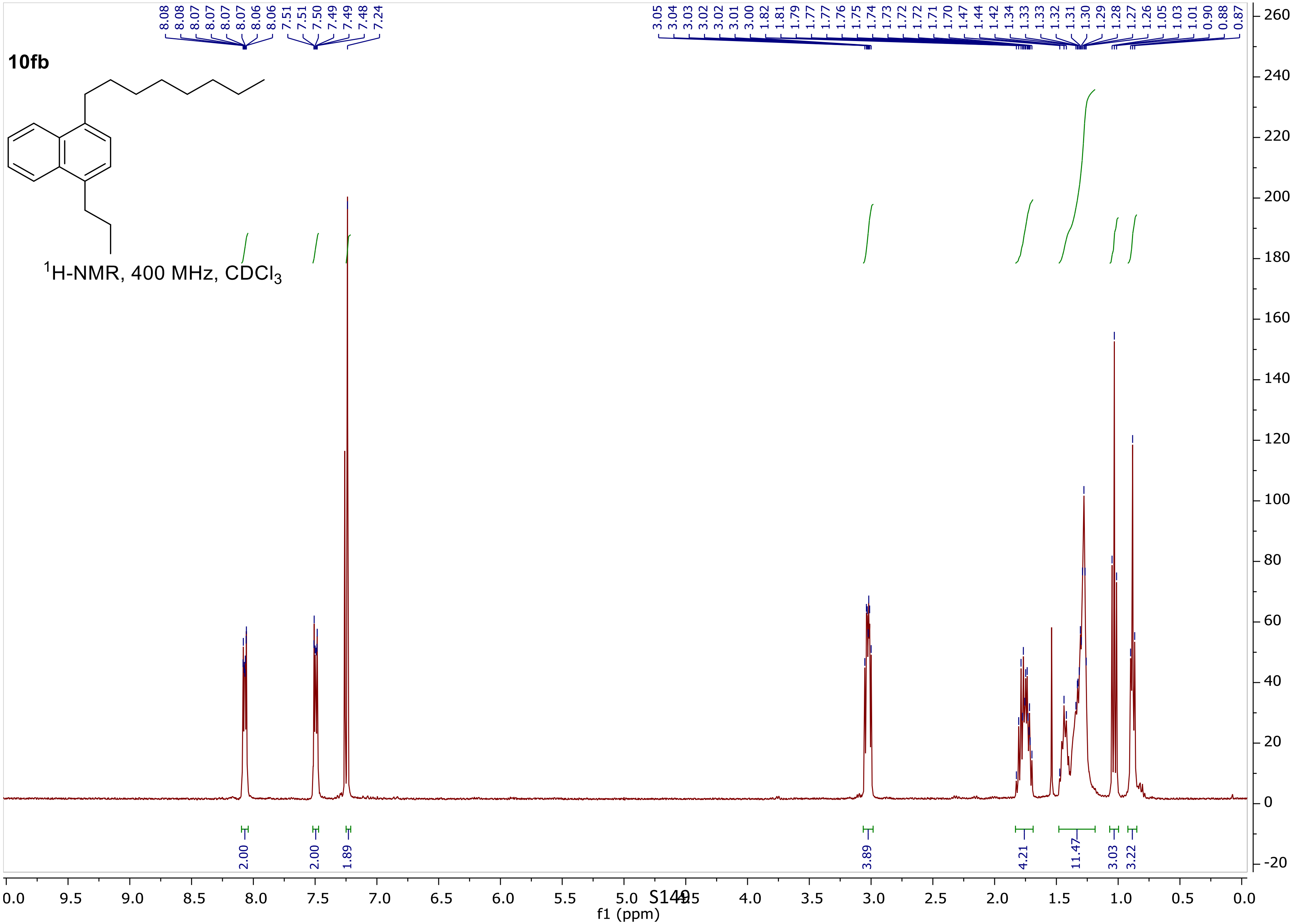

10fb
¹H-NMR, 400 MHz, CDCl₃
8.08 8.08 8.07 8.07 8.07 8.07 8.06 7.51 7.51 7.50 7.49 7.49 7.48 7.24
3.05 3.04 3.03 3.02 3.02 3.01 3.00 1.82 1.81 1.79 1.77 1.77 1.76 1.75 1.74 1.73 1.72 1.72 1.71 1.70 1.47 1.44 1.42 1.34 1.33 1.32 1.31 1.30 1.29 1.28 1.27 1.26 1.05 1.03 1.01 0.90 0.88 0.87
2.00 2.00 1.89 3.89 4.21 11.47 3.03 3.22
f1 (ppm)

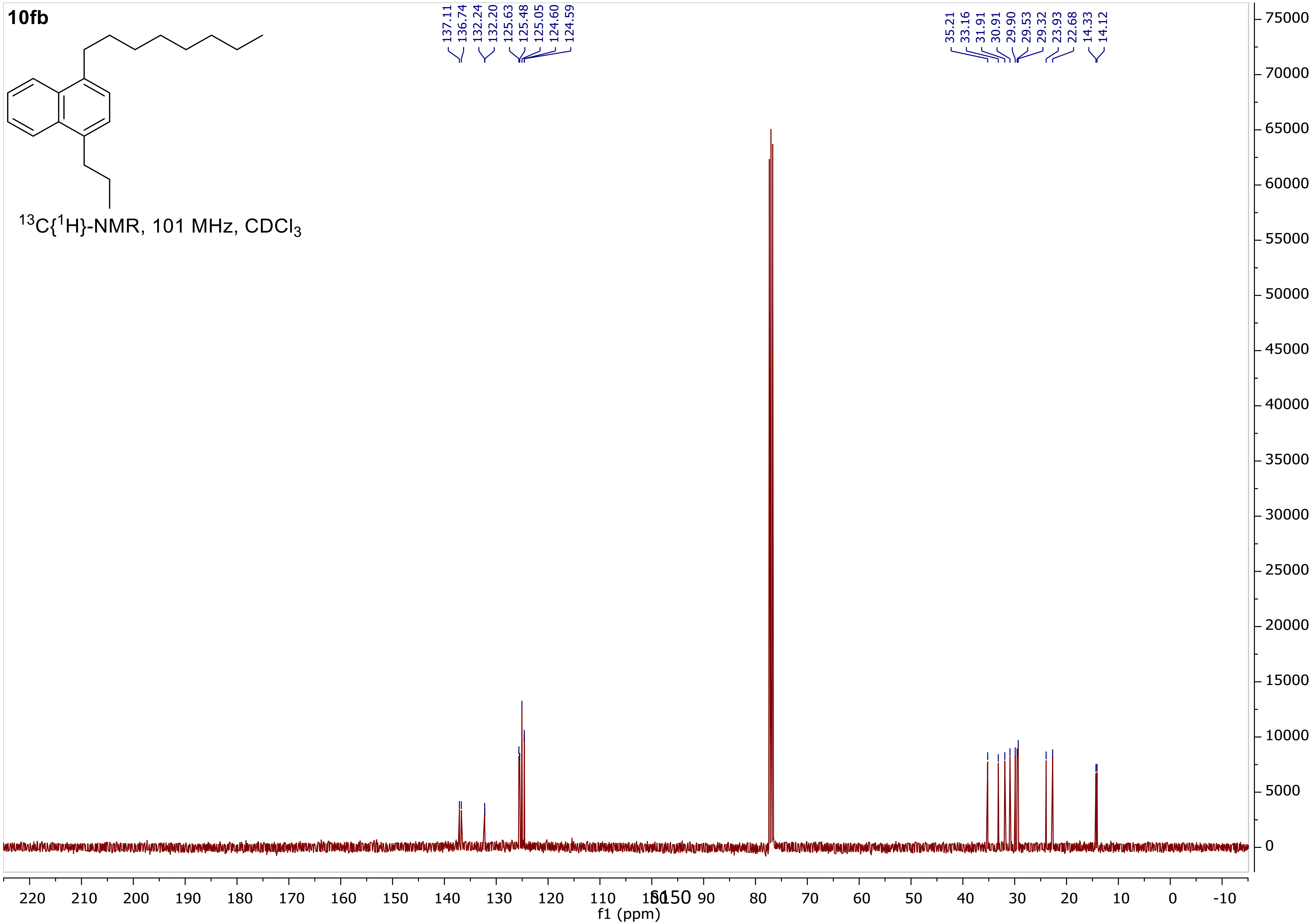
10fb
13C{1H}-NMR, 101 MHz, CDCl3
137.11
136.74
132.24
132.20
125.63
125.48
125.05
124.60
124.59
35.21
33.16
31.91
30.91
29.90
29.53
29.32
23.93
22.68
14.33
14.12
f1 (ppm)

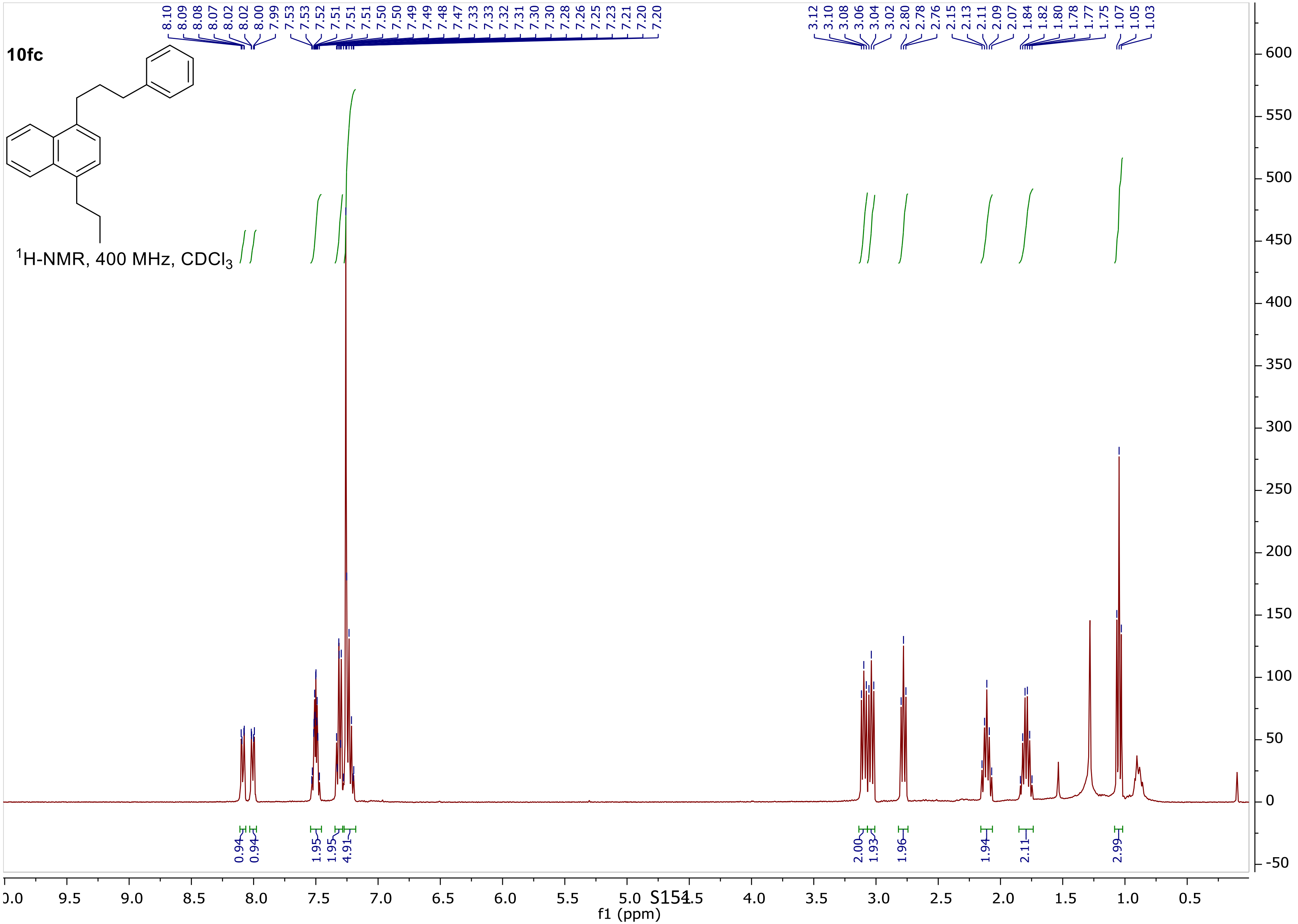
10fc
1H-NMR, 400 MHz, CDCl3
8.10 8.09 8.08 8.07 8.02 8.02 8.00 7.99 7.53 7.53 7.52 7.51 7.51 7.51 7.50 7.50 7.49 7.48 7.47 7.33 7.33 7.32 7.31 7.30 7.30 7.28 7.26 7.25 7.23 7.21 7.20 7.20
3.12 3.10 3.08 3.06 3.04 3.02 2.80 2.78 2.76 2.15 2.13 2.11 2.09 2.07 1.84 1.82 1.80 1.78 1.77 1.75 1.07 1.05 1.03
0.94 0.94 1.95 1.95 4.91 2.00 1.93 1.96 1.94 2.11 2.99
f1 (ppm)

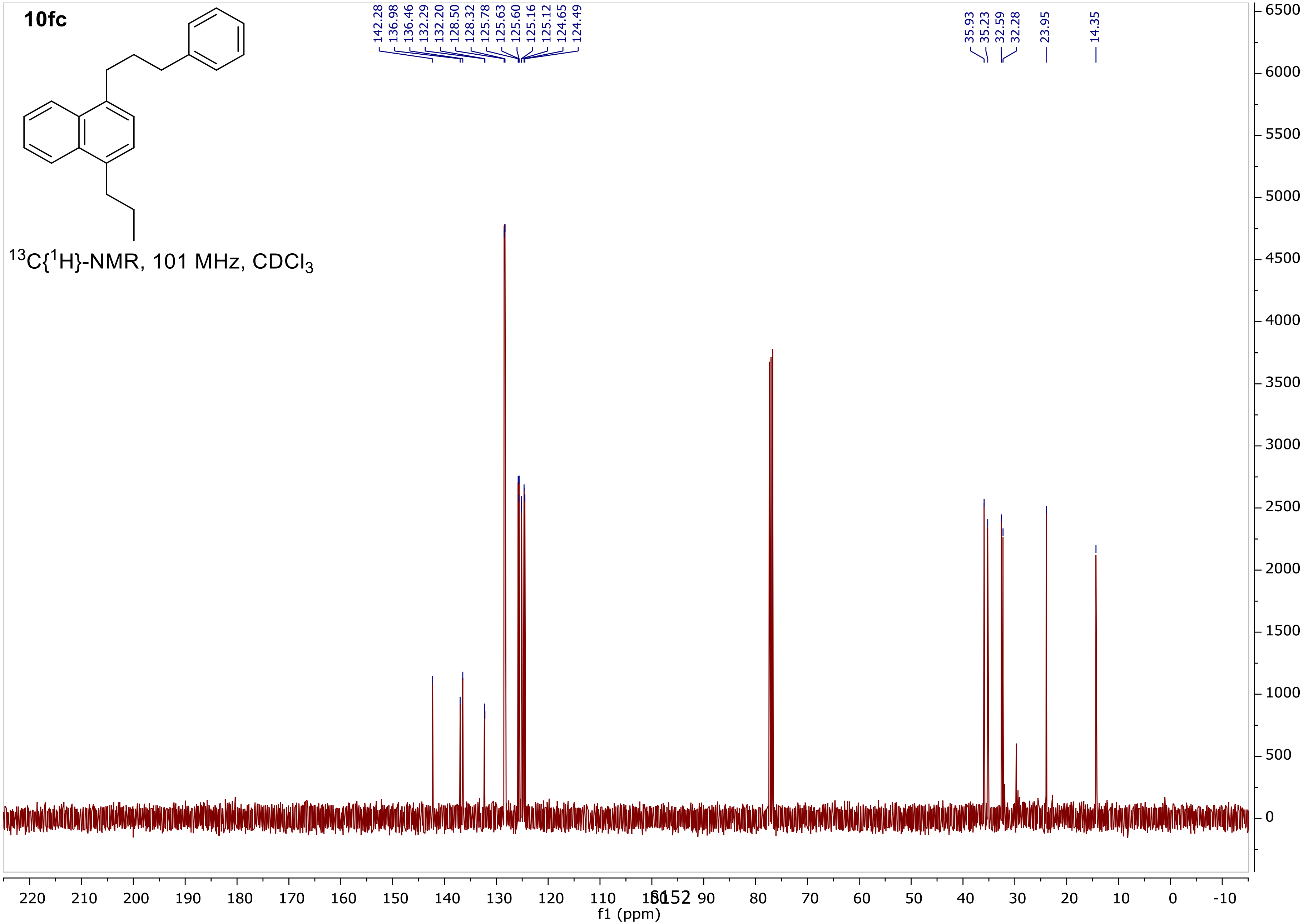
10fc
13C{1H}-NMR, 101 MHz, CDCl3
142.28
136.98
136.46
132.29
132.20
128.50
128.32
125.78
125.63
125.60
125.16
125.12
124.65
124.49
35.93
35.23
32.59
32.28
23.95
14.35
f1 (ppm)

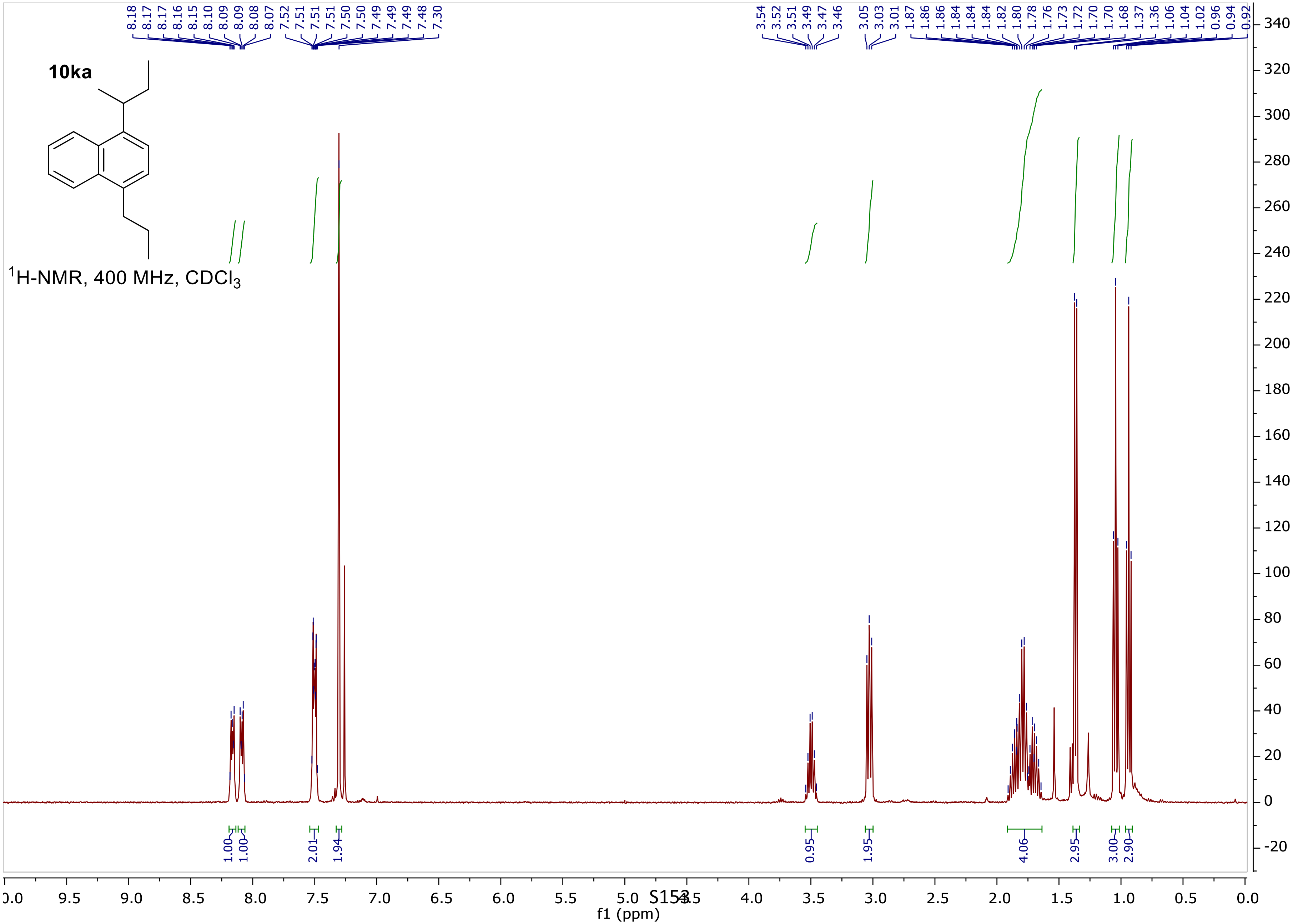
10ka
1H-NMR, 400 MHz, CDCl3
f1 (ppm)

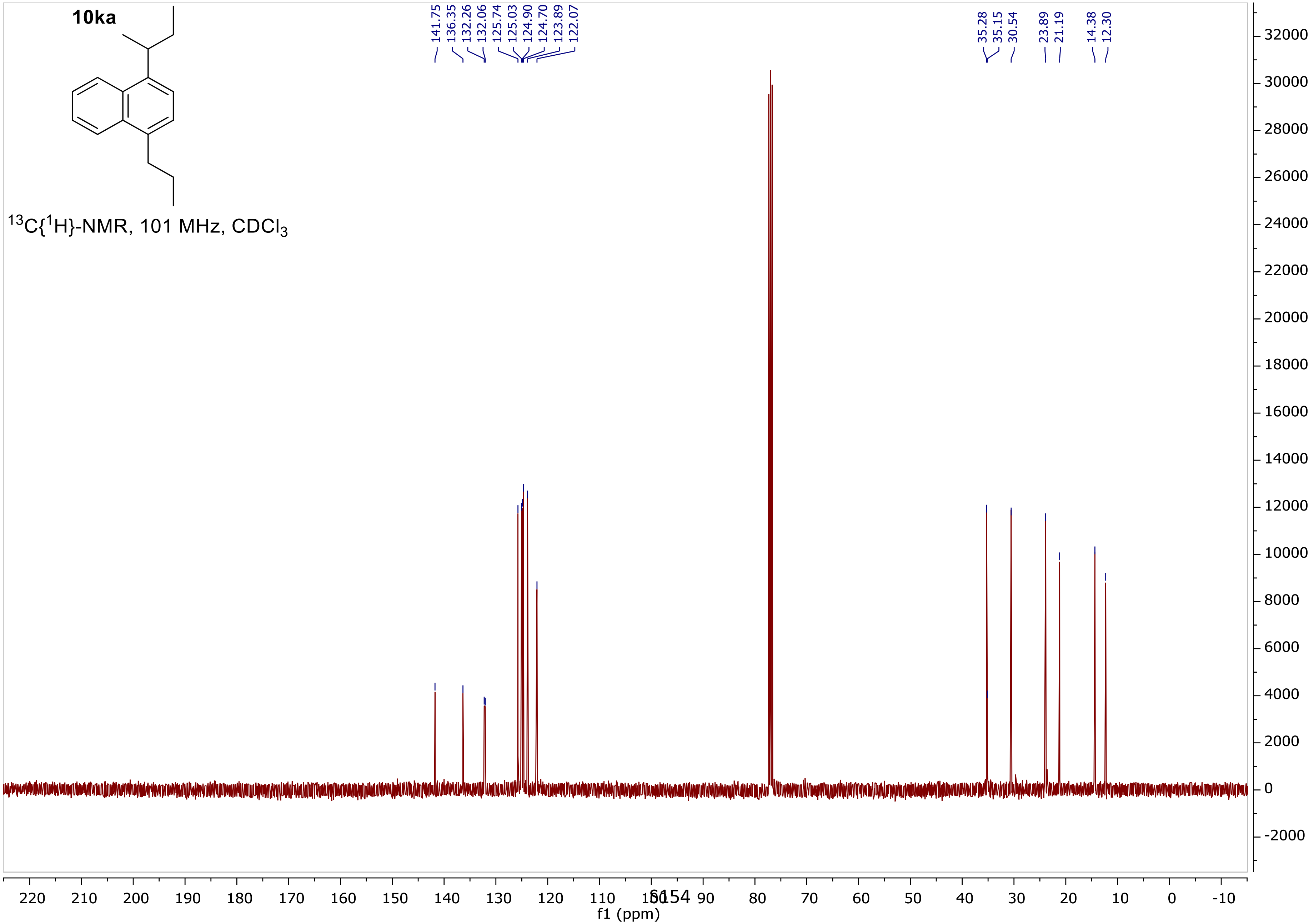
10ka
$^{13}C\{^{1}H\}$-NMR, 101 MHz, $CDCl_3$
141.75
136.35
132.26
132.06
125.74
125.03
124.90
124.70
123.89
122.07
35.28
35.15
30.54
23.89
21.19
14.38
12.30
f1 (ppm)
220 210 200 190 180 170 160 150 140 130 120 110 100 90 80 70 60 50 40 30 20 10 0 -10
32000 30000 28000 26000 24000 22000 20000 18000 16000 14000 12000 10000 8000 6000 4000 2000 0 -2000

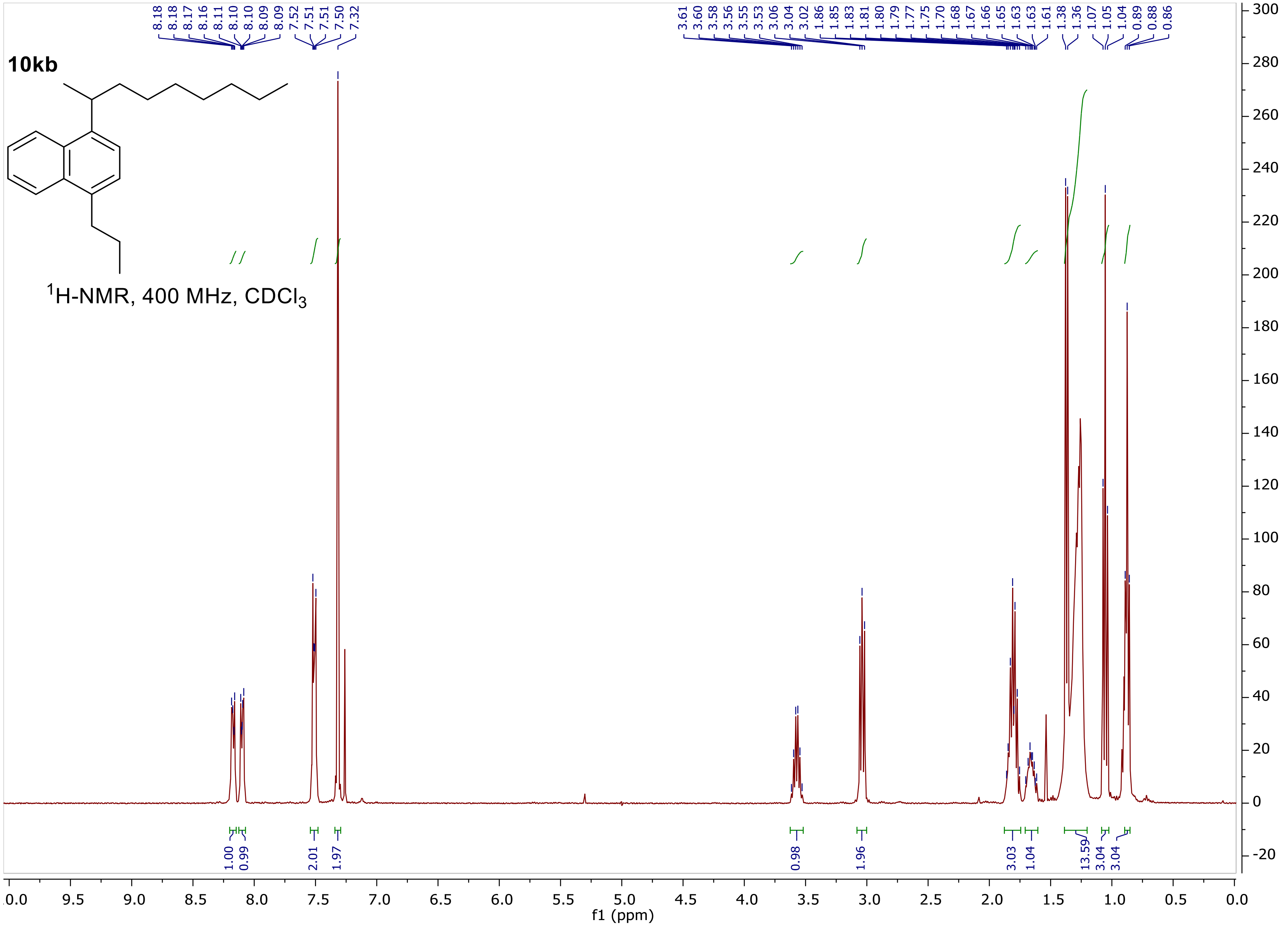
10kb
1H-NMR, 400 MHz, CDCl3
8.18 8.18 8.17 8.16 8.11 8.10 8.10 8.09 8.09 7.52 7.51 7.51 7.50 7.32
3.61 3.60 3.58 3.56 3.55 3.53 3.06 3.04 3.02 1.86 1.85 1.83 1.81 1.80 1.79 1.77 1.75 1.70 1.68 1.67 1.66 1.65 1.63 1.63 1.61 1.38 1.36 1.07 1.05 1.04 0.89 0.88 0.86
1.00 0.99 2.01 1.97 0.98 1.96 3.03 1.04 13.59 3.04 3.04
f1 (ppm)

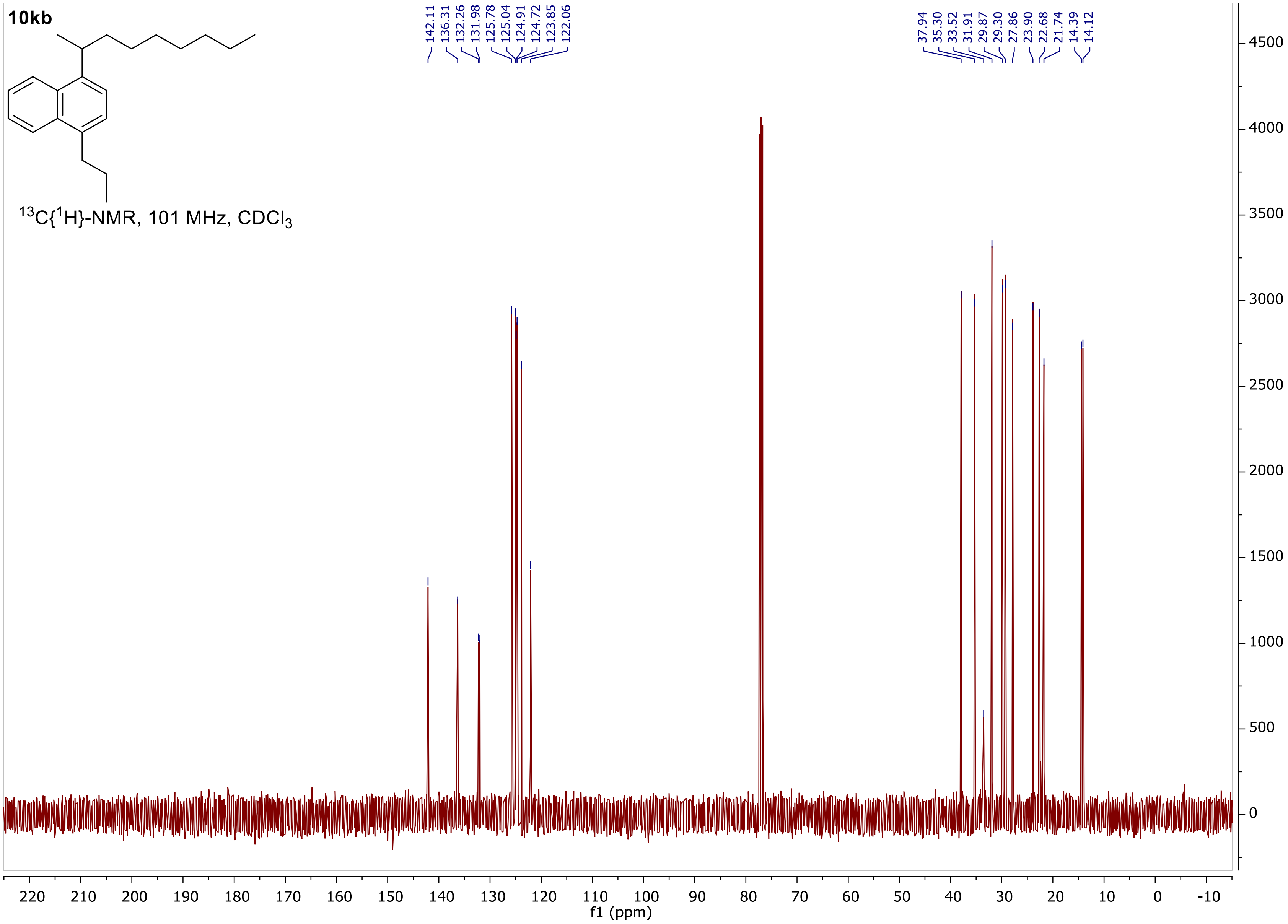
10kb
$^{13}C\{^1H\}$-NMR, 101 MHz, $CDCl_3$
142.11
136.31
132.26
131.98
125.78
125.04
124.91
124.72
123.85
122.06
37.94
35.30
33.52
31.91
29.87
29.30
27.86
23.90
22.68
21.74
14.39
14.12
f1 (ppm)

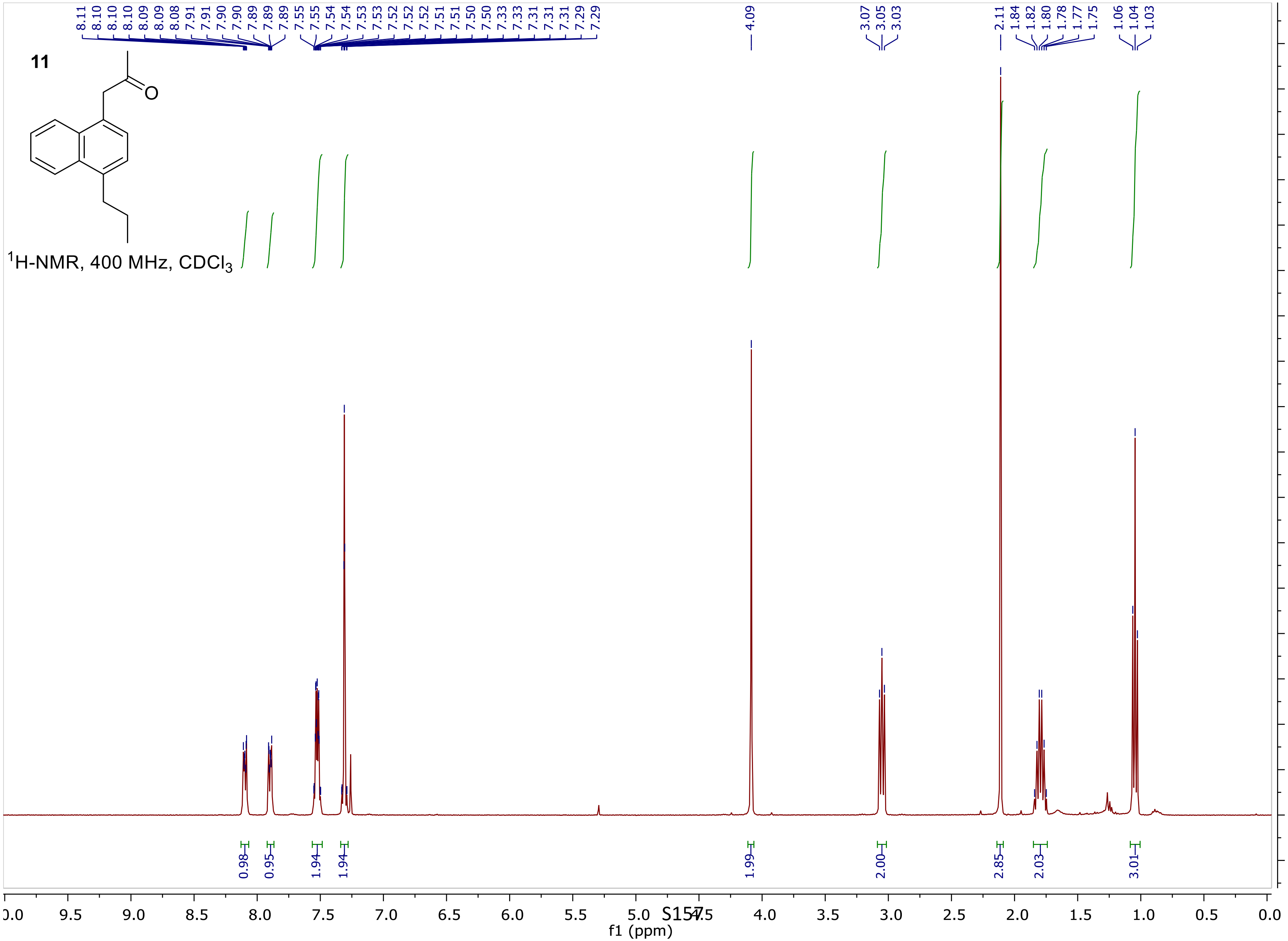
11
O
1H-NMR, 400 MHz, CDCl3
8.11 8.10 8.10 8.10 8.09 8.09 8.08 7.91 7.91 7.90 7.89 7.89 7.89 7.55 7.55 7.54 7.54 7.53 7.52 7.52 7.52 7.51 7.51 7.50 7.50 7.33 7.33 7.31 7.31 7.31 7.29 7.29
4.09
3.07 3.05 3.03
2.11 1.84 1.82 1.80 1.78 1.77 1.75 1.06 1.04 1.03
0.98 0.95 1.94 1.94 1.99 2.00 2.85 2.03 3.01
f1 (ppm)

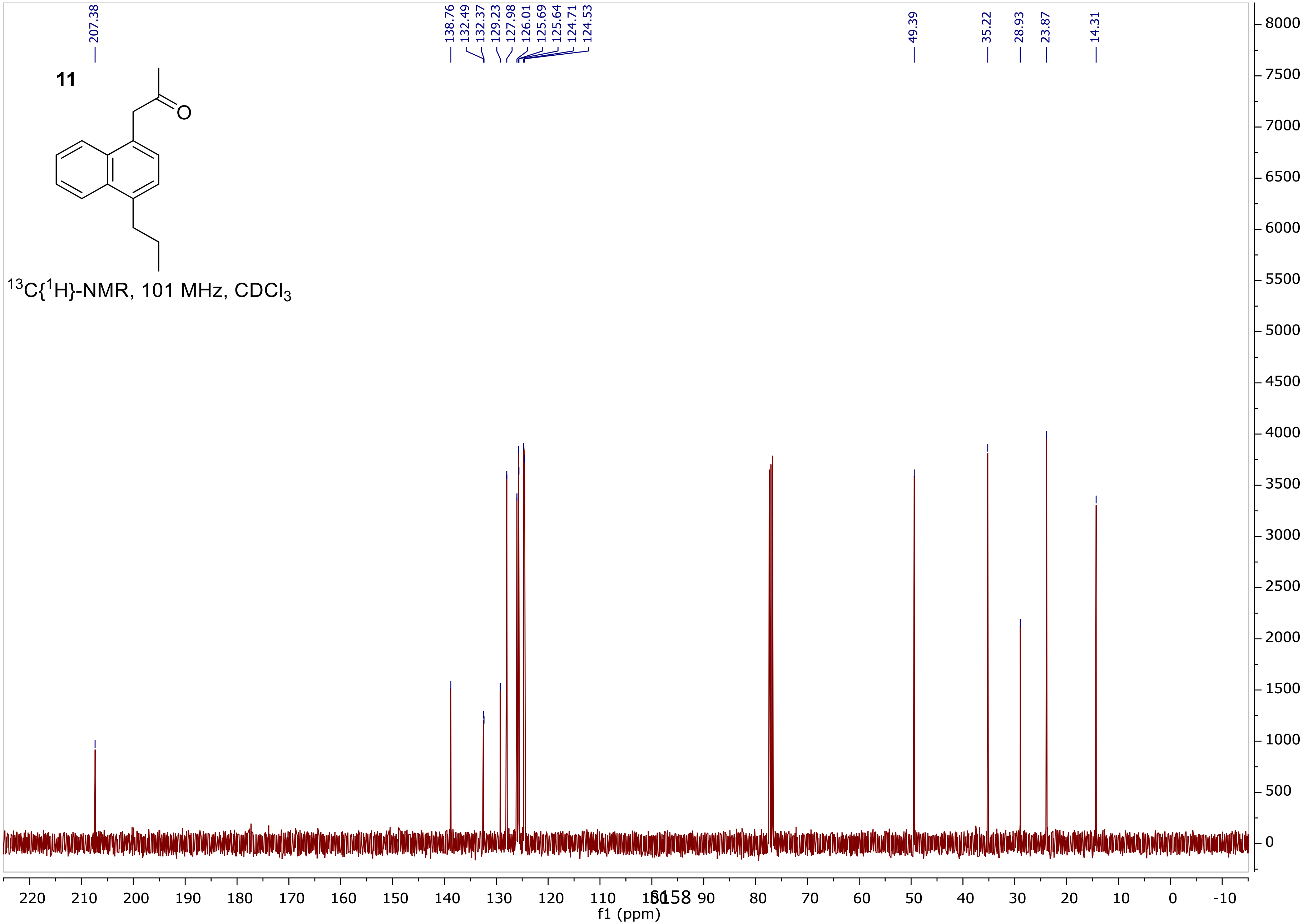
11
13C{1H}-NMR, 101 MHz, CDCl3
207.38
138.76
132.49
132.37
129.23
127.98
126.01
125.69
125.64
124.71
124.53
49.39
35.22
28.93
23.87
14.31
f1 (ppm)

11. Cartesian coordinates

I-Me

C	3.101390	-1.448530	-0.460097
C	2.799197	-0.102235	-0.371396
C	1.477730	0.331277	-0.063309
C	0.449726	-0.642623	0.151188
C	0.786471	-1.998449	0.059597
C	2.088195	-2.399433	-0.241228
H	4.118443	-1.771066	-0.695862
H	3.588986	0.631135	-0.537994
C	-0.970733	-0.230389	0.469334
H	0.026981	-2.765327	0.225050
H	2.319867	-3.466329	-0.306087
C	-1.142346	1.236701	0.568473
C	-0.142063	2.145062	0.375596
H	-2.152640	1.594364	0.792107
H	-0.351023	3.213749	0.460744
C	1.174671	1.727350	0.046556
C	2.216664	2.774478	-0.176965
H	3.044903	2.655153	0.545641
H	2.659205	2.675915	-1.184829
C	-1.929244	-0.729757	-0.705288
H	-1.740906	-1.810532	-0.818016
H	-1.604308	-0.241094	-1.639123

C	-3.396548	-0.504361	-0.467141
C	-4.144976	0.380867	-1.135650
H	-5.217685	0.487436	-0.935858
H	-3.867884	-1.133440	0.298580
H	-3.715452	1.025962	-1.913800
C	-1.421086	-0.851260	1.831328
H	-2.418962	-0.482806	2.116796
H	-1.467631	-1.949047	1.750501
H	-0.707919	-0.584425	2.627741
H	1.803741	3.788171	-0.071574

TS-(I-IIa-Me)

C	-1.539842	2.736891	0.176055
C	-2.087059	1.504687	-0.157375
C	-1.309410	0.324875	-0.113614
C	0.062121	0.429641	0.285718
C	0.596759	1.692208	0.616693
C	-0.192353	2.833293	0.564302
H	-2.162399	3.634940	0.135889
H	-3.135483	1.455536	-0.454322
C	0.887720	-0.774608	0.330498
H	1.640425	1.782889	0.918553
H	0.236215	3.804272	0.826216
C	0.266010	-2.014139	0.025582
C	-1.105029	-2.092305	-0.393721

H	0.903992	-2.887444	-0.135811
H	-1.507468	-3.072590	-0.656712
C	-1.880586	-0.967103	-0.478785
C	-3.305969	-1.061299	-0.948275
H	-4.004269	-0.711058	-0.167130
H	-3.471791	-0.430507	-1.839548
C	2.405838	-0.696966	0.324160
H	2.806196	-1.696485	0.570241
H	2.772210	-0.002860	1.096119
C	2.897320	-0.282202	-1.044496
C	3.633987	0.805954	-1.287113
H	3.974390	1.045692	-2.301071
H	2.614374	-0.947457	-1.871205
H	3.931427	1.493431	-0.484611
C	0.342572	-1.756746	1.913362
H	0.439165	-2.841446	2.063416
H	1.151477	-1.251193	2.459641
H	-0.650007	-1.370326	2.167826
H	-3.570925	-2.098473	-1.205421

IIa-Me

C	1.467635	2.845823	-0.305672
C	2.078503	1.640998	0.020607
C	1.343365	0.440160	0.066404
C	-0.076625	0.497314	-0.243800

C	-0.669559	1.765288	-0.564258
C	0.085258	2.914130	-0.598947
H	2.068800	3.759326	-0.332770
H	3.145105	1.636954	0.245726
C	-0.845308	-0.674045	-0.214884
H	-1.734065	1.820909	-0.789068
H	-0.374996	3.872073	-0.851970
C	-0.185785	-1.983895	-0.002800
C	1.235194	-1.946235	0.418889
H	-0.781491	-2.545151	0.746930
H	1.692621	-2.909978	0.663484
C	1.981449	-0.813109	0.441670
C	3.436445	-0.838751	0.835146
H	4.083328	-0.496077	0.007735
H	3.630521	-0.179988	1.700252
C	-2.345487	-0.675923	-0.354849
H	-2.689781	-1.705962	-0.546218
H	-2.662905	-0.062770	-1.213630
C	-3.009173	-0.178316	0.919063
C	-3.874081	0.838512	0.972235
H	-4.341478	1.131582	1.919452
H	-2.749833	-0.728037	1.832917
H	-4.157723	1.407919	0.077455
C	-0.284424	-2.833791	-1.330114
H	0.153365	-3.826463	-1.141351

H	-1.331723	-2.965305	-1.641372
H	0.278057	-2.338488	-2.137120
H	3.747762	-1.859492	1.107406

IIa-Me

C	1.467635	2.845823	-0.305672
C	2.078503	1.640998	0.020607
C	1.343365	0.440160	0.066404
C	-0.076625	0.497314	-0.243800
C	-0.669559	1.765288	-0.564258
C	0.085258	2.914130	-0.598947
H	2.068800	3.759326	-0.332770
H	3.145105	1.636954	0.245726
C	-0.845308	-0.674045	-0.214884
H	-1.734065	1.820909	-0.789068
H	-0.374996	3.872073	-0.851970
C	-0.185785	-1.983895	-0.002800
C	1.235194	-1.946235	0.418889
H	-0.781491	-2.545151	0.746930
H	1.692621	-2.909978	0.663484
C	1.981449	-0.813109	0.441670
C	3.436445	-0.838751	0.835146
H	4.083328	-0.496077	0.007735
H	3.630521	-0.179988	1.700252
C	-2.345487	-0.675923	-0.354849

H	-2.689781	-1.705962	-0.546218
H	-2.662905	-0.062770	-1.213630
C	-3.009173	-0.178316	0.919063
C	-3.874081	0.838512	0.972235
H	-4.341478	1.131582	1.919452
H	-2.749833	-0.728037	1.832917
H	-4.157723	1.407919	0.077455
C	-0.284424	-2.833791	-1.330114
H	0.153365	-3.826463	-1.141351
H	-1.331723	-2.965305	-1.641372
H	0.278057	-2.338488	-2.137120
H	3.747762	-1.859492	1.107406

TS-(I-IIb-Me)

C	2.820328	-1.720349	-0.592452
C	2.649703	-0.345630	-0.515707
C	1.454691	0.217655	-0.005718
C	0.410723	-0.670996	0.424594
C	0.617408	-2.071027	0.343542
C	1.801027	-2.589416	-0.157029
H	3.753176	-2.129836	-0.990194
H	3.456480	0.306611	-0.853294
C	-0.819769	-0.122870	0.942815
H	-0.163522	-2.755681	0.678282
H	1.942166	-3.672137	-0.213946
C	-0.958240	1.282444	0.978426

C	0.100520	2.151198	0.596426
H	-1.847643	1.706768	1.451900
H	-0.049749	3.229695	0.687488
C	1.286217	1.655396	0.103989
C	2.392640	2.588687	-0.307658
H	3.314782	2.393390	0.268643
H	2.645565	2.461718	-1.375855
C	-1.901509	0.759536	-1.044247
H	-0.919465	0.715918	-1.522921
H	-2.328670	1.759164	-0.921989
C	-2.809654	-0.335757	-1.208818
C	-4.141572	-0.208146	-0.991466
H	-4.825851	-1.048641	-1.152282
H	-2.397064	-1.299751	-1.526667
H	-4.578176	0.740453	-0.654645
C	-1.858990	-0.988817	1.590543
H	-2.767990	-0.415613	1.827689
H	-2.147625	-1.849383	0.965993
H	-1.451755	-1.393508	2.535883
H	2.104206	3.639379	-0.147516

IIb-Me

C	3.443653	-0.882821	-0.568426
C	2.837681	0.356357	-0.396705
C	1.489763	0.459080	-0.000083

C	0.747381	-0.769444	0.213633
C	1.414726	-2.027112	0.047256
C	2.733794	-2.084660	-0.342226
H	4.491856	-0.927188	-0.878109
H	3.425631	1.258008	-0.570490
C	-0.602717	-0.715627	0.601313
H	0.866321	-2.954940	0.214256
H	3.229750	-3.048487	-0.479030
C	-1.304530	0.581998	0.598912
C	-0.449280	1.790964	0.543490
H	-2.008884	0.630466	1.447974
H	-0.954834	2.750921	0.687369
C	0.870335	1.756832	0.231239
C	1.685420	3.019923	0.120397
H	2.548029	3.004072	0.810006
H	2.083254	3.152700	-0.901829
C	-2.264892	0.663503	-0.712614
H	-1.594906	0.628501	-1.586819
H	-2.710907	1.669138	-0.661496
C	-3.342098	-0.373493	-0.817508
C	-4.625440	-0.146432	-0.509989
H	-5.386336	-0.926713	-0.627851
H	-3.046388	-1.360695	-1.192928
H	-4.962954	0.829169	-0.135435
C	-1.366431	-1.932720	1.011678

H	-2.403790	-1.694522	1.282655
H	-1.374661	-2.699135	0.216876
H	-0.876464	-2.391174	1.891252
H	1.071096	3.901800	0.361404

I-Ph

C	2.947095	-2.148141	-1.302339
C	3.182890	-1.201811	-0.321566
C	2.149828	-0.317036	0.101662
C	0.857174	-0.410736	-0.503789
C	0.642013	-1.381560	-1.485960
C	1.669027	-2.238557	-1.883554
H	3.745448	-2.823324	-1.619562
H	4.173376	-1.140574	0.130861
C	-0.261547	0.542228	-0.123871
H	-0.345179	-1.483196	-1.941602
H	1.473393	-2.991135	-2.652656
C	0.094436	1.434116	1.009195
C	1.332874	1.490282	1.579301
H	-0.690420	2.108341	1.362461
H	1.530181	2.200879	2.384937
C	2.385049	0.642005	1.139879
C	3.720887	0.772072	1.794548
H	4.003695	-0.178995	2.282576
H	4.503776	0.987501	1.044941

C	-0.465646	1.532055	-1.363910
H	-0.742606	0.899309	-2.224070
H	0.518527	1.969637	-1.598867
C	-1.484693	2.620221	-1.160828
C	-1.202642	3.926791	-1.215027
H	-1.985664	4.683220	-1.086325
H	-2.516560	2.300804	-0.970717
H	-0.182282	4.289407	-1.397250
H	3.727234	1.569448	2.551568
C	-1.536619	-0.252164	0.245181
C	-1.598293	-0.851782	1.515466
C	-2.599852	-0.452898	-0.645986
C	-2.699945	-1.623921	1.891022
H	-0.769290	-0.724638	2.218607
C	-3.702902	-1.229748	-0.271317
H	-2.588361	-0.014861	-1.645455
C	-3.759243	-1.814681	0.996797
H	-2.728006	-2.079445	2.885243
H	-4.522614	-1.374303	-0.981100
H	-4.624006	-2.418497	1.286984

TS-(I-IIa-Ph)

C	-3.102898	-0.777026	1.826434
C	-2.677118	-1.459841	0.691997
C	-1.596460	-0.985281	-0.083061

C	-0.955799	0.226701	0.316234
C	-1.400349	0.901221	1.466917
C	-2.459194	0.406116	2.220507
H	-3.938379	-1.168368	2.413486
H	-3.185385	-2.383149	0.410677
C	0.149384	0.775942	-0.496807
H	-0.910410	1.823294	1.781855
H	-2.787139	0.940331	3.116236
C	0.620978	-0.035065	-1.587601
C	-0.041744	-1.274123	-1.947377
H	1.201936	0.448213	-2.377139
H	0.338742	-1.817397	-2.815096
C	-1.127686	-1.725646	-1.259852
C	-1.837092	-2.979507	-1.692322
H	-1.803766	-3.748043	-0.898820
H	-2.902172	-2.779924	-1.906433
C	0.325974	2.294680	-0.541095
H	1.166552	2.541985	-1.207433
H	0.576526	2.689457	0.455581
C	-0.916929	2.954714	-1.091213
C	-1.614967	3.904203	-0.461405
H	-2.493647	4.365699	-0.926802
H	-1.229806	2.617174	-2.088462
H	-1.333949	4.260201	0.538422
H	-1.376545	-3.403227	-2.598380

C	1.662953	-1.165312	0.669176
C	1.684505	-0.064910	-0.221507
C	2.883627	0.669237	-0.383145
C	4.020813	0.321117	0.341094
C	3.981396	-0.759068	1.230015
C	2.800349	-1.503216	1.387573
H	0.751987	-1.750952	0.798066
H	2.938873	1.501915	-1.085517
H	4.942340	0.892483	0.204540
H	4.874485	-1.028602	1.800819
H	2.771301	-2.350701	2.077022

IIa-Ph

C	-3.841573	-0.818302	1.175394
C	-3.025313	-1.598303	0.363027
C	-1.806514	-1.102301	-0.136099
C	-1.421825	0.252214	0.230772
C	-2.298050	1.025701	1.064183
C	-3.482485	0.503010	1.528526
H	-4.781837	-1.237068	1.545874
H	-3.347251	-2.609232	0.112668
C	-0.214427	0.785796	-0.236610
H	-2.022459	2.044418	1.334945
H	-4.140044	1.099143	2.165275
C	0.714754	-0.064606	-1.029390

C	0.211494	-1.402709	-1.433107
H	0.971441	0.508196	-1.943940
H	0.876278	-1.991808	-2.070887
C	-0.970192	-1.915555	-1.010374
C	-1.416600	-3.296014	-1.419051
H	-1.543008	-3.953976	-0.540701
H	-2.384547	-3.265073	-1.950177
C	0.201320	2.216184	-0.025441
H	1.292696	2.292431	-0.165269
H	-0.018052	2.551430	0.999739
C	-0.495217	3.113722	-1.035588
C	-1.294073	4.135390	-0.714548
H	-1.747817	4.761192	-1.491814
H	-0.297445	2.882681	-2.090314
H	-1.516016	4.394986	0.328707
H	-0.675522	-3.762093	-2.087211
C	2.027929	-0.897200	0.991121
C	2.037447	-0.211923	-0.232740
C	3.231748	0.334174	-0.718366
C	4.414459	0.188515	0.016376
C	4.405037	-0.493920	1.237362
C	3.209242	-1.036320	1.724137
H	1.095077	-1.326866	1.369598
H	3.244214	0.870778	-1.671832
H	5.346209	0.610899	-0.371052

H	5.330217	-0.604376	1.810510
H	3.194901	-1.570979	2.678368

TS-(I-IIb-Ph)

C	3.184615	-2.366743	-0.708394
C	3.395742	-1.090135	-0.212308
C	2.310428	-0.254898	0.156903
C	0.970828	-0.744235	-0.024249
C	0.789583	-2.065959	-0.494065
C	1.873777	-2.862782	-0.833939
H	4.037342	-2.992948	-0.984669
H	4.419307	-0.730767	-0.097372
C	-0.154896	0.123205	0.302523
H	-0.215814	-2.477676	-0.578052
H	1.705161	-3.881297	-1.194405
C	0.143743	1.384556	0.878394
C	1.458420	1.818790	1.119811
H	-0.688265	2.032256	1.163194
H	1.613398	2.799785	1.574686
C	2.544327	1.042055	0.754348
C	3.941905	1.542341	0.989833
H	4.503096	0.855660	1.648783
H	4.505479	1.611706	0.041924
C	-0.256624	1.514563	-1.517522
H	-0.164546	0.563717	-2.045248

H	0.669638	2.090144	-1.432558
C	-1.512532	2.206235	-1.584290
C	-1.605267	3.547586	-1.417049
H	-2.560915	4.068869	-1.541072
H	-2.406272	1.619335	-1.819223
H	-0.726394	4.156682	-1.170599
H	3.934015	2.538586	1.458558
C	-1.559940	-0.380674	0.376412
C	-2.263290	-0.240598	1.587424
C	-2.216441	-0.981886	-0.714970
C	-3.580920	-0.692528	1.706640
H	-1.768461	0.208852	2.452848
C	-3.535119	-1.426121	-0.597459
H	-1.707968	-1.090588	-1.676581
C	-4.221543	-1.285168	0.614494
H	-4.105952	-0.581106	2.659735
H	-4.029948	-1.880908	-1.460374
H	-5.253684	-1.635916	0.705191

IIb-Ph

C	3.523666	-2.168849	-0.386514
C	3.584651	-0.786700	-0.244580
C	2.430329	-0.028960	0.031372
C	1.156621	-0.722075	0.102301
C	1.150335	-2.154899	0.028589

C	2.305577	-2.863001	-0.221569
H	4.441014	-2.727603	-0.593627
H	4.552591	-0.292419	-0.331378
C	-0.036193	0.011865	0.291667
H	0.219965	-2.695921	0.199024
H	2.282889	-3.954043	-0.275258
C	0.025300	1.491713	0.422436
C	1.378152	2.079977	0.560403
H	-0.620282	1.818088	1.258225
H	1.419867	3.147738	0.797068
C	2.522237	1.398346	0.307609
C	3.866751	2.078997	0.348732
H	4.534632	1.610977	1.093712
H	4.374988	2.021099	-0.630595
C	-0.642636	2.200233	-0.870148
H	-0.089948	1.835767	-1.751387
H	-0.417127	3.271592	-0.751698
C	-2.121658	2.015330	-1.041809
C	-3.026946	2.934109	-0.680732
H	-4.098749	2.770817	-0.842731
H	-2.462334	1.085244	-1.508822
H	-2.730875	3.882763	-0.213100
H	3.756447	3.143589	0.608804
C	-1.362264	-0.607701	0.299075
C	-2.308304	-0.252266	1.288018

C	-1.754857	-1.502124	-0.726204
C	-3.588293	-0.803356	1.273346
H	-2.026880	0.426793	2.095979
C	-3.047703	-2.019793	-0.755005
H	-1.061191	-1.736632	-1.536724
C	-3.963892	-1.681601	0.249918
H	-4.299662	-0.539670	2.060546
H	-3.345600	-2.685382	-1.569723
H	-4.974981	-2.098268	0.230252

I_*p*OMePh

C	2.946678	-2.619083	-1.489614
C	3.284381	-1.991563	-0.304149
C	2.512507	-0.901070	0.189535
C	1.380593	-0.449942	-0.559302
C	1.053933	-1.107561	-1.748670
C	1.823297	-2.175768	-2.211496
H	3.544208	-3.455848	-1.859286
H	4.151867	-2.343813	0.255362
C	0.558989	0.742101	-0.104162
H	0.177042	-0.793043	-2.318811
H	1.544351	-2.675153	-3.143712
C	0.962848	1.249425	1.231148
C	2.034140	0.782147	1.936874
H	0.373201	2.077296	1.634002

H	2.290113	1.230296	2.899387
C	2.840137	-0.275050	1.436680
C	4.016061	-0.715539	2.245310
H	3.908829	-1.777879	2.532499
H	4.946547	-0.641183	1.653701
C	0.898315	1.942261	-1.107587
H	0.599840	1.598150	-2.112172
H	1.994511	2.055490	-1.126441
C	0.243738	3.257578	-0.784106
C	0.918580	4.377476	-0.500795
H	0.396864	5.319562	-0.295441
H	-0.852700	3.279120	-0.797686
H	2.016173	4.396756	-0.470359
H	4.129717	-0.115529	3.159762
C	-0.943297	0.387362	-0.069187
C	-1.433550	-0.350272	1.018598
C	-1.839671	0.708192	-1.102826
C	-2.769492	-0.752564	1.095157
H	-0.758535	-0.634930	1.832239
C	-3.172872	0.311783	-1.043027
H	-1.513037	1.271532	-1.978988
C	-3.656527	-0.420344	0.056791
H	-3.102059	-1.322924	1.963395
H	-3.867831	0.563169	-1.848007
O	-4.964020	-0.754487	0.024761

C	-5.524624	-1.490365	1.099264
H	-6.586681	-1.636002	0.851438
H	-5.038603	-2.478279	1.210595
H	-5.446636	-0.936115	2.053825

TS-(I-IIa-*p*OMePh)

C	-3.246690	-0.995788	2.266656
C	-3.022109	-1.611593	1.037521
C	-2.140675	-1.052707	0.089446
C	-1.489925	0.175372	0.413114
C	-1.729195	0.781933	1.658472
C	-2.594069	0.203653	2.583107
H	-3.931719	-1.454419	2.985370
H	-3.536110	-2.548314	0.817228
C	-0.601331	0.820504	-0.576842
H	-1.228888	1.717323	1.913039
H	-2.761604	0.686122	3.549850
C	-0.279730	0.064451	-1.774444
C	-0.981570	-1.192494	-2.058912
H	0.024773	0.634189	-2.658073
H	-0.766890	-1.678758	-3.013864
C	-1.884950	-1.722036	-1.197299
C	-2.619514	-2.992037	-1.537093
H	-2.399332	-3.789904	-0.804578
H	-3.713097	-2.835789	-1.527275

C	-0.525451	2.345394	-0.588479
H	0.151004	2.667335	-1.394980
H	-0.107007	2.728329	0.355529
C	-1.889104	2.939843	-0.854235
C	-2.495296	3.833902	-0.067601
H	-3.475373	4.249286	-0.329621
H	-2.381523	2.601338	-1.775707
H	-2.035032	4.188025	0.864157
H	-2.333738	-3.358569	-2.535707
C	1.205169	-1.081341	0.120576
C	0.975262	0.038185	-0.739584
C	2.086561	0.880849	-1.031447
C	3.336754	0.652741	-0.488666
C	3.530412	-0.446812	0.381681
C	2.441164	-1.315142	0.666952
H	0.385832	-1.762415	0.353548
H	1.969484	1.722904	-1.715849
H	4.159498	1.319520	-0.746727
H	2.617245	-2.165284	1.329227
O	4.677511	-0.747750	0.966328
C	5.841936	0.058898	0.766433
H	6.634383	-0.408161	1.367153
H	6.135624	0.064081	-0.297502
H	5.666485	1.090502	1.118013

IIa-*p*OMePh

C	-4.163305	-1.088484	1.619052
C	-3.478479	-1.743963	0.600451
C	-2.391443	-1.137190	-0.055210
C	-2.003166	0.199305	0.365563
C	-2.740065	0.841995	1.414380
C	-3.797145	0.212311	2.031497
H	-5.002324	-1.592040	2.108066
H	-3.800346	-2.744842	0.312189
C	-0.930642	0.844774	-0.268949
H	-2.457246	1.843670	1.736714
H	-4.346413	0.709705	2.834109
C	-0.102594	0.102954	-1.257761
C	-0.622106	-1.215815	-1.702375
H	0.037730	0.756363	-2.140512
H	-0.051219	-1.714269	-2.490847
C	-1.693653	-1.823385	-1.137831
C	-2.155162	-3.184162	-1.593115
H	-2.091995	-3.924435	-0.775211
H	-3.204960	-3.159729	-1.935470
C	-0.578094	2.289197	-0.048794
H	0.483636	2.441628	-0.304530
H	-0.708587	2.589943	1.000850
C	-1.441864	3.151341	-0.957071
C	-2.328685	4.051945	-0.523779

H	-2.906854	4.658287	-1.230710
H	-1.297441	2.997231	-2.034220
H	-2.503161	4.229178	0.545212
H	-1.533253	-3.546824	-2.426513
C	1.472906	-0.880733	0.507861
C	1.304323	-0.062457	-0.623299
C	2.414595	0.601458	-1.151977
C	3.683104	0.449485	-0.580519
C	3.847559	-0.372328	0.548130
C	2.725992	-1.035655	1.086618
H	0.615129	-1.408603	0.935669
H	2.303954	1.243301	-2.031041
H	4.529789	0.971878	-1.027470
H	2.868220	-1.672526	1.963093
O	5.021694	-0.587526	1.175427
C	6.199978	0.039316	0.694655
H	7.013927	-0.285734	1.359621
H	6.429659	-0.273238	-0.341530
H	6.115868	1.141800	0.733934

TS-(I-IIb-*p*OMePh)

C	-3.496583	-2.712024	0.628226
C	-3.847187	-1.507863	0.038987
C	-2.868684	-0.531291	-0.279226
C	-1.495583	-0.796743	0.051997

C	-1.164564	-2.051725	0.611915
C	-2.144729	-2.991583	0.900099
H	-4.268362	-3.449882	0.863815
H	-4.896829	-1.317988	-0.189625
C	-0.484180	0.222788	-0.216975
H	-0.122015	-2.299158	0.809579
H	-1.860540	-3.954177	1.334274
C	-0.915829	1.395595	-0.892221
C	-2.248569	1.605127	-1.283175
H	-0.169138	2.156716	-1.128478
H	-2.505473	2.528390	-1.807630
C	-3.235189	0.684367	-0.973113
C	-4.661190	0.949752	-1.366000
H	-5.044914	0.153971	-2.029403
H	-5.320746	0.976483	-0.479780
C	-0.778299	1.650364	1.512922
H	-0.776047	0.724272	2.091080
H	-1.769733	2.073256	1.327109
C	0.347793	2.533486	1.656417
C	0.256900	3.864620	1.427949
H	1.109481	4.529200	1.606461
H	1.292310	2.098619	1.999009
H	-0.675198	4.324385	1.075606
H	-4.754581	1.911673	-1.893336
C	0.977525	-0.059368	-0.138941

C	1.779469	0.155945	-1.272040
C	1.612971	-0.526401	1.032407
C	3.155469	-0.089755	-1.259226
H	1.321691	0.504031	-2.202283
C	2.980107	-0.764117	1.063946
H	1.038467	-0.685600	1.948710
C	3.770374	-0.553713	-0.084383
H	3.731433	0.081185	-2.169548
H	3.469781	-1.112636	1.976425
O	5.085252	-0.818796	0.042934
C	5.950087	-0.631660	-1.066365
H	6.957031	-0.908550	-0.720462
H	5.664365	-1.281842	-1.914519
H	5.956344	0.423214	-1.399663

IIb-*p*OMePh

C	-2.824053	-3.220202	0.758294
C	-3.274539	-2.168786	-0.041686
C	-2.463954	-1.051972	-0.302978
C	-1.121561	-1.050036	0.218895
C	-0.705340	-2.115789	1.064347
C	-1.545198	-3.184353	1.337430
H	-3.487373	-4.067531	0.954807
H	-4.284481	-2.216018	-0.451401
C	-0.247307	0.044783	-0.100289

H	0.268889	-2.060410	1.553001
H	-1.218078	-3.983346	2.007402
C	-0.904968	1.358305	-0.389554
C	-2.229495	1.225211	-1.080522
H	-0.245329	1.984904	-1.007986
H	-2.620265	2.137773	-1.539844
C	-2.965279	0.094627	-1.072276
C	-4.306828	0.015741	-1.754514
H	-4.346948	-0.830058	-2.464411
H	-5.123893	-0.131790	-1.024792
C	-1.127759	2.132931	0.985445
H	-0.167837	2.139074	1.530794
H	-1.850718	1.559568	1.588867
C	-1.595566	3.542664	0.766066
C	-2.792838	4.011785	1.134699
H	-3.074397	5.057192	0.961297
H	-0.881336	4.211098	0.264902
H	-3.532348	3.369255	1.630460
H	-4.516449	0.942327	-2.312500
C	1.176678	-0.077982	-0.136121
C	2.026352	1.063282	-0.011394
C	1.819587	-1.338164	-0.365938
C	3.403312	0.956322	-0.021536
H	1.599389	2.055358	0.140648
C	3.190883	-1.450042	-0.411921

H	1.216795	-2.221430	-0.581043
C	4.009863	-0.310348	-0.217009
H	4.009209	1.852059	0.116325
H	3.675374	-2.406391	-0.620162
O	5.318218	-0.516330	-0.258111
C	6.239520	0.565915	-0.105272
H	7.240342	0.117606	-0.173348
H	6.109096	1.307949	-0.912200
H	6.115555	1.050100	0.879066

BF_4

B	0.000000	0.000000	0.000000
F	0.811563	0.811563	0.811563
F	-0.811563	-0.811563	0.811563
F	0.811563	-0.811563	-0.811563
F	-0.811563	0.811563	-0.811563

H_2O

O	0.000000	0.000000	0.120029
H	0.000000	0.765480	-0.480116
H	-0.000000	-0.765480	-0.480116

IV

C	-1.565724	-3.464368	-0.388003
C	-0.944976	-2.307154	-0.823092
C	-1.594474	-1.045331	-0.720602
C	-2.932548	-0.994312	-0.208295
C	-3.529894	-2.174693	0.243849
C	-2.857228	-3.394813	0.163974
H	-1.050500	-4.424631	-0.468419
H	0.056521	-2.372803	-1.240656
H	-4.535549	-2.143857	0.671128
H	-3.344468	-4.304016	0.527442
C	-0.919061	0.174608	-1.042920
C	-3.691959	0.299145	-0.154104
C	-1.613631	1.415435	-0.963662
H	-1.078852	2.330402	-1.222736
C	-2.900425	1.487728	-0.519097
H	-3.385632	2.464682	-0.439022
C	0.535939	0.240527	-1.422813
H	0.671225	1.185110	-1.965839
H	0.791865	-0.570513	-2.116445
C	1.552002	0.275405	-0.191414
H	-4.072535	0.455126	0.875588
C	-4.999799	0.223992	-1.061397
H	-5.572722	-0.653584	-0.724941
H	-4.667773	0.031795	-2.096015

C	-5.859485	1.455584	-0.996072
C	-7.000928	1.533575	-0.302144
H	-7.601118	2.450929	-0.290275
H	-7.381682	0.679932	0.274727
H	-5.516590	2.330459	-1.564252
C	0.807286	1.064225	0.911879
C	0.871732	2.467321	0.943686
C	-0.110404	0.430656	1.771383
C	0.052857	3.211001	1.799604
H	1.558671	2.994071	0.277834
C	-0.929747	1.169776	2.628971
H	-0.216162	-0.655142	1.755691
C	-0.854118	2.566042	2.646773
H	0.123069	4.302841	1.798084
H	-1.635624	0.647052	3.281297
H	-1.495748	3.146974	3.315812
C	2.865936	0.979356	-0.615448
C	3.771798	1.367452	0.389559
C	3.247204	1.163687	-1.951853
C	5.003355	1.940084	0.071335
H	3.506549	1.218683	1.439594
C	4.486520	1.733638	-2.275760
H	2.596048	0.857450	-2.772828
C	5.367972	2.128595	-1.268195
H	5.683988	2.238189	0.874621

H	4.757384	1.865392	-3.327746
H	6.334006	2.575580	-1.521202
C	2.032146	-1.135739	0.232762
C	2.431571	-2.062913	-0.748775
C	2.257738	-1.480791	1.575861
C	2.966656	-3.306110	-0.406602
H	2.350696	-1.811626	-1.809730
C	2.798805	-2.723886	1.925327
H	2.028065	-0.772810	2.373180
C	3.142473	-3.650441	0.938462
H	3.256906	-4.003942	-1.197674
H	2.954422	-2.961426	2.982035
H	3.560000	-4.624006	1.211656

TS-(IV-V)

C	-2.480299	3.027605	0.006197
C	-1.687064	1.973703	0.433882
C	-2.073619	0.630980	0.219324
C	-3.339342	0.402161	-0.436514
C	-4.134922	1.504179	-0.864505
C	-3.710468	2.799274	-0.653586
H	-2.144221	4.053383	0.183236
H	-0.748508	2.196695	0.934953
H	-5.088721	1.302649	-1.360130

H	-4.319554	3.644201	-0.985135
C	-1.246054	-0.502392	0.597820
C	-3.787952	-0.917670	-0.629592
C	-1.734260	-1.773206	0.397614
H	-1.122118	-2.638105	0.663087
C	-3.035500	-2.005687	-0.138195
H	-3.322961	-3.022305	-0.419334
C	0.159401	-0.357149	1.147222
H	0.374179	-1.296846	1.674235
H	0.192899	0.436898	1.906640
C	1.337056	-0.163679	0.099946
H	-4.741186	-1.098515	-1.133975
C	-4.321364	-1.987546	1.623494
H	-3.747853	-2.846336	1.985719
H	-5.266635	-2.232665	1.130203
C	-4.196054	-0.742370	2.315220
C	-3.152375	-0.483665	3.143113
H	-3.053840	0.484023	3.646898
H	-2.385432	-1.239186	3.347870
H	-4.932623	0.040239	2.106615
C	1.010695	-1.112150	-1.072638
C	1.488647	-2.432672	-1.081088
C	0.080082	-0.752196	-2.065672
C	1.076956	-3.349795	-2.055515
H	2.189747	-2.761130	-0.311076

C	-0.328531	-1.659876	-3.045807
H	-0.359328	0.247178	-2.064147
C	0.171178	-2.966497	-3.048497
H	1.470475	-4.370806	-2.034034
H	-1.050721	-1.343775	-3.804833
H	-0.147217	-3.680412	-3.814252
C	2.695496	-0.532826	0.753096
C	3.836627	-0.585102	-0.070918
C	2.871793	-0.743400	2.127687
C	5.099847	-0.857047	0.452909
H	3.728324	-0.409069	-1.144687
C	4.141883	-1.010517	2.660646
H	2.028818	-0.695720	2.818769
C	5.260123	-1.072993	1.828534
H	5.965688	-0.896983	-0.215274
H	4.248194	-1.169377	3.738298
H	6.250034	-1.283287	2.244642
C	1.541816	1.322883	-0.299440
C	1.649104	2.291788	0.717674
C	1.782115	1.744986	-1.616211
C	1.908746	3.632358	0.429766
H	1.550811	1.995806	1.765703
C	2.046375	3.088930	-1.912602
H	1.781797	1.024397	-2.434733
C	2.095802	4.043839	-0.895467

H	1.976031	4.356866	1.247154
H	2.220043	3.382947	-2.952371
H	2.298252	5.093741	-1.127415

V

C	1.419866	3.858382	-0.655697
C	0.849736	2.622446	-0.937192
C	1.564523	1.426845	-0.735359
C	2.930707	1.549401	-0.239464
C	3.486751	2.844498	0.042637
C	2.742465	3.980721	-0.158362
H	0.826893	4.762190	-0.823958
H	-0.169533	2.594133	-1.312654
H	4.512617	2.901736	0.415975
H	3.157880	4.968418	0.054584
C	1.007027	0.115093	-1.005574
C	3.700807	0.417797	-0.056733
C	1.814157	-0.976882	-0.860988
H	1.412016	-1.973182	-1.065271
C	3.189119	-0.927386	-0.323031
H	3.081570	-1.364492	0.708126
C	-0.442787	-0.113782	-1.381447
H	-0.469005	-1.049593	-1.957434
H	-0.803146	0.673003	-2.057955

C	-1.457911	-0.308129	-0.175462
H	4.730270	0.509021	0.305401
C	4.215201	-1.866487	-1.051005
H	4.425349	-1.446295	-2.048419
H	3.702714	-2.833713	-1.196212
C	5.480248	-2.069072	-0.266306
C	6.690321	-1.646487	-0.649320
H	7.580811	-1.833213	-0.037721
H	6.836302	-1.107079	-1.594787
H	5.368862	-2.601436	0.689059
C	-0.765213	-1.283394	0.800715
C	-0.949426	-2.670799	0.671982
C	0.210769	-0.843129	1.715916
C	-0.207836	-3.579338	1.435999
H	-1.677178	-3.056388	-0.044888
C	0.944930	-1.744051	2.492606
H	0.427424	0.222679	1.810962
C	0.739927	-3.121491	2.356053
H	-0.376299	-4.652940	1.308303
H	1.687708	-1.364034	3.200832
H	1.315382	-3.830198	2.958974
C	-2.790898	-0.908490	-0.693331
C	-3.767501	-1.266593	0.256270
C	-3.108918	-1.060482	-2.049667
C	-5.007272	-1.772266	-0.132128

H	-3.547518	-1.145364	1.320640
C	-4.357966	-1.562669	-2.445497
H	-2.398932	-0.786380	-2.831346
C	-5.310590	-1.923735	-1.492256
H	-5.743124	-2.046401	0.630127
H	-4.578964	-1.668912	-3.512075
H	-6.283612	-2.316752	-1.802121
C	-1.893800	1.046764	0.444482
C	-2.378874	2.055650	-0.410976
C	-1.984368	1.279267	1.825961
C	-2.857556	3.269869	0.082528
H	-2.413803	1.884576	-1.490456
C	-2.467034	2.494973	2.329230
H	-1.697074	0.504051	2.536909
C	-2.890225	3.504160	1.462537
H	-3.218443	4.031530	-0.615389
H	-2.515842	2.643333	3.412393
H	-3.263586	4.454476	1.855657

TS-(IV-VII)

C	1.735550	3.280752	0.123894
C	1.021490	2.203825	-0.361712
C	1.625384	0.924568	-0.512873
C	3.013499	0.775045	-0.164756

C	3.716034	1.894284	0.336365
C	3.092909	3.123436	0.477602
H	1.248300	4.253194	0.233792
H	-0.025616	2.341357	-0.621015
H	4.766968	1.798571	0.612265
H	3.659604	3.977187	0.859912
C	0.887735	-0.202977	-1.009546
C	3.654015	-0.540720	-0.278363
C	1.539647	-1.429564	-1.175484
H	0.982967	-2.286221	-1.561227
C	2.889715	-1.579548	-0.880946
H	3.368856	-2.550449	-1.041280
C	-0.569353	-0.141410	-1.417964
H	-0.737535	-1.029155	-2.040216
H	-0.717548	0.725417	-2.076461
C	-1.679346	-0.177419	-0.279585
H	3.391119	-0.990214	0.906655
C	5.188256	-0.663180	-0.320776
H	5.458621	-1.725854	-0.197231
H	5.624716	-0.130479	0.541751
C	5.765251	-0.129962	-1.608561
C	6.449607	-0.861474	-2.494016
H	6.843239	-0.418651	-3.416693
H	6.647291	-1.929129	-2.328379
H	5.582965	0.934423	-1.809938

C	-1.048609	-0.946717	0.900586
C	-1.052402	-2.353220	0.911261
C	-0.348706	-0.290322	1.925886
C	-0.429348	-3.077324	1.931065
H	-1.557801	-2.897582	0.110293
C	0.245560	-1.007345	2.973607
H	-0.277286	0.798657	1.930673
C	0.204782	-2.407946	2.983856
H	-0.457905	-4.170645	1.912585
H	0.720274	-0.459188	3.794400
H	0.659162	-2.970124	3.805312
C	-2.952781	-0.891687	-0.813321
C	-3.890502	-1.411983	0.097958
C	-3.273993	-0.935674	-2.178950
C	-5.088900	-1.979520	-0.338862
H	-3.682785	-1.374247	1.169974
C	-4.477439	-1.500188	-2.621250
H	-2.600155	-0.516276	-2.928547
C	-5.388952	-2.029971	-1.705436
H	-5.793617	-2.382250	0.395135
H	-4.698245	-1.520383	-3.692874
H	-6.327794	-2.473263	-2.050631
C	-2.233584	1.223880	0.103028
C	-2.409051	2.222968	-0.871424
C	-2.746027	1.482294	1.387020

C	-3.008138	3.448328	-0.565014
H	-2.093176	2.055029	-1.903615
C	-3.348858	2.705042	1.699562
H	-2.689929	0.718967	2.164716
C	-3.472798	3.702703	0.728719
H	-3.117010	4.203441	-1.349277
H	-3.727536	2.871984	2.712549
H	-3.940065	4.661316	0.972784
O	3.243411	-1.317264	2.260839
H	3.602103	-2.204922	2.460687
H	2.276649	-1.346263	2.457767

VII

C	-0.041177	4.103749	0.100593
C	-0.466098	2.874233	-0.361934
C	0.449829	1.897730	-0.844148
C	1.854673	2.232672	-0.858247
C	2.256020	3.510717	-0.374736
C	1.335794	4.425217	0.097631
H	-0.773171	4.827602	0.471106
H	-1.529511	2.645395	-0.344220
H	3.315125	3.769284	-0.361923
H	1.672676	5.397781	0.468607
C	0.011271	0.608009	-1.308570

C	2.812773	1.278217	-1.354371
C	0.976250	-0.299371	-1.708552
H	0.678854	-1.298659	-2.036543
C	2.353016	0.027493	-1.738915
H	3.066811	-0.725307	-2.077506
C	-1.447683	0.210154	-1.482268
H	-1.427681	-0.654978	-2.158120
H	-1.962738	1.007011	-2.037754
C	-2.324795	-0.243600	-0.238906
H	2.585183	0.137934	0.349075
C	4.299960	1.594951	-1.463518
H	4.446056	2.621928	-1.837674
H	4.729092	0.915394	-2.220455
C	5.066661	1.397232	-0.174880
C	5.705348	2.355694	0.504406
H	6.251270	2.130698	1.428506
H	5.716737	3.398784	0.160434
H	5.096916	0.364426	0.192525
C	-1.337651	-0.862687	0.767036
C	-0.872230	-2.177166	0.573777
C	-0.776305	-0.122309	1.817516
C	0.068966	-2.753987	1.427686
H	-1.254438	-2.764601	-0.264371
C	0.140414	-0.705577	2.706549
H	-1.059113	0.921179	1.965907

C	0.560255	-2.031048	2.521610
H	0.419576	-3.772097	1.241573
H	0.516378	-0.119063	3.550915
H	1.277608	-2.486745	3.210383
C	-3.386287	-1.282513	-0.703039
C	-3.948040	-2.180805	0.222774
C	-3.903479	-1.282811	-2.008623
C	-4.965756	-3.063301	-0.146429
H	-3.584704	-2.195081	1.253023
C	-4.926699	-2.162905	-2.382343
H	-3.527312	-0.583793	-2.757998
C	-5.460471	-3.061931	-1.455771
H	-5.374996	-3.755109	0.596406
H	-5.306630	-2.139298	-3.408477
H	-6.257885	-3.751913	-1.747989
C	-3.224433	0.872344	0.363553
C	-3.789396	1.872775	-0.447265
C	-3.652198	0.817446	1.703223
C	-4.688451	2.812967	0.066400
H	-3.544830	1.929364	-1.509824
C	-4.551186	1.753366	2.223477
H	-3.290611	0.025214	2.361106
C	-5.067674	2.766941	1.410759
H	-5.096956	3.581973	-0.596373
H	-4.851512	1.682187	3.273461

H	-5.768516	3.502686	1.816491
O	2.680916	-0.129159	1.312330
H	3.448442	-0.858482	1.379370
H	1.811205	-0.499013	1.649431
B	4.239168	-2.567272	0.012616
F	4.855335	-1.847165	-0.999760
F	4.450443	-1.791785	1.271590
F	4.787171	-3.810736	0.193467
F	2.859071	-2.603250	-0.165036

VIII

C	1.440044	3.503740	-0.386356
C	0.808017	2.332271	-0.760733
C	1.474659	1.077053	-0.679202
C	2.838533	1.038224	-0.232739
C	3.451114	2.239343	0.140789
C	2.766835	3.454063	0.073112
H	0.910291	4.457290	-0.449748
H	-0.218356	2.380931	-1.115486
H	4.483969	2.241590	0.494209
H	3.274920	4.374165	0.375572
C	0.796058	-0.140452	-1.000974
C	3.616107	-0.258235	-0.176331
C	1.499740	-1.375198	-0.943633

H	0.968148	-2.293662	-1.197052
C	2.801697	-1.436625	-0.547387
H	3.291401	-2.413693	-0.493624
C	-0.654623	-0.211377	-1.401376
H	-0.770430	-1.151794	-1.956079
H	-0.898786	0.602161	-2.096674
C	-1.701048	-0.266827	-0.199961
C	4.200131	-0.507147	1.246455
C	4.817690	-0.152938	-1.232555
H	5.426902	0.711051	-0.923284
H	4.370359	0.088241	-2.212126
C	5.682524	-1.376443	-1.342392
C	6.912688	-1.473381	-0.823884
H	7.514624	-2.381594	-0.945562
H	7.365896	-0.644184	-0.264417
H	5.268337	-2.228195	-1.897846
C	-0.980511	-1.056439	0.918002
C	-1.034566	-2.460285	0.941388
C	-0.104362	-0.419364	1.816495
C	-0.252323	-3.201036	1.833271
H	-1.687449	-2.989816	0.244231
C	0.670418	-1.155599	2.717378
H	-0.001666	0.667001	1.805677
C	0.602604	-2.552129	2.730014
H	-0.313694	-4.293379	1.824360

H	1.332582	-0.629601	3.411465
H	1.210995	-3.129731	3.432103
C	-2.996040	-0.981304	-0.664752
C	-3.923490	-1.389153	0.312364
C	-3.340980	-1.155627	-2.012368
C	-5.140362	-1.971074	-0.043230
H	-3.687032	-1.248770	1.370340
C	-4.565480	-1.734990	-2.373885
H	-2.672548	-0.833927	-2.813360
C	-5.468415	-2.149440	-1.393547
H	-5.838196	-2.284411	0.739249
H	-4.807751	-1.858538	-3.433811
H	-6.422842	-2.603688	-1.675969
C	-2.211732	1.134837	0.222222
C	-2.591638	2.066207	-0.763212
C	-2.483942	1.464889	1.560440
C	-3.153283	3.299440	-0.427168
H	-2.474542	1.826145	-1.823388
C	-3.051792	2.697832	1.903456
H	-2.270126	0.753071	2.358728
C	-3.376147	3.629097	0.914542
H	-3.427302	4.000774	-1.220929
H	-3.243518	2.923616	2.956819
H	-3.814598	4.594750	1.182986
H	4.756169	-1.457648	1.265294

H	4.896443	0.300597	1.522341
H	3.389055	-0.550352	1.990513

TS-(VIII-IX)

C	2.105511	3.124825	0.103892
C	1.367912	2.062735	-0.389704
C	1.841296	0.730065	-0.305775
C	3.134606	0.508835	0.292958
C	3.866081	1.622005	0.794385
C	3.362916	2.905796	0.707028
H	1.706379	4.140070	0.025689
H	0.401844	2.264606	-0.845353
H	4.841245	1.462327	1.256984
H	3.938264	3.748668	1.099177
C	1.042479	-0.398850	-0.753416
C	3.665147	-0.817103	0.371452
C	1.596260	-1.654459	-0.694385
H	1.020395	-2.523146	-1.020003
C	2.911391	-1.867378	-0.198940
H	3.257484	-2.895333	-0.065301
C	-0.389931	-0.269168	-1.230157
H	-0.596571	-1.180797	-1.806775
H	-0.485470	0.569239	-1.934615
C	-1.518232	-0.188107	-0.112650

C	4.303222	-1.385394	-1.997062
H	4.445620	-2.466821	-2.065959
H	5.135458	-0.821549	-1.568115
C	3.409063	-0.734621	-2.889101
C	3.344992	0.620730	-2.979482
H	2.629013	1.111983	-3.647064
H	4.016911	1.265125	-2.400924
H	2.707352	-1.352421	-3.458154
C	-1.035887	-1.120832	1.017800
C	-1.351274	-2.488889	0.996533
C	-0.106271	-0.681937	1.978903
C	-0.777824	-3.381747	1.909435
H	-2.047310	-2.873481	0.247854
C	0.465743	-1.566965	2.896056
H	0.204620	0.364273	1.998361
C	0.131023	-2.924942	2.868266
H	-1.045147	-4.442149	1.865979
H	1.185318	-1.191002	3.629956
H	0.577981	-3.621031	3.584584
C	-2.878996	-0.658467	-0.691299
C	-3.943820	-0.889114	0.200866
C	-3.137866	-0.782326	-2.063820
C	-5.210507	-1.250916	-0.257109
H	-3.773802	-0.782641	1.275625
C	-4.411653	-1.139678	-2.529680

H	-2.358089	-0.593511	-2.803539
C	-5.452300	-1.380086	-1.631382
H	-6.015171	-1.429907	0.462782
H	-4.583143	-1.228011	-3.607029
H	-6.445249	-1.661107	-1.995235
C	-1.827945	1.266040	0.334126
C	-2.010177	2.265758	-0.640828
C	-2.099737	1.611971	1.667624
C	-2.376100	3.568215	-0.296080
H	-1.886665	2.026876	-1.700567
C	-2.470473	2.915825	2.020464
H	-2.038175	0.859076	2.454497
C	-2.596446	3.906350	1.044226
H	-2.499485	4.319662	-1.081963
H	-2.666015	3.150656	3.071347
H	-2.881932	4.925952	1.319908
C	4.949010	-1.120685	1.093078
H	5.221539	-2.182990	0.993489
H	5.793090	-0.510581	0.727933
H	4.832427	-0.896950	2.169079

IX

C	1.512959	3.609191	-0.802646
C	0.925908	2.389287	-1.109937

C	1.506535	1.171492	-0.707555
C	2.775008	1.231693	0.001520
C	3.336358	2.512285	0.323379
C	2.720788	3.677877	-0.070207
H	1.025469	4.532089	-1.129675
H	-0.003860	2.385545	-1.672180
H	4.283303	2.564517	0.862122
H	3.165169	4.647323	0.167298
C	0.864583	-0.109010	-0.960511
C	3.447584	0.048744	0.348782
C	1.589300	-1.234836	-0.732896
H	1.160277	-2.215360	-0.959057
C	2.969703	-1.237265	-0.200825
H	3.066820	-2.029009	0.564589
C	-0.584786	-0.250222	-1.371279
H	-0.674059	-1.213604	-1.893139
H	-0.871477	0.522607	-2.097732
C	-1.637690	-0.285029	-0.183628
C	3.946682	-1.697591	-1.402091
H	3.919213	-0.912516	-2.174369
H	3.457901	-2.597237	-1.813773
C	5.351026	-2.033440	-1.008169
C	6.434725	-1.405310	-1.480599
H	7.445271	-1.718981	-1.194183
H	6.350327	-0.561937	-2.178664

H	5.470405	-2.871692	-0.308220
C	-1.120816	-1.342041	0.817029
C	-1.568022	-2.672636	0.752096
C	-0.068401	-1.059270	1.711762
C	-1.009634	-3.671189	1.558875
H	-2.363007	-2.944274	0.055162
C	0.485233	-2.048885	2.529079
H	0.345515	-0.051063	1.766317
C	0.015992	-3.364323	2.457720
H	-1.384255	-4.696461	1.481280
H	1.294640	-1.787642	3.217753
H	0.448000	-4.142817	3.093720
C	-3.033609	-0.692679	-0.721710
C	-4.090906	-0.794905	0.203587
C	-3.320278	-0.931633	-2.072200
C	-5.380752	-1.134846	-0.201109
H	-3.893308	-0.604861	1.262312
C	-4.618838	-1.267312	-2.485575
H	-2.544984	-0.860255	-2.836159
C	-5.653144	-1.373217	-1.555484
H	-6.179703	-1.210811	0.542965
H	-4.813379	-1.446408	-3.547598
H	-6.664844	-1.636143	-1.878972
C	-1.864916	1.131139	0.411191
C	-2.239751	2.169986	-0.463729

C	-1.856208	1.421176	1.784236
C	-2.515020	3.456673	-0.001092
H	-2.345821	1.967478	-1.533160
C	-2.134138	2.711249	2.257288
H	-1.651714	0.637351	2.513716
C	-2.448010	3.740705	1.368619
H	-2.794808	4.238652	-0.713694
H	-2.110716	2.902075	3.334669
H	-2.661510	4.748312	1.737782
C	4.677584	0.038666	1.192316
H	4.921552	-0.973680	1.541946
H	5.543439	0.400635	0.604309
H	4.569861	0.701027	2.067205

TS-(IX-X)

C	2.385154	3.074478	-0.087294
C	1.557435	2.047723	-0.480371
C	1.916400	0.679017	-0.293191
C	3.223137	0.393324	0.289984
C	4.050238	1.496444	0.687791
C	3.643288	2.797306	0.512666
H	2.071497	4.111052	-0.239155
H	0.600734	2.289511	-0.935802
H	5.024097	1.296465	1.137231

H	4.290583	3.621025	0.825927
C	1.046520	-0.396530	-0.654141
C	3.644031	-0.957190	0.497969
C	1.530357	-1.688003	-0.510731
H	0.895286	-2.531976	-0.791413
C	2.810505	-1.968591	0.048931
H	3.104572	-3.013668	0.178530
C	-0.365281	-0.221662	-1.174437
H	-0.579415	-1.109811	-1.786377
H	-0.427478	0.640674	-1.853713
C	-1.529984	-0.137996	-0.100812
C	3.452863	-1.865489	-2.560317
H	2.761833	-2.644425	-2.896765
H	4.389284	-2.188834	-2.096058
C	3.229958	-0.525086	-2.852370
C	4.074765	0.440266	-2.344580
H	3.872722	1.507790	-2.479465
H	5.006599	0.162969	-1.842104
H	2.313430	-0.229492	-3.371387
C	-1.288286	-1.297940	0.889571
C	-1.941280	-2.532043	0.732272
C	-0.290112	-1.217237	1.880806
C	-1.633871	-3.629372	1.545896
H	-2.702392	-2.649342	-0.041282
C	0.014226	-2.305444	2.703000

H	0.281485	-0.296026	2.004223
C	-0.659724	-3.520319	2.542004
H	-2.164760	-4.574710	1.396490
H	0.792173	-2.202166	3.465603
H	-0.423124	-4.375085	3.182966
C	-2.908041	-0.297393	-0.794987
C	-4.060387	-0.279181	0.015510
C	-3.085846	-0.413924	-2.179912
C	-5.337360	-0.388560	-0.532578
H	-3.948233	-0.178563	1.098702
C	-4.369689	-0.516982	-2.737654
H	-2.232988	-0.422153	-2.859783
C	-5.499836	-0.508120	-1.919969
H	-6.212048	-0.376281	0.125105
H	-4.476752	-0.604388	-3.823408
H	-6.500297	-0.589720	-2.355589
C	-1.608346	1.271656	0.545723
C	-1.742760	2.387525	-0.304714
C	-1.690211	1.504251	1.926656
C	-1.877591	3.681849	0.197097
H	-1.766414	2.243311	-1.388544
C	-1.828308	2.802236	2.438701
H	-1.666598	0.671418	2.629675
C	-1.906557	3.899797	1.580296
H	-1.971156	4.523426	-0.496235

H	-1.881176	2.946336	3.522317
H	-2.010618	4.912754	1.980653
C	4.973541	-1.282027	1.128963
H	5.817405	-0.869883	0.544860
H	5.051230	-0.859210	2.146508
H	5.116837	-2.371689	1.203618

X

C	-2.475299	-3.260722	-1.319856
C	-1.617531	-2.188697	-1.394052
C	-1.990455	-0.894430	-0.896410
C	-3.311904	-0.731266	-0.311245
C	-4.162970	-1.852642	-0.261820
C	-3.755724	-3.088608	-0.748057
H	-2.169377	-4.238145	-1.699811
H	-0.635560	-2.337043	-1.838852
H	-5.157501	-1.762351	0.175200
H	-4.438536	-3.940958	-0.686039
C	-1.087976	0.184746	-0.906052
C	-3.730961	0.541758	0.250473
C	-1.532185	1.520623	-0.438232
H	-0.778827	1.838552	0.318565
C	-2.874357	1.591543	0.178241
H	-3.177436	2.572026	0.556198

C	0.347423	0.027035	-1.287153
H	0.672363	0.908765	-1.853869
H	0.476593	-0.835908	-1.947531
C	1.364975	-0.182416	-0.070286
C	-1.415513	2.632700	-1.572558
H	-2.193608	2.437778	-2.328143
H	-0.434983	2.516695	-2.060533
C	-1.524726	4.021071	-1.012767
C	-2.494645	4.889998	-1.318394
H	-2.506790	5.901815	-0.896523
H	-3.306783	4.624573	-2.008006
H	-0.729543	4.311859	-0.311943
C	1.497782	1.108467	0.773347
C	1.724569	2.336861	0.119008
C	1.529850	1.108813	2.177874
C	1.913201	3.521478	0.832726
H	1.778441	2.376694	-0.971355
C	1.725078	2.293780	2.898365
H	1.420791	0.175093	2.730696
C	1.902808	3.508331	2.232351
H	2.078838	4.456722	0.289902
H	1.742009	2.258223	3.991722
H	2.048983	4.434254	2.796080
C	2.786508	-0.429218	-0.636803
C	3.824771	-0.657691	0.287103

C	3.110379	-0.415440	-1.999687
C	5.135251	-0.875576	-0.135521
H	3.598164	-0.668724	1.356600
C	4.429222	-0.628470	-2.428464
H	2.352346	-0.237242	-2.763721
C	5.445911	-0.861066	-1.502091
H	5.919699	-1.054687	0.606027
H	4.653181	-0.610495	-3.499371
H	6.473771	-1.027488	-1.837944
C	0.854541	-1.417039	0.700572
C	1.351950	-2.699473	0.416587
C	-0.233837	-1.322114	1.588338
C	0.796865	-3.840020	1.008505
H	2.180826	-2.818555	-0.283707
C	-0.788540	-2.456152	2.186000
H	-0.677545	-0.348703	1.811281
C	-0.273350	-3.724533	1.899335
H	1.206706	-4.824999	0.765613
H	-1.635141	-2.344876	2.869818
H	-0.707329	-4.615416	2.362793
C	-5.089405	0.685583	0.888348
H	-5.898291	0.464275	0.169253
H	-5.208541	-0.006620	1.740768
H	-5.237163	1.711592	1.260486

TS-(IX-XI)

C	1.144034	3.777334	-0.669881
C	0.650078	2.494741	-0.831440
C	1.471640	1.355991	-0.650098
C	2.859194	1.568275	-0.304812
C	3.332817	2.906442	-0.146568
C	2.496604	3.988944	-0.321362
H	0.478111	4.632584	-0.816460
H	-0.396407	2.367358	-1.097605
H	4.376945	3.085456	0.111255
H	2.879422	5.005085	-0.195381
C	0.956952	0.015400	-0.813951
C	3.751061	0.463436	-0.169479
C	1.827031	-1.026027	-0.645332
H	1.467653	-2.051371	-0.767689
C	3.222473	-0.868467	-0.261775
H	3.125136	-0.969561	0.996655
C	-0.468869	-0.307268	-1.227850
H	-0.435147	-1.347052	-1.578357
H	-0.737241	0.297970	-2.105970
C	-1.629736	-0.251846	-0.149621
C	4.123316	-2.081625	-0.585224
H	3.568156	-2.997233	-0.323059
H	5.021234	-2.072873	0.053250

C	4.514565	-2.104010	-2.040613
C	4.134463	-3.040328	-2.916107
H	4.445347	-2.999928	-3.966837
H	3.501840	-3.885582	-2.613700
H	5.142210	-1.267741	-2.379604
C	-0.995713	-0.688549	1.186276
C	-0.967487	-2.041313	1.564588
C	-0.321644	0.232552	2.008842
C	-0.356136	-2.451887	2.754019
H	-1.443022	-2.791840	0.930050
C	0.275904	-0.165085	3.208056
H	-0.269310	1.283933	1.719835
C	0.246111	-1.511675	3.599429
H	-0.366837	-3.510455	3.029534
H	0.765744	0.581401	3.840581
H	0.683798	-1.824080	4.552868
C	-2.785928	-1.204871	-0.564603
C	-3.789680	-1.502203	0.377509
C	-2.941413	-1.709103	-1.864224
C	-4.889421	-2.292857	0.044221
H	-3.708125	-1.105368	1.392795
C	-4.048968	-2.498435	-2.206003
H	-2.211843	-1.489884	-2.645378
C	-5.025029	-2.799331	-1.254987
H	-5.647632	-2.511212	0.802580

H	-4.141730	-2.876598	-3.228809
H	-5.887623	-3.417404	-1.521674
C	-2.352969	1.123209	-0.086163
C	-2.696254	1.785791	-1.280159
C	-2.832302	1.673351	1.114356
C	-3.422760	2.978585	-1.273577
H	-2.409970	1.362428	-2.246007
C	-3.564398	2.866816	1.127658
H	-2.649155	1.168500	2.063980
C	-3.852647	3.535624	-0.064125
H	-3.661169	3.468676	-2.222440
H	-3.913820	3.269539	2.083371
H	-4.420713	4.470595	-0.054002
C	5.202514	0.690514	0.141877
H	5.747266	-0.242954	0.330317
H	5.690775	1.197777	-0.710097
H	5.328120	1.342887	1.022390
O	3.099141	-1.420066	2.385065
H	3.519091	-0.779545	2.992413
H	2.161354	-1.505740	2.677389

XI

C	1.055505	4.318558	-0.269389
C	1.056887	3.046753	0.268819

C	-0.148707	2.322634	0.479055
C	-1.396595	2.959424	0.148382
C	-1.359796	4.269690	-0.411516
C	-0.166788	4.933926	-0.622048
H	2.001442	4.847238	-0.420003
H	2.007553	2.588809	0.533483
H	-2.291845	4.770878	-0.676411
H	-0.170493	5.939804	-1.052552
C	-0.156670	0.986828	1.008544
C	-2.650889	2.294570	0.403906
C	-1.378731	0.387698	1.234649
H	-1.405949	-0.628128	1.634101
C	-2.631053	1.015455	0.964154
H	-1.879600	-0.122012	-2.212157
C	1.089828	0.209393	1.400761
H	0.719507	-0.656768	1.964033
H	1.670695	0.807526	2.118480
C	2.056136	-0.382855	0.288798
C	-3.892425	0.261693	1.373105
H	-3.663357	-0.809438	1.457033
H	-4.667092	0.344037	0.589205
C	-4.441003	0.768040	2.686204
C	-4.548783	0.029561	3.796139
H	-4.947826	0.449697	4.727419
H	-4.244977	-1.025703	3.812297

H	-4.747805	1.823139	2.709007
C	1.177464	-0.680634	-0.942482
C	0.491381	-1.901414	-1.055554
C	0.944792	0.292648	-1.930630
C	-0.333961	-2.176964	-2.148487
H	0.595355	-2.656259	-0.273874
C	0.136043	0.022215	-3.039533
H	1.409664	1.276012	-1.848485
C	-0.496647	-1.225594	-3.163937
H	-0.874049	-3.124977	-2.194808
H	-0.002665	0.789405	-3.807091
H	-1.112770	-1.451415	-4.040090
C	2.743333	-1.673868	0.821149
C	3.345498	-2.564661	-0.087759
C	2.895607	-1.940837	2.190715
C	4.043693	-3.691030	0.350240
H	3.266676	-2.375444	-1.161010
C	3.600725	-3.067230	2.635776
H	2.478717	-1.268738	2.942557
C	4.173479	-3.951881	1.719948
H	4.491372	-4.367668	-0.384378
H	3.699141	-3.247408	3.710843
H	4.720713	-4.833511	2.067279
C	3.288132	0.513757	-0.026782
C	3.944217	1.217489	0.999896

C	3.891867	0.523639	-1.296771
C	5.111018	1.948212	0.758855
H	3.554499	1.196079	2.019966
C	5.060758	1.251323	-1.544827
H	3.455559	-0.049880	-2.116153
C	5.672920	1.979674	-0.521319
H	5.584738	2.490702	1.582813
H	5.494595	1.241685	-2.549586
H	6.584688	2.552864	-0.714501
C	-3.943299	3.005230	0.070793
H	-4.829306	2.386897	0.268026
H	-4.049454	3.930532	0.665126
H	-3.976969	3.298711	-0.993601
O	-2.749055	0.232893	-1.857356
H	-2.587645	0.676368	-0.965664
H	-3.439036	-0.563421	-1.732186
B	-3.648822	-2.689432	-0.700144
F	-4.295493	-1.610416	-1.498053
F	-2.516015	-2.097711	-0.139835
F	-3.308921	-3.702298	-1.573156
F	-4.547474	-3.084492	0.262969

12. Reference

[1] Berlin, D. K.; Shupe, R. D. Mass spectrometry of five classes of trityl compounds-loss of ^{12}C from $(C_6H_5)_3{}^{13}CH$. *Org. Mass Spectrom.* **1969**, 447-466.
[2] Kohn, W.; Sham, L. J. Self-consistent equations including exchange and correlation effects. *Phys. Rev. A*. **1965**, 140, 1133-1138.
[3] (a) Becke, A. D. Density-functional Thermochemistry. III. The role of Exact Exchange. *J. Chem. Phys*. **1993**, *98*, 5648-5652. (b) Becke, A. D. A New Mixing of Hartree-Fock and Local DensityFunctional Theories. *J. Chem. Phys.* **1993**, *98*, 1372-1377. (c) Lee, C.; Yang, W.; Parr, R. G. Development of the Colle-Salvetti Correlation-Energy Formula into a Functional of the Electron Density. *Phys. Rev. B: Condens. Matter Mater. Phys.* **1988**, *37*, 785-789.
[4] Weigend, F.; Ahlrichs, R. Balanced Basis Sets of Split Valence, Triple Zeta Valence and Quadruple Zeta Valence Quality for H to Rn: Design and Assessment of Accuracy. *Phys. Chem. Chem. Phys.* **2005**, 7, 3297-3305.
[5] Tomasi, J.; Mennucci, B.; Cammi, R. Quantum mechanical continuum solvation models. *Chem. Rev.* **2005**, *105*, 2999-3093.
[6] Gaussian 09, Revision A02, Frisch, M. J.; Trucks, G. W.; Schlegel, H. B.; Scuseria, G. E.; Robb, M. A.; Cheeseman, J. R.; Scalmani, G.; Barone, V.; Petersson, G. A.; Nakatsuji, H.; Li, X.; Caricato, M.; Marenich, A. V.; Bloino, J.; Janesko, B. G.; Gomperts, R.; Mennucci, B.; Hratchian, H. P.; Ortiz, J. V.; Izmaylov, A. F.; Sonnenberg, J. L.; Williams-Young, D.; Ding, F.; Lipparini, F.; Egidi, F.; Goings, J.; Peng, B.; Petrone, A.; Henderson, T.; Ranasinghe, D.; Zakrzewski, V. G.; Gao, J.; Rega, N.; Zheng, G.; Liang, W.; Hada, M.; Ehara, M.; Toyota, K.; Fukuda, R.; Hasegawa, J.; Ishida, M.; Nakajima, T.; Honda, Y.; Kitao, O.; Nakai, H.; Vreven, T.; Throssell, K.; Montgomery, J. A., Jr.; Peralta, J. E.; Ogliaro, F.; Bearpark, M. J.; Heyd, J. J.; Brothers, E. N.; Kudin, K. N.; Staroverov, V. N.; Keith, T. A.; Kobayashi, R.; Normand, J.; Raghavachari, K.; Rendell, A. P.; Burant, J. C.; Iyengar, S. S.; Tomasi, J.; Cossi, M.; Millam, J. M.; Klene, M.; Adamo, C.; Cammi, R.; Ochterski, J. W.; Martin, R. L.; Morokuma, K.; Farkas, O.; Foresman, J. B.; Fox, D. J. Gaussian, Inc., Wallingford CT, 2009.
[7] (a) Schlegel, H. B.; McDouall, J. J. W. Do You Have SCF Stability and Convergence Problems? in Computational Advances in Organic Chemistry: Molecular Structure and Reactivity **1991**, 94, 167-185. (b) Bauernschmitt, R.; Ahlrichs, R. Stability analysis for solutions of the closed shell Kohn-Sham equation. *J. Chem. Phys.* **1996**, *104*, 9047-9052.
[8] (a) González, C.; Schlegel, H. B. Reaction path following in mass-weighted internal coordinates. *J. Phys. Chem.* **1990**, *94*, 5523–5527. (b) Fukui, K. The path of chemical reactions – the IRC approach. *Acc. Chem. Res.* **1981**, *14*, 363–368. (c) Maeda, S.; Harabuchi, Y.; Ono, Y.; Taketsugu T.; Morokuma, K. Intrinsic reaction coordinate: Calculation, bifurcation, and automated search. *Int. J. Quantum Chem.* **2015**, 115, 258-269.
[9] Schaftenaar, G.; Noordik, J. Molden: a pre- and postprocessing program for molecular and electronic structures. *J. Comput.-Aided Mol. Des.* **2000**, *14*, 123-134.
[10] CYLview, 1.0b; Legault, C. Y., Université de Sherbrooke, 2009 (http://www.cylview.org).
[11] West, T. H.; Walden, D. M.; Taylor, J. E.; Brueckner, A. C.; Johnston, R. C.; Cheong, P. H.; Lloyd-Jones, G. C.; Smith, A. D. Catalytic Enantioselective [2,3]-Rearrangements of Allylic Ammonium Ylides: A Mechanistic and Computational Study. *J. Am. Chem. Soc.* **2017**, *139*, 4366–4375.
[12] Huang, X.; Ding, R.; Wang, P.; Xu, Y.; Loh, T. Palladium-catalyzed silylation reaction between benzylic halides and silylboronate. *Chem. Commun.* **2016**, *52*, 5609-5612.
[13] Fanfrlík, J.; Kolář, M.; Kamlar, M.; Hurný, D.; Ruiz, F. X.; Cousido-Siah, A.; Mitschler, A.; Řezáč, J.; Munusamy, E.; Lepšík, M.; Matějíček, P.; Veselý, J.; Podjarny, A.; Hobza, P. Modulation of Aldose Reductase Inhibition by Halogen Bond Tuning. *ACS Chem. Biol.* **2013**, *8*, 2484–2492.
[14] Pinto-Bazurco Mendieta, M. A. E.; Negri, M.; Hu, Q.; Hille, U. E.; Jagusch, C.; Jahn-Hoffmann, K.; Muller-Vieira, U.; Schmidt, D.; Lauterbach, T.; Hartmann, R. W. CYP17 Inhibitors. Annulations of Additional Rings in Methylene Imidazole Substituted Biphenyls: Synthesis, Biological Evaluation and Molecular Modelling. Arch. Pharm. Chem. Life Sci. 2008, 597-609.
[15] Chen, Y.; Qi, L.; Fang, F.; Tan, B. Organocatalytic Atroposelective Arylation of 2-Naphthylamines as a Practical Approach to Axially Chiral Biaryl Amino Alcohols. *Angew. Chem. Int. Ed.* **2017**, *56*, 16308-16312.
[16] Ryu, E.-H., Cho, H.; Zhao, Y. Catalyzing Methanolysis of Alkyl Halides in the Interior of an Amphiphilic Molecular Basket. *Org. Lett.* **2007**, *9*, 5147-5150.
[17] Peng, B.; Feng, X.; Zhang, X.; Zhang, S.; Bao, B. Propargylic and Allenic Carbocycle Synthesis through Palladium-Catalyzed Dearomatization Reaction. Pd-catalyzed allylative dearomatisation using Grignard reagents. *J. Org. Chem.*, **2010**, *75*, 2619-2627.
[18] Boldrini, C.; Harutyunyan, S. R. Pd-catalyzed allylative dearomatisation using Grignard reagents. *Chem. Commun.* **2021**, *57*, 11807-11810.

[19] Gualandi, A.; Emer, E.; Guiteras Capdevila, M.; Cozzi, P. G. Highly Enantioselective α Alkylation of Aldehydes with 1,3-Benzodithiolylium Tetrafluoroborate: A Formal Organocatalytic α Alkylation of Aldehydes by the Carbenium Ion. *Angew. Chem. Int. Ed.* **2011**, *50*, 7842-7846.
[20] Sawama, Y.; Ogata, Y.; Kawamoto, K.; Satake, H.; Shibata, K.; Monguchi, Y.; Sajiki, H.; Kita, Y. Lewis Acid-Catalyzed Ring-Opening Functionalizations of 1,4-Epoxy-1,4-dihydronaphthalenes. *Adv. Synth. Catal.* **2013**, *355*, 517-528.
[21] Martínez-García, L.; Lobato, R.; Prado, G.; Monje, P.; Sardina, F. J.; Paleo, M. R. C-C Bond-Forming and Bond-Breaking Processes from the Reaction of Diesters with Me_3SnLi. Synthesis of Complex Bridged Polycycles and Dialkyl Aromatic Compounds. *J. Org. Chem.* **2019**, *84*, 1887-1897.
